# Safety and immunogenicity of PanChol, a single-dose live-attenuated oral cholera vaccine: results from a phase 1a, double-blind, randomised, placebo-controlled trial

**DOI:** 10.1016/S1473-3099(25)00682-6

**Published:** 2026-05

**Authors:** Deborah R Leitner, Stephen R Walsh, Masataka Suzuki, Michaël Desjardins, Alisse Hannaford, Amy C Sherman, Hannah Levine, Lena Carr, Elliot Hammerness, Akina Osaki, Emily Sullivan, Bryan Wang, George I Balazs, Jun Bai Park Chang, Damien M Slater, Nirajan Puri, Carole J Kuehl, Wilbur H Chen, Jason B Harris, Steven Piantadosi, Lindsey R Baden, Matthew K Waldor, PanChol study group, August Heithoff, Katherine G Dailey, Ruchika Dehinwal, Alexander A Morano, Kimberly A Dufresne, Lindsey A Parisi, Aidan Eustace, Bonnie Piantadosi, Xiaofang Li

**Affiliations:** a Division of Infectious Diseases, Brigham and Women's Hospital, Boston, MA, USA; b Department of Microbiology, Harvard Medical School, Boston, MA, USA; c Department of Medicine, Harvard Medical School, Boston, MA, USA; d Howard Hughes Medical Institute, Boston, MA, USA; e Centre Hospitalier de l'Université de Montréal, Montreal, QC, Canada; f Division of Infectious Diseases, Massachusetts General Hospital, Boston, MA, USA; g Department of Pediatrics, Harvard Medical School, Boston, MA, USA; h Center for Clinical Investigation, Brigham and Women's Hospital, Boston, MA, USA; i Center for Vaccine Development and Global Health, University of Maryland School of Medicine, Baltimore, MA, USA; j Department of Surgery, Brigham and Women's Hospital, Harvard Medical School, Boston, MA, USA

## Abstract

**Background:**

Killed whole-cell oral cholera vaccines can be used to prevent cholera but require multiple doses and have limited efficacy in young children. PanChol is a single-dose, live-attenuated, oral cholera vaccine derived from a current seventh pandemic *Vibrio cholerae* O1 strain. It co-expresses Inaba and Ogawa antigens, over-expresses the non-toxic cholera toxin B subunit, and is designed to minimise reactogenicity and be incapable of toxigenic reversion. We aimed to assess safety and immunogenicity of PanChol in a first-in-human trial.

**Methods:**

This phase 1a trial was conducted at the Brigham and Women's Hospital (Boston, MA, USA) and involved an open-label fixed dose-escalation module, followed by a randomised, double-blind, placebo-controlled dose-expansion module. Eligible participants were healthy adults aged 18–55 years without a previous *V cholerae* infection or cholera vaccination or a history of gastrointestinal disorders. In the open-label dose-escalation phase, eligible participants were enrolled into one of five cohorts receiving one dose of oral 10^6^–10^10^ colony-forming units (CFU) of PanChol. A dose de-escalation (10^4^ CFU and 10^5^ CFU) module was added after protocol amendment. In the subsequent randomised, double-blind module, participants were randomly assigned (7:7:4) to one of two dosing groups of one oral dose of PanChol (2 × 10^7^ CFU or 2 × 10^8^ CFU) or one oral dose of placebo (matching diluent). The list of assignments was generated from a custom program written by the statistician using blocked random assignments with a hidden block size (two blocks of 18). The co-primary outcomes were safety, including solicited, unsolicited, and serious adverse events following a single-dose of PanChol, and seroconversion (four-fold rise in titre over baseline) of vibriocidal titres to both Inaba and Ogawa *V cholerae* at 14 days post-vaccination (day 15). Safety was assessed in all participants who received the study product, and immunogenicity was assessed in all vaccine recipients who had samples available past day 7. Stool shedding of PanChol organisms was assessed as a secondary outcome in all participants. This trial is registered with ClinicalTrials.gov, NCT05657782, and is ongoing.

**Findings:**

Between Dec 13, 2022, and Feb 7, 2025, 57 healthy adults were enrolled, including 15 in the dose-escalation module (three in each group), six in the dose de-escalation module (three in each group), and 36 in the dose-expansion module (14 assigned to 10^7^ CFU PanChol, 14 to 10^8^ CFU PanChol, and eight to placebo); all participants received the allocated intervention. 27 (47%) of 57 participants were male, 30 (53%) were female, and the median age was 30·6 years (IQR 25·1–45·1); the majority were White (35 [61%]) and not Hispanic or Latino (51 [89%]). 34 (69%) of 49 PanChol recipients reported at least one solicited adverse event, compared with three (38%) of eight placebo recipients. Most solicited adverse events in PanChol recipients were mild and transient. The most common solicited adverse event was diarrhoea, reported in 19 (39%) of 49 PanChol recipients (15 mild and four moderate) and in three (38%) of eight recipients of placebo (one severe and two mild). In the dose-escalation and dose de-escalation modules, 18 (86%) of 21 participants had 39 unsolicited adverse events. In the randomised module, at least one unsolicited adverse event occurred in ten (71%) of 14 participants given 10^7^ CFU, in 12 (86%) of 14 participants given 10^8^ CFU, and in seven (88%) of eight placebo recipients. Most unsolicited adverse events were mild and only four were higher than grade 2, all of which were deemed unrelated to vaccination. One unsolicited adverse event was deemed related to vaccination (mild gassy sensation on day 3 in a 10^7^ CFU recipient). Shedding was detected in no placebo recipients, in one (33%) of three recipients of 10^4^ CFU PanChol, and in 44 (96%) of 46 recipients of at least 10^5^ CFU (two recipients of 10^8^ CFU did not shed PanChol). All 45 vaccinees given at least 10^5^ CFU PanChol who had samples available past day 7 seroconverted vibriocidal antibodies to both serotypes.

**Interpretation:**

A single oral dose of PanChol was safe and well tolerated at all doses and induced 100% vibriocidal seroconversion over a 100 000-fold dose range. These findings support the progression of PanChol into later phase clinical trials, including studies in endemic settings and in children.

**Funding:**

Wellcome Trust.

## Introduction

Vaccines are a key tool for the prevention and elimination of cholera, an acute and potentially lethal diarrhoeal disease.^1^ It is estimated that there are 1·3–4 million cholera cases and 21 000–143 000 related deaths annually, with approximately 1·3 billion people at risk of disease.^1^ Cholera is caused by ingestion of cholera toxin-producing strains of *Vibrio cholerae*, a curved Gram-negative rod. *V cholerae* has an unusual capacity to colonise the human small intestine. While proliferating in the small intestine, the pathogen secretes cholera toxin. Genes encoding this AB_5_-type toxin, *ctxAB*, are not an ancestral part of the *V cholerae* genome but instead are located within a prophage (CTXφ) that was acquired by horizontal gene transfer.^2^ Among diarrhoeal diseases, cholera is distinctive because it causes global pandemics in which a single strain becomes predominant throughout the globe. All recorded cholera pandemics, including the ongoing seventh pandemic, have been caused by the O1 serogroup, which is divided into two major serotypes—Inaba and Ogawa.

At present, the only cholera vaccines prequalified by WHO for use in cholera-endemic areas or in outbreaks are oral killed whole-cell vaccines that consist of killed Inaba and Ogawa *V cholerae*.^3^ Clinical trials have shown that these vaccines, which primarily represent oral administration of *V cholerae* lipopolysaccharide, confer an average two-dose protective efficacy of 55%, with some studies showing rapid waning of efficacy after 2 years.^4^ Although an advance for cholera control, these vaccines have important limitations, including the requirement of two doses for maximum efficacy and their limited efficacy (less than 30%) in children younger than 5 years, the group most susceptible to death from cholera.^4^

Studies of both naturally occurring and experimentally induced human cholera have revealed that infection stimulates protective immunity against subsequent cholera.^5^, ^6^ These observations have inspired the creation of live-attenuated cholera vaccines. Although previous live-attenuated vaccines, such as Vaxchora^7^ and Peru-15,^8^ showed promise as single-dose vaccines to engender robust and protective immune responses, including in children younger than 5 years,^9^, ^10^ they have important shortcomings. Vaxchora, which is licensed in the USA and the EU for travellers, was created in the now extinct classical *V cholerae* biotype and is capable of reversion to toxigenicity. Peru-15 was not pursued due to commercial reasons.

PanChol, named for pandemic cholera vaccine, is a novel live-attenuated cholera vaccine derived from the *V cholerae* O1 strain that is representative of the current pandemic El Tor *V cholerae* biotype, the cause of the vast majority of global cholera.^11^ PanChol was extensively engineered to ensure its biosafety for individuals and communities and protect its genetic stability while leaving intact its capacity for colonisation (appendix 1 p 10).^11^, ^12^ PanChol expresses both Inaba and Ogawa serotype antigens^12^ and was engineered to overexpress the B subunit of cholera toxin (CT-B) because immune responses to this non-toxic part of cholera toxin might confer some protection against cholera, as well as against diarrhoea due to enterotoxigenic *Escherichia coli*, which expresses a related toxin.^13^ In addition to deletion of *ctxA* and other potential toxins linked to reactogenicity,^14^ PanChol is resistant to toxigenic reversion. It contains deletions of the entire CTXφ prophage, including the site where the CTXφ genome integrates into the *V cholerae* chromosome, and *recA*, a gene linked to recombination. Moreover, a CRISPR system that recognises and degrades *ctxA* was introduced into the PanChol genome, rendering the vaccine strain resistant to reacquisition of this cholera toxin gene by means of horizontal gene transfer. In pre-clinical studies in infant rabbits and mice, PanChol was safe, immunogenic, and elicited protection in lethal challenge models.^12^ We carried out a first-in-human trial of a single dose of PanChol in healthy volunteers to assess its safety, tolerability, and immunogenicity.

**Research in context**

**Evidence before this study**

Cholera is a global public health threat; killed oral cholera vaccines (OCVs) are important for control. However, they have limited efficacy in young children and require multiple doses for maximum efficacy. Live-attenuated OCVs, similar to natural infection, can induce protective immunity with a single dose. Although other live-attenuated OCVs have been developed, none are WHO-prequalified. We searched PubMed from database inception to July 22, 2025, for studies evaluating OCVs using the terms “live oral cholera vaccine trial O1”, yielding 25 non-review clinical trial articles. The search was limited to the English language. All tested live vaccines were derived from the extinct classical *Vibrio cholerae* biotype (Vaxchora) or early El Tor biotype strains (Peru-15) and were Inaba serotype. None were engineered to be resistant to reversion to toxigenicity.

**Added value of this study**

This first-in-human trial establishes that PanChol, a live-attenuated OCV engineered from the current global pandemic El Tor *V cholerae* O1 strain, is safe and immunogenic in an adult population in the USA. Unlike previous live-attenuated vaccines, PanChol expresses both Inaba and Ogawa serotype antigens, is engineered for enhanced genetic stability, and resists toxigenic reversion. PanChol shedding, a marker for vaccine replication in the intestine, was detectable across doses 10^5^–10^10^ colony-forming units (CFU). Whole-genome sequencing of PanChol isolated from stool samples confirmed the vaccine's genomic stability. Across all doses, 100% of people who were vaccinated seroconverted to both Inaba and Ogawa serotypes 2 weeks after vaccination, showing potent immunogenicity.

**Implications of all the available evidence**

PanChol's favorable safety profile and immunogenicity support its additional development as a new agent for cholera control. Since PanChol is derived from the current pandemic strain, and natural infection stimulates more potent immunity to cholera than killed vaccines, PanChol might offer effective single-dose protection for children, a key and vulnerable population that is essential to target for cholera control. It might also be beneficial for reactive vaccination campaigns and for alleviating the global shortage of killed OCVs. However, further advancement of PanChol will require the development of large-scale manufacturing of an easily administrable, heat-stable product, feasible for deployment in cholera-endemic areas. Overall, these positive results warrant future studies of the vaccine's safety and immunogenicity in cholera-endemic settings and age de-escalation trials.

## Methods

### Study design

This phase 1a, first-in-human study was conducted at the Brigham and Women's Hospital (Boston, MA, USA). Participants were first enrolled in an open-label, fixed dose-escalation module to evaluate safety (range of 10^6^–10^10^ colony-forming units [CFU] PanChol). Before each log 10 dose-escalation from 10^6^ to 10^10^ CFU, the study team reviewed reactogenicity (solicited) and unsolicited adverse events reported by participants to ensure that no dose-limiting toxicities (defined as grade 3 in accordance with the FDA Toxicity Grading Scale for Healthy Adult and Adolescent Volunteers Enrolled in Preventive Vaccine Clinical Trials, September, 2007, and deemed related to vaccination) had occurred. An independent data and safety monitoring board was convened and reviewed unmasked data from the dose-escalation cohort before commencement of the randomised, double-blind, placebo-controlled module, in which participants were given 2 × 10^7^ CFU PanChol, 2 × 10^8^ CFU PanChol, or placebo. These doses were chosen based on preliminary assessment of immunogenicity and tolerability in the dose-escalation module and on previous studies of live-attenuated cholera vaccines.^7^, ^10^, ^15^, ^16^, ^17^ Since an interim analysis of the safety and immunogenicity data from the open-label dose-escalation phase revealed no dose-limiting toxicities over the tested dose range, a planned dose refinement module was removed. A dose de-escalation module with 10^4^ CFU and 10^5^ CFU PanChol groups was added via a protocol amendment following completion of the dose-expansion cohort to identify the minimum dose of PanChol that could lead to colonisation and immunogenicity. There was no patient or public involvement in study design, conduct, or reporting of the trial.

The research protocol (appendix 2) was approved by the Mass General Brigham Healthcare Institutional Review Board (2022P000461) and Institutional Biosafety Committee (2022B000015) and conducted under a US Food and Drug Administration (FDA) Investigational New Drug Application (28217). This study is registered with ClinicalTrials.gov, NCT05657782, and is ongoing.

### Participants

Healthy adults aged 18–55 years were recruited through the Mass General Brigham Rally volunteer portal. Volunteers were screened and enrolled if they were deemed medically healthy and met eligibility criteria. Participants were excluded if they had a history of gastrointestinal disorders, fever within 7 days, gastrointestinal illness or diarrhoea, previous *V cholerae* infection or cholera vaccination, abnormal stool patterns, recent antibiotic use or vaccine administration, or known allergy to doxycycline or related antibiotics. The complete inclusion and exclusion criteria are listed in appendix 1 (pp 3–4). Participants of childbearing potential agreed to use acceptable methods of contraception, as outlined by the study procedures, for at least 28 days before vaccination and until 3 months after receipt of the study product. Written informed consent was provided by all participants before enrolment. Sex (male or female) and gender (male, female, intersex, transgender, prefer not to disclose, or other) were self-reported by participants; sex was reported based on assigned biological sex at birth. Race (American Indian or Alaska Native; Asian; Black or African American; Native Hawaiian or Other Pacific Islander; White; or other) and ethnicity (Hispanic or Latino; or not Hispanic or Latino) were self-reported by participants.

### Randomisation and masking

Participants, three per dose, were first enrolled in an open-label fixed, dose-escalation module to 10^6^ CFU, 10^7^ CFU, 10^8^ CFU, 10^9^ CFU, or 10^10^ CFU. Then for the dose-expansion module, randomised assignments were generated in advance and placed into a Research Electronic Data Capture (REDCap) randomisation database.^18^ Assignments were dispensed one by one by Data Coordinating Centre (DCC) staff, under the Center for Clinical Investigation at Brigham and Women's Hospital, or by pharmacy staff using outlined procedures and REDCap's dedicated randomisation module. The list of assignments was generated from a custom program written by the DCC statistician in Mathematica (version 13)^19^ using blocked random assignments with a hidden block size (two blocks of 18). With this system, participants were randomly assigned (7:7:4) in a double-blind method to one of two dosing groups of PanChol (2 × 10^7^ CFU or 2 × 10^8^ CFU) or placebo (matching diluent).

Participants, investigators, monitors, and endpoint assessors were masked until all study visits were completed for the dose-expansion module. The pharmacists who dispensed and prepared the study product and the pharmacy monitor were not masked. PanChol and placebo were prepared in opaque amber bottles by the pharmacy to maintain masking.

### Procedures

After written informed consent was obtained and eligibility was confirmed, participants were enrolled and admitted to the Center for Clinical Investigation at the Brigham and Women's Hospital and dosed once with oral PanChol or placebo by study nurses. PanChol was produced under standards of good manufacturing practice at *Walter Reed Army Institute of Research* (Silver Spring, MD, USA; lot number 2081) and stored at –80°C. Vaccine viability, monitored via CFU enumeration, remained intact over the study duration. PanChol was diluted to the desired concentration in sodium bicarbonate buffer (2·5 g sodium bicarbonate, 1·6 g ascorbic acid, and 0·2 g lactose dissolved in 100 mL sterile water for irrigation) by study pharmacists and all doses were confirmed by independent titration by study laboratory members at the time of each volunteer dosing. Blood and stool samples were collected throughout the hospital period, vital signs were checked daily, and reactogenicity (solicited adverse events) and unsolicited adverse events were ascertained. Blood was taken for immunogenicity studies and for safety tests. Participants were administered doxycycline (initial dose 200 mg, followed by 100 mg twice a day for 4 days) 5 days after receipt of the study product to prevent potential shedding of PanChol organisms, and they were discharged 7 days after receipt of the study product if *V cholerae* stool cultures were negative. Participants with ongoing shedding on day 7 were discharged once a negative stool sample was documented. Follow-up occurred on days 15, 29, 57, 85, and 180, with additional blood and stool collections and unsolicited adverse event assessments. Serum antibodies and antibody-secreting cell responses were measured with a multiplex bead assay (Luminex). Stool samples were used to quantify PanChol bacteria, assess the genetic stability of PanChol after passage through the intestine, and assess PanChol related changes in the faecal microbiome (16S rRNA analysis). All unsolicited adverse events were collected up to day 29; medically attended adverse events, new onset chronic medical conditions, and serious adverse events were collected up to day 180. Adverse events were graded in accordance with the FDA Toxicity Grading Scale for Healthy Adult and Adolescent Volunteers Enrolled in Preventive Vaccine Clinical Trials, September, 2007. appendix 1 (pp 4–5) has details of laboratory procedures.

### Outcomes

The primary safety outcome was the incidence of solicited and unsolicited adverse events, including serious adverse events, following PanChol vaccination. The primary immunogenicity outcome was seroconversion (4-fold rise in titre over baseline) of vibriocidal titres to both Inaba and Ogawa *V cholerae* at 14 days post-vaccination (ie, day 15).

Secondary outcomes were the magnitude of pre-vaccination and post-vaccination serum vibriocidal titres to both Inaba and Ogawa *V cholerae*, as well as direct assessment of stool shedding of PanChol organisms using both quantitative and qualitative stool cultures. Exploratory outcomes were measurements of IgM, IgG, and IgA antibodies in serum; antibody-secreting cell responses (antibodies in lymphocyte supernatant [ALS]) targeting Inaba and Ogawa O-specific polysaccharides (OSP), CT-B, and toxin co-regulated pilus subunit A (TcpA; the major subunit of *V cholerae*'s chief intestinal colonisation factor); and PanChol-related changes in the faecal microbiome. Post-hoc analyses included slide agglutination to test serotype stability and whole-genome sequencing to assess the genetic stability of PanChol.

### Statistical analysis

No formal sample size calculation was performed. Based on experience from previous studies with other cholera vaccines, the chosen cohort sizes were considered sufficient to meet the objectives of the study while minimising unnecessary exposure. If no adverse events were observed in a cohort the size of our expansion cohort, the upper one-sided 97·5% exact binomial confidence bound on the event rate would be approximately 12%. Equal allocation was used between 10^7^ and 10^8^ CFUs PanChol because of the uncertainty of the optimal dose. Primary analysis for safety analyses included all participants who received the study product. The incidence of any adverse event (solicited, unsolicited, and serious adverse event) was determined. Numbers of participants with an adverse event, overall and by grade and relationship with vaccination, were calculated. For both dose-escalation and dose-expansion modules, immunogenicity data were presented as the frequency and magnitude of antibody responses (fold change between baseline and peak titre or peak titre) in all vaccinees who had samples available past day 7. Comparison of peak vibriocidal titres between participants who received PanChol doses 10^7^ CFU or 10^8^ CFU and those who received placebo were performed using the Mann–Whitney test with a two-sided 5% type I error rate. Stool shedding of PanChol organisms was assessed in all participants. Measurement of IgM, IgG, and IgA antibodies in serum and ALS were assessed in all participants who had samples available past day 7. PanChol-related changes in the faecal microbiome were assessed in stool samples from some participants in the dose escalation module and placebo recipients. Slide agglutination was performed on shed PanChol organism of PanChol vaccinees. Whole genome sequencing was assessed from faecal samples of five participants receiving 10^6^ (n=1), 10^7^ (n=3), and 10^8^ (n=1) CFU. GraphPad Prism (version 10) was used for all statistical analyses. Values for missing safety and immunogenicity data were not imputed and were excluded from the calculation of summary statistics for that datapoint. The dose-escalation and dose de-escalation modules were combined for analyses and are generally referred to together as the dose-escalation module for simplicity.

### Role of the funding source

The funder of the study had no role in study design, data collection, data analysis, data interpretation, or writing of the report.

## Results

**Figure 1:**
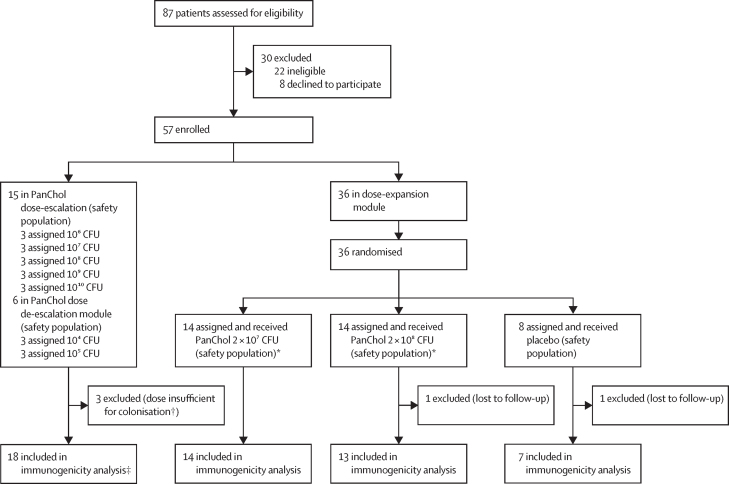
Trial profile CFU=colony-forming units. *One participant in each of these groups received doxycycline on day 3, withdrew early during the inpatient period, and were followed up as outpatients. †All three participants who received 10^4^ CFU were excluded from the immunogenicity analyses because studies of vaccine shedding suggest that a PanChol dose greater than 10^4^ CFU is required for colonisation. ‡Two participants were analysed only up to day 85 as day 180 samples were not yet available at the data cutoff for this study.

Between Dec 13, 2022, and Feb 7, 2025, 57 participants aged 18–55 years were enrolled, 15 in the dose-escalation module, six in the dose de-escalation module, and 36 in the dose-expansion module (figure 1). All participants received the assigned intervention and were assessed for safety. Two participants (one in the 2 × 10^8^ CFU PanChol dosing group and one in the placebo group) were lost to follow-up after the inpatient period and were therefore excluded from the immunogenicity analysis. 45 (92%) of 49 participants who received a dose of 10^6^ or higher or placebo completed the 6 months of follow-up; follow-up was not yet complete for the 10^4^ and 10^5^ dose recipients at the data cutoff for this study. Of 57 total participants, 27 (47%) were male, 30 (53%) were female, and the median age was 30·6 years (IQR 25·1–45·1); the majority of participants were White (35 [61%]) and not Hispanic or Latino (51 [89%]; table 1).

**Table 1:** Baseline characteristics

		Dose-escalation 10^4^–10^10^ CFU (n=21)*	**Dose-expansion**			**Total (n=57)**
			2 × 10^7^ CFU (n=14)	2 × 10^8^ CFU (n=14)	Placebo (n=8)	
Age at consent		37·1 (28·0–43·0)	29·1 (22·9–50·8)	28·1 (24·5–47·3)	29·1 (21·8–36·4)	30·6 (25·1–45·1)
Sex						
	Male	11 (52%)	5 (36%)	7 (50%)	4 (50%)	27 (47%)
	Female	10 (48%)	9 (64%)	7 (50%)	4 (50%)	30 (53%)
Gender						
	Male	10 (48%)	5 (36%)	7 (50%)	3 (38%)	25 (44%)
	Female	9 (43%)	4 (29%)	6 (43%)	3 (38%)	22 (39%)
	Transgender	2 (10%)	4 (29%)	1 (7%)	1 (13%)	8 (14%)
	Other	0	1 (7%)	0	1 (13%)	2 (4%)
Ethnicity						
	Hispanic or Latino	1 (5%)	2 (14%)	1 (7%)	1 (13%)	5 (9%)
	Not Hispanic or Latino	20 (95%)	12 (86%)	13 (93%)	6 (75%)	51 (89%)
	Not reported	0	0	0	1 (13%)	1 (2%)
Race						
	Asian	3 (14%)	0	4 (29%)	1 (13%)	8 (14%)
	Black or African American	7 (33%)	2 (14%)	2 (14%)	2 (25%)	13 (23%)
	White	11 (52%)	12 (86%)	8 (57%)	4 (50%)	35 (61%)
	Not reported	0	0	0	1 (13%)	1 (2%)

Data are median (IQR) or n (%). CFU=colony-forming units.

* The dose-escalation and dose de-escalation modules were combined for analyses and are generally referred to together as the dose-escalation module for simplicity.

**Figure 2:**
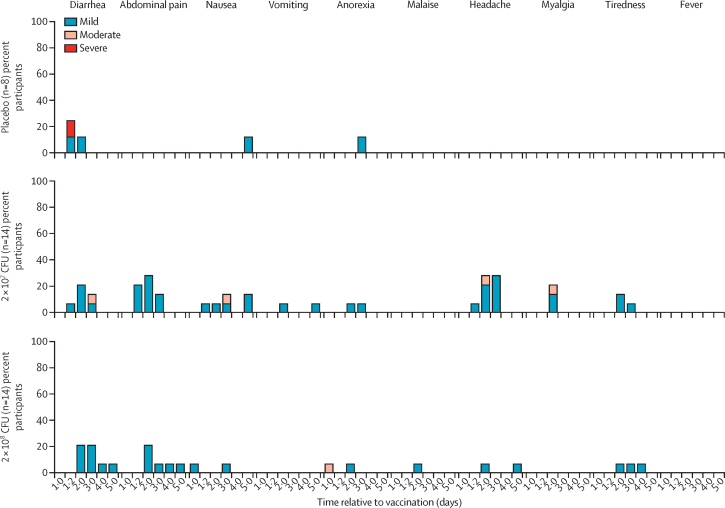
Solicited adverse events among study participants in the dose-expansion module Recorded adverse events from hospital admission (day 1) until administration of doxycycline (day 5) for participants dosed with placebo, 2 × 10^7^ CFU PanChol, or 2 × 10^8^ CFU PanChol. Day 1·2 represents approximately 4 h post-vaccination. Two study participants started doxycycline on day 3 (one dosed with 2 × 10^7^ CFU and one dosed with 2 × 10^8^ CFU). CFU=colony-forming units.

PanChol was safe and generally well-tolerated at all doses administered (figure 2; appendix 1 pp 7, 11–12). In the dose-escalation module, 20 (87%) of 23 solicited adverse events during the first 5 days of the inpatient period were graded as mild and three (13%) of 23 were graded as moderate (all moderate solicited adverse events were diarrhoea; one each in 10^5^ CFU, 10^7^ CFU, and 10^9^ CFU dose groups; appendix 1 pp 7). No dose-limiting toxicities were noted during dose-escalation.

In the dose-expansion module, 50 (89%) of 56 solicited adverse events in vaccine and placebo recipients were mild, with similar frequencies in recipients of the two vaccine doses (figure 2). Three (38%) of eight recipients of placebo had five solicited adverse events (four [80%] of five were mild) during the first 5 days of their inpatient period (figure 2). No severe solicited adverse events were reported among the 28 people vaccinated with PanChol in the dose-expansion module, supporting the hypothesis that at the lower dose (2 × 10^7^), the true frequency of solicited adverse events has a 97·5% probability of being equal to or less than 12%; the corresponding upper confidence bound for the higher dose (2 × 10^8^) is 23%. There was one severe solicited adverse event, diarrhoea, in a placebo recipient observed in the hours following administration of placebo buffer.

Among all 49 vaccine recipients, 15 (31%) had no solicited adverse events during the first 5 days of the inpatient period. In 34 (69%) vaccine recipients who had an adverse reaction, there were 74 solicited adverse events, 66 (89%) of which were graded as mild, with the remaining graded as moderate (figure 2; appendix 1 p 7). The most common solicited adverse event was diarrhoea (19 [39%] of 49 vaccine recipients, 15 mild and four moderate; three (38%) of eight placebo recipients, one severe and two mild). With one exception, solicited adverse events were transient and limited to 2 days.

47 (82%) of 57 participants reported 110 unsolicited adverse events during the study follow-up period (appendix 1 pp 13–18), including 18 (86%) of 21 participants in the dose-escalation phase (who reported 39 unsolicited adverse events) and ten (71%) of 14 people given the 10^7^ CFU dose, 12 (86%) of 14 people given the 10^8^ CFU dose, and seven (88%) of eight given placebo in the dose-expansion phase (who reported 27, 29, and 15 unsolicited adverse events, respectively). Only one unsolicited adverse event (mild gassy sensation) was deemed related to the vaccine (dose-expansion module, 10^7^ group, occurring on day 3 and resolving within 1 day). Of 109 unsolicited adverse events unrelated to treatment, 72 (66%) were graded mild (grade 1), 33 (30%) were moderate (grade 2), two (2%) were severe (grade 3), and two (2%) were graded as potentially life-threatening (grade 4). The four adverse events worse than grade 2 were: hospitalisation for depression (grade 3) in a recipient of 10^9^ PanChol in the dose-escalation module, occurring on day 27 after vaccination and considered a serious adverse event; increased aspartate aminotransferase (grade 3) in a placebo recipient in the dose-expansion module on day 11 after placebo administration; hospitalisation for depression (grade 4) in a recipient of 10^8^ PanChol in the dose-expansion module, occurring on day 39 after vaccination and considered a serious adverse event and graded as potentially life-threatening; and a fall (grade 4) in a 10^9^ PanChol recipient in the dose-escalation module, occurring on day 33 and graded as potentially life-threatening.

**Figure 3:**
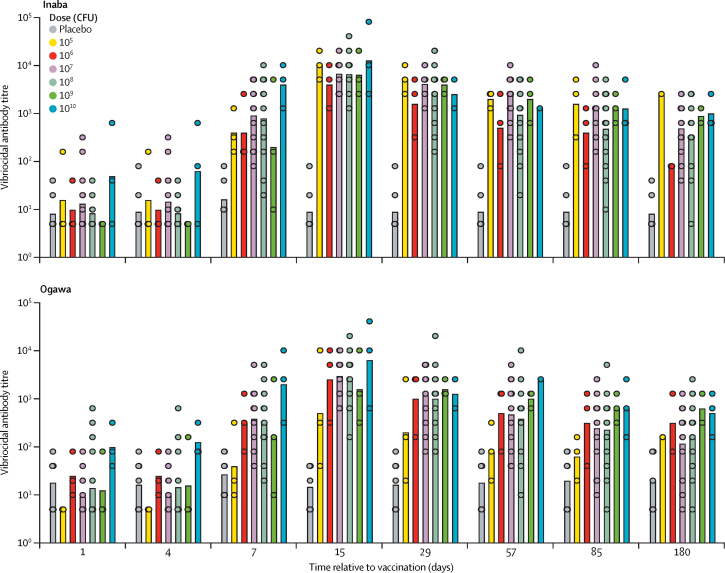
Serum vibriocidal antibody responses to Inaba and Ogawa *Vibrio cholerae* over time Sample sizes: day 0–day 85 n=52; day 85–180 n=50. Bars represent geometric means. Geometric means with 95% CI for 10^7^ and 10^8^ doses are available in appendix 1 (p 21). CFU=colony-forming units.

Serum vibriocidal antibody titres against isogenic Inaba and Ogawa *V cholerae* O1 strains^20^ were assessed to evaluate the immunogenicity of a single PanChol dose. Since analyses of vaccine shedding suggested that a PanChol dose greater than 10^4^ CFU was required for colonisation, we focused the immunogenicity analyses on all participants who received at least 10^5^ CFU. All vaccinees administered 10^5^–10^10^ PanChol CFU showed increases in vibriocidal antibody titres against both serotypes, including two vaccine recipients without detectable shedding in the 10^8^ dose group, 14 days post-vaccination, whereas no response was detectable in placebo recipients (figure 3; appendix 1 pp 19–20). All 45 (100%) vaccinees had an at least 4-fold increase (seroconversion) in vibriocidal antibody titres, comparing baseline with the individuals' peak responses, against both serotypes regardless of baseline titres (appendix 1 pp 19–20). Similar vibriocidal antibody titres were detected in all vaccinees over the 6-log dose range studied, suggesting that regardless of vaccine dose, vaccine replication in the intestine allowed similar immune responses.

Vaccine-induced vibriocidal titres became detectable at day 7 and generally peaked 14 days after vaccination (figure 3). At day 15, vibriocidal antibody responses to both serotypes were significantly induced compared with placebo recipients in vaccinees dosed with 10^7^ or 10^8^ CFU (p<0·0001; appendix 1 p 21). Titres remained significantly elevated up to at least 6 months post-vaccination in most participants and remained approximately 37-fold above baseline for Inaba (37·1 × for the 10^7^ CFU group and 39·9 × for the 10^8^ CFU group) and approximately 12-fold above baseline for Ogawa (12·8 × for the 10^7^ group and 12·4 × for the 10^8^ group; figure 3; appendix 1 p 21). Overall, PanChol induced similar vibriocidal responses to both serotypes, although Inaba antibody titres were numerically higher than Ogawa antibody titres: day 15 geometric mean titres (GMTs) for Inaba were 6811 (95% CI 4567–10 157) and 6640 (3815–11 555), and for Ogawa were 3013 (1778–5107) and 2451 (1241–4842) in response to the 10^7^ and 10^8^ CFU doses of PanChol, respectively (appendix 1 p 21). At most doses, both the mean individual peak serum vibriocidal antibody response (mean peak titre) and the mean peak fold increase in titre were approximately twice as great for Inaba compared with Ogawa (appendix 1 p 22).

**Figure 4:**
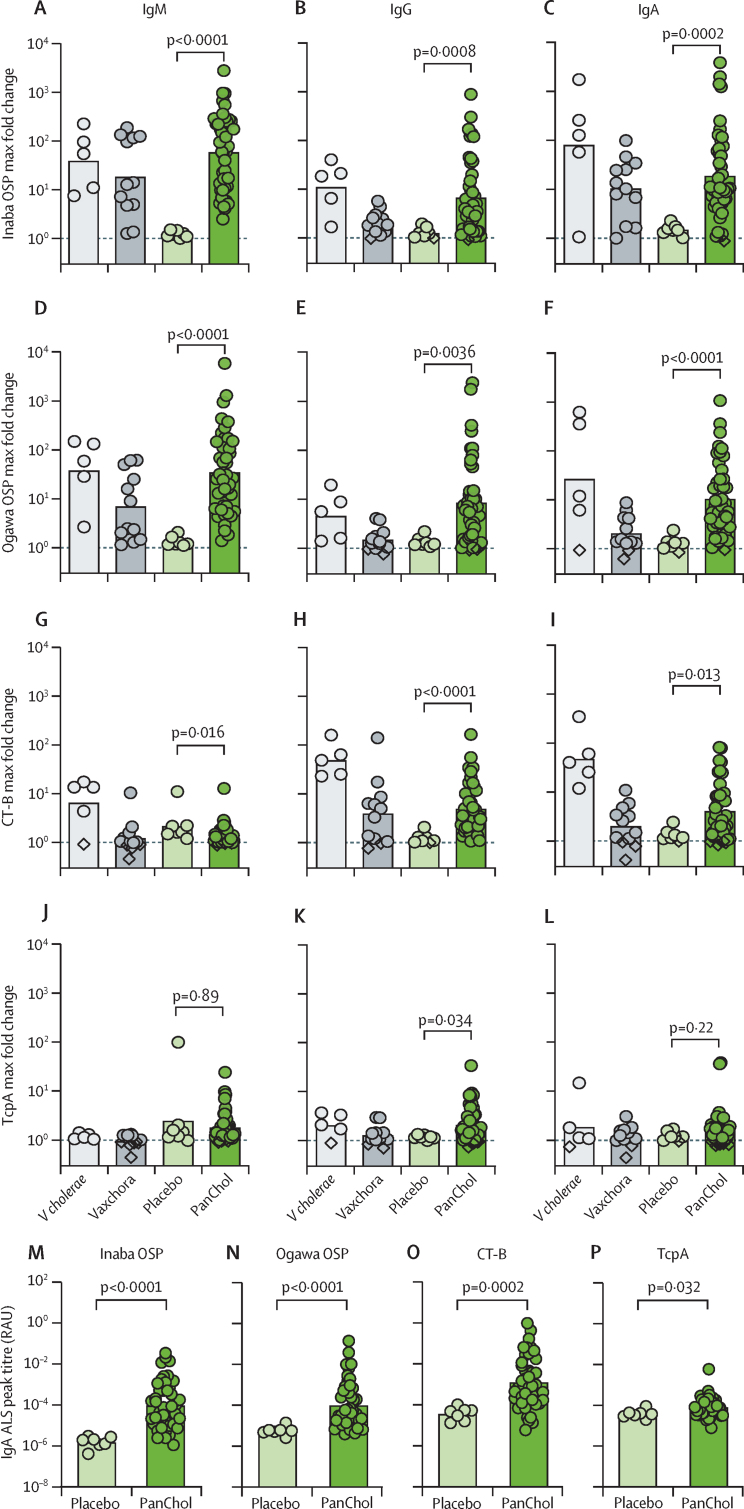
Antigen-specific and isotype-specific immune responses in serum and lymphocyte supernatant Antigen-specific and isotype-specific immune responses to Inaba OSP (A–C, M), Ogawa OSP (D–F, N), CT-B (G–I, O), and TcpA (J–L, P) in serum (A–L) and in lymphocyte supernatant (M–P). (A–L) Maximum fold-changes in antigen-specific and isotype-specific immune responses to the indicated antigens are shown. Biobanked serum samples from a previous trial of Vaxchora,^15^ a different live-attenuated cholera vaccine, were also analysed for peak antigen-specific and isotype-specific responses. The *Vibrio cholerae* group represents data from placebo participants who received the Inaba wild-type *V cholerae* challenge strain (N16961) in the Vaxchora trial.^15^ Values were calculated by comparing an individual's baseline titre to their peak response. If no baseline data (day 1) were available, the next reported data (day 4 or day 7) were used for the calculation. Closed circles represent an increase in the respective isotype-specific immune response, diamonds represent a fold change below 1, and the dotted line indicates a fold change of 1. Sample sizes: *V cholerae* n=5; Vaxchora n=12; PanChol placebo n=7; PanChol vaccine n=45. (M–P) Peak IgA responses to indicated antigens in lymphocyte supernatant of PanChol and placebo recipients. Values were calculated using an individual's peak response. Data are presented as RAU. Sample sizes: placebo n=7; PanChol n=45. In all panels, the PanChol group represents combined data from all vaccine recipients, and statistical comparisons of placebo versus PanChol were performed using a Mann–Whitney test. For all graphs, bars represent geometric means. Geometric means with 95% CI of placcebo and PanChol recipients are available in appendix 1 for serum (p 35) and antibodies in lymphocyte supernatant (p 48). ALS=antibodies in lymphocyte supernatant. CT-B=B subunit of cholera toxin. OSP=O-specific polysaccharides. RAU=relative antibody units. TcpA=toxin co-regulated pilus subunit A.

Antigen-specific and isotype-specific responses to Inaba and Ogawa OSP, CT-B, and TcpA in serum samples from recipients of vaccine and placebo were also monitored.^21^ In a post-hoc analysis, biobanked serum from a previous trial including serum samples from participants challenged with a virulent *V cholerae* Inaba strain (N16961) and recipients of Vaxchora, a different live-attenuated cholera vaccine,^15^ were analysed concurrently for comparison. Importantly, IgM, IgG, and IgA responses to Inaba and Ogawa OSP and CT-B subunit were detected in most vaccinees and were similar to those seen in experimentally induced cholera from participants of the Vaxchora challenge study (figure 4 A–I; appendix 1 pp 23–31, 35). Serum antibody responses to TcpA were not as potently induced compared with the other antigens (figure 4 J–L; appendix 1 pp 32–35). Compared with Vaxchora (an Inaba strain of classical *V cholerae*), PanChol induced more robust antibody responses to Ogawa-specific OSP and similar responses to Inaba-specific OSP (figure 4 A–F).

Antigen-specific ALS, a surrogate marker for mucosal immune responses,^22^ was also measured to evaluate antigen-specific and isotype-specific mucosal immune responses to PanChol. PanChol vaccinees had significantly induced IgA ALS responses to all four tested antigens compared with placebo (peak GMT × 10 000 for Inaba OSP 0·95 [95% CI 0·43–2·10], for Ogawa OSP 0·99 [0·45–2·20], for CT-B 13·00 [5·30–30·00], and for TcpA 0·81 [0·59–1·10]; figure 4 M–P; appendix 1 pp 38, 41, 44, 47–48). IgM ALS responses were restricted to Inaba and Ogawa OSP (peak GMT × 10 000 for Inaba OSP 7·90 [2·90–22·00] and for Ogawa OSP 10·00 [4·40–23·00]), whereas IgG ALS responses were confined to CT-B (peak GMT × 10 000 for CT-B 3·20 [1·40–7·50]; appendix 1 pp 8, 36–37, 39–40, 42–43, 45–46, 48). There were no discernible differences in the magnitude of antigen-specific and isotype-specific responses in serum or in lymphocyte supernatants in vaccinees who received either 10^7^ or 10^8^ CFU of PanChol (appendix 1 pp 49–50).

**Figure 5:**
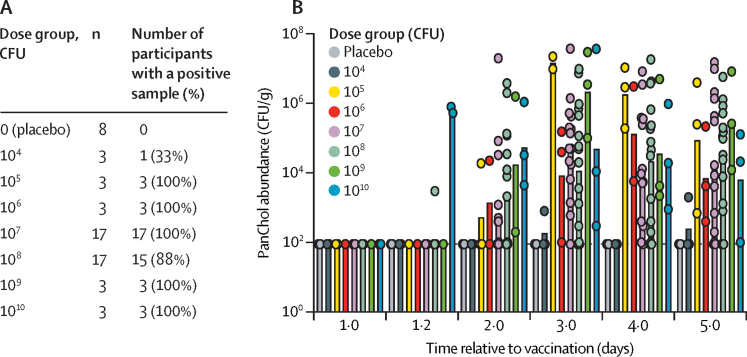
PanChol shedding in faeces (A) Number of participants in each dose group with PanChol detected in at least one stool culture within the first 4 days after vaccination. (B) The abundance of PanChol CFU in stool samples collected once daily during the first 4 days post-vaccination. The day 1·2 point represents approximately 4 h post-vaccination. Two study participants started doxycycline on day 3 (one in the 2 × 10^7^ group and one in the 2 × 10^8^ group) and no stool specimen was collected afterwards. The limit of detection is 100 CFU/g and no shedding is presented as 90 CFU/g. Bars indicate geometric means. CFU=colony-forming units.

After vaccination, daily PanChol shedding was monitored by counting CFU in faecal samples until day 5 when doxycycline was given. In the dose-escalation module, PanChol bacteria were detected in the stool of all vaccinees dosed with 10^5^–10^10^ CFU. However, only one of three recipients of 10^4^ CFU shed PanChol bacteria, suggesting that the minimum colonisation dose of PanChol when administered with buffer lies between 10^4^ and 10^5^ CFU. PanChol shedding was not detected in any placebo recipient and was detected in 44 (96%) of 46 participants who received doses of 10^5^–10^10^ CFU (figure 5A; appendix 1 p 51). Two recipients of the highest dose (10^10^ CFU) started excreting PanChol several hours after vaccination, probably representing direct passage of the vaccine through the intestine. At the other doses, faecal shedding generally became detectable 24 h post-vaccination, with similar numbers of PanChol CFU detected after 3 days (figure 5B; appendix 1 p 51). The absence of a correlation between vaccine dose and faecal CFU suggests that if PanChol establishes a replicative niche in the intestine, the abundance of vaccine shedding is independent of dose. In most vaccinees (37 [88%] of 42), shedding remained detectable throughout the inpatient period before doxycycline administration. The morning following the first dose of doxycycline administration, all stool cultures were PanChol negative in the hospital clinical microbiology laboratory, except for two samples that were culture negative later that day or the next day.

Although PanChol was detectable in faecal samples from most vaccinees by plating colonies on selective media, the vaccine organisms represented a small fraction of the faecal microbiota during the first 5 days post-vaccination and were only detectable in 16S rRNA analyses of faecal samples from recipients receiving 10^10^ PanChol CFU (appendix 1 p 9). Furthermore, the vaccine did not have a detectable effect on the composition of the microbiota based on analyses of 16S rRNA-sequences (appendix 1 p 9). Stool samples from nine vaccinees (one in the 10^6^ group, two in the 10^7^ group, three in the 10^8^ group, one in the 10^9^ group, and two in the 10^10^ group) and three placebo recipients showed minimal changes in participants' microbiota diversity during the inpatient period, with a similar highest dissimilarity index (Bray Curtis; appendix 1 p 9). Thus, PanChol intestinal colonisation appears to have a negligible effect on the intestinal microbial community.

Since PanChol is a bivalent vaccine expressing both the Inaba and Ogawa antigens, we evaluated whether the vaccine stably expressed both serotypes during its passage through the intestinal tract using slide agglutination assays with specific antisera targeting Inaba or Ogawa *V cholerae*. All tested colonies derived from faecal samples (44 [100%]), including through day 4 post-vaccination, agglutinated with both antisera, reflecting the stability of the bivalent phenotype.

Whole-genome sequencing was performed to probe genetic stability on samples from participants' stool obtained 3 days or 4 days following vaccination by comparison to the vaccine strain sequence. In samples prepared from five participants (one in the 10^6^ group, three in the 10^7^ group, and one in the 10^8^ group), only a single nucleotide polymorphism (at position 690612) was detected in one individual in the 10^7^ dosing group, suggesting that the PanChol genome is stable during passage through, and replication in, the human gut.

## Discussion

Cholera continues to threaten public health in many parts of the world, and vaccines are increasingly recognised as important tools for cholera control.^1^ Large-scale clinical trials have established the utility of killed whole-cell OCVs in reducing disease but have also revealed limited efficacy in young children.^23^, ^24^ However, natural infection with *V cholerae* generally leads to protection against subsequent disease, even in young children.^5^, ^25^ Protection from natural infection might also be more durable than that elicited by killed OCVs.^5^, ^6^ PanChol, a live vaccine engineered from a current wave three clinical isolate of El Tor *V cholerae*,^11^ was created to harness the superior immunogenicity engendered by *V cholerae* replication in the small intestine compared with that elicited by killed OCVs. Here, we report the first-in-human study of PanChol safety and immunogenicity in healthy adults. A single oral dose of PanChol was generally well tolerated and safe up to the highest administered dose of 10^10^ CFU. Vaccine shedding, a marker for vaccine replication in the intestine, was detectable across a dose range spanning from 10^5^ CFU to 10^10^ CFU. Notably, PanChol was immunogenic at all doses across this 100 000-fold dose range, eliciting seroconverting vibriocidal titres to both Inaba and Ogawa serotypes in every vaccinee.

PanChol had a similar reactogenicity profile to Peru-15, another El Tor-based live attenuated vaccine that underwent clinical trials in both the USA and low-income and middle-income countries (LMICs).^8^, ^10^, ^16^, ^17^ These results are in contrast to several early trials of different live-attenuated vaccines based on El Tor *V cholerae*, in which an unacceptable frequency of reactogenic diarrhoea was observed,^14^ even though these vaccines did not have *ctxA*, the enzymatic component of cholera toxin. Precise mechanistic understanding of the cause of the reactogenicity of these vaccines has been elusive, but subsequent studies have suggested a link between *V cholerae* motility and reactogenic diarrhoea. Peru-15, a non-motile Inaba El Tor-based vaccine did not cause clinically relevant reactogenic diarrhea.^8^, ^10^, ^16^, ^17^ PanChol is also non-motile; all five *V cholerae* flagellin genes were deleted from the PanChol genome because preclinical studies suggested that reactogenic diarrhoea was dependent on flagellins, perhaps due to TLR5-dependent inflammation.^26^

Fecal shedding of PanChol was detected in 44 (96%) of 46 recipients given at least 10^5^ CFU, indicating that the vaccine strain has a substantial capacity to overcome human barriers that can limit *V cholerae* infection and establish intestinal colonisation. Only one of the three recipients of 10^4^ CFU dose shed the vaccine, suggesting that the infectious dose of the vaccine lies between 10^4^ CFU and 10^5^ CFU, similar to the infectious dose for N16961, the wild-type El Tor *V cholerae* strain that has often been used as a challenge strain for testing vaccine efficacy.^27^ The high fraction of vaccinees who shed PanChol over a 100 000-fold dose range reflects the vaccine strain's capacity for intestinal colonisation and contrasts with that observed for Vaxchora, a live-attenuated vaccine created from a classical *V cholerae* strain and licensed in the USA for travellers, which was only shed by 11% of vaccinees receiving a dose of approximately 4 × 10^8^ CFU.^7^

In contrast to Vaxchora, PanChol was specifically engineered to be resistant to toxigenic reversion and to have genetic stability. Whole-genome sequencing confirmed in-vivo genetic stability despite intestinal replication. Furthermore, despite detectable shedding, microbiome analyses suggested minimal impact on the intestinal microbiome, indicating that the intestinal replication of the vaccine is probably not nearly as robust as that of wild-type *V cholerae*.^28^

PanChol's capacity for intestinal colonisation might underlie its potent immunogenicity given the substantial increase in vaccine antigens delivered during its replication in the human small intestine. PanChol led to 100% of vaccinees seroconverting vibriocidal titres to both *V cholerae* serotypes across doses of 10^5^ CFU to 10^10^ CFU. Such high levels of seroconversion to both serotypes have not been observed in previous trials of single-dose live attenuated OCVs or multiple-dose killed OCVs.^7^, ^8^, ^16^, ^29^ Geometric mean vibriocidal titres elicited by PanChol also compare favourably with those elicited by other live-attenuated vaccines tested in North Americans.^7^, ^8^, ^16^ Although PanChol expresses both Inaba and Ogawa antigens, vibriocidal titres were numerically higher against Inaba than Ogawa, suggesting that, at least in the context of PanChol vaccination, Inaba is more immunogenic. Nevertheless, the bivalent PanChol formulation appears to have been beneficial since, in a post-hoc analysis, IgM, IgG, and IgA anti-Ogawa OSP titres elicited by PanChol were numerically greater than those detected after immunisation with Vaxchora, an Inaba vaccine. There was also a tendency of PanChol to stimulate greater IgA titres targeting CT-B than Vaxchora. There is some debate about the importance of immunity to cholera toxin in protection from cholera, but large-scale clinical trials of a killed vaccine with the addition of CT-B (Dukoral) showed that this non-toxic subunit of cholera toxin enhanced short term protection from cholera as well enterotoxigenic *E coli*.^13^ However, infection with wild-type *V cholerae* stimulated greater IgA titres to CT-B than PanChol, potentially due to the adjuvant activity of CT-A. As observed in previous studies of immune responses in patients with cholera and with other live-attenuated vaccines, serum antibody and ALS titres to TcpA, the major subunit of *V cholerae*'s most important colonisation factor, were more modestly induced by PanChol.^30^ Such antibodies, which are not stimulated by killed whole-cell vaccines because TCP is not expressed, might provide augmented protection against cholera.^21^

Limitations of this study include its small size and inherent random unmeasured confounders, as well as conduct at a single institution in a non-endemic setting. Several live attenuated vaccines, including oral live-attenuated rotavirus vaccines, have shown reduced efficacy in LMICs.^31^ Therefore, a crucial next step in the development of PanChol will be to investigate its safety and immunogenicity in a cholera-endemic region. A phase 1b study (NCT07107516) of PanChol is being planned in Lusaka, Zambia, wherein participants will not receive doxycycline to clear the vaccine until 14 days after vaccination. This study will enable increased understanding of the duration of PanChol shedding, which was curtailed in the current study on day 5, and evaluation of whether longer duration of intestinal colonisation could increase immunogenicity. Future studies should assess the durability of immune responses elicited by PanChol beyond 6 months, test close contacts of vaccinees for the vaccine strain, and evaluate the safety and immunogenicity of PanChol in children—a key and vulnerable population that is essential to target for cholera control. Furthermore, future advancement of PanChol will require the development of large-scale manufacturing of a heat-stable, easily administrable product that will be feasible for deployment in cholera-endemic areas. Finally, the capacity of PanChol to induce rapid protection even before stimulating adaptive immunity, as observed in experimental animals,^11^ should be assessed in humans. The potential value of a second PanChol dose is currently under investigation at the Brigham and Women's Hospital.

A single-dose, oral, live-attenuated vaccine, such as PanChol, could become an important tool for global cholera control and close key gaps left by killed OCVs. In contrast to current killed OCVs and potential future investigational parental subunit vaccines, PanChol, a derivative of the currently circulating wave three El Tor *V cholerae*, will closely mimic natural infection and express nearly the full panoply of *V cholerae* antigens, while replicating in the small intestine. Moreover, unlike killed vaccines, which only have marginal efficacy in young children, natural infection stimulates more potent antibody and memory responses to Inaba and Ogawa OSP,^32^ which is thought to be the major protective antigen for immunity to cholera. A single-dose vaccine, such as PanChol, could also be beneficial given the shortage of killed OCVs and might be particularly valuable in reactive vaccination campaigns to curtail cholera outbreaks, thereby strengthening global cholera control efforts.

### Contributors

### Data sharing

De-identified individual participant data and the data dictionary or supporting clinical documents will be made available after publication for at least 5 years. Requests to access the data should be directed to the corresponding author along with a research proposal. Proposals will undergo scientific review by the PanChol protocol team, will require a signed data-access agreement, and might undergo institutional ethics committee review. The protocol for this Article is available in appendix 2 (pp 1–82).

## Declaration of interests

MKW is an inventor on a patent related to PanChol. SRW has received institutional grants or contracts from Sanofi Pasteur, Janssen Vaccines/Johnson & Johnson, Moderna Tx, Pfizer, AbbVie, F2G, Hookipa, Vir Biotechnology, and Worcester HIV Vaccine; has participated on data safety monitoring or advisory boards for Janssen Vaccines/Johnson & Johnson, CyanVac, CSL Behring, and BioNTech; and his spouse holds stock or stock options in Regeneron Pharmaceuticals. ACS reports consulting fees from Bain Capital. WHC reports being on the board of directors for the National Foundation of Infectious Diseases. SP has participated on data safety monitoring or advisory boards for the National Eye Institute/ Wilmer JHMI, Janssen Pharmaceuticals, and Cranius. LRB participated on data safety monitoring or advisory boards for National Institutes of Health (NIH) and the US Food and Drug Administration (FDA; Antimicrobial Drugs Advisory Committee), and is involved in HIV and SARS-CoV-2 vaccine clinical trials conducted in collaboration with the NIH, HIV Vaccine Trials Network, Covid Vaccine Prevention Network, International AIDS Vaccine Initiative, Crucell/Janssen, Moderna, Military HIV Research Program, the Gates Foundation, and Harvard Medical School. All other authors declare no competing interests.
