# Supplementary material for: Safety and immunogenicity of PanChol, a single-dose live-attenuated oral cholera vaccine: results from a phase 1a, double-blind, randomised, placebo-controlled trial
Source: Lancet Infect Dis. 2026 May;26(5):497–509. doi: 10.1016/S1473-3099(25)00682-6 (PMC13103297; doi:10.1016/S1473-3099(25)00682-6)
Supplement: Supplementary appendix 1 [file mmc1.pdf]

THE LANCET
Infectious Diseases

Supplementary appendix 1

This appendix formed part of the original submission and has been peer reviewed. We post it as supplied by the authors.

Supplement to: Leitner DR, Walsh SR, Suzuki M, et al. Safety and immunogenicity of PanChol, a single-dose live-attenuated oral cholera vaccine: results from a phase 1a, double-blind, randomised, placebo-controlled trial. *Lancet Infect Dis* 2026; published online Jan 7. https://doi.org/10.1016/S1473-3099(25)00682-6.

Safety and immunogenicity of PanChol, a single-dose live-attenuated oral cholera vaccine: results from a phase 1a, double-blind, randomised, placebo-controlled trial

Deborah R. Leitner, PhD [a,b*], Stephen R. Walsh, MDCM [a,c*], Masataka Suzuki, PhD [a,b], Michaël Desjardins, MD [a ‡], Alisse Hannaford, MD [a,c], Amy C. Sherman, MD [a,c], Hannah Levine, BS [a], Lena Carr, BA [a], Elliot Hammerness, BA [a], Akina Osaki, MD, PhD [a,b], Emily Sullivan, BSc [a,b], Bryan Wang, MS [a,b], George I. Balazs, BA[a,b], Jun Bai Park Chang, BS [a], Damien M. Slater, PhD [d,e], Nirajan Puri, MPH [f], Carole J. Kuehl, PhD [a], Prof. Wilbur H. Chen, MD [g], Prof. Jason B. Harris, MD [d,e], Prof. Steven Piantadosi, MD, PhD [h], Prof. Lindsey R. Baden, MD [a,c**], Prof. Matthew K. Waldor, MD, PhD [a,b,c,i**], on behalf of the PanChol study group [†]

[a] Division of Infectious Diseases, Brigham and Women's Hospital, Boston, MA, USA
[b] Department of Microbiology, Harvard Medical School, Boston, MA, USA
[c] Department of Medicine, Harvard Medical School, Boston, MA, USA
[d] Division of Infectious Diseases, Massachusetts General Hospital, Boston, MA, USA
[e] Department of Pediatrics, Harvard Medical School, Boston, MA, USA
[f] Center for Clinical Investigation, Brigham and Women's Hospital, Boston, MA, USA
[g] Center for Vaccine Development and Global Health, University of Maryland School of Medicine, Baltimore, MD, USA
[h] Department of Surgery, Brigham and Women's Hospital, Harvard Medical School, Boston, MA, USA
[i] Howard Hughes Medical Institute, Boston, MA, USA
[†] Members are listed in the supplementary appendix

* These authors contributed equally.
** These authors contributed equally.

Current address:
‡Centre Hospitalier de l'Université de Montréal, Montreal, QC, Canada

[¶] To whom correspondence should be addressed at:
Lindsey R. Baden, MD, Division of Infectious Diseases, Brigham and Women's Hospital, Boston, MA, USA, 02115, lbaden@bwh.harvard.edu

Matthew K. Waldor, MD, Division of Infectious Diseases, Brigham and Women's Hospital, Boston, MA, USA, 02115, mwaldor@bwh.harvard.edu

Table of Contents

PanChol study group 3

Full eligibility criteria 3

Vibriocidal antibody assay 4

Multiplex Bead Assay for isotype- and antigen-specific antibody responses 4

Antibody in lymphocyte supernatant (ALS) assay 4

Quantification of PanChol bacteria 5

PanChol genetic stability 5

16S rRNA amplicon sequence and analysis 5

Supplemental References 6

Figure S1: Solicited adverse events among study participants in the dose-escalation module. 7

Figure S2: Peak IgM and IgG specific immune responses in lymphocyte supernatant to Inaba (A, B), and Ogawa (C, D) specific polysaccharides (OSP), cholera toxin B subunit, CT-B (E, F) and TcpA (G, H). 8

Figure S3: Microbiome composition following PanChol ingestion. 9

Table S1: Genetic design features of PanChol 10

Table S2: Solicited adverse events reported from participants of the dose-escalation module during the inpatient period (day 1-7). 11

Table S3: Solicited adverse events reported from participants of the dose-expansion module during the inpatient period (day 1-7). 12

Table S4A: Unsolicited adverse events greater than grade 2 13

Table S4B: Unsolicited adverse events grade 2 and below 14

Table S5: Serum vibriocidal antibody responses (VAT) to Inaba and Ogawa *V. cholerae* O1. 19

Table S6: Kinetics of mean vibriocidal responses to both serotypes in serum of vaccine (10^7 and 10^8 CFU of PanChol) and placebo recipients 21

Table S7: Mean peak vibriocidal responses and mean peak fold increase in vibriocidal responses by dose group. 22

Table S8: Antigen- and isotype-specific immune responses in serum derived from vaccine and placebo recipients. 23

Table S9: Geometric mean peak fold increase in antigen- and isotype-specific immune responses to Inaba, and Ogawa OSP, CT-B and TcpA in serum of vaccine and placebo recipients. 35

Table S10: Antigen- and isotype-specific antibody responses in lymphocyte supernatant of vaccine and placebo recipients. 36

Table S11: Geometric mean peak antibody responses (RAU) to Inaba, and Ogawa OSP, CT-B and TcpA in lymphocyte supernatant of vaccine and placebo recipients. 48

Table S12: Geometric mean peak fold increase in antigen- and isotype-specific immune responses to Inaba, and Ogawa OSP, CT-B and TcpA in serum of vaccine (10^7 and 10^8 CFU of PanChol) and placebo recipients. 49

Table S13: Geometric mean peak antibody responses (RAU) to Inaba, and Ogawa OSP, CT-B and TcpA in lymphocyte supernatant of vaccine (10^7 and 10^8 CFU of PanChol) and placebo recipients. 50

Table S14: Quantification of PanChol bacteria in fecal samples derived from vaccine and placebo recipients. 51

PanChol study group

August Heithoff, BA; Katherine G. Dailey, BA; Ruchika Dehinwal, PhD; Alexander A. Morano, PhD; Kimberly A. Dufresne, BSc; Lindsey A. Parisi, BS; Aidan Eustace, BSc; Bonnie Piantadosi, MPH; Xiaofang Li, MD

Full eligibility criteria

To be eligible to participate in this study healthy volunteers must meet the following criteria within 56 days of Study Day 1 or at the time point specified in the individual eligibility criterion listed.

Inclusion Criteria

1. Must have given written informed consent (signed and dated) and any other authorizations required by local law and be able to comply with all study requirements.
2. Healthy adults aged from 18 to 55 years old.
3. Considered healthy, as judged by the clinical investigator, according to medical history, physical examination, vital signs, screening laboratories, and medication history.
4. Capable of understanding, consenting, and complying with the entire study protocol including the inpatient period.
5. Female participants must be non-pregnant and non-lactating and either
    a. surgically sterile (history of bilateral ligation, bilateral salpingectomy, bilateral oophorectomy, total hysterectomy) or postmenopausal (defined as as amenorrhea for at least 12 consecutive months before screening without an alternative medical cause)
    b. be of child-bearing potential and practicing an acceptable method of contraception or abstaining from all activities that could result in pregnancy for at least 28 days before vaccination until 3 months after receiving the IP.

    Acceptable methods of contraception include barrier methods (such as condom, diaphragm, or cervical cap used in conjunction with spermicide), intrauterine device, hormonal contraception (that may be taken or administered by oral, intravaginal, transdermal, subdermal or IM route), vasectomized partner (the vasectomized partner should be the sole partner for that participant)

Exclusion Criteria

1. Confirmed or suspected immunosuppressive condition, as a result of a disease (e.g., primary immune deficiency, malignancy, HIV infection) or have taken any systemic immunosuppressive therapy within 6 months of enrollment.
2. Pregnant or lactating women.
3. History of gastrointestinal (GI) disorder, such as previous major GI surgery, malabsorption, or any chronic GI disorders that would interfere, according to the investigator, with the IP.
4. Acute GI or febrile illness within 7 days of enrollment.
5. Have any acute or chronic medical condition that, in the opinion of the investigator, would make vaccination unsafe or interfere with the evaluation of immune response to study vaccination.
6. History of cholera vaccination.
7. History of cholera infection.
8. Abnormal stool pattern, defined as < 3 or >21 stools per week.
9. Serious allergic reaction to PanChol or placebo component (sodium bicarbonate, lactose, ascorbic acid)
10. Use of any systemic antibiotics within 1 month of PanChol administration.
11. Receipt of a live vaccine in the previous 4 weeks or planned in the 4 weeks following enrollment.
12. Receipt of a killed or subunit (non-live) vaccine in the previous 2 weeks or planned in the 2 weeks following enrollment.
13. Individuals who do not speak English will not be enrolled into this trial. This study involves more than minimal risk and no prospect of direct benefit for participants. Additionally, a subject who did not speak English may not be able to easily communicate safety concerns in a timely fashion to the study investigators
14. Childcare workers with direct contact with children ≤ 2 years of age
15. Individuals whose occupation involves handling of food

16. Healthcare workers who have direct contact with patients who are immunodeficient, HIV-positive, or have an unstable medical condition
17. Use laxatives regularly
18. Have diarrhea within 48 hours before enrollment
19. Have a history of hypersensitivity to any of the tetracyclines
20. Have a history of hypersensitivity to streptomycin or any aminoglycoside due to the known cross-sensitivity of patients to drugs in this class.
21. Individuals who have a household member who are immunodeficient, HIV-positive, or have an unstable medical condition.

Vibriocidal antibody assay

Serum samples were collected before vaccination and on day 4, 7, 15, 29, 57, 85 and 180 days post vaccination. Quantification of serum vibriocidal antibodies using the isogenic Inaba and Ogawa ZChol *V. cholerae* strain*s* as targets was performed as described[1,2]. Briefly, the ZChol strains were grown in brain heart infusion (BHI) media (BD Difco), harvested and resuspended in sterile normal saline. A mixture of guinea pig complement (Sigma) and the respective target strains was added to a 96-well plate containing serial dilutions of heat-inactivated serum samples from vaccine and placebo recipients (1:10 starting dilution). After 60 min at 37°C, BHI was added to the wells which were then incubated at 37°C to assess the vibriocidal antibody activity. Bacterial growth was measured using the BioTek Epoch2 microplate reader at an optical density of 595 nm. The titers of vibriocidal Inaba and Ogawa antibodies were reported as the highest dilution of serum resulting in a ≥50% reduction in target optical density compared to control wells without serum. A titer of 5 was assigned when no vibriocidal antibody responses were detected. The mouse monoclonal antibody 432A.1G8.G1.H12 targeting *V. cholerae* O1 OSP[3] was used as a positive control; a ≥4-fold increase in titer relative to baseline was defined as seroconversion.

Multiplex Bead Assay for isotype- and antigen-specific antibody responses

Serum samples were diluted 100-fold (final test concentration of 1:1000). Samples were tested in duplicate on a FlexMap3D instrument (Luminex, Austin, Texas; xPonent software v.4.3.1). A standard curve, prepared from a pool of convalescent-phase cholera patient sera, was run on each plate and relative antibody units (RAU) were predicted from averaged NetMFI values, using 4-parameter logistic regression (GraphPad Prism, v.10.4.0). A detailed version of the protocol can be found at: **dx.doi.org/10.17504/protocols.io.3byl4b1x8vo5/v2**

A random selection of anonymized samples from a previously reported clinical trial (http://clinicaltrials.gov/show/NCT01895855), during which consent was obtained for secondary use in additional immunologic evaluations, were included for comparison. Samples were selected from 3 cohorts: Day 1 and 39 samples from a placebo group challenged at Day 11, Day 1 and Day 11 samples from a vaccinated group challenged at Day 11, and Day 1, Day 11, and Day 91 samples from a vaccinated group challenged at Day 91[4].

Antibody in lymphocyte supernatant (ALS) assay

Participant blood samples (~24 mL) were collected and mixed in a sodium citrate CPT tube (BD Vacutainer 362761). After centrifugation at 1,800 × g for 30 min the plasma was removed by aspiration. The cells were transferred to a 50 mL conical tube and washed twice in 45 mL PBS centrifugated at 300 × g for 10 min. The cells were resuspended in 10 mL of PBS, and live cells were counted using 3% Acetic acid with methylene blue (Stem Cell Technologies, 07060) and a hemocytometer. After centrifugation at 300 x g for 10 min, cells were resuspended in fetal bovine serum (FBS) at a concentration of 10 million cells/mL and were kept at 4 °C Ten million cells were centrifuged at 300 × g for 10 min and then resuspended in 1 mL of RPMI1640 medium (Gibco, 11875119) containing 10% FBS, 50 mg/mL gentamicin (Gibco, 15710-072) and cultured in a 24-well plate in 5% CO_2 incubator at 37 °C for 48 hours. The culture supernatant was collected, centrifuged at 300 × g for 10 min to remove cells, and stored at -80 °C until subsequent Luminex analysis, where they were analyzed at 10-fold, and when needed, 2-fold dilutions.

Quantification of PanChol bacteria

Vaccine: Every vaccine dose was independently titrated by plating on Luria-Bertani agar containing streptomycin (200 µg/mL) and X-gal (40µg/mL) to determine the number of PanChol CFU per 100 ml.

Stool sample: During the inpatient period (day 1-5), one stool sample per day per study participant was collected and serial dilutions were directly plated without enrichment on thiosulfate-citrate-bile salts-sucrose (TCBS) agar (Sigma) containing streptomycin (200 µg/mL) to determine the number of PanChol CFU per gram of stool. Collected stool samples were stored at room temperature and plated within an hour after collection. If no stool specimen was produced, a rectal swab was obtained and streaked on TCBS plates to monitor for the presence of PanChol. During the inpatient period, PanChol CFU isolated from fecal samples were tested for agglutination with specific antisera targeting Inaba or Ogawa *V. cholerae* (Difco Laboratories).

PanChol genetic stability

Genomic DNA was isolated from PanChol CFU from the oral suspension used for vaccination and from fecal samples obtained three- or four-days following vaccination with 10^6 (n=1), 10^7 (n=3) and 10^8 (n=1) CFU using the GeneJet gDNA Isolation Kit (Thermo Fisher Scientific). Whole genome sequencing was carried out at the SeqCenter, Pittsburgh. Reads were mapped to the PanChol genome (PRJNA793890) with Snippy v4.6.0 (https://github.com/tseemann/snippy) using freebayes v1.3.6 (https://github.com/freebayes/freebayes) with a minimum read coverage of 4, minimum base quality of 13, a mapping quality of 60, and a requirement of 75% read concordance[5]. Differences in the sequences of the genomes of the ingested and shed vaccine were identified by comparing the mapping results of the ingested PanChol genome with those from the shed PanChol genome.

16S rRNA amplicon sequence and analysis

Stool samples were collected from participants and stored at -80°C until use. Stool samples from participants in the dose-escalation module (dosing groups (CFU): 10^6 (n=1), 10^7 (n=2), 10^8 (n=3), 10^9 (n=1), 10^{10} (n=2)) and from three placebo recipients were used for 16S rRNA sequencing if pre-vaccine comparison samples were available. Bacterial DNA was extracted from ~250mg of stool using DNeasy PowerLyzer PowerSoil Kit (Qiagen: 12855-50) and purified with a spin column. The V3-V4 region of 16S rRNA was PCR amplified from 12·5 ng of genomic DNA using Phusion DNA polymerase (New England Biolab: M0531S) with 341F+adaptor (5'-TCG TCG GCA GCG TCA GAT GTG TAT AAG AGA CAG CCT ACG GGN GGC WGC AG-3') and 805R+adaptor (5'-GTC TCG TGG GCT CGG AGA TGT GTA TAA GAG ACA GGA CTA CHV GGG TAT CTA ATC C-3') primers. PCR amplicons were purified using Ampure XP beads (Beckman Coulter: No: A63881) and were subjected to a 2nd PCR using Phusion DNA polymerase with the Nextera XT Index Kit v2 Set A and D primers (Illumina). Each PCR amplicon was purified using Ampure XP beads and the concentration of the purified amplicon was determined with Qubit. Paired-end reads (301 cycles each) of a 650 pM pooled amplicon mix was sequenced using a NextSeq™ 1000/2000 P1 XLEAP-SBS™ Reagent Kit (600 Cycles).

Sequencing results were analyzed using Qiime2 version 2025.5[6]. Briefly, fastq files were demultiplexed and adaptor sequences were trimmed using cutadapt. Forward and reverse sequences were merged using the "v-search merge-pairs" command, and low-quality reads were filtered with the "quality-filter q-score" command with default settings. Sequences were denoised using "qiime deblur denoise-16S" function with a parameter "--p-trim-length 401", since >98% of sequences were more than 401 nt in length with quality scores of 40. Sequence read depth in all samples ranged from 64,841 to 320,337. Shannon index was calculated using the "qiime2 diversity core-metrics-phylogenetic" command with sampling-depth 64800. Bray-Curtis distance was calculated with the "qiime2 diversity beta-group-significance" command.

Supplemental References

1. Son MS, Taylor RK. Vibriocidal assays to determine the antibody titer of patient sera samples. *Curr Protoc Microbiol* 2011; **Chapter 6**: Unit6A 3.
2. Sit B, Fakoya B, Zhang T, Billings G, Waldor MK. Dissecting serotype-specific contributions to live oral cholera vaccine efficacy. *Proc Natl Acad Sci U S A* 2021; **118**(7).
3. Sayeed MA, Bufano MK, Xu P, et al. A Cholera Conjugate Vaccine Containing O-specific Polysaccharide (OSP) of V. cholerae O1 Inaba and Recombinant Fragment of Tetanus Toxin Heavy Chain (OSP:rTTHc) Induces Serum, Memory and Lamina Proprial Responses against OSP and Is Protective in Mice. *PLoS Negl Trop Dis* 2015; **9**(7): e0003881.
4. Mayo-Smith LM, Simon JK, Chen WH, et al. The Live Attenuated Cholera Vaccine CVD 103-HgR Primes Responses to the Toxin-Coregulated Pilus Antigen TcpA in Subjects Challenged with Wild-Type Vibrio cholerae. *Clin Vaccine Immunol* 2017; **24**(1).
5. Rubin DHF, Zingl FG, Leitner DR, et al. Reemergence of Cholera in Haiti. *N Engl J Med* 2022; **387**(25): 2387-9.
6. Bolyen E, Rideout JR, Dillon MR, et al. Reproducible, interactive, scalable and extensible microbiome data science using QIIME 2. *Nat Biotechnol* 2019; **37**(8): 852-7.

Figure S1: Solicited adverse events among study participants in the dose-escalation module. Shown are recorded adverse events from hospital admission (day 1) until administration of doxycycline (day 5) for recipients of PanChol doses from 10^4 to 10^{10} CFU. The 1·2 point is ~4hr post-vaccination. Solicited adverse events included diarrhea, abdominal pain, nausea, vomiting, anorexia, malaise, headache, myalgia, tiredness and fever. Grading of adverse events is indicated by color; blue: mild; yellow: moderate.

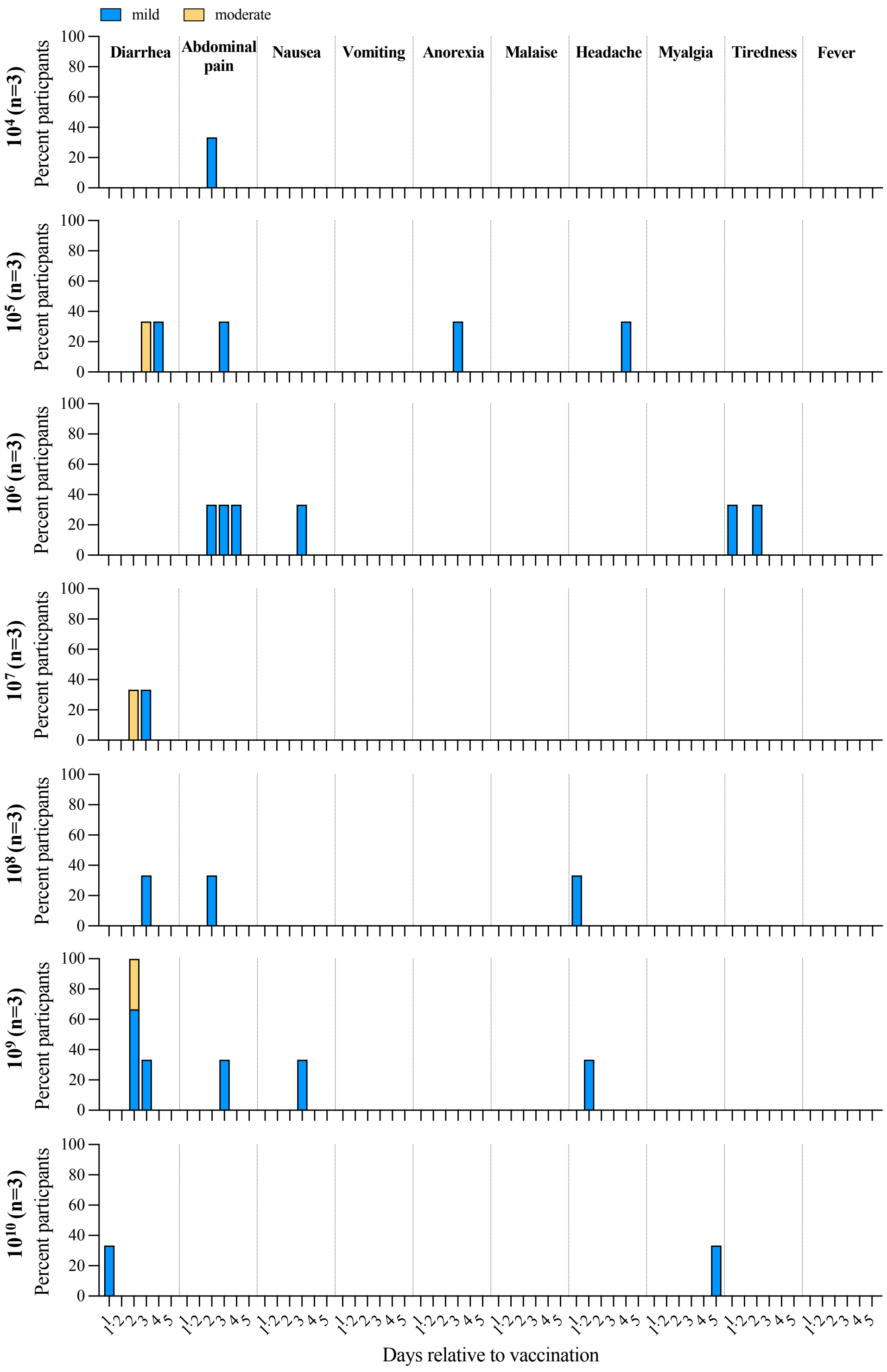

Figure S2: Peak IgM and IgG specific immune responses in lymphocyte supernatant to Inaba (A, B), and Ogawa (C, D) specific polysaccharides (OSP), cholera toxin B subunit, CT-B (E, F) and TcpA (G, H). PanChol group, combined data of all vaccine recipients. Statistical comparison of Placebo vs PanChol was performed using a Mann-Whitney test. Placebo: ≥4; PanChol: ≥41. For all graphs, bars represent geometric means, and data is presented as relative antibody units (RAU).

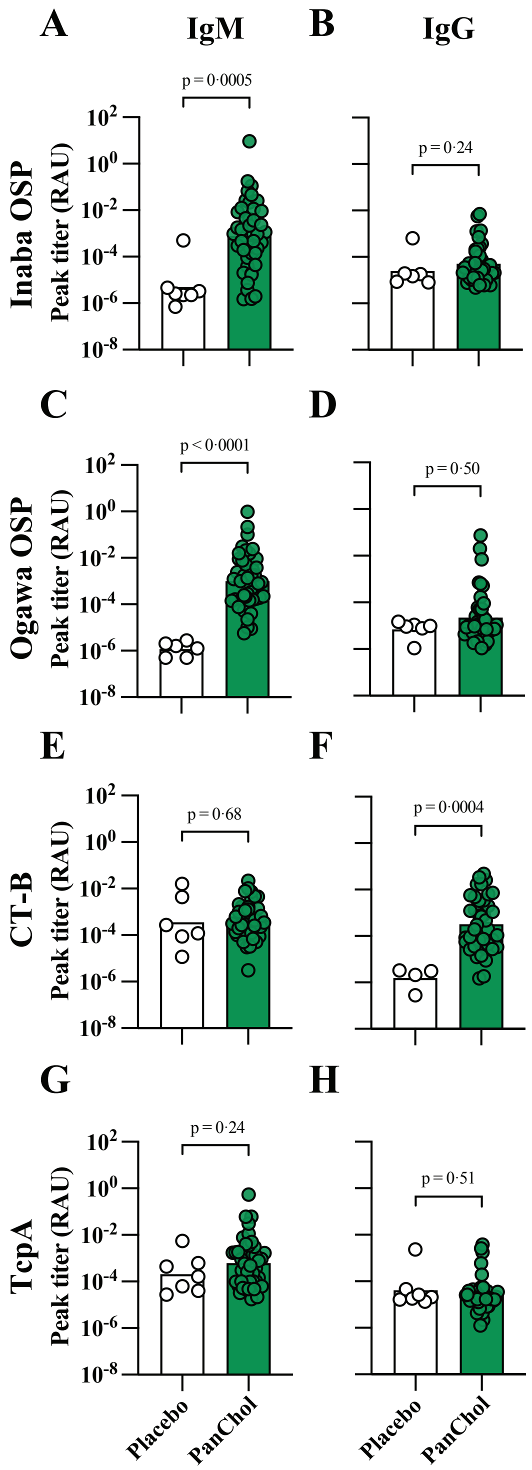

Figure S3: Microbiome composition following PanChol ingestion. A) Fraction of Vibrio species among total sequencing reads. B) Change in the Shannon index in stool samples from individual participants; follow-up is day 180. C) Maximal Bray-Curtis dissimilarity index based on comparison of a pre-dose stool specimen to samples during the inpatient period. Data is represented as mean with standard deviation. Stool samples from participants in the dose-escalation module (dosing groups (CFU): 10^6 (n=1), 10^7 (n=2), 10^8 (n=3), 10^9 (n=1), 10^{10} (n=2)) and from three placebo recipients were analyzed.

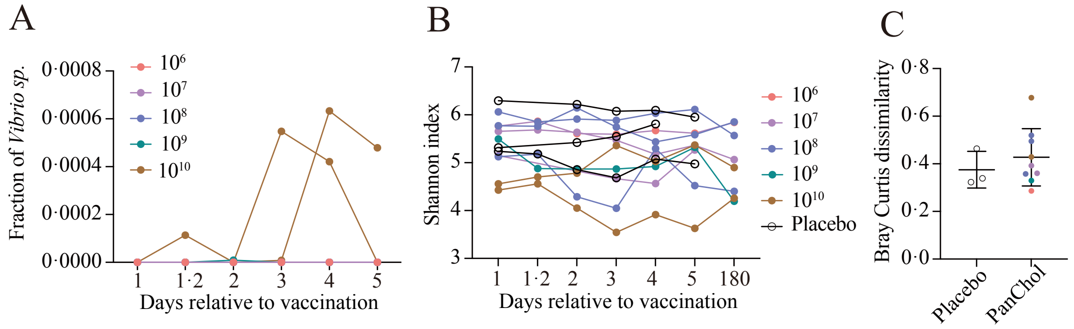

Table S1: Genetic design features of PanChol

Design principles	Genetic features of PanChol
**Target antigens**	
Globally predominant strain	A derivative of 2010 Haiti outbreak
Bivalent expression of Inaba and Ogawa serotypes	Hypomorphic WbeT methylase
Provide short-term protection vs ETEC	Overexpression of CT-B (*ctxB*)
**Safety**	
Remove capacity to make cholera toxin	Deletion of *ctxAB* and entire CTX prophage
Eliminate reactogenicity	Deletion of flagellins, *hylA* and *rtxA*
Sensitive to antibiotics, incapable of transfer	Deletion of most of SXT mobile element
**Genetic stability (no reversion)**	
Eliminate capacity to regain CTX phage	Deletion of CTX phage attachment site
Prevent acquisition of *ctxA* gene	CRISPR targets *ctxA* for destruction
Eliminate capacity for recombination	Deletion of *recA*

Table S2: Solicited adverse events reported from participants of the dose-escalation module during the inpatient period (day 1-7).

	No. of subjects with symptom/total no. of subjects in the dosing group during the inpatient period (dose-escalation module)						
**Symptom**	**10^4 CFU**	**10^5 CFU**	**10^6 CFU**	**10^7 CFU**	**10^8 CFU**	**10^9 CFU**	**10^{10} CFU**
Diarrhea							
Mild grade	0/3	0/3	0/3	1/3	1/3	2/3	1/3
Moderate grade	0/3	1/3	0/3	1/3	0/3	1/3	0/3
Severe grade	0/3	0/3	0/3	0/3	0/3	0/3	0/3
Abdominal pain							
Mild grade	1/3	1/3	2/3	0/3	1/3	1/3	0/3
Moderate grade	0/3	0/3	0/3	0/3	0/3	0/3	0/3
Nausea							
Mild grade	1/3	0/3	2/3	1/3	0/3	1/3	0/3
Moderate grade	0/3	0/3	0/3	0/3	0/3	0/3	0/3
Vomiting							
Mild grade	0/3	0/3	1/3	1/3	0/3	0/3	0/3
Moderate grade	0/3	0/3	0/3	0/3	0/3	0/3	0/3
Anorexia							
Mild grade	0/3	1/3	0/3	0/3	0/3	0/3	0/3
Moderate grade	0/3	0/3	0/3	0/3	0/3	0/3	0/3
Malaise							
Mild grade	0/3	0/3	0/3	0/3	0/3	0/3	0/3
Moderate grade	0/3	0/3	0/3	0/3	0/3	0/3	0/3
Headache							
Mild grade	0/3	1/3	0/3	0/3	1/3	1/3	0/3
Moderate grade	0/3	0/3	0/3	0/3	0/3	0/3	0/3
Myalgia							
Mild grade	0/3	0/3	0/3	0/3	0/3	0/3	1/3
Moderate grade	0/3	0/3	0/3	0/3	0/3	0/3	0/3
Tiredness							
Mild grade	0/3	0/3	2/3	0/3	0/3	0/3	0/3
Moderate grade	0/3	0/3	0/3	0/3	0/3	0/3	0/3
Fever							
Mild grade	0/3	0/3	0/3	0/3	0/3	0/3	0/3
Moderate grade	0/3	0/3	0/3	0/3	0/3	0/3	0/3

Table S3: Solicited adverse events reported from participants of the dose-expansion module during the inpatient period (day 1-7).

	**No. of subjects with symptom/total no. of subjects in group during the inpatient period (dose-expansion module)**		
**Symptom**	**Placebo**	**$2{\times}10^7$ CFU**	**$2{\times}10^8$ CFU**
Diarrhea			
Mild grade	2/8	4/14	6/14
Moderate grade	0/8	1/14	0/14
Severe grade	1/8	0/14	0/14
Abdominal pain			
Mild grade	0/8	5/14	5/14
Moderate grade	0/8	0/14	0/14
Nausea			
Mild grade	2/8	7/14	4/14
Moderate grade	0/8	1/14	0/14
Vomiting			
Mild grade	1/8	2/14	0/14
Moderate grade	0/8	0/14	0/14
Anorexia			
Mild grade	1/8	2/14	1/14
Moderate grade	0/8	0/14	1/14
Malaise			
Mild grade	0/8	0/14	2/14
Moderate grade	0/8	0/14	0/14
Headache			
Mild grade	0/8	6/14	2/14
Moderate grade	0/8	2/14	0/14
Myalgia			
Mild grade	0/8	2/14	0/14
Moderate grade	0/8	1/14	0/14
Tiredness			
Mild grade	0/8	2/14	3/14
Moderate grade	0/8	0/14	0/14
Fever			
Mild grade	0/8	0/14	0/14
Moderate grade	0/8	0/14	0/14

Table S4A: Unsolicited adverse events greater than grade 2

Pub-ID	Dose	Module	Verbatim Term	Days since vaccination	Maximum Severity Grade[1]	Relatedness
100-08	10^9	1[2]	Fall	33	4	Not related
100-12	10^9	1	Depression - SAE	27	3	Not related
100-44	2×10^8	3[3]	Major Depression Episode - SAE	39	4	Not related
100-40	Placebo	3	Increased AST	11	3	Not related

[1]Graded according to the FDA Toxicity Grading Scale for Healthy Adult and Adolescent Volunteers Enrolled in Preventive Vaccine Clinical Trials (September 2007); [2]dose-escalation module; [3]dose-expansion module

Table S4B: Unsolicited adverse events grade 2 and below

Pub-ID	Dose	Module	Verbatim Term	Days since vaccination	Maximum Severity Grade[1]	Relatedness
100-01	10^6	1[2]	Tachypnea	2	1	Not related
100-01	10^6	1	Nausea and Vomiting	5	1	Not related
100-01	10^6	1	Menstrual Cramps	3	1	Not related
100-02	10^6	1	Tachypnea	2	1	Not related
100-02	10^6	1	Elevated Bilirubin	3	1	Not related
100-02	10^6	1	Chills	0	1	Not related
100-02	10^6	1	Sprained ankle - Right	130	2	Not related
100-03	10^6	1	Tachypnea	2	1	Not related
100-04	10^7	1	Diarrhea	0	2	Not related
100-04	10^7	1	Tachypnea	2	1	Not related
100-04	10^7	1	Tachypnea	5	1	Not related
100-05	10^8	1	Tachypnea	1	1	Not related
100-06	10^7	1	Nausea and Vomiting	5	1	Not related
100-06	10^7	1	Elevated Absolute Eosinophil Count	6	1	Not related
100-07	10^7	1	Increased ALT	6	2	Not related
100-09	10^8	1	Thyroid Nodule	43	1	Not related
100-10	10^8	1	Systolic Hypertension	0	2	Not related
100-10	10^8	1	Systolic Hypertension	4	2	Not related
100-11	10^9	1	Systolic Hypertension	2	1	Not related
100-11	10^9	1	Elevated ALT	6	1	Not related
100-11	10^9	1	Elevated AST	6	1	Not related
100-13	10^{10}	1	Decreased Absolute Neutrophil Count	3	1	Not related
100-13	10^{10}	1	Decreased White Blood Cell Count	3	1	Not related
100-19	2×10^8	3[3]	Tachypnea	1	1	Not related
100-19	2×10^8	3	Increased Total Bilirubin	1	1	Not related

Pub-ID	Dose	Module	Verbatim Term	Days since vaccination	Maximum Severity Grade[1]	Relatedness
100-19	2×10^8	3	Decreased Absolute Neutrophil Count	3	2	Not related
100-19	2×10^8	3	Decreased Heart Rate	4	2	Not related
100-20	2×10^7	3	Decreased Absolute Neutrophil Count	3	1	Not related
100-20	2×10^7	3	Upper Respiratory Infection	66	2	Not related
100-21	2×10^7	3	Sore throat	1	2	Not related
100-21	2×10^7	3	Headache	1	1	Not related
100-21	2×10^7	3	Dehydration	42	1	Not related
100-22	2×10^8	3	Tachypnea	1	1	Not related
100-22	2×10^8	3	Tachypnea	6	1	Not related
100-23	2×10^7	3	Tachypnea	1	1	Not related
100-23	2×10^7	3	Elevated ALT	6	1	Not related
100-23	2×10^7	3	Increased Total Bilirubin	29	1	Not related
100-24	Placebo	3	Tachypnea	0	2	Not related
100-24	Placebo	3	Diastolic Hypertension	1	1	Not related
100-24	Placebo	3	Decreased Hemoglobin	14	1	Not related
100-25	2×10^8	3	Headache	1	1	Not related
100-26	Placebo	3	Decreased Hemoglobin	22	1	Not related
100-27	Placebo	3	Decreased Hemoglobin	16	2	Not related
100-28	2×10^8	3	Tachypnea	2	1	Not related
100-28	2×10^8	3	Increased White Blood Cells	1	1	Not related
100-28	2×10^8	3	Upper Respiratory Infection	13	2	Not related
100-29	2×10^7	3	Decreased Absolute Neutrophil Count	3	1	Not related
100-30	Placebo	3	Tachypnea	2	1	Not related
100-30	Placebo	3	Elevated White Blood Cell Count	6	1	Not related
100-31	2×10^7	3	Gassy abdominal sensation	3	1	Related

Pub-ID	Dose	Module	Verbatim Term	Days since vaccination	Maximum Severity Grade[1]	Relatedness
100-31	2×10^7	3	Tachypnea	1	1	Not related
100-31	2×10^7	3	Decreased WBC	29	1	Not related
100-31	2×10^7	3	Decreased Neutrophils	29	2	Not related
100-31	2×10^7	3	Decreased Absolute Neutrophil Count	29	2	Not related
100-32	2×10^8	3	Lowered Hemoglobin	0	2	Not related
100-32	2×10^8	3	Lowered Hemoglobin	31	2	Not related
100-33	2×10^8	3	Decreased Absolute Neutrophil Count	1	2	Not related
100-33	2×10^8	3	Decreased White Blood Cells	3	1	Not related
100-33	2×10^8	3	Decreased White Blood Cells	27	1	Not related
100-34	2×10^8	3	Tachypnea	0	1	Not related
100-34	2×10^8	3	Orthostatic tachycardia	2	1	Not related
100-34	2×10^8	3	Increased Total Bilirubin	1	2	Not related
100-34	2×10^8	3	Lowered Absolute Neutrophil Count	11	1	Not related
100-34	2×10^8	3	Kidney Infection	28	2	Not related
100-35	Placebo	3	Tachypnea	2	1	Not related
100-35	Placebo	3	COVID-19 Infection	18	2	Not related
100-35	Placebo	3	Pneumonia	115	2	Not related
100-36	Placebo	3	Tachypnea	2	1	Not related
100-37	2×10^7	3	Systolic hypertension	5	1	Not related
100-37	2×10^7	3	Tachypnea	1	1	Not related
100-38	2×10^7	3	Increased White Blood Cells	1	1	Not related
100-38	2×10^7	3	Increased Sodium	1	2	Not related
100-38	2×10^7	3	COVID infection	36	1	Not related
100-38	2×10^7	3	Hemorrhoids	122	1	Not related

Pub-ID	Dose	Module	Verbatim Term	Days since vaccination	Maximum Severity Grade[1]	Relatedness
100-39	2×10^7	3	Right great toe abrasion	6	1	Not related
100-40	Placebo	3	Tachypnea	0	1	Not related
100-40	Placebo	3	Upper Respiratory Infection	7	1	Not related
100-40	Placebo	3	Increased ALT	11	1	Not related
100-41	2×10^8	3	Diastolic hypertension	2	2	Not related
100-41	2×10^8	3	Increased ALT	3	1	Not related
100-41	2×10^8	3	Increased AST	3	1	Not related
100-42	2×10^8	3	Decreased hemoglobin	13	1	Not related
100-42	2×10^8	3	Hyperkalemia	26	2	Not related
100-43	2×10^8	3	Urticaria	44	2	Not related
100-14	10^4	1	Decreased Absolute Neutrophil Count	1	1	Not related
100-14	10^4	1	Tachypnea	0	1	Not related
100-45	2×10^7	3	External Hemorrhoids	2	2	Not related
100-45	2×10^7	3	Headaches	6	2	Not related
100-45	2×10^7	3	Upper Respiratory Illness	21	2	Not related
100-45	2×10^7	3	Upper respiratory illness	142	1	Not related
100-46	2×10^7	3	Tachycardia	1	1	Not related
100-46	2×10^7	3	Elevated White Blood Cells	1	2	Not related
100-15	10^5	1	Decreased Hemoglobin	16	1	Not related
100-47	2×10^8	3	Chills	2	1	Not related
100-47	2×10^8	3	Gassy GI sensation	4	1	Not related

Pub-ID	Dose	Module	Verbatim Term	Days since vaccination	Maximum Severity Grade[1]	Relatedness
100-16	10^5	1	Lowered Absolute Lymphocytes	3	2	Not related
100-16	10^5	1	Pruritus of the extremities	46	1	Not related
100-17	10^5	1	Viral Upper Respiratory Tract Syndrome	2	2	Not related
100-17	10^5	1	Tachypnea	2	1	Not related
100-17	10^5	1	Elevated AST	14	1	Not related
100-17	10^5	1	Decreased Absolute Neutrophil Count	28	1	Not related
100-18	10^4	1	Tachypnea	1	1	Not related
100-18	10^4	1	Decreased Hemoglobin	0	2	Not related
100-18	10^4	1	Decreased Lymphocytes	1	2	Not related
100-18	10^4	1	Leukopenia	26	1	Not related
100-18	10^4	1	Decreased Absolute Neutrophil Count	26	2	Not related

[1]Graded according to the FDA Toxicity Grading Scale for Healthy Adult and Adolescent Volunteers Enrolled in Preventive Vaccine Clinical Trials (September 2007); [2]dose-escalation module; [3]dose-expansion module

Table S5: Serum vibriocidal antibody responses (VAT) to Inaba and Ogawa *V. cholerae* O1.

Dark blue: no serum sample; D: day.

Dose-Escalation Module

Inaba (VAT)									
**Dose (CFU)**	**D1**	**D4**	**D7**	**D15**	**D29**	**D57**	**D85**	**D180**	**max**
10^5	160	160	320	10240	5120	2560	5120	2560	10240
10^5	5	5	160	5120	2560	2560	2560		5120
10^5	5	5	1280	20480	10240	1280	320		20480
10^6	40	40	2560	10240	5120	2560	1280		10240
10^6	5	5	160	1280	320	80	80	80	1280
10^6	5	5	160	5120	2560	640	640	80	5120
10^7	5	5	160	2560	5120	5120	2560	1280	5120
10^7	5	5	320	5120	2560	2560	320	160	5120
10^7	5	5	160	5120	2560	640	320	40	5120
10^8	5	5	20	1280	640	80	40	40	1280
10^8	20	10	2560	10240	5120	1280	640	320	10240
10^8	40	20	2560	10240	5120	1280	640	640	10240
10^9	5	5	5120	20480	5120	5120	1280	1280	20480
10^9	5	5	10	2560	5120	2560	2560		5120
10^9	5	5	160	5120	2560	640	640	640	5120
10^{10}	5	5	1280	2560	1280		640	640	2560
10^{10}	640	640	10240	81920			5120	2560	81920
10^{10}	40	80	5120	10240	5120	1280	640	640	10240

Fold change to baseline Inaba									
**Dose (CFU)**	**D1**	**D4**	**D7**	**D15**	**D29**	**D57**	**D85**	**D180**	**max FC**
10^5	1	1	2	64	32	16	32	16	64
10^5	1	1	32	1024	512	512	512		1024
10^5	1	1	256	4096	2048	256	64		4096
10^6	1	1	64	256	128	64	32		256
10^6	1	1	32	256	64	16	16	16	256
10^6	1	1	32	1024	512	128	128	16	1024
10^7	1	1	32	512	1024	1024	512	256	1024
10^7	1	1	64	1024	512	512	64	32	1024
10^7	1	1	32	1024	512	128	64	8	1024
10^8	1	1	4	256	128	16	8	8	256
10^8	1	0·5	128	512	256	64	32	16	512
10^8	1	0·5	64	256	128	32	16	16	256
10^9	1	1	1024	4096	1024	1024	256	256	4096
10^9	1	1	2	512	1024	512	512		1024
10^9	1	1	32	1024	512	128	128	128	1024
10^{10}	1	1	256	512	256		128	128	512
10^{10}	1	1	16	128			8	4	128
10^{10}	1	2	128	256	128	32	16	16	256

Dose-Expansion Module

Inaba (VAT)									
**Dose (CFU)**	**D1**	**D4**	**D7**	**D15**	**D29**	**D57**	**D85**	**D180**	**max**
0	5	5	10	5	5	5	5	5	10
0	5	5	10	5	5	5	5	5	10
0	20	20	40	20	20	20	20	20	40
0	5	5	10	5	5	5	5	5	10
0	40	80	80	80	80	80	80	40	80
0	5	5	10	5	5	5	5	5	10
0	5	5	10	5	5	5	5	5	10
2×10^7	320	320	5120	10240	5120	5120	2560	2560	10240
2×10^7	5	5	160	2560	2560	1280	320	80	2560
2×10^7	20	40	5120	20480	5120	2560	1280	640	20480
2×10^7	40	40	640	10240	5120	2560	1280	1280	10240
2×10^7	5	5	2560	10240	5120	2560	1280	320	10240
2×10^7	160	160	1280	5120	5120	5120	5120	1280	5120
2×10^7	20		160	2560	2560	1280	1280		2560
2×10^7	10	10	80	2560	1280	1280	640	320	2560
2×10^7	5	5	1280	2560	1280	320	160	80	2560
2×10^7	10	20	5120	20480	5120	1280	1280	1280	20480
2×10^7	5	5	2560	10240	5120	5120	2560	1280	10240
2×10^7	5	5	1280	10240	10240	10240	10240	2560	10240
2×10^7	20	20	5120	20480	10240	5120	1280	320	20480
2×10^7	20	40	2560	10240	10240	2560		640	10240
2×10^8	10	10	2560	10240	5120	5120	2560	1280	10240
2×10^8	5	5	320	5120	2560	320	160	80	5120
2×10^8	40	40	5120	20480	10240	5120	2560	2560	20480
2×10^8	5	5	320	2560	1280	2560	1280	640	2560
2×10^8	10	10	5120	40960	20480	5120	2560	2560	40960
2×10^8	5	5	5120	10240					10240
2×10^8	5	5	40	1280	320	160	80	80	1280
2×10^8	5	5	20	1280	160	20	10	5	1280
2×10^8	5	5	5120	10240	5120	2560	1280	640	10240
2×10^8	10	10	10240	20480	20480	5120	5120	2560	20480
2×10^8	5	5	160	5120	2560	640	320	320	5120
2×10^8	5	20	640	5120	1280		320	320	5120
2×10^8	10	10	1280	10240	5120	1280	640	320	10240

Fold change to baseline Inaba									
**Dose (CFU)**	**D1**	**D4**	**D7**	**D15**	**D29**	**D57**	**D85**	**D180**	**max FC**
0	1	1	2	1	1	1	1	1	2
0	1	1	2	1	1	1	1	1	2
0	1	1	2	1	1	1	1	1	2
0	1	1	2	1	1	1	1	1	2
0	1	2	2	2	2	2	2	1	2
0	1	1	2	1	1	1	1	1	2
0	1	1	2	1	1	1	1	1	2
2×10^7	1	1	16	32	16	16	8	8	32
2×10^7	1	1	32	512	512	256	64	16	512
2×10^7	1	2	256	1024	256	128	64	32	1024
2×10^7	1	1	16	256	128	64	32	32	256
2×10^7	1	1	512	2048	1024	512	256	64	2048
2×10^7	1	1	8	32	32	32	32	8	32
2×10^7	1		8	128	128	64	64		128
2×10^7	1	1	8	256	128	128	64	32	256
2×10^7	1	1	256	512	256	64	32	16	512
2×10^7	1	2	512	2048	512	128	128	128	2048
2×10^7	1	1	512	2048	1024	1024	512	256	2048
2×10^7	1	1	256	2048	2048	2048	2048	512	2048
2×10^7	1	1	256	1024	512	256	64	16	1024
2×10^7	1	2	128	512	512	128		32	512
2×10^8	1	1	256	1024	512	512	256	128	1024
2×10^8	1	1	64	1024	512	64	32	16	1024
2×10^8	1	1	128	512	256	128	64	64	512
2×10^8	1	1	64	512	256	512	256	128	512
2×10^8	1	1	512	4096	2048	512	256	256	4096
2×10^8	1	1	1024	2048					2048
2×10^8	1	1	8	256	64	32	16	16	256
2×10^8	1	1	4	256	32	4	2	1	256
2×10^8	1	1	1024	2048	1024	512	256	128	2048
2×10^8	1	1	1024	2048	2048	512	512	256	2048
2×10^8	1	1	32	1024	512	128	64	64	1024
2×10^8	1	4	128	1024	256		64	64	1024
2×10^8	1	1	128	1024	512	128	64	32	1024

no serum sample

Dose-Escalation Module

Ogawa (VAT)									
Dose (CFU)	D1	D4	D7	D15	D29	D57	D85	D180	max
10^5	5	5	20	320	160	80	160	160	320
10^5	5	5	10	40	20	20	20		40
10^5	5	5	320	10240	2560	320	80		10240
10^6	80	80	1280	5120	2560	1280	640		5120
10^6	20	20	80	320	160	80	40	80	320
10^6	10	10	320	10240	2560	1280	1280	1280	10240
10^7	20	20	1280	5120	5120	2560	1280	640	5120
10^7	40	40	1280	5120	5120	1280	640	160	5120
10^7	5	5	40	2560	640	160	160	20	2560
10^8	80	80	160	5120	1280	640	320	320	5120
10^8	5	5	160	5120	1280	640	640	320	5120
10^8	640	640	640	2560	1280	640	640	1280	2560
10^9	5	5	2560	10240	2560	1280	320	320	10240
10^9	5	5	10	320	1280	1280	1280		1280
10^9	80	160	160	1280	1280	640	640	1280	1280
10^{10}	80	80	320	640	640		160	160	640
10^{10}	320	320	2560	40960			2560	1280	40960
10^{10}	40	80	10240	10240	2560	2560	640	640	10240

Fold change to baseline Ogawa									
Dose (CFU)	D1	D4	D7	D15	D29	D57	D85	D180	max FC
10^5	1	1	4	64	32	16	32	32	64
10^5	1	1	2	8	4	4	4		8
10^5	1	1	64	2048	512	64	16		2048
10^6	1	1	16	64	32	16	8		64
10^6	1	1	4	16	8	4	2	4	16
10^6	1	1	32	1024	256	128	128	128	1024
10^7	1	1	64	256	256	128	64	32	256
10^7	1	1	32	128	128	32	16	4	128
10^7	1	1	8	512	128	32	32	4	512
10^8	1	1	2	64	16	8	4	4	64
10^8	1	1	32	1024	256	128	128	64	1024
10^8	1	1	1	4	2	1	1	2	4
10^9	1	1	512	2048	512	256	64	64	2048
10^9	1	1	2	64	256	256	256		256
10^9	1	2	2	16	16	8	8	16	16
10^{10}	1	1	4	8	8		2	2	8
10^{10}	1	1	8	128			8	4	128
10^{10}	1	2	256	256	64	64	16	16	256

Dose-Expansion Module

Ogawa (VAT)									
Dose (CFU)	D1	D4	D7	D15	D29	D57	D85	D180	max
0	5	5	10	5	5	5	5	5	10
0	5	5	10	5	5	5	5	5	10
0	80	40	80	40	40	40	40	80	80
0	40	80	80	40	80	80	80	80	80
0	40	40	40	40	40	40	40	20	40
0	40	20	40	20	20	40	80	80	80
0	5	5	10	5	5	5	5	5	10
2×10^7	20	20	5120	10240	2560	2560	2560	1280	10240
2×10^7	80	80	640	10240	5120	5120	2560	640	10240
2×10^7	10	10	1280	5120	2560	1280	640	320	5120
2×10^7	10	20	80	1280	160	40	20	20	1280
2×10^7	5	5	1280	5120	2560	640	320	80	5120
2×10^7	5	5	640	2560	640	80	80	10	2560
2×10^7	5		40	640	80	80	20		640
2×10^7	20	40	80	1280	2560	1280	640	160	2560
2×10^7	5	5	320	1280	320	40	20	10	1280
2×10^7	5	10	1280	10240	5120	1280	1280	320	10240
2×10^7	5	5	640	5120	1280	1280	640	320	5120
2×10^7	5	5	80	640	320	320	320	320	640
2×10^7	5	5	160	640	160	40	10	5	640
2×10^7	5	5	640	10240	5120	2560		640	10240
2×10^8	5	5	1280	5120	2560	2560	640	320	5120
2×10^8	5	5	640	2560	2560	640	320	160	2560
2×10^8	5	5	320	2560	1280	320	160	40	2560
2×10^8	160	160	640	1280	1280	640	640	1280	1280
2×10^8	5	5	160	640	320	80	40	40	640
2×10^8	20	20	1280	10240					10240
2×10^8	5	5	40	640	160	80	80	80	640
2×10^8	5	5	20	640	80	10	5	5	640
2×10^8	5	5	2560	10240	2560	1280	1280	640	10240
2×10^8	320	160	5120	20480	20480	10240	5120	2560	20480
2×10^8	5	5	320	5120	5120	640	320	320	5120
2×10^8	5	20	160	1280	640		160	80	1280
2×10^8	5	5	20	160	80	40	20	20	160

Fold change to baseline Ogawa									
Dose (CFU)	D1	D4	D7	D15	D29	D57	D85	D180	max FC
0	1	1	2	1	1	1	1	1	2
0	1	1	2	1	1	1	1	1	2
0	1	0·5	1	0·5	0·5	0·5	0·5	1	1
0	1	2	2	1	2	2	2	2	2
0	1	1	1	1	1	1	1	0·5	1
0	1	0·5	1	0·5	0·5	1	2	2	2
0	1	1	2	1	1	1	1	1	2
2×10^7	1	1	256	512	128	128	128	64	512
2×10^7	1	1	8	128	64	64	32	8	128
2×10^7	1	1	128	512	256	128	64	32	512
2×10^7	1	2	8	128	16	4	2	2	128
2×10^7	1	1	256	1024	512	128	64	16	1024
2×10^7	1	1	128	512	128	16	16	2	512
2×10^7	1		8	128	16	16	4		128
2×10^7	1	2	4	64	128	64	32	8	128
2×10^7	1	1	64	256	64	8	4	2	256
2×10^7	1	2	256	2048	1024	256	256	64	2048
2×10^7	1	1	128	1024	256	256	128	64	1024
2×10^7	1	1	16	128	64	64	64	64	128
2×10^7	1	1	32	128	32	8	2	1	128
2×10^7	1	1	128	2048	1024	512		128	2048
2×10^8	1	1	256	1024	512	512	128	64	1024
2×10^8	1	1	128	512	512	128	64	32	512
2×10^8	1	1	64	512	256	64	32	8	512
2×10^8	1	1	4	8	8	4	4	8	8
2×10^8	1	1	32	128	64	16	8	8	128
2×10^8	1	1	64	512					512
2×10^8	1	1	8	128	32	16	16	16	128
2×10^8	1	1	4	128	16	2	1	1	128
2×10^8	1	1	512	2048	512	256	256	128	2048
2×10^8	1	0·5	16	64	64	32	16	8	64
2×10^8	1	1	64	1024	1024	128	64	64	1024
2×10^8	1	4	32	256	128		32	16	256
2×10^8	1	1	4	32	16	8	4	4	32

Table S6: Kinetics of mean vibriocidal responses to both serotypes in serum of vaccine (10^7 and 10^8 CFU of PanChol) and placebo recipients. Geometric mean titers with 95% confidence intervals are presented at baseline and day 7, 15, 29 and 180 post-vaccination. * p<0·0001 representing the comparison of day 15 vibriocidal titers of placebo and vaccine recipients dosed either with 10^7 or 10^8 CFU (Mann-Whitney test). GMT, geometric mean titer; CI, confidence interval.

Serotype	Dose group (CFU)	n	GMT (95% CI) post vaccination (days)				
			Baseline	7	15	29	180
Inaba	0 (Placebo)	7	8·2 (3·7-18·3)	16·4 (7·3-36·7)	9·1 (3·3-24·8)	9·1 (3·3-24·8)	8·2 (3·7-18·3)
	10^7	17	13·3 (6·9-25·6)	923·7 (433·2-1970)	6811 (4567-10157)*	4176 (3009-5795)	493·5 (248·5-980)
	10^8	16	8·4 (5·7-12·5)	794·8 (262-2411)	6640 (3815-11555)*	2808 (1288-6122)	335·1 (128·8-872·2)
Ogawa	0 (Placebo)	7	18·1 (5·8-56·4)	26·9 (11-65·9)	14·9 (5·6-39·2)	16·4 (5·5-48·9)	20 (5·5-72·1)
	10^7	17	9·2 (5·9-14·5)	392·4 (186·7-824·8)	3013 (1778-5107)*	1180 (566·6-2457)	118·1 (46-303·6)
	10^8	16	14·1 (5·6-35·7)	306·4 (129·9-722·7)	2451 (1241-4842)*	1016 (437·8-2358)	175·5 (67·9-453·7)

Table S7: Mean peak vibriocidal responses and mean peak fold increase in vibriocidal responses by dose group. Mean peak fold increase in titer was calculated using each participant's baseline and peak titer.

Serotype	Dose group (CFU)	n	Mean peak titer	Mean peak fold increase	Serotype	Dose group (CFU)	n	Mean peak titer	Mean peak fold increase
**Inaba**	0 (Placebo)	7	24·3	2	**Ogawa**	0 (Placebo)	7	44·3	1·7
	10^5	3	11947	1728		10^5	3	3533	706·7
	10^6	3	5547	512		10^6	3	5227	368
	10^7	17	9035	914·8		10^7	17	4631	564·7
	10^8	16	10320	1120		10^8	16	4610	466·8
	10^9	3	10240	2048		10^9	3	4267	773·3
	10^{10}	3	31573	298·7		10^{10}	3	17280	130·7

Table S8: Antigen- and isotype-specific immune responses in serum derived from vaccine and placebo recipients. Dark blue: no serum sample; yellow: below limit of detection; D: day.

Dose-Escalation Module

Inaba OSP IgM (RAU)									
Dose (CFU)	D1	D4	D7	D15	D29	D57	D85	D180	max
10^5	0·00175	0·00144	0·00306	0·01644	0·00958	0·01024	0·01103	0·01715	0·01715
10^5	0·00126	0·00095	0·00162	0·01764	0·01516	0·00761	0·01024		0·01764
10^5	0·01793	0·01833	0·03245	0·31615	0·14471	0·03311	0·01909		0·31615
10^6	0·00363	0·00276	0·01923	0·93973	0·52374	0·13277	0·07381		0·93973
10^6	0·00561	0·00682	0·00965	0·01408	0·00768	0·00737	0·00640	0·00437	0·01408
10^6	0·00093	0·00068	0·00456	0·16252	0·06483	0·03039	0·02319	0·00299	0·16252
10^7	0·00414	0·00369	0·00648	0·02198	0·01506	0·01381	0·01072	0·00439	0·02198
10^7	0·00124	0·00179	0·00505	0·21703	0·16044	0·04923	0·01157	0·00277	0·21703
10^7	0·00768	0·00790	0·00545	0·09049	0·03188	0·01712	0·01207	0·00696	0·09049
10^8	0·00089	0·00076	0·00075	0·02221	0·01372	0·00391	0·00228	0·00177	0·02221
10^8	0·01379	0·01208	0·01937	0·18950	0·05626	0·03126	0·00977	0·00854	0·18950
10^8	0·00656	0·00631	0·06425	0·39774	0·18408	0·01766	0·00958	0·01381	0·39774
10^9	0·00441	0·00434	0·24562	4·31117	1·03921	0·14913	0·04487	0·02506	4·31117
10^9	0·00347	0·00346	0·00243	0·03168	0·06364	0·07431	0·14028		0·14028
10^9	0·00590	0·00575	0·00717	0·05801	0·01343	0·01043	0·00990	0·00846	0·05801
10^{10}	0·00375	0·00431	0·00617	0·01547	0·01696		0·00773	0·00613	0·01696
10^{10}	0·01880	0·02335	0·30780	6·79807			0·46855	0·10156	6·79807
10^{10}	0·00426	0·00453	0·33315	0·94811	0·26635	0·11948	0·03265	0·01855	0·94811

Fold change Inaba OSP IgM									
Dose (CFU)	D1	D4	D7	D15	D29	D57	D85	D180	max FC
10^5	1·00	0·82	1·75	9·40	5·48	5·85	6·31	9·81	9·81
10^5	1·00	0·75	1·29	14·03	12·06	6·06	8·14		14·03
10^5	1·00	1·02	1·81	17·63	8·07	1·85	1·06		17·63
10^6	1·00	0·76	5·29	258·60	144·13	36·54	20·31		258·60
10^6	1·00	1·22	1·72	2·51	1·37	1·31	1·14	0·78	2·51
10^6	1·00	0·73	4·88	174·07	69·43	32·55	24·83	3·20	174·07
10^7	1·00	0·89	1·57	5·31	3·64	3·34	2·59	1·06	5·31
10^7	1·00	1·44	4·07	174·76	129·20	39·64	9·32	2·23	174·76
10^7	1·00	1·03	0·71	11·78	4·15	2·23	1·57	0·91	11·78
10^8	1·00	0·85	0·84	24·93	15·40	4·39	2·56	1·98	24·93
10^8	1·00	0·88	1·40	13·74	4·08	2·27	0·71	0·62	13·74
10^8	1·00	0·96	9·80	60·65	28·07	2·69	1·46	2·11	60·65
10^9	1·00	0·98	55·69	977·49	235·62	33·81	10·17	5·68	977·49
10^9	1·00	1·00	0·70	9·13	18·35	21·42	40·44		40·44
10^9	1·00	0·98	1·22	9·83	2·28	1·77	1·68	1·43	9·83
10^{10}	1·00	1·15	1·65	4·13	4·53		2·06	1·64	4·53
10^{10}	1·00	1·24	16·37	361·61			24·92	5·40	361·61
10^{10}	1·00	1·06	78·23	222·63	62·54	28·06	7·67	4·36	222·63

Dose-Expansion Module

Inaba OSP IgM (RAU)									
Dose (CFU)	D1	D4	D7	D15	D29	D57	D85	D180	max
0	0·01556	0·01464	0·01584	0·01284	0·01609	0·01492	0·01502	0·01872	0·01872
0	0·00211	0·00208	0·00191	0·00195	0·00177	0·00213	0·00131	0·00140	0·00213
0	0·00627	0·00622	0·00801	0·00736	0·00683	0·00492	0·00421	0·00236	0·00801
0	0·01555	0·01616	0·01718	0·01349	0·01278	0·01087	0·00582	0·00623	0·01718
0	0·00812	0·00851	0·01115	0·01079	0·01197	0·00603	0·00527	0·00341	0·01197
0	0·01112	0·01201	0·01267	0·00696	0·00476	0·00736	0·00760	0·01049	0·01267
0	0·00373	0·00315	0·00445	0·00364	0·00566	0·00495	0·00297	0·00525	0·00566
2×10^7	0·02693	0·02433	0·05189	1·64138	0·54016	0·10056	0·06938	0·05244	1·64138
2×10^7	0·00400	0·00392	0·00891	1·10863	0·82969	0·28677	0·12419	0·02575	1·10863
2×10^7	0·00803	0·00948	0·26074	1·80558	0·82150	0·19866	0·07150	0·03734	1·80558
2×10^7	0·01078	0·01480	0·01288	0·06363	0·02056	0·01478	0·01271	0·00346	0·06363
2×10^7	0·01156	0·00962	0·02580	0·78914	0·33725	0·08086	0·05265	0·01212	0·78914
2×10^7	0·00365	0·00378	0·00385	0·03952	0·01945	0·00633	0·00645	0·00205	0·03952
2×10^7	0·01209		0·01357	0·08424	0·05591	0·03802	0·02052		0·08424
2×10^7	0·00975	0·01157	0·02102	1·71489	1·01604	0·45261	0·24544	0·05908	1·71489
2×10^7	0·00456	0·00541	0·03353	0·54943	0·28543	0·02710	0·01302	0·00696	0·54943
2×10^7	0·01890	0·02335	0·04092	1·06095	0·28424	0·08038	0·05760	0·03796	1·06095
2×10^7	0·00423	0·00670	0·01116	0·09685	0·09707	0·05488	0·04045	0·02794	0·09707
2×10^7	0·00272	0·00239	0·00892	0·20537	0·21348	0·15186	0·11463	0·06743	0·21348
2×10^7	0·00332	0·00415	0·18596	3·16258	1·40009	0·28981	0·06714	0·00838	3·16258
2×10^7	0·00398	0·00284	0·03938	0·59937	0·44841	0·16582		0·04012	0·59937
2×10^8	0·01083	0·01016	0·28587	3·14981	1·57439	0·32397	0·15242	0·07598	3·14981
2×10^8	0·00125	0·00141	0·00262	0·11775	0·03358	0·00738	0·00570	0·00283	0·11775
2×10^8	0·01293	0·01374	0·14948	3·30181	0·96768	0·39240	0·10271	0·03311	3·30181
2×10^8	0·02113	0·02248	0·02040	0·06589	0·08768	0·05750	0·03167	0·01348	0·08768
2×10^8	0·00649	0·00764	0·02231	0·78785	0·38783	0·02816	0·02252	0·01384	0·78785
2×10^8	0·01192	0·01281	0·01424	0·20385					0·20385
2×10^8	0·02210	0·02278	0·02289	0·11330	0·02619	0·00940	0·00640	0·00484	0·11330
2×10^8	0·00196	0·00151	0·00151	0·03434	0·01100	0·00211	0·00145	0·00043	0·03434
2×10^8	0·00449	0·00388	0·13228	3·00340	1·02997	0·21627	0·14575	0·07240	3·00340
2×10^8	0·00143	0·00166	0·20431	3·97839	2·31500	0·77571	0·37617	0·17124	3·97839
2×10^8	0·00100	0·00112	0·00353	0·30476	0·18180	0·05164	0·02710	0·01474	0·30476
2×10^8	0·00136	0·00173	0·01995	0·24154	0·08180		0·01286	0·01154	0·24154
2×10^8	0·00042	0·00049	0·02048	0·23459	0·12405	0·03463	0·02207	0·00872	0·23459

Fold change Inaba OSP IgM									
Dose (CFU)	D1	D4	D7	D15	D29	D57	D85	D180	max FC
0	1·00	0·94	1·02	0·82	1·03	0·96	0·97	1·20	1·20
0	1·00	0·99	0·91	0·93	0·84	1·01	0·62	0·66	1·01
0	1·00	0·99	1·28	1·17	1·09	0·78	0·67	0·38	1·28
0	1·00	1·04	1·10	0·87	0·82	0·70	0·37	0·40	1·10
0	1·00	1·05	1·37	1·33	1·47	0·74	0·65	0·42	1·47
0	1·00	1·08	1·14	0·63	0·43	0·66	0·68	0·94	1·14
0	1·00	0·85	1·20	0·98	1·52	1·33	0·80	1·41	1·52
2×10^7	1·00	0·90	1·93	60·96	20·06	3·73	2·58	1·95	60·96
2×10^7	1·00	0·98	2·22	276·88	207·21	71·62	31·02	6·43	276·88
2×10^7	1·00	1·18	32·48	224·95	102·35	24·75	8·91	4·65	224·95
2×10^7	1·00	1·37	1·19	5·90	1·91	1·37	1·18	0·32	5·90
2×10^7	1·00	0·83	2·23	68·25	29·17	6·99	4·55	1·05	68·25
2×10^7	1·00	1·03	1·05	10·81	5·32	1·73	1·77	0·56	10·81
2×10^7	1·00		1·12	6·97	4·63	3·15	1·70		6·97
2×10^7	1·00	1·19	2·16	175·87	104·20	46·42	25·17	6·06	175·87
2×10^7	1·00	1·19	7·36	120·58	62·64	5·95	2·86	1·53	120·58
2×10^7	1·00	1·24	2·16	56·13	15·04	4·25	3·05	2·01	56·13
2×10^7	1·00	1·58	2·64	22·87	22·92	12·96	9·55	6·60	22·92
2×10^7	1·00	0·88	3·28	75·43	78·41	55·78	42·11	24·77	78·41
2×10^7	1·00	1·25	56·05	953·26	422·01	87·35	20·24	2·52	953·26
2×10^7	1·00	0·71	9·90	150·66	112·71	41·68		10·08	150·66
2×10^8	1·00	0·94	26·38	290·71	145·31	29·90	14·07	7·01	290·71
2×10^8	1·00	1·14	2·10	94·56	26·97	5·93	4·57	2·27	94·56
2×10^8	1·00	1·06	11·56	255·29	74·82	30·34	7·94	2·56	255·29
2×10^8	1·00	1·06	0·97	3·12	4·15	2·72	1·50	0·64	4·15
2×10^8	1·00	1·18	3·44	121·41	59·77	4·34	3·47	2·13	121·41
2×10^8	1·00	1·07	1·20	17·10					17·10
2×10^8	1·00	1·03	1·04	5·13	1·18	0·43	0·29	0·22	5·13
2×10^8	1·00	0·77	0·77	17·52	5·61	1·08	0·74	0·22	17·52
2×10^8	1·00	0·86	29·47	669·00	229·42	48·17	32·47	16·13	669·00
2×10^8	1·00	1·16	143·35	2791·22	1624·19	544·24	263·92	120·14	2791·22
2×10^8	1·00	1·13	3·54	305·70	182·36	51·80	27·19	14·78	305·70
2×10^8	1·00	1·27	14·66	177·48	60·10		9·45	8·48	177·48
2×10^8	1·00	1·15	48·37	554·02	292·97	81·78	52·11	20·59	554·02

no serum sample

	RAU		
	D1	D39	
V. cholerae	0·00146	0·01628	
	0·00700	0·67315	
	0·00300	0·02255	
	0·02800	1·54587	
	0·00411	0·92681	
	D1	D11	
Vaxchora D11 Challenge	0·00092	0·17460	
	0·01711	0·02180	
	0·00105	0·10184	
	0·00231	0·01162	
	0·00513	0·02466	
	0·00275	0·37451	
	D1	D11	D91
Vaxchora D91 Challenge	0·00083	0·00602	0·00159
	0·00573	0·08117	0·00542
	0·00011	0·01309	0·00124
	0·02200	0·02229	0·03015
	0·01382	0·17817	0·01984
	0·00047	0·05876	0·00221

	RAU fold change			max FC
	D1	D39		
V. cholerae	1·00	11·17		11·17
	1·00	96·15		96·15
	1·00	7·52		7·52
	1·00	55·20		55·20
	1·00	225·62		225·62
	D1	D11		
Vaxchora D11 Challenge	1·00	188·92		188·92
	1·00	1·27		1·27
	1·00	96·76		96·76
	1·00	5·04		5·04
	1·00	4·80		4·80
	1·00	136·23		136·23
	D1	D11	D91	
Vaxchora D91 Challenge	1·00	7·26	1·92	7·26
	1·00	14·17	0·95	14·17
	1·00	117·29	11·13	117·29
	1·00	1·01	1·37	1·37
	1·00	12·89	1·44	12·89
	1·00	125·07	4·70	125·07

Dose-Escalation Module

Inaba OSP IgG (RAU)									
**Dose (CFU)**	**D1**	**D4**	**D7**	**D15**	**D29**	**D57**	**D85**	**D180**	**max**
10^5	0·00194	0·00115	0·00177	0·00163	0·00139	0·00100	0·00174	0·00441	0·00441
10^5	0·00140	0·00130	0·00205	0·00660	0·00423	0·00433	0·00456		0·00660
10^5	0·00250	0·00239	0·00268	2·22367	0·94033	0·17544	0·13084		2·22367
10^6	0·00307	0·00318	0·00297	0·10718	0·06311	0·06985	0·07886		0·10718
10^6	0·00241	0·00286	0·00338	0·00497	0·00590	0·01282	0·01726	0·02262	0·02262
10^6	0·00430	0·00421	0·01310	0·52376	0·29138	0·14099	0·07167	0·02842	0·52376
10^7	0·01248	0·01254	0·01109	0·01214	0·01443	0·03133	0·03248	0·08199	0·08199
10^7	0·00544	0·00684	0·00786	0·01541	0·00871	0·00655	0·00909	0·00746	0·01541
10^7	0·00455	0·00438	0·00472	0·01068	0·00739	0·00560	0·00619	0·00675	0·01068
10^8	0·01150	0·01121	0·01150	0·01074	0·01214	0·01020	0·01503	0·07338	0·07338
10^8	0·00977	0·00909	0·00793	0·08510	0·04097	0·03496	0·01479	0·01644	0·08510
10^8	0·01265	0·00877	0·01044	0·01197	0·01271	0·00704	0·01687	0·04659	0·04659
10^9	0·00480	0·00641	0·00705	0·00725	0·00746	0·00610	0·00870	0·01516	0·01516
10^9	0·00307	0·00058	0·00203	0·00352	0·01442	0·02464	0·13151		0·13151
10^9	0·00528	0·00450	0·00434	0·00573	0·00535	0·00505	0·00580	0·00808	0·00808
10^{10}	0·00318	0·00361	0·00466	0·01116	0·00372		0·00490	0·00774	0·01116
10^{10}	0·00168	0·00157	0·00208	0·00580			0·00265	0·00168	0·00580
10^{10}	0·00815	0·00801	0·01056	0·01120	0·01153	0·01305	0·01632	0·02353	0·02353

Fold change Inaba OSP IgG									
**Dose (CFU)**	**D1**	**D4**	**D7**	**D15**	**D29**	**D57**	**D85**	**D180**	**max FC**
10^5	1·00	0·59	0·91	0·84	0·72	0·52	0·90	2·28	2·28
10^5	1·00	0·92	1·46	4·71	3·01	3·08	3·25		4·71
10^5	1·00	0·95	1·07	890·25	376·46	70·24	52·38		890·25
10^6	1·00	1·03	0·97	34·86	20·53	22·72	25·65		34·86
10^6	1·00	1·19	1·40	2·06	2·45	5·32	7·16	9·39	9·39
10^6	1·00	0·98	3·05	121·87	67·80	32·80	16·68	6·61	121·87
10^7	1·00	1·00	0·89	0·97	1·16	2·51	2·60	6·57	6·57
10^7	1·00	1·26	1·44	2·83	1·60	1·20	1·67	1·37	2·83
10^7	1·00	0·96	1·04	2·34	1·62	1·23	1·36	1·48	2·34
10^8	1·00	0·97	1·00	0·93	1·06	0·89	1·31	6·38	6·38
10^8	1·00	0·93	0·81	8·71	4·19	3·58	1·51	1·68	8·71
10^8	1·00	0·69	0·83	0·95	1·00	0·56	1·33	3·68	3·68
10^9	1·00	1·33	1·47	1·51	1·55	1·27	1·81	3·16	3·16
10^9	1·00	0·19	0·66	1·15	4·69	8·01	42·77		42·77
10^9	1·00	0·85	0·82	1·09	1·01	0·96	1·10	1·53	1·53
10^{10}	1·00	1·13	1·47	3·51	1·17		1·54	2·43	3·51
10^{10}	1·00	0·94	1·24	3·46			1·58	1·00	3·46
10^{10}	1·00	0·98	1·29	1·37	1·41	1·60	2·00	2·89	2·89

Dose-Expansion Module

Inaba OSP IgG (RAU)									
**Dose (CFU)**	**D1**	**D4**	**D7**	**D15**	**D29**	**D57**	**D85**	**D180**	**max**
0	0·00267	0·00398	0·00320	0·00341	0·00386	0·00347	0·00426	0·00524	0·00524
0	0·01160	0·01445	0·01264	0·01043	0·01103	0·01073	0·00973	0·00931	0·01445
0	0·00664	0·00784	0·00824	0·00678	0·00791	0·00789	0·00689	0·00092	0·00824
0	0·02507	0·02676	0·02513	0·02255	0·02755	0·00386	0·00278	0·00312	0·02755
0	0·01818	0·01676	0·01799	0·01884	0·01738	0·00482	0·00439	0·00378	0·01884
0	0·00483	0·00444	0·00477	0·00060	0·00025	0·00094	0·00055	0·00047	0·00483
0	0·00040	0·00047	0·00055	0·00068	0·00046	0·00021	0·00016	0·00051	0·00068
2×10^7	0·01302	0·00912	0·00878	0·01040	0·01040	0·01079	0·01079	0·01450	0·01450
2×10^7	0·00764	0·00837	0·00866	2·31134	1·23467	0·43538	0·22251	0·07230	2·31134
2×10^7	0·00458	0·00412	0·00329	0·00632	0·00504	0·00390	0·00299	0·00378	0·00632
2×10^7	0·00418	0·00450	0·00398	0·00416	0·00329	0·00412	0·00171	0·00112	0·00450
2×10^7	0·00787	0·00764	0·00832	0·09016	0·04984	0·03909	0·04295	0·08024	0·09016
2×10^7	0·00975	0·00796	0·00797	0·00682	0·00923	0·00857	0·01040	0·00207	0·01040
2×10^7	0·00792		0·00683	0·01018	0·01085	0·00951	0·00936		0·01085
2×10^7	0·00243	0·00199	0·00250	0·00364	0·00374	0·00095		0·00055	0·00374
2×10^7	0·01160	0·00981	0·00961	0·01058	0·00981	0·02887	0·03418	0·08353	0·08353
2×10^7	0·01253	0·01253	0·01472	0·01523	0·00485	0·00716	0·01027	0·05494	0·05494
2×10^7	0·00122	0·00089	0·00154	0·00230	0·00269	0·00356	0·00625	0·02420	0·02420
2×10^7	0·00525	0·00504	0·00557	0·00580	0·00670	0·00694	0·00906	0·03351	0·03351
2×10^7	0·00192	0·00231	0·00167	0·00257	0·00299	0·00279	0·00230	0·00249	0·00299
2×10^7	0·00055	0·00047	0·00095	0·02504	0·02310	0·01352		0·01527	0·02504
2×10^8	0·00620	0·00594	0·00720	0·00946	0·01004	0·02894	0·08113	0·09170	0·09170
2×10^8	0·00411	0·00379	0·00373	0·00293	0·00337	0·00360	0·00337	0·00362	0·00411
2×10^8	0·00404	0·00400	0·00490	0·00629	0·00616	0·01773	0·06905	0·15288	0·15288
2×10^8	0·01246	0·01342	0·01285	0·01217	0·01325	0·02392	0·02033	0·06327	0·06327
2×10^8	0·00362	0·00217	0·00232	0·00500	0·00446	0·00021	0·00152	0·00391	0·00500
2×10^8	0·00221	0·00258	0·00190	0·00245					0·00258
2×10^8	0·00772	0·00852	0·00840	0·00931	0·00201	0·01163	0·02465	0·03705	0·03705
2×10^8	0·00151	0·00247	0·00254	0·00125	0·00007			0·00004	0·00254
2×10^8	0·00675	0·00481	0·00747	0·00678	0·00381	0·00319	0·00474	0·02701	0·02701
2×10^8	0·00382	0·00457	0·00319	0·00405	0·00518	0·00441	0·00380	0·00448	0·00518
2×10^8	0·00087	0·00076	0·00066	0·00142	0·00083	0·02790	0·06279	0·11848	0·11848
2×10^8	0·00207	0·00218	0·00257	0·35286	0·23629		0·20667	0·27097	0·35286
2×10^8	0·00021	0·00011	0·00064	0·00099	0·00062	0·01304	0·01182	0·02579	0·02579

Fold change Inaba OSP IgG									
**Dose (CFU)**	**D1**	**D4**	**D7**	**D15**	**D29**	**D57**	**D85**	**D180**	**max FC**
0	1·00	1·49	1·20	1·28	1·45	1·30	1·60	1·96	1·96
0	1·00	1·25	1·09	0·90	0·95	0·93	0·84	0·80	1·25
0	1·00	1·18	1·24	1·02	1·19	1·19	1·04	0·14	1·24
0	1·00	1·07	1·00	0·90	1·10	0·15	0·11	0·12	1·10
0	1·00	0·92	0·99	1·04	0·96	0·26	0·24	0·21	1·04
0	1·00	0·92	0·99	0·13	0·05	0·19	0·11	0·10	0·99
0	1·00	1·19	1·37	1·69	1·14	0·53	0·40	1·28	1·69
2×10^7	1·00	0·70	0·67	0·80	0·80	0·83	0·83	1·11	1·11
2×10^7	1·00	1·10	1·13	302·55	161·62	56·99	29·13	9·46	302·55
2×10^7	1·00	0·90	0·72	1·38	1·10	0·85	0·65	0·83	1·38
2×10^7	1·00	1·08	0·95	1·00	0·79	0·99	0·41	0·27	1·08
2×10^7	1·00	0·97	1·06	11·45	6·33	4·96	5·45	10·19	11·45
2×10^7	1·00	0·82	0·82	0·70	0·95	0·88	1·07	0·21	1·07
2×10^7	1·00		0·86	1·28	1·37	1·20	1·18		1·37
2×10^7	1·00	0·82	1·03	1·50	1·54	0·39		0·23	1·54
2×10^7	1·00	0·85	0·83	0·91	0·85	2·49	2·95	7·20	7·20
2×10^7	1·00	1·00	1·17	1·21	0·39	0·57	0·82	4·38	4·38
2×10^7	1·00	0·73	1·27	1·89	2·21	2·93	5·14	19·89	19·89
2×10^7	1·00	0·96	1·06	1·11	1·28	1·32	1·73	6·39	6·39
2×10^7	1·00	1·20	0·87	1·34	1·55	1·45	1·19	1·29	1·55
2×10^7	1·00	0·87	1·74	45·65	42·11	24·65		27·83	45·65
2×10^8	1·00	0·96	1·16	1·53	1·62	4·67	13·09	14·80	14·80
2×10^8	1·00	0·92	0·91	0·71	0·82	0·88	0·82	0·88	0·92
2×10^8	1·00	0·99	1·21	1·55	1·52	4·38	17·08	37·81	37·81
2×10^8	1·00	1·08	1·03	0·98	1·06	1·92	1·63	5·08	5·08
2×10^8	1·00	0·60	0·64	1·38	1·23	0·06	0·42	1·08	1·38
2×10^8	1·00	1·17	0·86	1·11					1·17
2×10^8	1·00	1·10	1·09	1·20	0·26	1·51	3·19	4·80	4·80
2×10^8	1·00	1·64	1·69	0·83	0·05			0·03	1·69
2×10^8	1·00	0·71	1·11	1·01	0·56	0·47	0·70	4·00	4·00
2×10^8	1·00	1·20	0·84	1·06	1·36	1·16	0·99	1·17	1·36
2×10^8	1·00	0·88	0·76	1·63	0·96	32·11	72·27	136·36	136·36
2×10^8	1·00	1·05	1·24	170·19	113·97		99·68	130·69	170·19
2×10^8	1·00	0·50	3·01	4·64	2·92	61·29	55·58	121·20	121·20

below limit of detection

	RAU		
	D1	D39	
V. cholerae	0·00455	0·02975	
	0·00376	0·07930	
	0·01779	0·03023	
	0·01150	0·21244	
	0·00455	0·18440	
	D1	D11	
Vaxchora D11 Challenge	0·01321	0·03282	
	0·01684	0·01647	
	0·05171	0·07018	
	0·00921	0·01038	
	0·00909	0·03974	
	0·00759	0·04323	
	D1	D11	D91
Vaxchora D91 Challenge	0·00348	0·00634	0·00480
	0·01944	0·03842	0·01990
	0·00896	0·01679	0·01421
	0·02020	0·02733	0·02222
	0·00627	0·01877	0·01080
	0·00560	0·01443	0·00940

	RAU fold change			max FC
	D1	D39		
V. cholerae	1·00	6·53		6·53
	1·00	21·09		21·09
	1·00	1·70		1·70
	1·00	18·47		18·47
	1·00	40·49		40·49
	D1	D11		
Vaxchora D11 Challenge	1·00	2·48		2·48
	1·00	0·98		0·98
	1·00	1·36		1·36
	1·00	1·13		1·13
	1·00	4·37		4·37
	1·00	5·69		5·69
	D1	D11	D91	
Vaxchora D91 Challenge	1·00	1·82	1·38	1·82
	1·00	1·98	1·02	1·98
	1·00	1·87	1·59	1·87
	1·00	1·35	1·10	1·35
	1·00	3·00	1·72	3·00
	1·00	2·58	1·68	2·58

Dose-Escalation Module

Inaba OSP IgA (RAU)									
Dose (CFU)	D1	D4	D7	D15	D29	D57	D85	D180	max
10^5	0·00017	0·00042	0·00087	0·00129	0·00130	0·00143	0·00128	0·00106	0·00143
10^5	0·00175	0·00139	0·00190	0·00196	0·00191	0·00186	0·00193		0·00196
10^5	0·00140	0·00167	0·00194	0·04121	0·01029	0·00513	0·00761		0·04121
10^6	0·00013	0·00025	0·00111	0·25844	0·01975	0·00918	0·00783		0·25844
10^6	0·00191	0·00208	0·01421	0·11625	0·02677	0·01284	0·01007	0·00819	0·11625
10^6	0·00017	0·00035	0·00047	0·20900	0·03196	0·00641	0·00184	0·00145	0·20900
10^7	0·00044	0·00036	0·00035	0·00058	0·00119	0·00157	0·00223	0·00231	0·00231
10^7	0·00035	0·00034	0·00113	0·00153	0·00120	0·00078	0·00036	0·00005	0·00153
10^7	0·00096	0·00090	0·00067	0·01184	0·00181	0·00071	0·00082	0·00009	0·01184
10^8	0·00032	0·00032	0·00036	0·00111	0·00047	0·00077	0·00097	0·00206	0·00206
10^8	0·00090	0·00093	0·00058	0·00321	0·00128	0·00110	0·00352	0·00462	0·00462
10^8	0·00043	0·00028	0·01524	0·10628	0·01708	0·00417	0·00482	0·00480	0·10628
10^9	0·00073	0·00055	0·00219	0·03534	0·01117	0·00405	0·00177	0·00136	0·03534
10^9	0·00241	0·00228	0·00268	0·00271	0·01569	0·01230	0·02330		0·02330
10^9	0·00059	0·00052	0·00067	0·00107	0·00125	0·00115	0·00125	0·00193	0·00193
10^{10}	0·00006	0·00003	0·00022	0·00066	0·00030		0·00027	0·00028	0·00066
10^{10}	0·00075	0·00083	0·00319	0·02563			0·01147	0·00367	0·02563
10^{10}	0·00008	0·00005	0·00403	0·11487	0·01491	0·00439	0·00373	0·00443	0·11487

Fold change Inaba OSP IgA									
Dose (CFU)	D1	D4	D7	D15	D29	D57	D85	D180	max FC
10^5	1·00	2·49	5·14	7·61	7·62	8·38	7·52	6·21	8·38
10^5	1·00	0·79	1·08	1·12	1·09	1·06	1·10		1·12
10^5	1·00	1·19	1·38	29·36	7·33	3·66	5·42		29·36
10^6	1·00	1·95	8·60	1999·18	152·81	71·01	60·58		1999·18
10^6	1·00	1·09	7·45	60·95	14·03	6·73	5·28	4·29	60·95
10^6	1·00	2·05	2·77	1240·03	189·62	38·05	10·93	8·61	1240·03
10^7	1·00	0·83	0·78	1·30	2·69	3·55	5·05	5·22	5·22
10^7	1·00	0·98	3·26	4·43	3·47	2·25	1·05	0·14	4·43
10^7	1·00	0·93	0·69	12·30	1·88	0·74	0·85	0·10	12·30
10^8	1·00	1·00	1·12	3·46	1·48	2·42	3·02	6·44	6·44
10^8	1·00	1·04	0·65	3·58	1·43	1·23	3·93	5·15	5·15
10^8	1·00	0·66	35·80	249·75	40·15	9·80	11·33	11·28	249·75
10^9	1·00	0·75	2·99	48·36	15·28	5·54	2·42	1·86	48·36
10^9	1·00	0·95	1·11	1·12	6·51	5·11	9·68		9·68
10^9	1·00	0·88	1·13	1·82	2·12	1·94	2·12	3·26	3·26
10^{10}	1·00	0·45	3·57	10·80	4·87		4·38	4·59	10·80
10^{10}	1·00	1·11	4·28	34·35			15·38	4·92	34·35
10^{10}	1·00	0·59	49·77	1419·07	184·15	54·20	46·14	54·76	1419·07

Dose-Expansion Module

Inaba OSP IgA (RAU)									
Dose (CFU)	D1	D4	D7	D15	D29	D57	D85	D180	max
0	0·00168	0·00216	0·00216	0·00181	0·00216	0·00180	0·00221	0·00139	0·00221
0	0·00082	0·00033	0·00118	0·00059	0·00083	0·00052	0·00116	0·00088	0·00118
0	0·00088	0·00142	0·00042	0·00071	0·00124	0·00098	0·00108	0·00047	0·00142
0	0·00795	0·00644	0·00818	0·00584	0·00395	0·00107	0·00085	0·00069	0·00818
0	0·00049	0·00041	0·00111	0·00077	0·00094	0·00065	0·00057	0·00050	0·00111
0	0·00082	0·00142	0·00080	0·00050	0·00052	0·00054	0·00055	0·00043	0·00142
0	0·00095	0·00073	0·00093	0·00081	0·00142	0·00077	0·00028	0·00071	0·00142
2×10^7	0·00097	0·00152	0·00281	0·01799	0·00504	0·00448	0·00218	0·00154	0·01799
2×10^7	0·00104	0·00136	0·00384	4·00331	0·80326	0·07279	0·04657	0·02318	4·00331
2×10^7	0·00265	0·00265	0·01081	0·02800	0·01750	0·00802	0·00552	0·00322	0·02800
2×10^7	0·00238	0·00195	0·00218	0·00144	0·00193	0·00295	0·00201	0·00080	0·00295
2×10^7	0·00189	0·00186	0·00289	0·32011	0·03853	0·02678	0·03879	0·03264	0·32011
2×10^7	0·00191	0·00195	0·00311	0·03841	0·01760	0·00554	0·00458	0·00238	0·03841
2×10^7	0·00368		0·00244	0·00315	0·00404	0·00308	0·00196		0·00404
2×10^7	0·00121	0·00214	0·00243	0·00884	0·00831	0·00326	0·00224	0·00097	0·00884
2×10^7	0·00195	0·00237	0·00392	0·01994	0·01552	0·00686	0·00632	0·00543	0·01994
2×10^7	0·00242	0·00295	0·00356	0·24514	0·01111	0·00458	0·00363	0·00226	0·24514
2×10^7	0·00067	0·00066	0·00283	0·03527	0·02621	0·01573	0·01492	0·01323	0·03527
2×10^7	0·00092	0·00106	0·00156	0·00133	0·00197	0·00291	0·00382	0·00624	0·00624
2×10^7	0·00028	0·00015	0·00236	0·02150	0·00751	0·00211	0·00125	0·00050	0·02150
2×10^7	0·00056	0·00048	0·00345	0·07314	0·02480	0·00788		0·00346	0·07314
2×10^8	0·00342	0·00382	0·00710	0·01857	0·01686	0·01321	0·00872	0·00665	0·01857
2×10^8	0·00411	0·00500	0·00457	0·00543	0·00550	0·00523	0·00420	0·00243	0·00550
2×10^8	0·00079	0·00045	0·00138	0·09273	0·00891	0·00669	0·00599	0·00542	0·09273
2×10^8	0·00300	0·00358	0·00464	0·00716	0·00928	0·01226	0·00552	0·00590	0·01226
2×10^8	0·00049	0·00037	0·00074	0·00306	0·00197	0·00065	0·00047	0·00029	0·00306
2×10^8	0·00171	0·00160	0·00142	0·01651					0·01651
2×10^8	0·00067	0·00017	0·00034	0·00178	0·00110	0·00119	0·00141	0·00106	0·00178
2×10^8	0·00112	0·00047	0·00100	0·00097	0·00044	0·00059	0·00070	0·00073	0·00112
2×10^8	0·00743	0·00677	0·01973	0·06637	0·02397	0·00980	0·00731	0·00505	0·06637
2×10^8	0·00071	0·00113	0·00158	0·01213	0·00951	0·00520	0·00477	0·00315	0·01213
2×10^8	0·00033	0·00041	0·00047	0·00287	0·00165	0·00219	0·00172	0·00195	0·00287
2×10^8	0·00127	0·00247	0·00257	0·08704	0·01689		0·00551	0·00531	0·08704
2×10^8	0·00042	0·00036	0·00082	0·00137	0·00111	0·00165	0·00205	0·00471	0·00471

Fold change Inaba OSP IgA									
Dose (CFU)	D1	D4	D7	D15	D29	D57	D85	D180	max FC
0	1·00	1·29	1·29	1·08	1·29	1·07	1·32	0·83	1·32
0	1·00	0·40	1·45	0·72	1·01	0·63	1·42	1·07	1·45
0	1·00	1·60	0·48	0·80	1·40	1·11	1·22	0·53	1·60
0	1·00	0·81	1·03	0·73	0·50	0·13	0·11	0·09	1·03
0	1·00	0·85	2·29	1·58	1·94	1·34	1·18	1·04	2·29
0	1·00	1·74	0·98	0·61	0·64	0·66	0·67	0·53	1·74
0	1·00	0·76	0·98	0·85	1·49	0·81	0·30	0·75	1·49
2×10^7	1·00	1·57	2·90	18·56	5·20	4·62	2·24	1·58	18·56
2×10^7	1·00	1·31	3·70	3859·95	774·50	70·18	44·90	22·35	3859·95
2×10^7	1·00	1·00	4·08	10·58	6·61	3·03	2·08	1·22	10·58
2×10^7	1·00	0·82	0·92	0·60	0·81	1·24	0·85	0·34	1·24
2×10^7	1·00	0·99	1·53	169·62	20·42	14·19	20·56	17·30	169·62
2×10^7	1·00	1·02	1·62	20·09	9·20	2·90	2·39	1·25	20·09
2×10^7	1·00		0·66	0·86	1·10	0·84	0·53		1·10
2×10^7	1·00	1·78	2·01	7·32	6·89	2·70	1·86	0·80	7·32
2×10^7	1·00	1·22	2·02	10·25	7·98	3·52	3·25	2·79	10·25
2×10^7	1·00	1·22	1·47	101·27	4·59	1·89	1·50	0·93	101·27
2×10^7	1·00	0·99	4·25	52·97	39·37	23·63	22·41	19·87	52·97
2×10^7	1·00	1·15	1·70	1·44	2·14	3·16	4·14	6·78	6·78
2×10^7	1·00	0·55	8·38	76·18	26·61	7·49	4·42	1·77	76·18
2×10^7	1·00	0·85	6·12	129·71	43·99	13·98		6·13	129·71
2×10^8	1·00	1·12	2·08	5·43	4·93	3·86	2·55	1·95	5·43
2×10^8	1·00	1·22	1·11	1·32	1·34	1·27	1·02	0·59	1·34
2×10^8	1·00	0·57	1·75	117·10	11·25	8·45	7·56	6·85	117·10
2×10^8	1·00	1·19	1·55	2·38	3·09	4·08	1·84	1·96	4·08
2×10^8	1·00	0·75	1·53	6·30	4·06	1·33	0·97	0·60	6·30
2×10^8	1·00	0·94	0·83	9·64					9·64
2×10^8	1·00	0·25	0·51	2·64	1·63	1·76	2·09	1·57	2·64
2×10^8	1·00	0·42	0·89	0·86	0·39	0·53	0·62	0·65	0·89
2×10^8	1·00	0·91	2·66	8·93	3·23	1·32	0·98	0·68	8·93
2×10^8	1·00	1·57	2·21	16·98	13·31	7·28	6·68	4·41	16·98
2×10^8	1·00	1·24	1·43	8·66	4·98	6·60	5·17	5·87	8·66
2×10^8	1·00	1·94	2·02	68·60	13·31		4·34	4·18	68·60
2×10^8	1·00	0·85	1·95	3·27	2·63	3·93	4·87	11·20	11·20

	RAU		
	D1	D39	
V. cholerae	0·00082	1·42989	
	0·00110	0·06328	
	0·00375	0·00401	
	0·00309	0·39626	
	0·00288	0·74963	
	D1	D11	
Vaxchora D11 Challenge	0·00130	0·01043	
	0·00580	0·00587	
	0·01280	0·17380	
	0·00130	0·00212	
	0·00159	0·05441	
	0·00209	0·05283	
	D1	D11	D91
Vaxchora D91 Challenge	0·00019	0·00025	0·00034
	0·00064	0·06443	0·00206
	0·00031	0·01453	0·00080
	0·00262	0·01789	0·00357
	0·00113	0·02759	0·00466
	0·00046	0·00476	0·00186

	RAU fold change			max FC
	D1	D39		
V. cholerae	1·00	1750·61		1750·61
	1·00	57·63		57·63
	1·00	1·07		1·07
	1·00	128·30		128·30
	1·00	260·21		260·21
	D1	D11		
Vaxchora D11 Challenge	1·00	8·01		8·01
	1·00	1·01		1·01
	1·00	13·58		13·58
	1·00	1·63		1·63
	1·00	34·29		34·29
	1·00	25·32		25·32
	D1	D11	D91	
Vaxchora D91 Challenge	1·00	1·29	1·74	1·74
	1·00	99·92	3·20	99·92
	1·00	46·30	2·56	46·30
	1·00	6·83	1·36	6·83
	1·00	24·46	4·13	24·46
	1·00	10·25	4·01	10·25

Dose-Escalation Module

Ogawa OSP IgM (RAU)									
Dose (CFU)	D1	D4	D7	D15	D29	D57	D85	D180	max
10^5	0·00333	0·00166	0·00468	0·04103	0·02014	0·01370	0·01197	0·01685	0·04103
10^5	0·00079	0·00053	0·00039	0·00253	0·00345	0·00236	0·00244		0·00345
10^5	0·01259	0·01316	0·01319	0·01754	0·01359	0·00987	0·01069		0·01754
10^6	0·01044	0·00873	0·05409	0·56475	0·26043	0·08547	0·05172		0·56475
10^6	0·00076	0·00101	0·00146	0·00554	0·00366	0·00262	0·00176	0·00118	0·00554
10^6	0·00118	0·00099	0·01641	1·13709	0·30592	0·12220	0·09650	0·02275	1·13709
10^7	0·00223	0·00188	0·01992	0·50226	0·23810	0·13490	0·07292	0·02145	0·50226
10^7	0·00162	0·00169	0·01614	0·28529	0·21542	0·08579	0·02989	0·00738	0·28529
10^7	0·00303	0·00357	0·00286	0·01678	0·00812	0·00567	0·00516	0·00404	0·01678
10^8	0·00108	0·00106	0·00247	0·11431	0·06306	0·02353	0·01882	0·00874	0·11431
10^8	0·00210	0·00167	0·00389	0·11895	0·03692	0·01398	0·00621	0·00468	0·11895
10^8	0·00105	0·00097	0·00278	0·02695	0·01200	0·00312	0·00247	0·00433	0·02695
10^9	0·00046	0·00014	0·02686	0·20038	0·05962	0·01294	0·00429	0·00289	0·20038
10^9	0·00121	0·00103	0·00047	0·00218	0·00600	0·00707	0·01580		0·01580
10^9	0·00414	0·00428	0·00558	0·10099	0·02168	0·01780	0·02207	0·05674	0·10099
10^{10}	0·00149	0·00185	0·00305	0·00897	0·00726		0·00350	0·00397	0·00897
10^{10}	0·00903	0·00963	0·03222	0·14568			0·01764	0·00956	0·14568
10^{10}	0·00277	0·00272	0·37863	1·04964	0·26434	0·09112	0·02412	0·01330	1·04964

Fold change Ogawa OSP IgM									
Dose (CFU)	D1	D4	D7	D15	D29	D57	D85	D180	max FC
10^5	1·00	0·50	1·41	12·33	6·05	4·12	3·60	5·06	12·33
10^5	1·00	0·66	0·49	3·18	4·34	2·97	3·07		4·34
10^5	1·00	1·05	1·05	1·39	1·08	0·78	0·85		1·39
10^6	1·00	0·84	5·18	54·09	24·94	8·19	4·95		54·09
10^6	1·00	1·32	1·91	7·25	4·79	3·43	2·30	1·55	7·25
10^6	1·00	0·83	13·85	959·72	258·21	103·14	81·45	19·20	959·72
10^7	1·00	0·84	8·92	224·79	106·56	60·38	32·63	9·60	224·79
10^7	1·00	1·04	9·99	176·59	133·34	53·10	18·50	4·57	176·59
10^7	1·00	1·18	0·95	5·55	2·68	1·87	1·71	1·33	5·55
10^8	1·00	0·99	2·29	106·22	58·59	21·86	17·48	8·12	106·22
10^8	1·00	0·79	1·85	56·77	17·62	6·67	2·97	2·23	56·77
10^8	1·00	0·93	2·65	25·74	11·46	2·98	2·36	4·13	25·74
10^9	1·00	0·30	58·98	440·01	130·91	28·41	9·42	6·34	440·01
10^9	1·00	0·85	0·39	1·81	4·98	5·86	13·10		13·10
10^9	1·00	1·03	1·35	24·39	5·24	4·30	5·33	13·70	24·39
10^{10}	1·00	1·24	2·04	6·01	4·86		2·34	2·66	6·01
10^{10}	1·00	1·07	3·57	16·14			1·95	1·06	16·14
10^{10}	1·00	0·98	136·74	379·08	95·47	32·91	8·71	4·80	379·08

Dose-Expansion Module

Ogawa OSP IgM (RAU)									
Dose (CFU)	D1	D4	D7	D15	D29	D57	D85	D180	max
0	0·00393	0·00358	0·00348	0·00367	0·00377	0·00342	0·00435	0·00471	0·00471
0	0·00088	0·00091	0·00086	0·00105	0·00061	0·00083	0·00059	0·00071	0·00105
0	0·00719	0·00775	0·00804	0·00742	0·00839	0·00658	0·00437	0·00696	0·00839
0	0·01632	0·01702	0·01805	0·01455	0·01612	0·03393	0·01692	0·01141	0·03393
0	0·00540	0·00500	0·00668	0·00635	0·00683	0·00289	0·00343	0·00208	0·00683
0	0·00922	0·01003	0·01033	0·00557	0·00377	0·00592	0·00632	0·00818	0·01033
0	0·00906	0·00742	0·01104	0·01006	0·01381	0·01450	0·00575	0·01512	0·01512
$2×10^7$	0·01768	0·02049	0·02108	0·03254	0·02775	0·01785	0·02517	0·02646	0·03254
$2×10^7$	0·01969	0·02122	0·09912	2·77280	1·66929	0·60782	0·29698	0·10815	2·77280
$2×10^7$	0·00432	0·00467	0·05574	0·37278	0·21210	0·07035	0·02760	0·01294	0·37278
$2×10^7$	0·00437	0·00644	0·00617	0·02062	0·00742	0·00659	0·00589	0·00152	0·02062
$2×10^7$	0·00403	0·00353	0·02376	0·27542	0·10344	0·02921	0·01780	0·00335	0·27542
$2×10^7$	0·00172	0·00164	0·02717	0·16725	0·10500	0·02376	0·01750	0·00246	0·16725
$2×10^7$	0·01087		0·01302	0·06912	0·02820	0·01763	0·01095		0·06912
$2×10^7$	0·00959	0·01022	0·02270	1·67601	1·06496	0·49654	0·26575	0·05372	1·67601
$2×10^7$	0·00022	0·00051	0·00032	0·00154	0·00114	0·00082	0·00085	0·00032	0·00154
$2×10^7$	0·00858	0·01065	0·13789	2·47046	0·77335	0·18281	0·11008	0·06928	2·47046
$2×10^7$	0·00331	0·00444	0·01095	0·08820	0·08879	0·04942	0·02892	0·02063	0·08879
$2×10^7$	0·00156	0·00164	0·00286	0·02562	0·02084	0·02080	0·02200	0·01866	0·02562
$2×10^7$	0·00189	0·00166	0·00284	0·00553	0·00299	0·00231	0·00218	0·00261	0·00553
$2×10^7$	0·00386	0·00231	0·02341	0·43560	0·36247	0·15172		0·03788	0·43560
$2×10^8$	0·00566	0·00566	0·00772	0·02420	0·02532	0·01062	0·00787	0·00577	0·02532
$2×10^8$	0·00153	0·00151	0·00294	0·05252	0·02339	0·00668	0·00481	0·00203	0·05252
$2×10^8$	0·00376	0·00315	0·01289	0·11659	0·02460	0·01327	0·00897	0·00267	0·11659
$2×10^8$	0·02280	0·02315	0·05721	0·11660	0·11261	0·09070	0·11108	0·10286	0·11660
$2×10^8$	0·00325	0·00395	0·01203	0·06408	0·04840	0·00893	0·00596	0·00434	0·06408
$2×10^8$	0·00538	0·00548	0·01047	0·36779					0·36779
$2×10^8$	0·00769	0·00931	0·01095	0·10439	0·06109	0·02945	0·02604	0·02080	0·10439
$2×10^8$	0·00069	0·00077	0·00063	0·02231	0·00679	0·00151	0·00140	0·00057	0·02231
$2×10^8$	0·00331	0·00269	0·15740	4·28939	1·05323	0·23211	0·15355	0·07635	4·28939
$2×10^8$	0·01143	0·01262	0·17239	1·68965	1·02668	0·24002	0·09532	0·03970	1·68965
$2×10^8$	0·00095	0·00134	0·05602	5·67692	2·05979	0·26296	0·07343	0·00936	5·67692
$2×10^8$	0·00105	0·00118	0·02336	0·16031	0·06253		0·02294	0·01994	0·16031
$2×10^8$	0·00070	0·00029	0·00050	0·00103	0·00061	0·00135	0·00131	0·00156	0·00156

Fold change Ogawa OSP IgM									
Dose (CFU)	D1	D4	D7	D15	D29	D57	D85	D180	max FC
0	1·00	0·91	0·89	0·93	0·96	0·87	1·11	1·20	1·20
0	1·00	1·04	0·98	1·19	0·70	0·94	0·67	0·80	1·19
0	1·00	1·08	1·12	1·03	1·17	0·92	0·61	0·97	1·17
0	1·00	1·04	1·11	0·89	0·99	2·08	1·04	0·70	2·08
0	1·00	0·93	1·24	1·18	1·27	0·54	0·63	0·39	1·27
0	1·00	1·09	1·12	0·60	0·41	0·64	0·69	0·89	1·12
0	1·00	0·82	1·22	1·11	1·52	1·60	0·63	1·67	1·67
$2×10^7$	1·00	1·16	1·19	1·84	1·57	1·01	1·42	1·50	1·84
$2×10^7$	1·00	1·08	5·03	140·81	84·77	30·87	15·08	5·49	140·81
$2×10^7$	1·00	1·08	12·91	86·33	49·12	16·29	6·39	3·00	86·33
$2×10^7$	1·00	1·47	1·41	4·71	1·70	1·51	1·35	0·35	4·71
$2×10^7$	1·00	0·88	5·90	68·40	25·69	7·25	4·42	0·83	68·40
$2×10^7$	1·00	0·96	15·84	97·50	61·21	13·85	10·20	1·44	97·50
$2×10^7$	1·00		1·20	6·36	2·59	1·62	1·01		6·36
$2×10^7$	1·00	1·07	2·37	174·80	111·07	51·79	27·72	5·60	174·80
$2×10^7$	1·00	2·30	1·43	6·99	5·20	3·74	3·86	1·43	6·99
$2×10^7$	1·00	1·24	16·07	287·88	90·12	21·30	12·83	8·07	287·88
$2×10^7$	1·00	1·34	3·31	26·63	26·80	14·92	8·73	6·23	26·80
$2×10^7$	1·00	1·05	1·83	16·41	13·34	13·32	14·09	11·95	16·41
$2×10^7$	1·00	0·88	1·50	2·93	1·59	1·22	1·15	1·38	2·93
$2×10^7$	1·00	0·60	6·07	112·93	93·97	39·33		9·82	112·93
$2×10^8$	1·00	1·00	1·37	4·28	4·48	1·88	1·39	1·02	4·48
$2×10^8$	1·00	0·99	1·93	34·38	15·31	4·37	3·15	1·33	34·38
$2×10^8$	1·00	0·84	3·43	31·00	6·54	3·53	2·39	0·71	31·00
$2×10^8$	1·00	1·02	2·51	5·11	4·94	3·98	4·87	4·51	5·11
$2×10^8$	1·00	1·22	3·70	19·73	14·90	2·75	1·83	1·34	19·73
$2×10^8$	1·00	1·02	1·95	68·37					68·37
$2×10^8$	1·00	1·21	1·42	13·58	7·95	3·83	3·39	2·71	13·58
$2×10^8$	1·00	1·10	0·91	32·11	9·77	2·17	2·01	0·82	32·11
$2×10^8$	1·00	0·81	47·59	1297·03	318·48	70·19	46·43	23·09	1297·03
$2×10^8$	1·00	1·10	15·08	147·84	89·83	21·00	8·34	3·47	147·84
$2×10^8$	1·00	1·41	58·96	5974·49	2167·76	276·75	77·28	9·86	5974·49
$2×10^8$	1·00	1·12	22·29	152·97	59·67		21·89	19·03	152·97
$2×10^8$	1·00	0·42	0·72	1·48	0·88	1·94	1·88	2·24	2·24

	RAU		
	D1	D39	
V. cholerae	0·00088	0·05204	
	0·00349	0·52353	
	0·00079	0·00210	
	0·01538	2·03912	
	0·00378	0·10988	
	D1	D11	
Vaxchora D11 Challenge	0·00103	0·06372	
	0·00904	0·01051	
	0·00066	0·03332	
	0·00057	0·00140	
	0·00296	0·00386	
	0·01025	0·02432	
	D1	D11	D91
Vaxchora D91 Challenge	0·00028	0·00696	0·00173
	0·00113	0·01024	0·00114
	0·00013	0·00799	0·00131
	0·00637	0·01315	0·01167
	0·00710	0·01048	0·00700
	0·00013	0·00202	0·00020

	RAU fold change			max FC
	D1	D39		
V. cholerae	1·00	59·12		59·12
	1·00	149·89		149·89
	1·00	2·65		2·65
	1·00	132·54		132·54
	1·00	29·06		29·06
	D1	D11		
Vaxchora D11 Challenge	1·00	62·14		62·14
	1·00	1·16		1·16
	1·00	50·26		50·26
	1·00	2·46		2·46
	1·00	1·30		1·30
	1·00	2·37		2·37
	D1	D11	D91	
Vaxchora D91 Challenge	1·00	25·20	6·26	25·20
	1·00	9·09	1·01	9·09
	1·00	60·81	9·95	60·81
	1·00	2·06	1·83	2·06
	1·00	1·48	0·99	1·48
	1·00	16·07	1·63	16·07

Dose-Escalation Module

Ogawa OSP IgG (RAU)									
**Dose (CFU)**	**D1**	**D4**	**D7**	**D15**	**D29**	**D57**	**D85**	**D180**	**max**
10^5	0·00064	0·00005	0·00019	0·00053	0·00060	0·00028	0·00039	0·00094	0·00094
10^5	0·00001		0·00035	0·00030	0·00042	0·00030	0·00057		0·00057
10^5	0·00032	0·00049	0·00054	0·00470	0·00259	0·00064	0·00133		0·00470
10^6	0·00028	0·00071	0·00078	0·00347	0·00216	0·00239	0·00278		0·00347
10^6	0·00105	0·00071	0·00028	0·00119	0·00035	0·00085	0·00057	0·00092	0·00119
10^6	0·00211	0·00199	0·01323	5·08877	2·35763	0·86894	0·36844	0·08868	5·08877
10^7	0·00163	0·00144	0·00131	0·00284	0·00294	0·00702	0·00931	0·01666	0·01666
10^7	0·00057	0·00085	0·00092	0·00239	0·00205	0·00169	0·00289	0·00359	0·00359
10^7	0·00169	0·00138	0·00181	0·00181	0·00157	0·00169	0·00125	0·00134	0·00181
10^8	0·00131	0·00119	0·00105	0·00144	0·00138	0·00119	0·00251	0·00616	0·00616
10^8	0·00078	0·00092	0·00105	0·00560	0·00239	0·00228	0·00156	0·00187	0·00560
10^8	0·00150	0·00175	0·00216	0·00175	0·00157	0·00134	0·00470	0·01584	0·01584
10^9	0·00085	0·00057	0·00092	0·00181	0·00131	0·00129	0·00171	0·00223	0·00223
10^9	0·00071	0·00078	0·00112	0·00090	0·00396	0·00995	0·04516		0·04516
10^9	0·00156	0·00107	0·00156	0·00161	0·00101	0·00166	0·00156	0·00282	0·00282
10^{10}	0·00043	0·00090	0·00766	0·14250	0·11861		0·03381	0·01189	0·14250
10^{10}	0·00025	0·00067	0·00084	0·00140			0·00049	0·00055	0·00140
10^{10}	0·00134	0·00134	0·00084	0·00140	0·00129	0·00192	0·00182	0·00258	0·00258

Fold change Ogawa OSP IgG									
**Dose (CFU)**	**D1**	**D4**	**D7**	**D15**	**D29**	**D57**	**D85**	**D180**	**max FC**
10^5	1·00	0·08	0·30	0·83	0·94	0·43	0·61	1·47	1·47
10^5	1·00		66·62	58·41	80·25	58·41	109·76		109·76
10^5	1·00	1·53	1·71	14·77	8·14	2·01	4·18		14·77
10^6	1·00	2·58	2·83	12·57	7·84	8·67	10·07		12·57
10^6	1·00	0·68	0·26	1·13	0·33	0·81	0·54	0·87	1·13
10^6	1·00	0·94	6·28	2415·90	1119·29	412·53	174·92	42·10	2415·90
10^7	1·00	0·89	0·81	1·74	1·81	4·31	5·72	10·23	10·23
10^7	1·00	1·49	1·61	4·21	3·60	2·97	5·08	6·31	6·31
10^7	1·00	0·82	1·07	1·07	0·93	1·00	0·74	0·79	1·07
10^8	1·00	0·90	0·80	1·10	1·05	0·90	1·91	4·69	4·69
10^8	1·00	1·18	1·35	7·17	3·06	2·92	1·99	2·39	7·17
10^8	1·00	1·16	1·44	1·16	1·04	0·89	3·12	10·53	10·53
10^9	1·00	0·67	1·08	2·13	1·55	1·51	2·02	2·62	2·62
10^9	1·00	1·10	1·57	1·26	5·57	13·99	63·49		63·49
10^9	1·00	0·69	1·00	1·03	0·65	1·07	1·00	1·81	1·81
10^{10}	1·00	2·07	17·67	328·57	273·49		77·96	27·42	328·57
10^{10}	1·00	2·65	3·33	5·52			1·95	2·19	5·52
10^{10}	1·00	1·00	0·63	1·04	0·96	1·43	1·36	1·92	1·92

Dose-Expansion Module

Ogawa OSP IgG (RAU)									
**Dose (CFU)**	**D1**	**D4**	**D7**	**D15**	**D29**	**D57**	**D85**	**D180**	**max**
0	0·00127	0·00148	0·00192	0·00096	0·00178	0·00194	0·00099	0·00196	0·00196
0	0·00383	0·00465	0·00391	0·00286	0·00328	0·00313	0·00333	0·00306	0·00465
0	0·00283	0·00269	0·00244	0·00310	0·00342	0·00326	0·00364	0·00039	0·00364
0	0·00813	0·00877	0·00848	0·00743	0·00829	0·00175	0·00094	0·00080	0·00877
0	0·00495	0·00586	0·00528	0·00532	0·00471	0·00001	0·00023		0·00586
0	0·00249	0·00205	0·00306	0·00015	0·00015	0·00032	0·00002		0·00306
0	0·00018			0·00039		0·00019		0·00004	0·00039
2×10^7	0·00895	0·00598	0·00569	0·00592	0·00618	0·00629	0·00611	0·00833	0·00895
2×10^7	0·00403	0·00324	0·00726	0·18673	0·15346	0·10516	0·06851	0·01925	0·18673
2×10^7	0·00211	0·00172	0·00160	0·00246	0·00326	0·00207	0·00242	0·00152	0·00326
2×10^7	0·00253	0·00258	0·00255	0·00182	0·00262	0·00242	0·00176	0·00028	0·00262
2×10^7	0·00423	0·00439	0·00355	0·03180	0·01977	0·01358	0·01093	0·00389	0·03180
2×10^7	0·00260	0·00229	0·00228	0·00319	0·00352	0·00263	0·00296	0·00038	0·00352
2×10^7	0·00641		0·00590	0·00837	0·00740	0·00740	0·00464		0·00837
2×10^7	0·00170	0·00154	0·00144	0·00215	0·00244	0·00008		0·00001	0·00244
2×10^7	0·00590	0·00600	0·00571	0·00542	0·00527	0·00101	0·00132	0·00322	0·00600
2×10^7	0·00405	0·00430	0·00395	0·01063	0·00354	0·00750	0·00952	0·03584	0·03584
2×10^7			0·00005		0·00046	0·00047	0·00126	0·00256	0·00256
2×10^7	0·00064	0·00030	0·00036	0·00006	0·00089	0·00077	0·00104	0·00434	0·00434
2×10^7		0·00004	0·00049	0·00056	0·00016	0·00015	0·00016	0·00016	0·00056
2×10^7	0·00001	0·00012	0·00001	0·00620	0·00617	0·00335		0·00919	0·00919
2×10^8	0·00310	0·00349	0·00469	0·00688	0·00458	0·00647	0·01801	0·03126	0·03126
2×10^8	0·00040	0·00153	0·00078	0·00078	0·00076	0·00086	0·00106	0·00082	0·00153
2×10^8	0·00355	0·00318	0·00326	0·00321	0·00345	0·00701	0·02209	0·02314	0·02314
2×10^8	0·00452	0·00397	0·00403	0·00700	0·00710	0·00954	0·00721	0·01552	0·01552
2×10^8	0·00110	0·00180	0·00313	0·12342	0·09557	0·01816	0·00954	0·00180	0·12342
2×10^8	0·00164	0·00094	0·00042	0·00156					0·00164
2×10^8	0·00408	0·00468	0·00452	0·01236	0·00327	0·00625	0·00894	0·00484	0·01236
2×10^8	0·00127	0·00069	0·00062	0·00211	0·00029	0·00045		0·00008	0·00211
2×10^8	0·00293	0·00244	0·00283	0·00301	0·00151	0·00097	0·00433	0·02118	0·02118
2×10^8	0·00085	0·00108	0·00097	0·01293	0·01229	0·00586	0·00311	0·00184	0·01293
2×10^8	0·00020		0·00028	0·00276	0·00193	0·00709	0·02478	0·05072	0·05072
2×10^8	0·00038	0·00043	0·00008	0·00050	0·00007		0·00107	0·00227	0·00227
2×10^8	0·00008	0·00007	0·00014	0·00060	0·00026	0·00669	0·00638	0·00367	0·00669

Fold change Ogawa OSP IgG									
**Dose (CFU)**	**D1**	**D4**	**D7**	**D15**	**D29**	**D57**	**D85**	**D180**	**max FC**
0	1·00	1·17	1·51	0·76	1·40	1·53	0·78	1·54	1·54
0	1·00	1·21	1·02	0·75	0·86	0·82	0·87	0·80	1·21
0	1·00	0·95	0·86	1·10	1·21	1·15	1·29	0·14	1·29
0	1·00	1·08	1·04	0·91	1·02	0·21	0·12	0·10	1·08
0	1·00	1·18	1·07	1·08	0·95	0·002	0·05		1·18
0	1·00	0·82	1·23	0·06	0·06	0·13	0·01		1·23
0	1·00			2·20		1·08		0·21	2·20
2×10^7	1·00	0·67	0·64	0·66	0·69	0·70	0·68	0·93	0·93
2×10^7	1·00	0·80	1·80	46·33	38·07	26·09	17·00	4·78	46·33
2×10^7	1·00	0·82	0·76	1·17	1·55	0·98	1·15	0·72	1·55
2×10^7	1·00	1·02	1·01	0·72	1·03	0·96	0·70	0·11	1·03
2×10^7	1·00	1·04	0·84	7·52	4·68	3·21	2·59	0·92	7·52
2×10^7	1·00	0·88	0·88	1·23	1·35	1·01	1·14	0·14	1·35
2×10^7	1·00		0·92	1·31	1·15	1·15	0·72		1·31
2×10^7	1·00	0·91	0·84	1·26	1·43	0·05		0·003	1·43
2×10^7	1·00	1·02	0·97	0·92	0·89	0·17	0·22	0·55	1·02
2×10^7	1·00	1·06	0·98	2·63	0·87	1·85	2·35	8·86	8·86
2×10^7			1·00		9·48	9·77	25·99	52·81	52·81
2×10^7	1·00	0·48	0·56	0·09	1·39	1·21	1·63	6·78	6·78
2×10^7		1·00	12·89	14·72	4·31	3·95	4·31	4·31	14·72
2×10^7	1·00	23·53	1·00	1190·09	1184·97	643·75		1762·98	1762·98
2×10^8	1·00	1·13	1·52	2·22	1·48	2·09	5·82	10·10	10·10
2×10^8	1·00	3·81	1·94	1·94	1·89	2·15	2·64	2·05	3·81
2×10^8	1·00	0·90	0·92	0·90	0·97	1·98	6·22	6·52	6·52
2×10^8	1·00	0·88	0·89	1·55	1·57	2·11	1·59	3·43	3·43
2×10^8	1·00	1·64	2·85	112·55	87·15	16·56	8·70	1·64	112·55
2×10^8	1·00	0·57	0·26	0·95					0·95
2×10^8	1·00	1·15	1·11	3·03	0·80	1·53	2·19	1·19	3·03
2×10^8	1·00	0·54	0·48	1·66	0·23	0·35		0·07	1·66
2×10^8	1·00	0·83	0·97	1·03	0·51	0·33	1·48	7·24	7·24
2×10^8	1·00	1·27	1·14	15·16	14·41	6·88	3·65	2·16	15·16
2×10^8	1·00		1·35	13·47	9·41	34·57	120·87	247·44	247·44
2×10^8	1·00	1·15	0·22	1·34	0·19		2·84	6·06	6·06
2×10^8	1·00	0·85	1·62	7·14	3·12	79·75	76·02	43·77	79·75

	RAU		
	D1	D39	
V. cholerae	0·00466	0·02631	
	0·00352	0·03114	
	0·00432	0·00614	
	0·00578	0·11366	
	0·00437	0·00710	
	D1	D11	
Vaxchora D11 Challenge	0·00922	0·01624	
	0·01894	0·01845	
	0·01549	0·01549	
	0·00935	0·00994	
	0·01142	0·01459	
	0·01397	0·01065	
	D1	D11	D91
Vaxchora D91 Challenge	0·00398	0·00500	0·00476
	0·00105	0·00432	0·00393
	0·00627	0·01316	0·01042
	0·00978	0·01542	0·01010
	0·00256	0·00951	0·00990
	0·00300	0·00413	0·00383

	RAU fold change			max FC
	D1	D39		
V. cholerae	1·00	5·64		5·64
	1·00	8·84		8·84
	1·00	1·42		1·42
	1·00	19·66		19·66
	1·00	1·62		1·62
	D1	D11		
Vaxchora D11 Challenge	1·00	1·76		1·76
	1·00	0·97		0·97
	1·00	1·00		1·00
	1·00	1·06		1·06
	1·00	1·28		1·28
	1·00	0·76		0·76
	D1	D11	D91	
Vaxchora D91 Challenge	1·00	1·26	1·20	1·26
	1·00	4·11	3·73	4·11
	1·00	2·10	1·66	2·10
	1·00	1·58	1·03	1·58
	1·00	3·71	3·87	3·87
	1·00	1·38	1·28	1·38

Dose-Escalation Module

Ogawa OSP IgA (RAU)									
Dose (CFU)	D1	D4	D7	D15	D29	D57	D85	D180	max
10^5	0·00071	0·00130	0·00121	0·00604	0·00316	0·00213	0·00177	0·00174	0·00604
10^5	0·00260	0·00242	0·00273	0·00231	0·00269	0·00233	0·00260		0·00273
10^5	0·00230	0·00220	0·00230	0·00450	0·00209	0·00195	0·00200		0·00450
10^6	0·00096	0·00092	0·00327	0·05537	0·00727	0·00362	0·00301		0·05537
10^6	0·00478	0·00285	0·00548	0·00754	0·00395	0·00305	0·00278	0·00233	0·00754
10^6	0·00089	0·00134	0·00511	0·32380	0·04718	0·01249	0·00492	0·00281	0·32380
10^7	0·00073	0·00103	0·00106	0·00428	0·00560	0·00516	0·00443	0·00315	0·00560
10^7	0·00169	0·00165	0·01036	0·02986	0·00840	0·00241	0·00205	0·00050	0·02986
10^7	0·00117	0·00101	0·00067	0·00231	0·00146	0·00088	0·00097	0·00002	0·00231
10^8	0·00154	0·00175	0·00144	0·00722	0·00329	0·00252	0·00265	0·00174	0·00722
10^8	0·00133	0·00118	0·00203	0·05481	0·00898	0·00212	0·00158	0·00133	0·05481
10^8	0·00040	0·00023	0·00063	0·00389	0·00139	0·00116	0·00117	0·00156	0·00389
10^9	0·00101	0·00111	0·00194	0·00394	0·00226	0·00164	0·00108	0·00094	0·00394
10^9	0·00566	0·00457	0·00473	0·00187	0·01704	0·01148	0·01923		0·01923
10^9	0·00026	0·00033	0·00026	0·00091	0·00115	0·00066	0·00050	0·00100	0·00115
10^{10}	0·00003	0·00010	0·00087	0·00070	0·00050		0·00014	0·00014	0·00087
10^{10}	0·00101	0·00108	0·00126	0·00417			0·00233	0·00136	0·00417
10^{10}	0·00009	0·00016	0·00103	0·01074	0·00256	0·00117	0·00168	0·00307	0·01074

Fold change Ogawa OSP IgA									
Dose (CFU)	D1	D4	D7	D15	D29	D57	D85	D180	max FC
10^5	1·00	1·83	1·70	8·52	4·46	3·01	2·50	2·45	8·52
10^5	1·00	0·93	1·05	0·89	1·03	0·90	1·00		1·05
10^5	1·00	0·96	1·00	1·95	0·91	0·85	0·87		1·95
10^6	1·00	0·95	3·40	57·67	7·57	3·77	3·14		57·67
10^6	1·00	0·60	1·15	1·58	0·83	0·64	0·58	0·49	1·58
10^6	1·00	1·50	5·71	362·13	52·76	13·96	5·50	3·14	362·13
10^7	1·00	1·40	1·45	5·84	7·64	7·04	6·05	4·30	7·64
10^7	1·00	0·98	6·14	17·70	4·98	1·43	1·22	0·30	17·70
10^7	1·00	0·86	0·58	1·97	1·24	0·75	0·83	0·02	1·97
10^8	1·00	1·14	0·94	4·70	2·14	1·64	1·73	1·13	4·70
10^8	1·00	0·89	1·53	41·36	6·78	1·60	1·19	1·00	41·36
10^8	1·00	0·57	1·57	9·69	3·47	2·88	2·93	3·89	9·69
10^9	1·00	1·10	1·92	3·89	2·24	1·62	1·06	0·92	3·89
10^9	1·00	0·81	0·84	0·33	3·01	2·03	3·40		3·40
10^9	1·00	1·27	1·01	3·52	4·48	2·55	1·96	3·88	4·48
10^{10}	1·00	3·13	26·62	21·55	15·48		4·17	4·25	26·62
10^{10}	1·00	1·07	1·26	4·15			2·32	1·36	4·15
10^{10}	1·00	1·67	10·90	113·41	27·04	12·30	17·71	32·41	113·41

Dose-Expansion Module

Ogawa OSP IgA (RAU)									
Dose (CFU)	D1	D4	D7	D15	D29	D57	D85	D180	max
0	0·00255	0·00266	0·00276	0·00316	0·00320	0·00304	0·00357	0·00260	0·00357
0	0·00234	0·00166	0·00200	0·00179	0·00168	0·00157	0·00166	0·00170	0·00234
0	0·00242	0·00270	0·00207	0·00174	0·00217	0·00247	0·00148	0·00146	0·00270
0	0·00370	0·00455	0·00479	0·00312	0·00281	0·00173	0·00141	0·00111	0·00479
0	0·00118	0·00086	0·00155	0·00284	0·00104	0·00126	0·00107	0·00064	0·00284
0	0·00185	0·00191	0·00179	0·00081	0·00068	0·00079	0·00086	0·00061	0·00191
0	0·00133	0·00110	0·00148	0·00141	0·00179	0·00115	0·00072	0·00092	0·00179
2×10^7	0·00102	0·00179	0·00125	0·00567	0·00201	0·00149	0·00240	0·00176	0·00567
2×10^7	0·00195	0·00251	0·00330	0·24397	0·06934	0·02000	0·01342	0·00554	0·24397
2×10^7	0·00220	0·00257	0·00363	0·01226	0·00756	0·00379	0·00265	0·00135	0·01226
2×10^7	0·00373	0·00273	0·00195	0·00247	0·00296	0·00354	0·00283	0·00124	0·00373
2×10^7	0·00201	0·00188	0·00695	0·16153	0·02724	0·00582	0·00526	0·00365	0·16153
2×10^7	0·00257	0·00304	0·03470	0·23296	0·10663	0·02061	0·01426	0·00345	0·23296
2×10^7	0·00367		0·00291	0·02740	0·00698	0·00341	0·00220		0·02740
2×10^7	0·00180	0·00305	0·01172	0·03785	0·01655	0·00450	0·00327	0·00188	0·03785
2×10^7	0·00180	0·00201	0·00210	0·00217	0·00233	0·00220	0·00231	0·00255	0·00255
2×10^7	0·00182	0·00186	0·00480	0·43940	0·02034	0·00976	0·00708	0·00499	0·43940
2×10^7	0·00084	0·00059	0·00339	0·01923	0·01320	0·00507	0·00439	0·00443	0·01923
2×10^7	0·00136	0·00152	0·00268	0·00120	0·00196	0·00278	0·00388	0·00360	0·00388
2×10^7	0·00023	0·00012	0·00051	0·00130	0·00104	0·00052	0·00062	0·00008	0·00130
2×10^7	0·00105	0·00088	0·00446	0·09925	0·03131	0·01013		0·00494	0·09925
2×10^8	0·00381	0·00526	0·00344	0·00664	0·00580	0·00419	0·00364	0·00401	0·00664
2×10^8	0·00411	0·00520	0·00488	0·00508	0·00596	0·00554	0·00457	0·00207	0·00596
2×10^8	0·00079	0·00118	0·00108	0·00266	0·00192	0·00237	0·00200	0·00179	0·00266
2×10^8	0·00309	0·00257	0·03772	0·05365	0·02586	0·00842	0·00426	0·00286	0·05365
2×10^8	0·00097	0·00123	0·00450	0·01886	0·00898	0·00093	0·00042	0·00002	0·01886
2×10^8	0·00192	0·00099	0·00092	0·00321					0·00321
2×10^8	0·00149	0·00135	0·00160	0·01561	0·00239	0·00198	0·00201	0·00155	0·01561
2×10^8	0·00170	0·00126	0·00141	0·00272	0·00081	0·00066	0·00103	0·00107	0·00272
2×10^8	0·00388	0·00413	0·01759	0·07248	0·02783	0·01112	0·00821	0·00548	0·07248
2×10^8	0·00212	0·00232	0·13754	2·26371	0·75668	0·04771	0·02122	0·01139	2·26371
2×10^8	0·00086	0·00082	0·00170	0·02312	0·01066	0·00606	0·00284	0·00115	0·02312
2×10^8	0·00224	0·00393	0·00316	0·02160	0·00495		0·00342	0·00294	0·02160
2×10^8	0·00056	0·00052	0·00088	0·00061	0·00100	0·00078	0·00133	0·00157	0·00157

Fold change Ogawa OSP IgA									
Dose (CFU)	D1	D4	D7	D15	D29	D57	D85	D180	max FC
0	1·00	1·04	1·08	1·24	1·25	1·19	1·40	1·02	1·40
0	1·00	0·71	0·85	0·76	0·72	0·67	0·71	0·72	0·85
0	1·00	1·12	0·86	0·72	0·90	1·02	0·61	0·60	1·12
0	1·00	1·23	1·29	0·84	0·76	0·47	0·38	0·30	1·29
0	1·00	0·73	1·32	2·41	0·88	1·07	0·91	0·55	2·41
0	1·00	1·03	0·97	0·44	0·37	0·43	0·47	0·33	1·03
0	1·00	0·83	1·11	1·06	1·34	0·86	0·54	0·69	1·34
2×10^7	1·00	1·75	1·22	5·55	1·97	1·46	2·35	1·72	5·55
2×10^7	1·00	1·29	1·69	124·90	35·50	10·24	6·87	2·84	124·90
2×10^7	1·00	1·17	1·65	5·57	3·43	1·72	1·20	0·61	5·57
2×10^7	1·00	0·73	0·52	0·66	0·79	0·95	0·76	0·33	0·95
2×10^7	1·00	0·93	3·46	80·26	13·54	2·89	2·61	1·81	80·26
2×10^7	1·00	1·18	13·51	90·72	41·52	8·02	5·55	1·34	90·72
2×10^7	1·00		0·79	7·48	1·90	0·93	0·60		7·48
2×10^7	1·00	1·69	6·50	20·99	9·18	2·50	1·81	1·04	20·99
2×10^7	1·00	1·12	1·17	1·21	1·29	1·22	1·28	1·41	1·41
2×10^7	1·00	1·02	2·64	241·67	11·19	5·37	3·89	2·75	241·67
2×10^7	1·00	0·70	4·03	22·84	15·68	6·02	5·21	5·26	22·84
2×10^7	1·00	1·12	1·97	0·88	1·44	2·05	2·85	2·65	2·85
2×10^7	1·00	0·54	2·24	5·71	4·57	2·31	2·74	0·35	5·71
2×10^7	1·00	0·83	4·25	94·39	29·78	9·63		4·70	94·39
2×10^8	1·00	1·38	0·90	1·74	1·52	1·10	0·95	1·05	1·74
2×10^8	1·00	1·27	1·19	1·24	1·45	1·35	1·11	0·50	1·45
2×10^8	1·00	1·50	1·37	3·39	2·45	3·02	2·54	2·28	3·39
2×10^8	1·00	0·83	12·20	17·36	8·37	2·72	1·38	0·93	17·36
2×10^8	1·00	1·27	4·64	19·45	9·27	0·96	0·43	0·02	19·45
2×10^8	1·00	0·51	0·48	1·67					1·67
2×10^8	1·00	0·90	1·07	10·46	1·60	1·32	1·35	1·04	10·46
2×10^8	1·00	0·74	0·83	1·60	0·48	0·39	0·61	0·63	1·60
2×10^8	1·00	1·07	4·54	18·70	7·18	2·87	2·12	1·41	18·70
2×10^8	1·00	1·10	65·00	1069·80	357·60	22·55	10·03	5·38	1069·80
2×10^8	1·00	0·95	1·97	26·80	12·35	7·02	3·29	1·33	26·80
2×10^8	1·00	1·76	1·41	9·65	2·21		1·53	1·31	9·65
2×10^8	1·00	0·92	1·57	1·08	1·78	1·39	2·38	2·80	2·80

	RAU		
	D1	D39	
V. cholerae	0·00502	3·13893	
	0·00360	0·04287	
	0·00368	0·00345	
	0·00243	0·86786	
	0·00516	0·03164	
	D1	D11	
Vaxchora D11 Challenge	0·00669	0·00869	
	0·01473	0·02058	
	0·01351	0·01881	
	0·00433	0·00399	
	0·00608	0·03761	
	0·01111	0·00684	
	D1	D11	D91
Vaxchora D91 Challenge	0·00087	0·00118	0·00136
	0·00100	0·00434	0·00169
	0·00310	0·02712	0·00328
	0·00211	0·00541	0·00252
	0·00167	0·00550	0·00704
	0·00249	0·00324	0·00271

	RAU fold change			max FC
	D1	D39		
V. cholerae	1·00	625·15		625·15
	1·00	11·92		11·92
	1·00	0·94		0·94
	1·00	356·90		356·90
	1·00	6·14		6·14
	D1	D11		
Vaxchora D11 Challenge	1·00	1·30		1·30
	1·00	1·40		1·40
	1·00	1·39		1·39
	1·00	0·92		0·92
	1·00	6·19		6·19
	1·00	0·62		0·62
	D1	D11	D91	
Vaxchora D91 Challenge	1·00	1·35	1·56	1·56
	1·00	4·36	1·69	4·36
	1·00	8·76	1·06	8·76
	1·00	2·57	1·19	2·57
	1·00	3·30	4·23	4·23
	1·00	1·30	1·09	1·30

Dose-Escalation Module

CT-B IgM (RAU)									
**Dose (CFU)**	**D1**	**D4**	**D7**	**D15**	**D29**	**D57**	**D85**	**D180**	**max**
10^{5}	4·87681	3·98867	4·93276	3·55923	3·37757	3·15007	2·73105	3·19335	4·93276
10^{5}	0·43177	0·42725	0·48235	0·48335	0·88920	0·65285	0·85346		0·88920
10^{5}	0·95358	0·88401	1·08047	0·94074	0·94989	0·88229	0·95727		1·08047
10^{6}	0·55944	0·51347	0·52430	0·47094	0·50782	0·61324	0·54690		0·61324
10^{6}	4·16655	5·64419	6·28863	4·18381	2·92414	4·17635	3·66352	3·84355	6·28863
10^{6}	1·60650	1·37872	1·87657	1·53953	1·58055	1·47220	1·89365	0·88397	1·89365
10^{7}	3·91511	3·55127	4·55692	4·81872	4·08617	4·97729	6·30023	2·50933	6·30023
10^{7}	0·53346	0·56100	0·48418	0·51347	0·64255	0·56917	0·53980	0·46634	0·64255
10^{7}	0·82430	0·88641	0·66112	0·80020	0·73857	0·76316	0·81349	0·43412	0·88641
10^{8}	0·67071	0·59575	0·47675	0·40417	0·43163	0·48582	0·50863	0·41777	0·67071
10^{8}	0·90076	0·82534	0·84373	0·93846	0·82047	0·92457	0·35076	0·37795	0·93846
10^{8}	0·96409	0·97854	1·18589	1·01366	0·98659	0·98469	0·96533	1·60359	1·60359
10^{9}	0·63882	0·59345	0·67990	0·54847	0·54296	0·31768	0·32137	0·40957	0·67990
10^{9}	0·65520	0·60337	0·49401	0·43706	0·63251	0·45692	0·97566		0·97566
10^{9}	0·57821	0·63789	0·70435	0·69148	0·63504	0·67480	0·76381	0·68802	0·76381
10^{10}	0·39379	0·45920	0·56449	0·72536	0·65116		0·48836	0·56129	0·72536
10^{10}	0·87717	1·06467	0·70027	0·72599			0·67480	0·73569	1·06467
10^{10}	1·07689	1·01323	1·30508	2·43296	1·14982	0·85427	0·94011	0·80897	2·43296

Fold change CT-B IgM									
**Dose (CFU)**	**D1**	**D4**	**D7**	**D15**	**D29**	**D57**	**D85**	**D180**	**max FC**
10^{5}	1·00	0·82	1·01	0·73	0·69	0·65	0·56	0·65	1·01
10^{5}	1·00	0·99	1·12	1·12	2·06	1·51	1·98		2·06
10^{5}	1·00	0·93	1·13	0·99	1·00	0·93	1·00		1·13
10^{6}	1·00	0·92	0·94	0·84	0·91	1·10	0·98		1·10
10^{6}	1·00	1·35	1·51	1·00	0·70	1·00	0·88	0·92	1·51
10^{6}	1·00	0·86	1·17	0·96	0·98	0·92	1·18	0·55	1·18
10^{7}	1·00	0·91	1·16	1·23	1·04	1·27	1·61	0·64	1·61
10^{7}	1·00	1·05	0·91	0·96	1·20	1·07	1·01	0·87	1·20
10^{7}	1·00	1·08	0·80	0·97	0·90	0·93	0·99	0·53	1·08
10^{8}	1·00	0·89	0·71	0·60	0·64	0·72	0·76	0·62	0·89
10^{8}	1·00	0·92	0·94	1·04	0·91	1·03	0·39	0·42	1·04
10^{8}	1·00	1·01	1·23	1·05	1·02	1·02	1·00	1·66	1·66
10^{9}	1·00	0·93	1·06	0·86	0·85	0·50	0·50	0·64	1·06
10^{9}	1·00	0·92	0·75	0·67	0·97	0·70	1·49		1·49
10^{9}	1·00	1·10	1·22	1·20	1·10	1·17	1·32	1·19	1·32
10^{10}	1·00	1·17	1·43	1·84	1·65		1·24	1·43	1·84
10^{10}	1·00	1·21	0·80	0·83			0·77	0·84	1·21
10^{10}	1·00	0·94	1·21	2·26	1·07	0·79	0·87	0·75	2·26

Dose-Expansion Module

CT-B IgM (RAU)									
**Dose (CFU)**	**D1**	**D4**	**D7**	**D15**	**D29**	**D57**	**D85**	**D180**	**max**
0	0·19038	0·20065	0·22448	0·19413	0·22285	0·23885	2·11801	0·41024	2·11801
0	0·16485	0·18299	0·18140	0·19127	0·26778	0·19566	0·14712	0·14018	0·26778
0	0·43432	0·46302	0·49423	0·44139	0·43243	0·34401	0·27802	0·71160	0·71160
0	3·72078	4·16211	4·44041	3·79207	3·69638	8·32086	6·97915	6·87031	8·32086
0	0·32106	0·28629	0·36935	0·32758	0·34934	0·63596	0·60827	0·69474	0·69474
0	0·55765	0·56905	0·58598	0·79676	0·46655	0·79362	0·74938	0·84349	0·84349
0	1·43808	1·29317	1·60618	1·19490	1·74674	1·41165	1·15488	1·68740	1·74674
2×10^{7}	2·40857	2·41834	2·14040	2·30546	2·25363	1·87744	1·95633	2·47112	2·47112
2×10^{7}	0·56271	0·55908	0·56401	0·43040	0·41883	0·44226	0·58814	0·43649	0·58814
2×10^{7}	0·61090	0·66454	0·56103	0·58151	0·54235	0·50607	0·52953	0·49259	0·66454
2×10^{7}	1·25464	1·95569	1·60379	1·48629	1·29106	1·50548	1·20859	1·32007	1·95569
2×10^{7}	0·98241	0·93126	1·13472	1·22269	1·04226	0·84987	0·92273	0·81421	1·22269
2×10^{7}	0·36131	0·34433	0·33486	0·31904	0·33057	0·30901	0·33617	0·95358	0·95358
2×10^{7}	0·90962		0·84625	0·81234	0·81771	0·93977	1·80794		1·80794
2×10^{7}	0·32106	0·35431	0·35207	1·09079	0·79098	1·13101	0·77351	4·15786	4·15786
2×10^{7}	0·32641	0·37977	0·35367	0·33007	0·33519	0·60213	0·46752	0·48941	0·60213
2×10^{7}	1·36033	1·52247	1·77578	1·39511	2·16282	1·97118	2·06870	2·61957	2·61957
2×10^{7}	1·19490	1·78840	1·88798	1·29802	1·48129	1·36263	1·34999	1·21080	1·88798
2×10^{7}	3·94900	4·01532	5·53359	3·89665	4·04886	4·35495	4·75105	4·62821	5·53359
2×10^{7}	6·44998	7·06733	7·67717	7·03415	7·04519	7·11180	6·96820	7·17896	7·67717
2×10^{7}	0·68508	0·53157	0·75534	0·65812	0·76136	0·65680		0·53596	0·76136
2×10^{8}	0·47533	0·47087	0·48929	0·68490	0·45766	0·42280	0·47129	0·44082	0·68490
2×10^{8}	8·25645	8·41662	9·33131	9·59979	8·32462	8·82217	8·30182	6·05049	9·59979
2×10^{8}	0·71517	0·71885	0·72182	0·75208	0·65330	0·61216	0·79833	0·75534	0·79833
2×10^{8}	1·17598	1·20657	1·07723	0·64975	0·75173	0·90996	0·88401	0·65548	1·20657
2×10^{8}	1·83957	2·02094	2·04380	1·30529	1·49143	2·07239	2·45568	2·59235	2·59235
2×10^{8}	0·71030	0·68610	0·69043	0·65893					0·71030
2×10^{8}	0·96334	1·18608	1·06117	2·37578	2·77874	1·52005	1·67208	1·18590	2·77874
2×10^{8}	0·27511	0·24392	0·25143	0·22992	0·30127	0·33581	0·46460	0·38846	0·46460
2×10^{8}	0·51993	0·46006	0·48530	0·38354	0·49144	0·42367	0·41658	0·45310	0·51993
2×10^{8}	0·32580	0·38272	0·31469	0·27476	0·22739	0·24538	0·26581	0·24868	0·38272
2×10^{8}	1·25256	1·56546	1·62095	1·70904	1·58279	1·57410	1·63285	1·69356	1·70904
2×10^{8}	0·39849	0·35973	0·27174	0·26817	0·27476		0·26172	0·39680	0·39849
2×10^{8}	0·26876	0·24429	0·34909	0·33292	0·39596	0·34984	0·32934	0·39012	0·39596

Fold change CT-B IgM									
**Dose (CFU)**	**D1**	**D4**	**D7**	**D15**	**D29**	**D57**	**D85**	**D180**	**max FC**
0	1·00	1·05	1·18	1·02	1·17	1·25	11·13	2·15	11·13
0	1·00	1·11	1·10	1·16	1·62	1·19	0·89	0·85	1·62
0	1·00	1·07	1·14	1·02	1·00	0·79	0·64	1·64	1·64
0	1·00	1·12	1·19	1·02	0·99	2·24	1·88	1·85	2·24
0	1·00	0·89	1·15	1·02	1·09	1·98	1·89	2·16	2·16
0	1·00	1·02	1·05	1·43	0·84	1·42	1·34	1·51	1·51
0	1·00	0·90	1·12	0·83	1·21	0·98	0·80	1·17	1·21
2×10^{7}	1·00	1·00	0·89	0·96	0·94	0·78	0·81	1·03	1·03
2×10^{7}	1·00	0·99	1·00	0·76	0·74	0·79	1·05	0·78	1·05
2×10^{7}	1·00	1·09	0·92	0·95	0·89	0·83	0·87	0·81	1·09
2×10^{7}	1·00	1·56	1·28	1·18	1·03	1·20	0·96	1·05	1·56
2×10^{7}	1·00	0·95	1·16	1·24	1·06	0·87	0·94	0·83	1·24
2×10^{7}	1·00	0·95	0·93	0·88	0·91	0·86	0·93	2·64	2·64
2×10^{7}	1·00		0·93	0·89	0·90	1·03	1·99		1·99
2×10^{7}	1·00	1·10	1·10	3·40	2·46	3·52	2·41	12·95	12·95
2×10^{7}	1·00	1·16	1·08	1·01	1·03	1·84	1·43	1·50	1·84
2×10^{7}	1·00	1·12	1·31	1·03	1·59	1·45	1·52	1·93	1·93
2×10^{7}	1·00	1·50	1·58	1·09	1·24	1·14	1·13	1·01	1·58
2×10^{7}	1·00	1·02	1·40	0·99	1·03	1·10	1·20	1·17	1·40
2×10^{7}	1·00	1·10	1·19	1·09	1·09	1·10	1·08	1·11	1·19
2×10^{7}	1·00	0·78	1·10	0·96	1·11	0·96		0·78	1·11
2×10^{8}	1·00	0·99	1·03	1·44	0·96	0·89	0·99	0·93	1·44
2×10^{8}	1·00	1·02	1·13	1·16	1·01	1·07	1·01	0·73	1·16
2×10^{8}	1·00	1·01	1·01	1·05	0·91	0·86	1·12	1·06	1·12
2×10^{8}	1·00	1·03	0·92	0·55	0·64	0·77	0·75	0·56	1·03
2×10^{8}	1·00	1·10	1·11	0·71	0·81	1·13	1·33	1·41	1·41
2×10^{8}	1·00	0·97	0·97	0·93					0·97
2×10^{8}	1·00	1·23	1·10	2·47	2·88	1·58	1·74	1·23	2·88
2×10^{8}	1·00	0·89	0·91	0·84	1·10	1·22	1·69	1·41	1·69
2×10^{8}	1·00	0·88	0·93	0·74	0·95	0·81	0·80	0·87	0·95
2×10^{8}	1·00	1·17	0·97	0·84	0·70	0·75	0·82	0·76	1·17
2×10^{8}	1·00	1·25	1·29	1·36	1·26	1·26	1·30	1·35	1·36
2×10^{8}	1·00	0·90	0·68	0·67	0·69		0·66	1·00	1·00
2×10^{8}	1·00	0·91	1·30	1·24	1·47	1·30	1·23	1·45	1·47

	RAU		
	D1	D39	
V. cholerae	0·94893	12·85977	
	1·61163	28·32030	
	0·55553	2·45622	
	0·69451	0·64255	
	0·46343	6·29635	
	D1	D11	
Vaxchora D11 Challenge	0·30816	0·38228	
	2·66614	2·81467	
	0·42775	4·44870	
	0·58424	0·53346	
	0·60566	0·63096	
	0·71375	0·54690	
	D1	D11	D91
Vaxchora D91 Challenge	0·44531	0·39351	0·39217
	5·53714	2·54225	1·49817
	0·18829	0·19988	0·19747
	2·98887	2·10560	4·74493
	0·58809	1·24505	0·59575
	0·58732	0·58885	0·51267

	RAU fold change			max FC
	D1	D39		
V. cholerae	1·00	13·55		13·55
	1·00	17·57		17·57
	1·00	4·42		4·42
	1·00	0·93		0·93
	1·00	13·59		13·59
	D1	D11		
Vaxchora D11 Challenge	1·00	1·24		1·24
	1·00	1·06		1·06
	1·00	10·40		10·40
	1·00	0·91		0·91
	1·00	1·04		1·04
	1·00	0·77		0·77
	D1	D11	D91	
Vaxchora D91 Challenge	1·00	0·88	0·88	0·88
	1·00	0·46	0·27	0·46
	1·00	1·06	1·05	1·06
	1·00	0·70	1·59	1·59
	1·00	2·12	1·01	2·12
	1·00	1·00	0·87	1·00

Dose-Escalation Module

CT-B IgG (RAU)									
**Dose (CFU)**	**D1**	**D4**	**D7**	**D15**	**D29**	**D57**	**D85**	**D180**	**max**
10^{5}	0·01851	0·01728	0·01877	0·07514	0·07877	0·05807	0·05334	0·03417	0·07877
10^{5}	0·00809	0·00862	0·01033	0·01042	0·01077	0·01244	0·01463		0·01463
10^{5}	0·01009	0·00964	0·00995	0·01234	0·02839	0·02199	0·02409		0·02839
10^{6}	0·02487	0·02378	0·02954	0·25102	0·13203	0·09238	0·08265		0·25102
10^{6}	0·02036	0·02579	0·03829	0·20417	0·11996	0·07454	0·04340	0·03820	0·20417
10^{6}	0·07032	0·06544	0·08750	0·77320	0·50655	0·37722	0·23921	0·12175	0·77320
10^{7}	0·04688	0·04671	0·04381	0·05281	0·05975	0·07482	0·05981	0·01927	0·07482
10^{7}	0·03965	0·04883	0·04498	0·04878	0·05913	0·06205	0·05586	0·01227	0·06205
10^{7}	0·01414	0·01231	0·01391	0·01863	0·01916	0·01672	0·01541	0·00677	0·01916
10^{8}	0·01487	0·01438	0·01523	0·04715	0·07052	0·05336	0·07113	0·04438	0·07113
10^{8}	0·02166	0·02025	0·02221	0·10638	0·10757	0·10950	0·05941	0·04704	0·10950
10^{8}	0·01288	0·00918	0·02078	0·26963	0·28151	0·10140	0·08876	0·06960	0·28151
10^{9}	0·14131	0·14954	0·31488	0·98135	0·67288	0·32378	0·29582	0·31486	0·98135
10^{9}	0·00244	0·00321	0·00342	0·00373	0·00420	0·00396	0·00535		0·00535
10^{9}	0·00185	0·00201	0·00216	0·03092	0·01869	0·01736	0·01622	0·00960	0·03092
10^{10}	0·00565	0·00616	0·00845	0·01218	0·01058		0·01011	0·01718	0·01718
10^{10}	0·04259	0·04580	0·04714	0·22594			0·09633	0·07051	0·22594
10^{10}	0·01006	0·01022	0·02122	1·67337	1·23423	0·82762	0·49335	0·26583	1·67337

Fold change CT-B IgG									
**Dose (CFU)**	**D1**	**D4**	**D7**	**D15**	**D29**	**D57**	**D85**	**D180**	**max FC**
10^{5}	1·00	0·93	1·01	4·06	4·26	3·14	2·88	1·85	4·26
10^{5}	1·00	1·07	1·28	1·29	1·33	1·54	1·81		1·81
10^{5}	1·00	0·95	0·99	1·22	2·81	2·18	2·39		2·81
10^{6}	1·00	0·96	1·19	10·09	5·31	3·71	3·32		10·09
10^{6}	1·00	1·27	1·88	10·03	5·89	3·66	2·13	1·88	10·03
10^{6}	1·00	0·93	1·24	11·00	7·20	5·36	3·40	1·73	11·00
10^{7}	1·00	1·00	0·93	1·13	1·27	1·60	1·28	0·41	1·60
10^{7}	1·00	1·23	1·13	1·23	1·49	1·56	1·41	0·31	1·56
10^{7}	1·00	0·87	0·98	1·32	1·36	1·18	1·09	0·48	1·36
10^{8}	1·00	0·97	1·02	3·17	4·74	3·59	4·78	2·98	4·78
10^{8}	1·00	0·93	1·03	4·91	4·97	5·05	2·74	2·17	5·05
10^{8}	1·00	0·71	1·61	20·93	21·85	7·87	6·89	5·40	21·85
10^{9}	1·00	1·06	2·23	6·94	4·76	2·29	2·09	2·23	6·94
10^{9}	1·00	1·31	1·40	1·53	1·72	1·62	2·19		2·19
10^{9}	1·00	1·08	1·17	16·71	10·10	9·38	8·76	5·19	16·71
10^{10}	1·00	1·09	1·50	2·16	1·87		1·79	3·04	3·04
10^{10}	1·00	1·08	1·11	5·30			2·26	1·66	5·30
10^{10}	1·00	1·02	2·11	166·32	122·68	82·26	49·04	26·42	166·32

Dose-Expansion Module

CT-B IgG (RAU)									
**Dose (CFU)**	**D1**	**D4**	**D7**	**D15**	**D29**	**D57**	**D85**	**D180**	**max**
0	0·00207	0·00221	0·00238	0·00219	0·00268	0·00220	0·00240	0·00253	0·00268
0	0·02358	0·02653	0·02537	0·02193	0·02353	0·02238	0·04874	0·03261	0·04874
0	0·01165	0·01143	0·01239	0·00971	0·01031	0·01504	0·01085	0·00127	0·01504
0	0·04453	0·04851	0·04485	0·04042	0·04928	0·02718	0·02181	0·02017	0·04928
0	0·00489	0·00454	0·00481	0·00486	0·00481	0·00394	0·00361	0·00318	0·00489
0	0·03057	0·03305	0·03172	0·02102	0·01737	0·02287	0·02015	0·02014	0·03305
0	0·00360	0·00292	0·00326	0·00344	0·00366	0·00279	0·00271	0·00319	0·00366
2×10^{7}	0·02704	0·02340	0·02596	0·09392	0·09417	0·07905	0·08374	0·07531	0·09417
2×10^{7}	0·01626	0·01855	0·02626	0·27783	0·17611	0·12094	0·08473	0·04446	0·27783
2×10^{7}	0·00162	0·00182	0·00147	0·00181	0·00168	0·00212	0·00217	0·00151	0·00217
2×10^{7}	0·02510	0·03196	0·02839	0·05873	0·05390	0·06067	0·03411	0·02853	0·06067
2×10^{7}	0·01200	0·01141	0·01027	0·03015	0·03656	0·03686	0·03377	0·01353	0·03686
2×10^{7}	0·01369	0·01239	0·01415	0·03064	0·03670	0·03559	0·03775	0·01573	0·03775
2×10^{7}	0·02377		0·02680	0·02603	0·03696	0·04059	0·03165		0·04059
2×10^{7}	0·02336	0·02500	0·03214	0·80040	0·64070	0·33133	0·12467	0·04366	0·80040
2×10^{7}	0·00452	0·00457	0·00434	0·00481	0·00755	0·00584	0·00480	0·00463	0·00755
2×10^{7}	0·03861	0·03261	0·04124	0·03529	0·02190	0·02078	0·01894	0·01526	0·04124
2×10^{7}	0·02151	0·02047	0·05198	0·24724	0·31328	0·14386	0·10090	0·09747	0·31328
2×10^{7}	0·01236	0·01224	0·01628	0·03996	0·04890	0·03307	0·03532	0·02204	0·04890
2×10^{7}	0·00412	0·00526	0·00512	0·00488	0·00577	0·00539	0·00516	0·00577	0·00577
2×10^{7}	0·00249	0·00240	0·00943	0·02416	0·02546	0·01883		0·00766	0·02546
2×10^{8}	0·03399	0·03559	0·04152	0·04572	0·06718	0·07432	0·07926	0·05454	0·07926
2×10^{8}	0·00527	0·00523	0·00591	0·03493	0·02960	0·02615	0·01986	0·01038	0·03493
2×10^{8}	0·03011	0·03274	0·05401	0·08919	0·06974	0·05313	0·02221	0·02277	0·08919
2×10^{8}	0·02948	0·02736	0·04662	0·34480	0·32304	0·17476	0·09586	0·03630	0·34480
2×10^{8}	0·04245	0·04202	0·14819	1·45274	1·04192	0·18872	0·10869	0·04679	1·45274
2×10^{8}	0·02681	0·02411	0·04389	0·09189					0·09189
2×10^{8}	0·06990	0·08113	0·11032	3·68765	3·89655	3·46795	1·76769	0·19164	3·89655
2×10^{8}	0·00700	0·00573	0·00662	0·01361	0·02172	0·02038	0·02489	0·01916	0·02489
2×10^{8}	0·00327	0·00243	0·00240	0·00396	0·01132	0·01535	0·01436	0·01348	0·01535
2×10^{8}	0·01108	0·01329	0·03141	0·32014	0·18031	0·04703	0·02616	0·01984	0·32014
2×10^{8}	0·02612	0·02495	0·02437	0·02646	0·02605	0·02694	0·02644	0·02891	0·02891
2×10^{8}	0·00527	0·00486	0·00443	0·00367	0·00394		0·00462	0·01069	0·01069
2×10^{8}	0·01031	0·00854	0·01343	0·02188	0·02080	0·01747	0·01543	0·01597	0·02188

Fold change CT-B IgG									
**Dose (CFU)**	**D1**	**D4**	**D7**	**D15**	**D29**	**D57**	**D85**	**D180**	**max FC**
0	1·00	1·07	1·15	1·06	1·30	1·06	1·16	1·22	1·30
0	1·00	1·12	1·08	0·93	1·00	0·95	2·07	1·38	2·07
0	1·00	0·98	1·06	0·83	0·89	1·29	0·93	0·11	1·29
0	1·00	1·09	1·01	0·91	1·11	0·61	0·49	0·45	1·11
0	1·00	0·93	0·98	0·99	0·98	0·81	0·74	0·65	0·99
0	1·00	1·08	1·04	0·69	0·57	0·75	0·66	0·66	1·08
0	1·00	0·81	0·91	0·96	1·02	0·78	0·75	0·89	1·02
2×10^{7}	1·00	0·87	0·96	3·47	3·48	2·92	3·10	2·79	3·48
2×10^{7}	1·00	1·14	1·62	17·09	10·83	7·44	5·21	2·73	17·09
2×10^{7}	1·00	1·12	0·91	1·12	1·04	1·31	1·34	0·93	1·34
2×10^{7}	1·00	1·27	1·13	2·34	2·15	2·42	1·36	1·14	2·42
2×10^{7}	1·00	0·95	0·86	2·51	3·05	3·07	2·81	1·13	3·07
2×10^{7}	1·00	0·91	1·03	2·24	2·68	2·60	2·76	1·15	2·76
2×10^{7}	1·00		1·13	1·10	1·56	1·71	1·33		1·71
2×10^{7}	1·00	1·07	1·38	34·26	27·43	14·18	5·34	1·87	34·26
2×10^{7}	1·00	1·01	0·96	1·06	1·67	1·29	1·06	1·02	1·67
2×10^{7}	1·00	0·84	1·07	0·91	0·57	0·54	0·49	0·40	1·07
2×10^{7}	1·00	0·95	2·42	11·49	14·56	6·69	4·69	4·53	14·56
2×10^{7}	1·00	0·99	1·32	3·23	3·96	2·68	2·86	1·78	3·96
2×10^{7}	1·00	1·28	1·24	1·18	1·40	1·31	1·25	1·40	1·40
2×10^{7}	1·00	0·96	3·79	9·70	10·22	7·56		3·07	10·22
2×10^{8}	1·00	1·05	1·22	1·34	1·98	2·19	2·33	1·60	2·33
2×10^{8}	1·00	0·99	1·12	6·63	5·62	4·96	3·77	1·97	6·63
2×10^{8}	1·00	1·09	1·79	2·96	2·32	1·76	0·74	0·76	2·96
2×10^{8}	1·00	0·93	1·58	11·70	10·96	5·93	3·25	1·23	11·70
2×10^{8}	1·00	0·99	3·49	34·22	24·54	4·45	2·56	1·10	34·22
2×10^{8}	1·00	0·90	1·64	3·43					3·43
2×10^{8}	1·00	1·16	1·58	52·75	55·74	49·61	25·29	2·74	55·74
2×10^{8}	1·00	0·82	0·95	1·95	3·10	2·91	3·56	2·74	3·56
2×10^{8}	1·00	0·74	0·73	1·21	3·46	4·69	4·39	4·12	4·69
2×10^{8}	1·00	1·20	2·83	28·89	16·27	4·24	2·36	1·79	28·89
2×10^{8}	1·00	0·95	0·93	1·01	1·00	1·03	1·01	1·11	1·11
2×10^{8}	1·00	0·92	0·84	0·70	0·75		0·88	2·03	2·03
2×10^{8}	1·00	0·83	1·30	2·12	2·02	1·69	1·50	1·55	2·12

	RAU		
	D1	D39	
V. cholerae	0·02551	4·13854	
	0·19926	5·01770	
	0·36006	8·30713	
	0·01204	0·59248	
	0·04858	3·11167	
	D1	D11	
Vaxchora D11 Challenge	0·01724	0·14968	
	0·02182	0·02640	
	0·13189	2·31666	
	0·03618	0·03522	
	0·01503	0·01580	
	0·12351	0·09401	
	D1	D11	D91
Vaxchora D91 Challenge	0·19207	0·26394	0·22632
	0·00626	0·00805	0·03951
	0·02442	0·14890	0·08181
	0·02854	0·03099	0·14233
	0·00554	0·78103	0·10327
	0·00950	0·03171	0·02646

	RAU fold change			max FC
	D1	D39		
V. cholerae	1·00	162·24		162·24
	1·00	25·18		25·18
	1·00	23·07		23·07
	1·00	49·21		49·21
	1·00	64·06		64·06
	D1	D11		
Vaxchora D11 Challenge	1·00	8·68		8·68
	1·00	1·21		1·21
	1·00	17·56		17·56
	1·00	0·97		0·97
	1·00	1·05		1·05
	1·00	0·76		0·76
	D1	D11	D91	
Vaxchora D91 Challenge	1·00	1·37	1·18	1·37
	1·00	1·29	6·31	6·31
	1·00	6·10	3·35	6·10
	1·00	1·09	4·99	4·99
	1·00	140·90	18·63	140·90
	1·00	3·34	2·78	3·34

Dose-Escalation Module

CT-B IgA (RAU)									
Dose (CFU)	D1	D4	D7	D15	D29	D57	D85	D180	max
10^5	0·03726	0·03573	0·03412	0·25297	0·21222	0·11656	0·08222	0·05404	0·25297
10^5	0·02759	0·02602	0·02710	0·02134	0·02405	0·02090	0·02694		0·02759
10^5	0·01653	0·01839	0·01947	0·02181	0·03444	0·02170	0·01917		0·03444
10^6	0·01793	0·02019	0·02462	0·14832	0·04823	0·03117	0·02598		0·14832
10^6	0·08621	0·06931	0·18598	0·59258	0·16387	0·08949	0·08354	0·07132	0·59258
10^6	0·05409	0·07933	0·09187	0·54065	0·11732	0·08498	0·05125	0·05972	0·54065
10^7	0·02508	0·02357	0·02379	0·05625	0·06848	0·06282	0·06772	0·03690	0·06848
10^7	0·01069	0·00967	0·01018	0·01395	0·03223	0·02103	0·01928	0·01389	0·03223
10^7	0·00797	0·00665	0·00703	0·00983	0·00887	0·00726	0·00766	0·00437	0·00983
10^8	0·06446	0·07413	0·06444	1·60204	1·05840	0·51290	0·57179	0·44060	1·60204
10^8	0·01603	0·01373	0·01451	0·05641	0·05567	0·02918	0·01614	0·01586	0·05641
10^8	0·00869	0·00785	0·06308	0·31965	0·38580	0·10644	0·09288	0·08963	0·38580
10^9	0·03562	0·03343	0·16580	0·23682	0·09774	0·05806	0·04668	0·04780	0·23682
10^9	0·02926	0·02401	0·02452	0·01565	0·01601	0·01327	0·02276		0·02926
10^9	0·01338	0·01383	0·01279	0·36413	0·20362	0·16801	0·19280	0·09348	0·36413
10^{10}	0·01308	0·01225	0·01451	0·01802	0·01769		0·01501	0·01663	0·01802
10^{10}	0·03379	0·03811	0·08379	0·29181			0·06284	0·05550	0·29181
10^{10}	0·00146	0·00158	0·00614	0·12045	0·03742	0·02164	0·01506	0·01038	0·12045

Fold change CT-B IgA									
Dose (CFU)	D1	D4	D7	D15	D29	D57	D85	D180	max FC
10^5	1·00	0·96	0·92	6·79	5·70	3·13	2·21	1·45	6·79
10^5	1·00	0·94	0·98	0·77	0·87	0·76	0·98		0·98
10^5	1·00	1·11	1·18	1·32	2·08	1·31	1·16		2·08
10^6	1·00	1·13	1·37	8·27	2·69	1·74	1·45		8·27
10^6	1·00	0·80	2·16	6·87	1·90	1·04	0·97	0·83	6·87
10^6	1·00	1·47	1·70	10·00	2·17	1·57	0·95	1·10	10·00
10^7	1·00	0·94	0·95	2·24	2·73	2·50	2·70	1·47	2·73
10^7	1·00	0·90	0·95	1·31	3·01	1·97	1·80	1·30	3·01
10^7	1·00	0·83	0·88	1·23	1·11	0·91	0·96	0·55	1·23
10^8	1·00	1·15	1·00	24·85	16·42	7·96	8·87	6·83	24·85
10^8	1·00	0·86	0·91	3·52	3·47	1·82	1·01	0·99	3·52
10^8	1·00	0·90	7·26	36·79	44·41	12·25	10·69	10·32	44·41
10^9	1·00	0·94	4·66	6·65	2·74	1·63	1·31	1·34	6·65
10^9	1·00	0·82	0·84	0·53	0·55	0·45	0·78		0·84
10^9	1·00	1·03	0·96	27·22	15·22	12·56	14·41	6·99	27·22
10^{10}	1·00	0·94	1·11	1·38	1·35		1·15	1·27	1·38
10^{10}	1·00	1·13	2·48	8·64			1·86	1·64	8·64
10^{10}	1·00	1·08	4·21	82·55	25·64	14·83	10·32	7·11	82·55

Dose-Expansion Module

CT-B IgA (RAU)									
Dose (CFU)	D1	D4	D7	D15	D29	D57	D85	D180	max
0	0·03728	0·03966	0·04158	0·04068	0·03949	0·03202	0·03370	0·03271	0·04158
0	0·03116	0·02779	0·02510	0·02603	0·02676	0·02388	0·07611	0·03122	0·07611
0	0·00940	0·01096	0·01058	0·00716	0·00914	0·00889	0·00848	0·00712	0·01096
0	0·06728	0·05666	0·06639	0·05694	0·04127	0·06243	0·04509	0·03932	0·06728
0	0·01500	0·01510	0·02092	0·01716	0·01543	0·02276	0·01799	0·01639	0·02276
0	0·01926	0·01904	0·01540	0·00656	0·00573	0·00667	0·00643	0·02580	0·02580
0	0·00355	0·00270	0·00302	0·00305	0·00421	0·00261	0·00135	0·00264	0·00421
2×10^7	0·01154	0·01039	0·01858	0·06233	0·03020	0·02182	0·01928	0·01875	0·06233
2×10^7	0·01141	0·01226	0·01998	0·09696	0·04559	0·02034	0·01781	0·01269	0·09696
2×10^7	0·01136	0·01118	0·00946	0·00988	0·00876	0·00946	0·01052	0·00868	0·01136
2×10^7	0·04119	0·04771	0·04350	0·05776	0·06048	0·06290	0·05024	0·02629	0·06290
2×10^7	0·03069	0·02525	0·02950	0·07328	0·05152	0·03268	0·02505	0·00800	0·07328
2×10^7	0·07462	0·08355	0·11789	0·17153	0·19611	0·14536	0·11870	0·04946	0·19611
2×10^7	0·31450		0·24463	0·19511	0·24246	0·23152	0·29074		0·31450
2×10^7	0·02049	0·02349	0·06504	0·83940	0·35016	0·11330	0·07455	0·04749	0·83940
2×10^7	0·01568	0·01670	0·01982	0·02319	0·02881	0·01776	0·01876	0·02153	0·02881
2×10^7	0·01900	0·01703	0·02023	0·02132	0·01562	0·01211	0·01049	0·01087	0·02132
2×10^7	0·02452	0·01704	0·05261	0·07290	0·08199	0·05833	0·05292	0·05925	0·08199
2×10^7	0·02642	0·02891	0·05162	0·17751	0·10531	0·05679	0·07933	0·05750	0·17751
2×10^7	0·00909	0·00769	0·01418	0·01846	0·01701	0·01403	0·01116	0·00972	0·01846
2×10^7	0·01108	0·01105	0·01421	0·01156	0·01217	0·01351		0·00806	0·01421
2×10^8	0·04062	0·03961	0·03912	0·04350	0·05119	0·04048	0·03949	0·04537	0·05119
2×10^8	0·03935	0·05003	0·04594	0·18997	0·11156	0·07056	0·05847	0·03994	0·18997
2×10^8	0·02841	0·02746	0·05777	0·07595	0·04710	0·03310	0·01541	0·02079	0·07595
2×10^8	0·02052	0·01901	0·09017	0·29997	0·20807	0·09570	0·05812	0·02518	0·29997
2×10^8	0·01871	0·02304	0·10560	0·17897	0·09562	0·02732	0·01817	0·03731	0·17897
2×10^8	0·02684	0·02030	0·03895	0·04358					0·04358
2×10^8	0·01314	0·01533	0·05163	0·34261	0·05408	0·03038	0·02163	0·01358	0·34261
2×10^8	0·01947	0·01907	0·01785	0·02697	0·02964	0·02451	0·02574	0·02815	0·02964
2×10^8	0·05973	0·06631	0·05716	0·03010	0·11680	0·07038	0·05513	0·03865	0·11680
2×10^8	0·01867	0·02081	0·29560	1·47954	0·42018	0·04436	0·03394	0·02601	1·47954
2×10^8	0·02455	0·02626	0·02397	0·02603	0·02124	0·02594	0·02133	0·02298	0·02626
2×10^8	0·03270	0·03223	0·02473	0·01782	0·02273		0·02230	0·03109	0·03270
2×10^8	0·00478	0·00356	0·00594	0·00763	0·00711	0·00727	0·00640	0·00978	0·00978

Fold change CT-B IgA									
Dose (CFU)	D1	D4	D7	D15	D29	D57	D85	D180	max FC
0	1·00	1·06	1·12	1·09	1·06	0·86	0·90	0·88	1·12
0	1·00	0·89	0·81	0·84	0·86	0·77	2·44	1·00	2·44
0	1·00	1·17	1·13	0·76	0·97	0·95	0·90	0·76	1·17
0	1·00	0·84	0·99	0·85	0·61	0·93	0·67	0·58	0·99
0	1·00	1·01	1·40	1·14	1·03	1·52	1·20	1·09	1·52
0	1·00	0·99	0·80	0·34	0·30	0·35	0·33	1·34	1·34
0	1·00	0·76	0·85	0·86	1·18	0·73	0·38	0·74	1·18
2×10^7	1·00	0·90	1·61	5·40	2·62	1·89	1·67	1·63	5·40
2×10^7	1·00	1·07	1·75	8·50	4·00	1·78	1·56	1·11	8·50
2×10^7	1·00	0·98	0·83	0·87	0·77	0·83	0·93	0·76	0·98
2×10^7	1·00	1·16	1·06	1·40	1·47	1·53	1·22	0·64	1·53
2×10^7	1·00	0·82	0·96	2·39	1·68	1·06	0·82	0·26	2·39
2×10^7	1·00	1·12	1·58	2·30	2·63	1·95	1·59	0·66	2·63
2×10^7	1·00		0·78	0·62	0·77	0·74	0·92		0·92
2×10^7	1·00	1·15	3·17	40·97	17·09	5·53	3·64	2·32	40·97
2×10^7	1·00	1·07	1·26	1·48	1·84	1·13	1·20	1·37	1·84
2×10^7	1·00	0·90	1·06	1·12	0·82	0·64	0·55	0·57	1·12
2×10^7	1·00	0·70	2·15	2·97	3·34	2·38	2·16	2·42	3·34
2×10^7	1·00	1·09	1·95	6·72	3·99	2·15	3·00	2·18	6·72
2×10^7	1·00	0·85	1·56	2·03	1·87	1·54	1·23	1·07	2·03
2×10^7	1·00	1·00	1·28	1·04	1·10	1·22		0·73	1·28
2×10^8	1·00	0·98	0·96	1·07	1·26	1·00	0·97	1·12	1·26
2×10^8	1·00	1·27	1·17	4·83	2·84	1·79	1·49	1·01	4·83
2×10^8	1·00	0·97	2·03	2·67	1·66	1·17	0·54	0·73	2·67
2×10^8	1·00	0·93	4·40	14·62	10·14	4·66	2·83	1·23	14·62
2×10^8	1·00	1·23	5·65	9·57	5·11	1·46	0·97	1·99	9·57
2×10^8	1·00	0·76	1·45	1·62					1·62
2×10^8	1·00	1·17	3·93	26·07	4·12	2·31	1·65	1·03	26·07
2×10^8	1·00	0·98	0·92	1·38	1·52	1·26	1·32	1·45	1·52
2×10^8	1·00	1·11	0·96	0·50	1·96	1·18	0·92	0·65	1·96
2×10^8	1·00	1·11	15·83	79·23	22·50	2·38	1·82	1·39	79·23
2×10^8	1·00	1·07	0·98	1·06	0·87	1·06	0·87	0·94	1·07
2×10^8	1·00	0·99	0·76	0·55	0·70		0·68	0·95	0·99
2×10^8	1·00	0·74	1·24	1·59	1·49	1·52	1·34	2·04	2·04

	RAU		
	D1	D39	
V. cholerae	0·04752	16·53919	
	0·04201	2·55035	
	0·10947	2·80699	
	0·01965	0·23643	
	0·07002	2·84187	
	D1	D11	
Vaxchora D11 Challenge	0·03642	0·11842	
	0·08355	0·09830	
	0·10063	0·59184	
	0·03685	0·04190	
	0·03632	0·03457	
	0·05450	0·02240	
	D1	D11	D91
Vaxchora D91 Challenge	0·60973	0·48230	0·38725
	0·01831	0·02750	0·02698
	0·02542	0·27882	0·04546
	0·02714	0·03339	0·06998
	0·18627	0·90015	0·21024
	0·02352	0·08027	0·02866

	RAU fold change			max FC
	D1	D39		
V. cholerae	1·00	348·05		348·05
	1·00	60·71		60·71
	1·00	25·64		25·64
	1·00	12·03		12·03
	1·00	40·59		40·59
	D1	D11		
Vaxchora D11 Challenge	1·00	3·25		3·25
	1·00	1·18		1·18
	1·00	5·88		5·88
	1·00	1·14		1·14
	1·00	0·95		0·95
	1·00	0·41		0·41
	D1	D11	D91	
Vaxchora D91 Challenge	1·00	0·79	0·64	0·79
	1·00	1·50	1·47	1·50
	1·00	10·97	1·79	10·97
	1·00	1·23	2·58	2·58
	1·00	4·83	1·13	4·83
	1·00	3·41	1·22	3·41

Dose-Escalation Module

TcpA IgM (RAU)									
**Dose (CFU)**	**D1**	**D4**	**D7**	**D15**	**D29**	**D57**	**D85**	**D180**	**max**
10^{5}	0·52587	0·41776	0·59419	0·64290	0·49831	0·58443	0·38355	0·70887	0·70887
10^{5}	0·32086	0·33258	0·39082	0·50454	0·36554	0·23154	0·33398		0·50454
10^{5}	0·39082	0·36478	0·46058	0·79350	0·58322	0·42920	0·40156		0·79350
10^{6}	0·38139	0·37482	0·32251	0·38981	0·40527	0·43650	0·46891		0·46891
10^{6}	1·01334	1·18599	1·42736	1·80176	1·01001	1·13339	1·01667	0·74940	1·80176
10^{6}	0·23649	0·19469	0·32322	0·40306	0·26343	0·22290	0·28312	0·13580	0·40306
10^{7}	0·51107	0·48071	0·61355	0·78613	0·59781	0·62450	0·75928	0·28706	0·78613
10^{7}	0·64042	0·66429	0·45604	0·46209	0·49257	0·54379	2·46632	0·34505	2·46632
10^{7}	0·60748	0·66802	0·51145	0·49949	0·58299	0·82963	0·76187	0·29487	0·82963
10^{8}	0·20165	0·19573	0·13347	0·14026	0·16218	0·17654	0·17934	0·08548	0·20165
10^{8}	0·83273	0·76748	0·77831	1·10424	0·81857	0·78788	0·34066	0·39152	1·10424
10^{8}	0·51920	0·47919	0·89064	1·79204	1·15381	0·40667	0·37725	0·60999	1·79204
10^{9}	0·53166	0·50991	0·66636	0·58020	0·57541	0·18579	0·20096	0·23369	0·66636
10^{9}	0·61314	0·52426	0·31252	0·17204	0·27225	0·18108	0·55545		0·61314
10^{9}	0·35978	0·36459	0·36719	0·39039	0·34249	0·40895	0·42119	0·39378	0·42119
10^{10}	0·25652	0·37800	0·41621	0·53890	0·57135		0·31743	0·34249	0·57135
10^{10}	0·37240	0·48458	0·26105	0·32828			0·40211	0·32429	0·48458
10^{10}	0·20740	0·23989	0·29772	1·47887	0·68902	0·34249	0·71388	0·33300	1·47887

Fold change TcpA IgM									
**Dose (CFU)**	**D1**	**D4**	**D7**	**D15**	**D29**	**D57**	**D85**	**D180**	**max FC**
10^{5}	1·00	0·79	1·13	1·22	0·95	1·11	0·73	1·35	1·35
10^{5}	1·00	1·04	1·22	1·57	1·14	0·72	1·04		1·57
10^{5}	1·00	0·93	1·18	2·03	1·49	1·10	1·03		2·03
10^{6}	1·00	0·98	0·85	1·02	1·06	1·14	1·23		1·23
10^{6}	1·00	1·17	1·41	1·78	1·00	1·12	1·00	0·74	1·78
10^{6}	1·00	0·82	1·37	1·70	1·11	0·94	1·20	0·57	1·70
10^{7}	1·00	0·94	1·20	1·54	1·17	1·22	1·49	0·56	1·54
10^{7}	1·00	1·04	0·71	0·72	0·77	0·85	3·85	0·54	3·85
10^{7}	1·00	1·10	0·84	0·82	0·96	1·37	1·25	0·49	1·37
10^{8}	1·00	0·97	0·66	0·70	0·80	0·88	0·89	0·42	0·97
10^{8}	1·00	0·92	0·93	1·33	0·98	0·95	0·41	0·47	1·33
10^{8}	1·00	0·92	1·72	3·45	2·22	0·78	0·73	1·17	3·45
10^{9}	1·00	0·96	1·25	1·09	1·08	0·35	0·38	0·44	1·25
10^{9}	1·00	0·86	0·51	0·28	0·44	0·30	0·91		0·91
10^{9}	1·00	1·01	1·02	1·09	0·95	1·14	1·17	1·09	1·17
10^{10}	1·00	1·47	1·62	2·10	2·23		1·24	1·34	2·23
10^{10}	1·00	1·30	0·70	0·88			1·08	0·87	1·30
10^{10}	1·00	1·16	1·44	7·13	3·32	1·65	3·44	1·61	7·13

Dose-Expansion Module

TcpA IgM (RAU)									
**Dose (CFU)**	**D1**	**D4**	**D7**	**D15**	**D29**	**D57**	**D85**	**D180**	**max**
0	0·29304	0·30452	0·32079	0·29148	0·34167	0·29956	0·37183	0·43321	0·43321
0	0·34061	0·32790	0·32711	0·29122	0·31895	0·34087	0·20424	0·17372	0·34087
0	0·77390	0·89383	0·90910	0·79886	0·87799	0·59140	0·45195	0·94721	0·94721
0	0·64380	0·72572	0·68331	0·55849	0·52566	9·55528	2·18979	63·97380	63·97380
0	0·71303	0·60041	0·88337	0·74576	0·81902	1·48303	1·31530	0·93920	1·48303
0	0·38286	0·41487	0·44836	0·37016	0·20014	0·35497	0·31749	0·39823	0·44836
0	1·36262	1·14129	1·60493	1·30952	2·12758	2·01420	1·00539	2·21101	2·21101
2×10^{7}	0·87925	0·75399	0·62555	0·77205	0·85561	0·63437	0·65030	0·82525	0·87925
2×10^{7}	0·24685	0·23664	0·30269	2·40990	1·17831	0·43129	0·49516	0·32105	2·40990
2×10^{7}	0·56683	0·67704	0·55964	0·70249	0·56625	0·49039	0·40615	0·43623	0·70249
2×10^{7}	0·34379	0·57779	0·48899	1·21735	0·54961	0·60682	0·47250	0·53576	1·21735
2×10^{7}	0·54446	0·45112	0·75461	2·46897	1·09158	0·47250	0·42854	0·45963	2·46897
2×10^{7}	0·17920	0·17970	0·16750	0·22800	0·29330	0·26121	0·30583	0·32979	0·32979
2×10^{7}	0·77513		0·75308	0·65267	0·59053	0·69139	0·92733		0·92733
2×10^{7}	0·22952	0·24660	0·22749	0·30269	0·28109	0·39082	0·31951	0·30110	0·39082
2×10^{7}	0·44836	0·49713	0·44036	0·42005	0·33160	0·35053	0·21967	0·41001	0·49713
2×10^{7}	1·32562	1·70566	2·27234	2·07857	1·35660	1·30090	1·24778	1·39939	2·27234
2×10^{7}	0·15834	0·22752	0·44003	1·35360	1·28385	0·59666	0·35796	0·26543	1·35360
2×10^{7}	0·48608	0·47219	0·67575	0·67857	0·64557	0·66877	0·64156	0·53687	0·67857
2×10^{7}	0·33894	0·36862	0·68709	0·93325	0·59666	0·39657	0·37405	0·34252	0·93325
2×10^{7}	0·44278	0·30432	0·52262	0·43730	0·46058	0·53798		0·42831	0·53798
2×10^{8}	0·27617	0·28368	0·33160	0·89605	0·40234	0·25530	6·67972	0·39367	6·67972
2×10^{8}	1·15540	1·12724	1·41426	1·68902	1·34503	1·48323	1·11497	0·51660	1·68902
2×10^{8}	0·55276	0·54818	0·63820	0·77113	0·48171	0·81840	0·58201	0·51831	0·81840
2×10^{8}	1·58581	1·72279	1·58841	0·86662	1·14503	1·16470	0·92930	0·66738	1·72279
2×10^{8}	0·40343	0·44670	0·50839	0·51716	0·58300	0·46346	0·54133	0·47612	0·58300
2×10^{8}	1·03750	1·08758	1·18104	1·98849					1·98849
2×10^{8}	0·92444	1·12108	1·14131	1·34008	0·74836	0·56072	0·78195	0·64960	1·34008
2×10^{8}	0·17272	0·14152	0·14350	0·19144	0·22752	0·23925	0·25204	0·24717	0·25204
2×10^{8}	0·54132	0·49910	0·51376	0·80185	0·97375	0·76576	0·75622	0·83623	0·97375
2×10^{8}	0·25754	0·27236	0·23769	0·22015	0·18087	0·18754	0·18501	0·20329	0·27236
2×10^{8}	0·70153	0·93721	1·00539	1·00539	0·94120	0·77868	0·87036	0·94721	1·00539
2×10^{8}	0·18669	0·18838	0·14351	0·16775	0·15042		0·14283	0·16168	0·18838
2×10^{8}	0·25368	0·19180	0·31415	0·30691	0·34109	0·30238	0·27003	0·27826	0·34109

Fold change TcpA IgM									
**Dose (CFU)**	**D1**	**D4**	**D7**	**D15**	**D29**	**D57**	**D85**	**D180**	**max FC**
0	1·00	1·04	1·09	0·99	1·17	1·02	1·27	1·48	1·48
0	1·00	0·96	0·96	0·85	0·94	1·00	0·60	0·51	1·00
0	1·00	1·15	1·17	1·03	1·13	0·76	0·58	1·22	1·22
0	1·00	1·13	1·06	0·87	0·82	14·84	3·40	99·37	99·37
0	1·00	0·84	1·24	1·05	1·15	2·08	1·84	1·32	2·08
0	1·00	1·08	1·17	0·97	0·52	0·93	0·83	1·04	1·17
0	1·00	0·84	1·18	0·96	1·56	1·48	0·74	1·62	1·62
2×10^{7}	1·00	0·86	0·71	0·88	0·97	0·72	0·74	0·94	0·97
2×10^{7}	1·00	0·96	1·23	9·76	4·77	1·75	2·01	1·30	9·76
2×10^{7}	1·00	1·19	0·99	1·24	1·00	0·87	0·72	0·77	1·24
2×10^{7}	1·00	1·68	1·42	3·54	1·60	1·77	1·37	1·56	3·54
2×10^{7}	1·00	0·83	1·39	4·53	2·00	0·87	0·79	0·84	4·53
2×10^{7}	1·00	1·00	0·93	1·27	1·64	1·46	1·71	1·84	1·84
2×10^{7}	1·00		0·97	0·84	0·76	0·89	1·20		1·20
2×10^{7}	1·00	1·07	0·99	1·32	1·22	1·70	1·39	1·31	1·70
2×10^{7}	1·00	1·11	0·98	0·94	0·74	0·78	0·49	0·91	1·11
2×10^{7}	1·00	1·29	1·71	1·57	1·02	0·98	0·94	1·06	1·71
2×10^{7}	1·00	1·44	2·78	8·55	8·11	3·77	2·26	1·68	8·55
2×10^{7}	1·00	0·97	1·39	1·40	1·33	1·38	1·32	1·10	1·40
2×10^{7}	1·00	1·09	2·03	2·75	1·76	1·17	1·10	1·01	2·75
2×10^{7}	1·00	0·69	1·18	0·99	1·04	1·21		0·97	1·21
2×10^{8}	1·00	1·03	1·20	3·24	1·46	0·92	24·19	1·43	24·19
2×10^{8}	1·00	0·98	1·22	1·46	1·16	1·28	0·97	0·45	1·46
2×10^{8}	1·00	0·99	1·15	1·40	0·87	1·48	1·05	0·94	1·48
2×10^{8}	1·00	1·09	1·00	0·55	0·72	0·73	0·59	0·42	1·09
2×10^{8}	1·00	1·11	1·26	1·28	1·45	1·15	1·34	1·18	1·45
2×10^{8}	1·00	1·05	1·14	1·92					1·92
2×10^{8}	1·00	1·21	1·23	1·45	0·81	0·61	0·85	0·70	1·45
2×10^{8}	1·00	0·82	0·83	1·11	1·32	1·39	1·46	1·43	1·46
2×10^{8}	1·00	0·92	0·95	1·48	1·80	1·41	1·40	1·54	1·80
2×10^{8}	1·00	1·06	0·92	0·85	0·70	0·73	0·72	0·79	1·06
2×10^{8}	1·00	1·34	1·43	1·43	1·34	1·11	1·24	1·35	1·43
2×10^{8}	1·00	1·01	0·77	0·90	0·81		0·77	0·87	1·01
2×10^{8}	1·00	0·76	1·24	1·21	1·34	1·19	1·06	1·10	1·34

	**RAU**		
	D1	D39	
V. cholerae	0·83096	1·19207	
	2·25324	2·41178	
	0·50720	0·67092	
	0·20096	0·23126	
	41·29516	43·55952	
	D1	D11	
Vaxchora D11 Challenge	0·30896	0·27502	
	1·24626	1·27372	
	0·45114	0·41675	
	0·49911	0·44099	
	0·31501	0·37446	
	0·58859	0·47500	
	D1	D11	D91
Vaxchora D91 Challenge	0·26483	0·23649	0·25012
	1·19207	0·53322	0·49142
	0·12446	0·12915	0·15760
	0·60910	0·47309	0·81284
	0·81504	1·02717	0·79180
	0·20897	0·24033	0·26764

	**RAU fold change**			**max FC**
	D1	D39		
V. cholerae	1·00	1·43		1·43
	1·00	1·07		1·07
	1·00	1·32		1·32
	1·00	1·15		1·15
	1·00	1·05		1·05
	D1	D11		
Vaxchora D11 Challenge	1·00	0·89		0·89
	1·00	1·02		1·02
	1·00	0·92		0·92
	1·00	0·88		0·88
	1·00	1·19		1·19
	1·00	0·81		0·81
	D1	D11	D91	
Vaxchora D91 Challenge	1·00	0·89	0·94	0·94
	1·00	0·45	0·41	0·45
	1·00	1·04	1·27	1·27
	1·00	0·78	1·33	1·33
	1·00	1·26	0·97	1·26
	1·00	1·15	1·28	1·28

Dose-Escalation Module

TcpA IgG (RAU)									
Dose (CFU)	D1	D4	D7	D15	D29	D57	D85	D180	max
10^5	0·00447	0·00294	0·00593	0·00447	0·00593	0·04180	0·01922	0·00788	0·04180
10^5	0·00358	0·00349	0·00737	0·00675	0·00506	0·01151	0·01684		0·01684
10^5	0·01062	0·01195	0·00995	0·01595	0·02076	0·05916	0·09098		0·09098
10^6	0·00707	0·00619	0·00583	0·01784	0·01291	0·01080	0·01194		0·01784
10^6	0·00565	0·00583	0·00565	0·00931	0·00777	0·00964	0·00511	0·00981	0·00981
10^6	0·02322	0·01918	0·01932	0·01754	0·02222	0·02477	0·02208	0·01121	0·02477
10^7	0·01291	0·01338	0·01146	0·01526	0·03602	0·07304	0·06159	0·01666	0·07304
10^7	0·00707	0·00829	0·00742	0·00547	0·00829	0·00637	0·00742	0·00448	0·00829
10^7	0·03615	0·03010	0·03472	0·02753	0·02889	0·02463	0·02616	0·00755	0·03615
10^8	0·02136	0·02364	0·02546	0·02265	0·02698	0·01417	0·01754	0·00652	0·02698
10^8	0·01146	0·01064	0·00760	0·00742	0·00742	0·00846	0·00652	0·00716	0·01146
10^8	0·03024	0·02643	0·02983	0·03498	0·05667	0·65286	1·02342	0·94380	1·02342
10^9	0·01194	0·01307	0·01918	0·01769	0·01495	0·00677	0·00911	0·00768	0·01918
10^9	0·01510	0·01572	0·01784	0·01068	0·01200	0·01068	0·02579		0·02579
10^9	0·00537	0·00524	0·00537	0·00575	0·00613	0·00613	0·00587	0·00486	0·00613
10^{10}	0·00136	0·00312	0·00448	0·01162	0·00716		0·00639	0·00511	0·01162
10^{10}	0·00264	0·00312	0·00312	0·00386			0·00373	0·00361	0·00386
10^{10}	0·01506	0·01613	0·01519	0·01280	0·02421	0·03881	0·03073	0·02502	0·03881

Fold change TcpA IgG									
Dose (CFU)	D1	D4	D7	D15	D29	D57	D85	D180	max FC
10^5	1·00	0·66	1·33	1·00	1·33	9·35	4·30	1·76	9·35
10^5	1·00	0·97	2·06	1·88	1·41	3·21	4·70		4·70
10^5	1·00	1·12	0·94	1·50	1·95	5·57	8·56		8·56
10^6	1·00	0·88	0·82	2·52	1·82	1·53	1·69		2·52
10^6	1·00	1·03	1·00	1·65	1·37	1·71	0·90	1·74	1·74
10^6	1·00	0·83	0·83	0·76	0·96	1·07	0·95	0·48	1·07
10^7	1·00	1·04	0·89	1·18	2·79	5·66	4·77	1·29	5·66
10^7	1·00	1·17	1·05	0·77	1·17	0·90	1·05	0·63	1·17
10^7	1·00	0·83	0·96	0·76	0·80	0·68	0·72	0·21	0·96
10^8	1·00	1·11	1·19	1·06	1·26	0·66	0·82	0·30	1·26
10^8	1·00	0·93	0·66	0·65	0·65	0·74	0·57	0·62	0·93
10^8	1·00	0·87	0·99	1·16	1·87	21·59	33·85	31·21	33·85
10^9	1·00	1·09	1·61	1·48	1·25	0·57	0·76	0·64	1·61
10^9	1·00	1·04	1·18	0·71	0·79	0·71	1·71		1·71
10^9	1·00	0·98	1·00	1·07	1·14	1·14	1·09	0·91	1·14
10^{10}	1·00	2·30	3·30	8·56	5·27		4·70	3·76	8·56
10^{10}	1·00	1·18	1·18	1·46			1·42	1·37	1·46
10^{10}	1·00	1·07	1·01	0·85	1·61	2·58	2·04	1·66	2·58

Dose-Expansion Module

TcpA IgG (RAU)									
Dose (CFU)	D1	D4	D7	D15	D29	D57	D85	D180	max
0	0·00867	0·01050	0·00935	0·00946	0·01016	0·00985	0·01072	0·01142	0·01142
0	0·01565	0·01776	0·01621	0·01373	0·01595	0·01634	0·01618	0·01300	0·01776
0	0·02299	0·02438	0·02660	0·02412	0·02512	0·02561	0·02471	0·00525	0·02660
0	0·02942	0·02671	0·02978	0·02674	0·02908	0·01107	0·00746	0·00442	0·02978
0	0·01106	0·01204	0·01106	0·01307	0·00993	0·00280	0·00403	0·00182	0·01307
0	0·00553	0·00747	0·00642	0·00033		0·00024		0·00002	0·00747
0	0·01049	0·00872	0·00746	0·01146	0·01273	0·00545	0·00670	0·00890	0·01273
2×10^7	0·07588	0·07588	0·06883	0·08107	0·20121	0·09726	0·08715	0·08879	0·20121
2×10^7	0·01578	0·01637	0·01522	0·01893	0·01709	0·01699	0·01858	0·01676	0·01893
2×10^7	0·01001	0·01001	0·00989	0·01261	0·01087	0·01575	0·01240	0·01057	0·01575
2×10^7	0·00974	0·01211	0·01168	0·01390	0·01095	0·02008	0·01959	0·01529	0·02008
2×10^7	0·01608	0·01641	0·01427	0·02482	0·02657	0·05390	0·05512	0·04582	0·05512
2×10^7	0·01562	0·01421	0·01397	0·01355	0·01488	0·01912	0·02257	0·01018	0·02257
2×10^7	0·01833		0·01909	0·02002	0·02100	0·01808	0·01320		0·02100
2×10^7	0·00993	0·01027	0·00997	0·01345	0·01400	0·00718	0·00378	0·00427	0·01400
2×10^7	0·01502	0·01268	0·01296	0·01407	0·01282	0·00235	0·00442	0·00329	0·01502
2×10^7	0·01581	0·01508	0·01750	0·01618	0·03640	0·05295	0·05178	0·04763	0·05295
2×10^7	0·00545	0·00260	0·00516	0·00486	0·00904	0·02353	0·03134	0·02839	0·03134
2×10^7	0·00477	0·00467	0·00349	0·00555	0·00755	0·00774	0·00858	0·00486	0·00858
2×10^7	0·00270	0·00388	0·00442	0·00344	0·00457	0·00353	0·00491	0·00447	0·00491
2×10^7	0·00383	0·00437	0·00574	0·00275	0·00289	0·00447		0·00231	0·00574
2×10^8	0·01247	0·01444	0·01208	0·02435	0·01471	0·02106	0·02224	0·01731	0·02435
2×10^8	0·00591	0·00526	0·00610	0·00469	0·00291	0·00530	0·00286	0·00391	0·00610
2×10^8	0·00997	0·01275	0·01050	0·01390	0·01502	0·02287	0·02182	0·01780	0·02287
2×10^8	0·01562	0·01588	0·01558	0·01410	0·02042	0·07168	0·07542	0·02353	0·07542
2×10^8	0·00619	0·00806	0·00814	0·00997	0·01153	0·00164	0·00083	0·00043	0·01153
2×10^8	0·01397	0·01349	0·01300	0·01451					0·01451
2×10^8	0·01495	0·01545	0·01673	0·02134	0·01476	0·01836	0·02456	0·01463	0·02456
2×10^8	0·00426	0·00297	0·00468	0·00568	0·00066	0·00043	0·00078	0·00066	0·00568
2×10^8	0·01012	0·01072	0·00935	0·00462	0·00353	0·00398	0·00521	0·00319	0·01072
2×10^8	0·00718	0·01004	0·00812	0·00904	0·01337	0·02250	0·02015	0·01632	0·02250
2×10^8	0·00314	0·00270	0·00211	0·00280	0·00226	0·00240	0·00211	0·00245	0·00314
2×10^8	0·00646	0·00477	0·00289	0·00211	0·00187		0·00339	0·00250	0·00646
2×10^8	0·00231	0·00187	0·00589	0·01816	0·01430	0·01844	0·01595	0·01753	0·01844

Fold change TcpA IgG									
Dose (CFU)	D1	D4	D7	D15	D29	D57	D85	D180	max FC
0	1·00	1·21	1·08	1·09	1·17	1·14	1·24	1·32	1·32
0	1·00	1·13	1·04	0·88	1·02	1·04	1·03	0·83	1·13
0	1·00	1·06	1·16	1·05	1·09	1·11	1·07	0·23	1·16
0	1·00	0·91	1·01	0·91	0·99	0·38	0·25	0·15	1·01
0	1·00	1·09	1·00	1·18	0·90	0·25	0·36	0·16	1·18
0	1·00	1·35	1·16	0·06		0·04		0·003	1·35
0	1·00	0·83	0·71	1·09	1·21	0·52	0·64	0·85	1·21
2×10^7	1·00	1·00	0·91	1·07	2·65	1·28	1·15	1·17	2·65
2×10^7	1·00	1·04	0·96	1·20	1·08	1·08	1·18	1·06	1·20
2×10^7	1·00	1·00	0·99	1·26	1·09	1·57	1·24	1·06	1·57
2×10^7	1·00	1·24	1·20	1·43	1·12	2·06	2·01	1·57	2·06
2×10^7	1·00	1·02	0·89	1·54	1·65	3·35	3·43	2·85	3·43
2×10^7	1·00	0·91	0·89	0·87	0·95	1·22	1·45	0·65	1·45
2×10^7	1·00		1·04	1·09	1·15	0·99	0·72		1·15
2×10^7	1·00	1·03	1·00	1·35	1·41	0·72	0·38	0·43	1·41
2×10^7	1·00	0·84	0·86	0·94	0·85	0·16	0·29	0·22	0·94
2×10^7	1·00	0·95	1·11	1·02	2·30	3·35	3·27	3·01	3·35
2×10^7	1·00	0·48	0·95	0·89	1·66	4·32	5·75	5·21	5·75
2×10^7	1·00	0·98	0·73	1·16	1·59	1·62	1·80	1·02	1·80
2×10^7	1·00	1·44	1·64	1·27	1·69	1·31	1·82	1·66	1·82
2×10^7	1·00	1·14	1·50	0·72	0·76	1·17		0·60	1·50
2×10^8	1·00	1·16	0·97	1·95	1·18	1·69	1·78	1·39	1·95
2×10^8	1·00	0·89	1·03	0·79	0·49	0·90	0·48	0·66	1·03
2×10^8	1·00	1·28	1·05	1·39	1·51	2·29	2·19	1·79	2·29
2×10^8	1·00	1·02	1·00	0·90	1·31	4·59	4·83	1·51	4·83
2×10^8	1·00	1·30	1·32	1·61	1·86	0·26	0·13	0·07	1·86
2×10^8	1·00	0·97	0·93	1·04					1·04
2×10^8	1·00	1·03	1·12	1·43	0·99	1·23	1·64	0·98	1·64
2×10^8	1·00	0·70	1·10	1·33	0·16	0·10	0·18	0·16	1·33
2×10^8	1·00	1·06	0·92	0·46	0·35	0·39	0·51	0·32	1·06
2×10^8	1·00	1·40	1·13	1·26	1·86	3·13	2·81	2·27	3·13
2×10^8	1·00	0·86	0·67	0·89	0·72	0·77	0·67	0·78	0·89
2×10^8	1·00	0·74	0·45	0·33	0·29		0·52	0·39	0·74
2×10^8	1·00	0·81	2·55	7·87	6·20	7·99	6·92	7·60	7·99

	RAU		
	D1	D39	
V. cholerae	0·03223	0·10861	
	0·02726	0·04767	
	0·02983	0·06321	
	0·02712	0·10099	
	0·15925	0·14285	
	D1	D11	
Vaxchora D11 Challenge	0·06414	0·08354	
	0·10390	0·11117	
	0·09341	0·07932	
	0·06356	0·06827	
	0·06124	0·06655	
	0·05537	0·03947	
	D1	D11	D91
Vaxchora D91 Challenge	0·05832	0·08243	0·08519
	0·01541	0·02546	0·04621
	0·03769	0·05513	0·04645
	0·08915	0·09287	0·08597
	0·01962	0·04694	0·05820
	0·01275	0·01814	0·01769

	RAU fold change			max FC
	D1	D39		
V. cholerae	1·00	3·37		3·37
	1·00	1·75		1·75
	1·00	2·12		2·12
	1·00	3·72		3·72
	1·00	0·90		0·90
	D1	D11		
Vaxchora D11 Challenge	1·00	1·30		1·30
	1·00	1·07		1·07
	1·00	0·85		0·85
	1·00	1·07		1·07
	1·00	1·09		1·09
	1·00	0·71		0·71
	D1	D11	D91	
Vaxchora D91 Challenge	1·00	1·41	1·46	1·46
	1·00	1·65	3·00	3·00
	1·00	1·46	1·23	1·46
	1·00	1·04	0·96	1·04
	1·00	2·39	2·97	2·97
	1·00	1·42	1·39	1·42

Dose-Escalation Module

TcpA IgA (RAU)									
Dose (CFU)	D1	D4	D7	D15	D29	D57	D85	D180	max
10^{5}	0·02262	0·02515	0·02399	0·01942	0·02499	0·03304	0·01943	0·01786	0·03304
10^{5}	0·01054	0·00950	0·01079	0·01215	0·01005	0·00975	0·01166		0·01215
10^{5}	0·00891	0·00921	0·00863	0·00905	0·01686	0·03347	0·02929		0·03347
10^{6}	0·00706	0·00836	0·00734	0·00942	0·00981	0·00967	0·01038		0·01038
10^{6}	0·02641	0·02250	0·01791	0·01732	0·01640	0·01876	0·01739	0·01833	0·02641
10^{6}	0·01014	0·01601	0·01142	0·01314	0·00904	0·01098	0·00784	0·00784	0·01601
10^{7}	0·01149	0·01156	0·01180	0·01552	0·02013	0·02098	0·02412	0·01235	0·02412
10^{7}	0·00972	0·00753	0·00768	0·00652	0·00742	0·00528	0·00730	0·00284	0·00972
10^{7}	0·01488	0·01175	0·00986	0·01168	0·01280	0·00892	0·01273	0·00177	0·01488
10^{8}	0·01601	0·01628	0·01381	0·01474	0·01442	0·01430	0·01704	0·01051	0·01704
10^{8}	0·01614	0·01229	0·01416	0·02266	0·02323	0·01906	0·01161	0·01334	0·02323
10^{8}	0·00430	0·00399	0·00614	0·00942	0·01755	0·07457	0·09681	0·16468	0·16468
10^{9}	0·01672	0·01695	0·02713	0·02061	0·02287	0·01293	0·01103	0·01089	0·02713
10^{9}	0·03542	0·02935	0·02804	0·01840	0·01860	0·01515	0·02818		0·03542
10^{9}	0·01193	0·01538	0·01357	0·01522	0·01413	0·01198	0·01461	0·01293	0·01538
10^{10}	0·00396	0·00365	0·00531	0·00645	0·01074		0·00723	0·01155	0·01155
10^{10}	0·01187	0·01227	0·01124	0·01123			0·01357	0·01256	0·01357
10^{10}	0·00722	0·00807	0·00709	0·01268	0·01114	0·00939	0·01042	0·00924	0·01268

Fold change TcpA IgA									
Dose (CFU)	D1	D4	D7	D15	D29	D57	D85	D180	max FC
10^{5}	1·00	1·11	1·06	0·86	1·10	1·46	0·86	0·79	1·46
10^{5}	1·00	0·90	1·02	1·15	0·95	0·92	1·11		1·15
10^{5}	1·00	1·03	0·97	1·02	1·89	3·76	3·29		3·76
10^{6}	1·00	1·18	1·04	1·33	1·39	1·37	1·47		1·47
10^{6}	1·00	0·85	0·68	0·66	0·62	0·71	0·66	0·69	0·85
10^{6}	1·00	1·58	1·13	1·30	0·89	1·08	0·77	0·77	1·58
10^{7}	1·00	1·01	1·03	1·35	1·75	1·82	2·10	1·07	2·10
10^{7}	1·00	0·78	0·79	0·67	0·76	0·54	0·75	0·29	0·79
10^{7}	1·00	0·79	0·66	0·79	0·86	0·60	0·86	0·12	0·86
10^{8}	1·00	1·02	0·86	0·92	0·90	0·89	1·06	0·66	1·06
10^{8}	1·00	0·76	0·88	1·40	1·44	1·18	0·72	0·83	1·44
10^{8}	1·00	0·93	1·43	2·19	4·08	17·34	22·51	38·29	38·29
10^{9}	1·00	1·01	1·62	1·23	1·37	0·77	0·66	0·65	1·62
10^{9}	1·00	0·83	0·79	0·52	0·53	0·43	0·80		0·83
10^{9}	1·00	1·29	1·14	1·28	1·18	1·00	1·22	1·08	1·29
10^{10}	1·00	0·92	1·34	1·63	2·71		1·83	2·92	2·92
10^{10}	1·00	1·03	0·95	0·95			1·14	1·06	1·14
10^{10}	1·00	1·12	0·98	1·76	1·54	1·30	1·44	1·28	1·76

Dose-Expansion Module

TcpA IgA (RAU)									
Dose (CFU)	D1	D4	D7	D15	D29	D57	D85	D180	max
0	0·01085	0·01276	0·01276	0·01207	0·01190	0·01125	0·01113	0·01235	0·01276
0	0·00733	0·00576	0·00694	0·00589	0·00733	0·00707	0·00723	0·00681	0·00733
0	0·00520	0·00605	0·00485	0·00385	0·00552	0·00626	0·00520	0·00466	0·00626
0	0·03047	0·02436	0·02850	0·02520	0·01957	0·02254	0·02164	0·02958	0·03047
0	0·00605	0·00705	0·00972	0·00744	0·00780	0·01031	0·00842	0·00762	0·01031
0	0·00404	0·00637	0·00493	0·00266	0·00310	0·00335	0·00365	0·00395	0·00637
0	0·00647	0·00501	0·00561	0·00547	0·00762	0·00494	0·00306	0·00469	0·00762
2×10^{7}	0·00589	0·00621	0·00570	0·01115	0·00937	0·00652	0·00733	0·00739	0·01115
2×10^{7}	0·00431	0·00562	0·00626	0·00757	0·00655	0·00728	0·01123	0·00490	0·01123
2×10^{7}	0·01133	0·01078	0·01068	0·01058	0·01230	0·00931	0·00987	0·00957	0·01230
2×10^{7}	0·01755	0·02239	0·01976	0·01738	0·01870	0·02807	0·02588	0·01922	0·02807
2×10^{7}	0·01788	0·01457	0·02023	0·03098	0·03022	0·02548	0·02053	0·01279	0·03098
2×10^{7}	0·01185	0·01299	0·01678	0·01997	0·02218	0·01894	0·01915	0·01221	0·02218
2×10^{7}	0·01535		0·01330	0·01472	0·01173	0·01045	0·01120		0·01535
2×10^{7}	0·00514	0·00634	0·00947	0·01428	0·01195	0·00761	0·00626	0·00605	0·01428
2×10^{7}	0·00896	0·00746	0·00898	0·01108	0·01002	0·01013	0·00997	0·01203	0·01203
2×10^{7}	0·00525	0·00671	0·00453	0·01068	0·00807	0·01054	0·00876	0·00561	0·01068
2×10^{7}	0·00364	0·00254	0·00292	0·00373	0·00596	0·00992	0·00689	0·00637	0·00992
2×10^{7}	0·00822	0·00927	0·01357	0·00974	0·00946	0·00835	0·01208	0·01136	0·01357
2×10^{7}	0·00200	0·00213	0·00288	0·00458	0·00392	0·00299	0·00332	0·00174	0·00458
2×10^{7}	0·00920	0·00749	0·00880	0·00543	0·00628	0·00725		0·00515	0·00920
2×10^{8}	0·01235	0·01472	0·01150	0·01733	0·01510	0·01028	0·01367	0·01491	0·01733
2×10^{8}	0·01480	0·01939	0·01831	0·01533	0·01996	0·02058	0·01742	0·00936	0·02058
2×10^{8}	0·00471	0·00482	0·00565	0·00637	0·00741	0·00837	0·00463	0·00597	0·00837
2×10^{8}	0·00967	0·00901	0·01000	0·00952	0·01374	0·01877	0·01196	0·01157	0·01877
2×10^{8}	0·00439	0·00272	0·00490	0·00455	0·00286	0·00251	0·00197	0·00171	0·00490
2×10^{8}	0·01296	0·01108	0·01070	0·01217					0·01296
2×10^{8}	0·00546	0·00702	0·00726	0·01681	0·00880	0·00830	0·00875	0·00650	0·01681
2×10^{8}	0·01088	0·00816	0·00893	0·00875	0·00604	0·00774	0·00774	0·00832	0·01088
2×10^{8}	0·01068	0·00898	0·01007	0·00863	0·01234	0·01134	0·01120	0·00985	0·01234
2×10^{8}	0·02204	0·02212	0·02345	0·02227	0·02840	0·02271	0·02103	0·01800	0·02840
2×10^{8}	0·00517	0·00602	0·00588	0·00656	0·00544	0·00552	0·00546	0·00531	0·00656
2×10^{8}	0·03359	0·04103	0·03486	0·02951	0·03299		0·04045	0·04911	0·04911
2×10^{8}	0·00398	0·00325	0·09183	0·14229	0·01544	0·00727	0·00779	0·00722	0·14229

Fold change TcpA IgA									
Dose (CFU)	D1	D4	D7	D15	D29	D57	D85	D180	max FC
0	1·00	1·18	1·18	1·11	1·10	1·04	1·03	1·14	1·18
0	1·00	0·79	0·95	0·80	1·00	0·96	0·99	0·93	1·00
0	1·00	1·16	0·93	0·74	1·06	1·20	1·00	0·90	1·20
0	1·00	0·80	0·94	0·83	0·64	0·74	0·71	0·97	0·97
0	1·00	1·17	1·61	1·23	1·29	1·71	1·39	1·26	1·71
0	1·00	1·58	1·22	0·66	0·77	0·83	0·90	0·98	1·58
0	1·00	0·77	0·87	0·85	1·18	0·76	0·47	0·72	1·18
2×10^{7}	1·00	1·05	0·97	1·89	1·59	1·11	1·25	1·25	1·89
2×10^{7}	1·00	1·31	1·45	1·76	1·52	1·69	2·61	1·14	2·61
2×10^{7}	1·00	0·95	0·94	0·93	1·09	0·82	0·87	0·84	1·09
2×10^{7}	1·00	1·28	1·13	0·99	1·07	1·60	1·48	1·10	1·60
2×10^{7}	1·00	0·82	1·13	1·73	1·69	1·43	1·15	0·72	1·73
2×10^{7}	1·00	1·10	1·42	1·69	1·87	1·60	1·62	1·03	1·87
2×10^{7}	1·00		0·87	0·96	0·76	0·68	0·73		0·96
2×10^{7}	1·00	1·23	1·84	2·78	2·32	1·48	1·22	1·18	2·78
2×10^{7}	1·00	0·83	1·00	1·24	1·12	1·13	1·11	1·34	1·34
2×10^{7}	1·00	1·28	0·86	2·03	1·54	2·01	1·67	1·07	2·03
2×10^{7}	1·00	0·70	0·80	1·03	1·64	2·73	1·90	1·75	2·73
2×10^{7}	1·00	1·13	1·65	1·18	1·15	1·02	1·47	1·38	1·65
2×10^{7}	1·00	1·06	1·44	2·29	1·95	1·49	1·66	0·87	2·29
2×10^{7}	1·00	0·81	0·96	0·59	0·68	0·79		0·56	0·96
2×10^{8}	1·00	1·19	0·93	1·40	1·22	0·83	1·11	1·21	1·40
2×10^{8}	1·00	1·31	1·24	1·04	1·35	1·39	1·18	0·63	1·39
2×10^{8}	1·00	1·02	1·20	1·35	1·57	1·78	0·98	1·27	1·78
2×10^{8}	1·00	0·93	1·03	0·98	1·42	1·94	1·24	1·20	1·94
2×10^{8}	1·00	0·62	1·12	1·04	0·65	0·57	0·45	0·39	1·12
2×10^{8}	1·00	0·85	0·83	0·94					0·94
2×10^{8}	1·00	1·28	1·33	3·08	1·61	1·52	1·60	1·19	3·08
2×10^{8}	1·00	0·75	0·82	0·80	0·55	0·71	0·71	0·76	0·82
2×10^{8}	1·00	0·84	0·94	0·81	1·16	1·06	1·05	0·92	1·16
2×10^{8}	1·00	1·00	1·06	1·01	1·29	1·03	0·95	0·82	1·29
2×10^{8}	1·00	1·17	1·14	1·27	1·05	1·07	1·06	1·03	1·27
2×10^{8}	1·00	1·22	1·04	0·88	0·98		1·20	1·46	1·46
2×10^{8}	1·00	0·82	23·08	35·76	3·88	1·83	1·96	1·82	35·76

	RAU		
	D1	D39	
V. cholerae	0·03990	0·07217	
	0·03895	0·04397	
	0·03091	0·03357	
	0·01042	0·15261	
	0·24698	0·18283	
	D1	D11	
Vaxchora D11 Challenge	0·04026	0·04608	
	0·08187	0·08196	
	0·08873	0·06844	
	0·02863	0·03028	
	0·02530	0·02752	
	0·05529	0·02441	
	D1	D11	D91
Vaxchora D91 Challenge	0·01284	0·01205	0·01833
	0·01284	0·01979	0·02002
	0·01910	0·03535	0·02036
	0·01442	0·02344	0·01894
	0·01289	0·02750	0·04008
	0·01706	0·01961	0·02152

	RAU fold change			max FC
	D1	D39		
V. cholerae	1·00	1·81		1·81
	1·00	1·13		1·13
	1·00	1·09		1·09
	1·00	14·64		14·64
	1·00	0·74		0·74
	D1	D11		
Vaxchora D11 Challenge	1·00	1·14		1·14
	1·00	1·00		1·00
	1·00	0·77		0·77
	1·00	1·06		1·06
	1·00	1·09		1·09
	1·00	0·44		0·44
	D1	D11	D91	
Vaxchora D91 Challenge	1·00	0·94	1·43	1·43
	1·00	1·54	1·56	1·56
	1·00	1·85	1·07	1·85
	1·00	1·63	1·31	1·63
	1·00	2·13	3·11	3·11
	1·00	1·15	1·26	1·26

Table S9: Geometric mean peak fold increase in antigen- and isotype-specific immune responses to Inaba, and Ogawa OSP, CT-B and TcpA in serum of vaccine and placebo recipients. PanChol group represents combined data of all vaccine recipients. Placebo:7; PanChol:45.

	IgM	IgG	IgA
**Placebo-geometric mean peak fold increase (95% CI)**			
**Inaba OSP**	1·2 (1·1-1·4)	1·3 (1·0-1·6)	1·5 (1·2-1·9)
**Ogawa OSP**	1·4 (1·1-1·7)	1·4 (1·1-1·7)	1·3 (1·0-1·7)
**CT-B**	2·2 (1·1-4·4)	1·2 (1·0-1·6)	1·3 (1·0-1·8)
**TcpA**	2·6 (0·6-12·0)	1·2 (1·1-1·3)	1·2 (1·0-1·5)
**PanChol-geometric mean peak fold increase (95% CI)**			
**Inaba OSP**	60·0 (35·0-101·0)	6·9 (4·1-12·0)	19·0 (10·0-35·0)
**Ogawa OSP**	36·0 (20·0-63·0)	8·6 (4·8-16·0)	11·0 (6·3-18·0)
**CT-B**	1·4 (1·2-1·6)	5·0 (3·5-7·1)	4·1 (2·8-6·0)
**TcpA**	1·9 (1·5-2·3)	2·1 (1·7-2·7)	1·7 (1·4-2·2)

Table S10: Antigen- and isotype-specific antibody responses in lymphocyte supernatant of vaccine and placebo recipients. Dark blue: no ALS sample; yellow: below limit of detection; D: day.

Dose-Escalation Module

Inaba OSP IgM (RAU)								
**Dose (CFU)**	**D1**	**D7**	**D15**	**D29**	**D57**	**D85**	**D180**	**max**
10^5		1·95E-07		1·49E-06				1·49E-06
10^5		3·89E-05	1·55E-05	4·24E-06				3·89E-05
10^5	6·55E-07	9·26E-04	5·88E-06	1·55E-06				9·26E-04
10^6		2·71E-02		1·39E-06	8·96E-06	1·79E-06		2·71E-02
10^6		9·23E-05						9·23E-05
10^6		4·43E-04	1·15E-03					1·15E-03
10^7		9·79E-04	4·74E-06	3·33E-05	6·20E-06	1·51E-06		9·79E-04
10^7		1·97E-03	2·78E-06		4·68E-07			1·97E-03
10^7	3·28E-07	2·19E-04	3·57E-05	6·34E-06		6·23E-07		2·19E-04
10^8		2·02E-05		1·63E-07		5·29E-07		2·02E-05
10^8		4·18E-03	2·60E-06	3·73E-06	6·82E-06	1·14E-05	1·45E-06	4·18E-03
10^8		8·30E-03	1·07E-07	1·23E-06	7·55E-06	4·62E-07		8·30E-03
10^9		2·20E-02	1·00E-04	5·98E-05	7·90E-07		1·64E-05	2·20E-02
10^9		7·76E-05	1·40E-04	1·27E-04	4·06E-05	1·00E-05		1·40E-04
10^9		1·80E-05		1·89E-06		3·05E-06	9·70E-07	1·80E-05
10^{10}		2·60E-04		5·01E-06		3·40E-07		2·60E-04
10^{10}		1·13E-01	2·52E-05			3·88E-07		1·13E-01
10^{10}		2·59E-02	1·64E-05	8·76E-05	4·48E-05	5·87E-06	5·30E-08	2·59E-02

Dose-Expansion Module

Inaba OSP IgM (RAU)								
**Dose (CFU)**	**D1**	**D7**	**D15**	**D29**	**D57**	**D85**	**D180**	**max**
0	1·59E-04	1·65E-04	1·47E-04	8·73E-06	2·79E-04		5·05E-04	5·05E-04
0	1·57E-06	2·30E-06		3·79E-07				2·30E-06
0	3·28E-06	9·03E-07	2·30E-06	3·00E-06				3·28E-06
0	2·58E-06	1·70E-06	6·28E-07	2·03E-06			5·00E-07	2·58E-06
0		6·81E-07	2·30E-06	4·26E-07			3·14E-07	2·30E-06
0	4·67E-06	5·21E-08					3·57E-07	4·67E-06
0	6·00E-07	7·08E-07						7·08E-07
2×10^7	1·47E-04		4·15E-04	3·24E-04			3·31E-04	4·15E-04
2×10^7	1·78E-04	1·14E-02	7·82E-04	6·22E-04			3·11E-04	1·14E-02
2×10^7	3·34E-07	6·67E-02	3·23E-05	1·68E-04			4·74E-06	6·67E-02
2×10^7	1·57E-06	3·64E-06	3·57E-06	2·79E-06			4·50E-07	3·64E-06
2×10^7	2·91E-07	1·78E-03	1·71E-05	8·33E-05				1·78E-03
2×10^7	5·24E-07	2·27E-04	2·17E-06	2·72E-06				2·27E-04
2×10^7	2·65E-06	4·37E-06	2·37E-06	1·71E-05				1·71E-05
2×10^7	1·32E-06	1·48E-03		8·46E-07			1·28E-07	1·48E-03
2×10^7	5·24E-07	1·24E-02	2·84E-05	7·25E-05			9·74E-08	1·24E-02
2×10^7	2·91E-07	9·47E-04	2·10E-04	4·15E-06			1·95E-07	9·47E-04
2×10^7	1·97E-06	3·06E-04	1·11E-06	5·03E-06			6·55E-07	3·06E-04
2×10^7		3·07E-04	4·77E-04	6·37E-06			7·08E-07	4·77E-04
2×10^7	3·14E-07	9·34E-03	7·79E-06	3·39E-06			1·62E-06	9·34E-03
2×10^7		4·77E-04	1·20E-05	1·81E-06			1·30E-06	4·77E-04
2×10^8	1·11E-04	9·13E+00	1·66E-02	6·08E-03	2·15E-03		2·34E-04	9·13E+00
2×10^8	2·15E-04	4·64E-02	8·79E-05	8·77E-04				4·64E-02
2×10^8	6·28E-07	4·45E-03	1·93E-05	4·23E-06			1·24E-06	4·45E-03
2×10^8	6·81E-07	1·43E-04	9·77E-05	1·92E-05				1·43E-04
2×10^8	7·90E-07	2·22E-03	2·72E-06	1·32E-06			3·14E-07	2·22E-03
2×10^8			1·57E-06					1·57E-06
2×10^8	1·37E-07	3·79E-06	3·71E-06	7·41E-06				7·41E-06
2×10^8	1·05E-07	1·97E-06	1·77E-06	6·28E-07				1·97E-06
2×10^8	1·02E-06	1·76E-01	4·79E-04	2·22E-05			4·63E-05	1·76E-01
2×10^8		3·65E-03	2·15E-06	5·60E-05				3·65E-03
2×10^8	1·68E-06	4·46E-05	3·70E-05	5·80E-06			2·76E-06	4·46E-05
2×10^8		1·93E-04	1·95E-07	2·42E-06				1·93E-04
2×10^8		2·37E-03	2·21E-06	5·88E-06				2·37E-03

no ALS sample
below limit of detection

D57 and D85 ALS samples were mainly collected during the dose-escalation module.

Dose-Escalation Module

Inaba OSP IgG (RAU)								
**Dose (CFU)**	**D1**	**D7**	**D15**	**D29**	**D57**	**D85**	**D180**	**max**
10^5				4·61E-05			7·87E-06	4·61E-05
10^5			7·33E-05					7·33E-05
10^5			7·87E-06					7·87E-06
10^6		1·27E-05	5·84E-06		1·62E-04	7·25E-05		1·62E-04
10^6	1·27E-05	3·76E-05			1·44E-05			3·76E-05
10^6	8·59E-06	2·50E-03	5·78E-03	5·84E-06	1·44E-05	5·84E-06	1·35E-05	5·78E-03
10^7		5·84E-06			1·27E-05	2·29E-05	2·10E-04	2·10E-04
10^7		3·66E-04	1·08E-05	2·97E-05		5·84E-06	8·85E-06	3·66E-04
10^7						2·97E-05	1·13E-05	2·97E-05
10^8			1·27E-05		2·53E-05		4·26E-05	4·26E-05
10^8		2·64E-04	5·84E-06			1·24E-05		2·64E-04
10^8				1·27E-05	1·69E-06	2·65E-05	1·78E-05	2·65E-05
10^9	5·84E-06				1·78E-05	3·34E-06		1·78E-05
10^9			4·85E-06	3·01E-05	8·85E-06	2·31E-04		2·31E-04
10^9	1·69E-06	7·58E-06	8·85E-06	1·01E-05	4·85E-06		8·85E-06	1·01E-05
10^{10}	6·25E-06	2·08E-05	1·24E-05	6·25E-06		7·58E-06	7·58E-06	2·08E-05
10^{10}	3·34E-06	1·69E-06				1·69E-06	4·85E-06	4·85E-06
10^{10}	1·01E-05	8·85E-06	1·01E-05		1·01E-05		2·83E-05	2·83E-05

Dose-Expansion Module

Inaba OSP IgG (RAU)								
**Dose (CFU)**	**D1**	**D7**	**D15**	**D29**	**D57**	**D85**	**D180**	**max**
0	4·67E-04	6·48E-04	2·16E-04	1·78E-04	5·79E-04		1·25E-04	6·48E-04
0	1·46E-05			5·22E-06			1·81E-05	1·81E-05
0	8·10E-06	8·10E-06	4·82E-06	4·43E-06				8·10E-06
0		1·51E-05	3·30E-06	1·42E-05				1·51E-05
0	6·02E-06	1·95E-05	8·95E-06	8·53E-06				1·95E-05
0	8·53E-06	8·10E-06						8·53E-06
0								
2×10^7	6·35E-05	7·63E-04	1·78E-04	9·27E-05			1·27E-03	1·27E-03
2×10^7	5·12E-04	7·11E-03	5·12E-04	1·25E-04			9·02E-04	7·11E-03
2×10^7	5·62E-06	5·30E-05	1·92E-06	2·24E-06			7·26E-06	5·30E-05
2×10^7		8·10E-06		5·62E-06				8·10E-06
2×10^7	8·95E-06	1·68E-05	1·51E-05	2·93E-06			4·40E-05	4·40E-05
2×10^7	1·92E-06	6·02E-06	8·08E-08					6·02E-06
2×10^7		1·07E-05	3·67E-06	3·30E-06				1·07E-05
2×10^7	4·04E-06	1·29E-05	6·43E-06	4·43E-06				1·29E-05
2×10^7	1·73E-05	2·04E-05	8·10E-06	1·42E-05				2·04E-05
2×10^7		3·30E-06	1·02E-05					1·02E-05
2×10^7		2·43E-05					3·10E-05	3·10E-05
2×10^7								
2×10^7								
2×10^7								
2×10^8	5·12E-04	5·79E-04	7·86E-04	7·63E-04	4·23E-04		1·37E-03	1·37E-03
2×10^8	2·35E-04	6·35E-05	7·16E-04	9·27E-05			2·12E-06	7·16E-04
2×10^8	1·92E-06	2·70E-05		1·31E-06				2·70E-05
2×10^8	1·02E-05	1·59E-05	1·46E-05	3·48E-05			7·87E-06	3·48E-05
2×10^8	2·08E-05	9·38E-06	4·82E-06	5·50E-07				2·08E-05
2×10^8			6·02E-06					6·02E-06
2×10^8	1·07E-05	2·93E-06	2·93E-06	1·24E-05				1·24E-05
2×10^8	6·43E-06		6·43E-06	1·29E-05				1·29E-05
2×10^8		1·11E-04	2·58E-06	1·92E-06			9·73E-05	1·11E-04
2×10^8								
2×10^8							1·68E-04	1·68E-04
2×10^8				5·42E-05				5·42E-05
2×10^8	6·93E-07	4·47E-07	3·35E-07	1·89E-06			6·05E-06	6·05E-06

Dose-Escalation Module

Inaba OSP IgA (RAU)								
**Dose (CFU)**	**D1**	**D7**	**D15**	**D29**	**D57**	**D85**	**D180**	**max**
10^5	1·10E-06	1·18E-05	9·82E-07	1·10E-06			1·93E-06	1·18E-05
10^5	1·57E-06	3·10E-06	2·25E-05	5·78E-06				2·25E-05
10^5	2·28E-06	7·36E-06	3·50E-06	1·28E-05				1·28E-05
10^6	7·03E-07	6·81E-04	6·48E-06	8·60E-06	9·36E-05	8·67E-05		6·81E-04
10^6	6·58E-07	1·14E-02	1·28E-05	5·57E-06	2·25E-05	3·58E-06	2·21E-06	1·14E-02
10^6	1·31E-06	1·89E-04	1·34E-02	2·51E-06	1·12E-06	2·44E-07	1·90E-07	1·34E-02
10^7	7·75E-07	1·15E-07	1·41E-07	6·80E-07	1·92E-06	4·47E-05	3·06E-05	4·47E-05
10^7	9·68E-07	1·46E-03	5·01E-06	2·63E-06	7·82E-07	4·87E-06	9·37E-07	1·46E-03
10^7	1·64E-07	4·70E-05	2·61E-05	1·22E-06	9·06E-07	7·82E-07	4·16E-07	4·70E-05
10^8	9·06E-07	2·90E-06	1·41E-06	2·45E-06		1·16E-06	5·26E-06	5·26E-06
10^8	1·72E-06	9·70E-05	1·28E-06	1·22E-06	5·01E-06	2·02E-06	9·29E-06	9·70E-05
10^8	5·10E-08	2·61E-03	1·92E-06	5·13E-06	3·29E-05	2·06E-07		2·61E-03
10^9	9·06E-07	1·20E-05	3·17E-06	3·65E-05	8·93E-06	3·11E-06	9·06E-07	3·65E-05
10^9	3·20E-07	1·05E-04	2·67E-05	1·54E-04	2·71E-05	9·60E-05		1·54E-04
10^9	2·44E-07	1·72E-06	8·44E-07	1·84E-06	3·29E-06	8·44E-07	3·40E-06	3·40E-06
10^{10}	5·75E-08	4·92E-04	2·04E-06	1·68E-06				4·92E-04
10^{10}	5·97E-07	3·87E-06	2·52E-05			1·27E-05	2·57E-05	2·57E-05
10^{10}	2·16E-07	4·97E-04	1·08E-04	2·56E-04	8·32E-05	6·45E-05	1·54E-04	4·97E-04

Dose-Expansion Module

Inaba OSP IgA (RAU)								
**Dose (CFU)**	**D1**	**D7**	**D15**	**D29**	**D57**	**D85**	**D180**	**max**
0	1·90E-07	4·16E-07	3·28E-07		2·99E-07		2·44E-07	4·16E-07
0	4·75E-07	5·36E-07	1·16E-06	4·75E-07			8·13E-07	1·16E-06
0	2·16E-07	2·99E-07		5·97E-07			1·28E-06	1·28E-06
0	5·29E-07	1·10E-06	7·52E-07	2·93E-06			1·93E-06	2·93E-06
0	1·93E-06	9·24E-07	1·45E-06	9·82E-07			1·22E-06	1·93E-06
0	1·69E-06	7·52E-07	2·69E-06	4·23E-07			1·10E-06	2·69E-06
0	1·69E-06	6·95E-07	3·21E-07	1·22E-06			2·05E-06	2·05E-06
2×10^7	7·51E-07	1·54E-04	4·16E-07	7·82E-07			3·57E-07	1·54E-04
2×10^7		2·06E-02	5·60E-03	8·28E-03			9·81E-06	2·06E-02
2×10^7	1·59E-06	1·97E-04	4·47E-05	7·07E-08			1·16E-06	1·97E-04
2×10^7	6·89E-07	1·12E-06	8·13E-07	6·58E-07			9·06E-07	1·12E-06
2×10^7	1·57E-06	1·75E-04	1·42E-05	3·83E-05			2·64E-05	1·75E-04
2×10^7	5·29E-07	5·45E-05	7·36E-05	6·26E-06			9·13E-06	7·36E-05
2×10^7	7·52E-07	4·57E-06	2·10E-06	2·52E-06				4·57E-06
2×10^7	8·66E-07	3·62E-06	1·81E-06	2·16E-06			5·84E-07	3·62E-06
2×10^7	2·16E-06	1·26E-04	4·50E-05	3·20E-04			1·93E-05	3·20E-04
2×10^7	6·39E-07	7·80E-05	1·42E-04	2·98E-06			1·38E-05	1·42E-04
2×10^7	1·51E-06	2·40E-03	1·07E-04	7·95E-05			1·20E-04	2·40E-03
2×10^7	2·93E-06	6·73E-06	2·40E-06	1·19E-05			2·09E-05	2·09E-05
2×10^7	3·71E-07	1·71E-03	4·01E-06	2·28E-06			1·81E-06	1·71E-03
2×10^7	1·40E-07	3·14E-05	1·70E-05	1·81E-07			9·24E-07	3·14E-05
2×10^8	1·06E-04	3·33E-02	2·88E-03	6·17E-03	3·35E-04		8·42E-04	3·33E-02
2×10^8	2·97E-04	1·24E-03	7·81E-05				1·59E-06	1·24E-03
2×10^8	1·10E-06	9·67E-06	7·51E-06	9·18E-06			8·38E-06	9·67E-06
2×10^8	1·26E-06	4·89E-04	2·50E-05	7·04E-05			1·18E-05	4·89E-04
2×10^8	9·05E-07	1·15E-05	5·79E-07	4·32E-07				1·15E-05
2×10^8			2·43E-06					2·43E-06
2×10^8	4·32E-06	7·38E-07	7·19E-06	5·64E-06				7·19E-06
2×10^8	2·43E-06	1·63E-06		2·43E-06				2·43E-06
2×10^8	1·44E-06	1·12E-03	4·58E-05	2·22E-06			2·36E-05	1·12E-03
2×10^8	1·04E-06	8·40E-05	2·19E-06	1·84E-05			3·45E-06	8·40E-05
2×10^8	1·10E-06	4·23E-07	2·28E-06	1·45E-06			7·46E-06	7·46E-06
2×10^8	3·16E-06	2·39E-05	2·69E-06	6·52E-06			1·40E-07	2·39E-05
2×10^8	2·87E-06	2·14E-05	1·63E-06	1·39E-06			2·40E-06	2·14E-05

Dose-Escalation Module

Ogawa OSP IgM (RAU)								
**Dose (CFU)**	**D1**	**D7**	**D15**	**D29**	**D57**	**D85**	**D180**	**max**
10^5		8·94E-06		8·31E-06				8·94E-06
10^5		8·01E-06		5·27E-05				5·27E-05
10^5								
10^6		1·03E-01	7·42E-06	2·84E-07	4·55E-06	1·73E-06		1·03E-01
10^6	7·88E-08	1·61E-04				1·27E-07		1·61E-04
10^6		8·79E-03	2·95E-03	3·23E-06	1·14E-06	1·54E-07		8·79E-03
10^7		4·71E-03	9·45E-05	4·07E-05	8·80E-06	2·04E-06	1·05E-06	4·71E-03
10^7	5·88E-08	9·15E-03	6·99E-06	1·31E-06	3·23E-06		2·53E-07	9·15E-03
10^7		1·76E-04	1·07E-05				3·42E-07	1·76E-04
10^8	5·88E-08	3·29E-04	1·25E-05	2·96E-06	1·27E-07	8·20E-07		3·29E-04
10^8	1·37E-06	8·91E-04	2·23E-05	1·23E-05	6·22E-07	5·46E-06	3·11E-07	8·91E-04
10^8		2·86E-04	2·49E-07	7·88E-08				2·86E-04
10^9		2·99E-03	8·51E-06	1·51E-05	1·71E-06		9·34E-06	2·99E-03
10^9		2·70E-06	6·09E-06	4·06E-05	4·89E-06	5·67E-06		4·06E-05
10^9		1·93E-04	5·31E-06		4·74E-07	1·71E-06	4·32E-06	1·93E-04
10^{10}		1·74E-04	4·07E-07					1·74E-04
10^{10}		2·40E-03	8·05E-06				5·25E-06	2·40E-03
10^{10}		3·09E-02	1·30E-05	1·41E-05	1·90E-05	2·57E-06	1·25E-07	3·09E-02

Dose-Expansion Module

Ogawa OSP IgM (RAU)								
**Dose (CFU)**	**D1**	**D7**	**D15**	**D29**	**D57**	**D85**	**D180**	**max**
0								
0							5·06E-07	5·06E-07
0	2·79E-06			1·71E-06				2·79E-06
0	1·05E-07		5·06E-07					5·06E-07
0	1·26E-06	6·39E-07						1·26E-06
0			1·63E-06					1·63E-06
0		2·02E-06						2·02E-06
2×10^7		1·76E-03					1·51E-05	1·76E-03
2×10^7		9·57E-01	6·64E-04				5·24E-04	9·57E-01
2×10^7	2·39E-06	1·54E-02		8·31E-06				1·54E-02
2×10^7	1·05E-07	1·03E-05	5·06E-07					1·03E-05
2×10^7		1·75E-03	2·22E-05	2·59E-06				1·75E-03
2×10^7	7·83E-07	5·25E-03	4·18E-05	1·50E-05				5·25E-03
2×10^7	1·44E-06	1·43E-04	3·21E-06	4·99E-06				1·43E-04
2×10^7	1·81E-07	1·81E-03	8·67E-06					1·81E-03
2×10^7	1·01E-06	5·80E-06		1·81E-07				5·80E-06
2×10^7	4·86E-08	1·23E-03	2·86E-04	1·53E-06				1·23E-03
2×10^7		9·87E-04	2·68E-05					9·87E-04
2×10^7		2·24E-04	3·66E-05	8·47E-06				2·24E-04
2×10^7		7·81E-05		9·66E-08				7·81E-05
2×10^7		1·56E-04	2·22E-06				2·02E-06	1·56E-04
2×10^8		2·07E-02	3·50E-04	3·57E-03				2·07E-02
2×10^8	5·09E-05	2·41E-02		6·81E-05				2·41E-02
2×10^8	1·05E-07	1·34E-03	2·75E-07	1·01E-06				1·34E-03
2×10^8	4·43E-07	7·74E-03	2·42E-05	6·15E-06				7·74E-03
2×10^8	3·28E-07	7·82E-04						7·82E-04
2×10^8			2·26E-05					2·26E-05
2×10^8	2·75E-07	1·48E-04	1·45E-05	4·86E-08				1·48E-04
2×10^8		2·18E-04	1·62E-06	2·75E-07				2·18E-04
2×10^8	7·43E-08	2·15E-01	5·79E-04	3·05E-05			5·55E-05	2·15E-01
2×10^8		1·58E-02	2·51E-05	5·85E-05				1·58E-02
2×10^8		3·82E-03	8·24E-05	1·63E-06				3·82E-03
2×10^8		2·42E-04	1·54E-05					2·42E-04
2×10^8								

Dose-Escalation Module

Ogawa OSP IgG (RAU)								
Dose (CFU)	D1	D7	D15	D29	D57	D85	D180	max
10^5		4·63E-07		2·59E-05			8·68E-07	2·59E-05
10^5								
10^5								
10^6	1·30E-06	2·02E-06	3·50E-06		8·50E-06	9·86E-06		9·86E-06
10^6	6·26E-07	4·24E-06	1·30E-06		2·76E-06		4·97E-06	4·97E-06
10^6		4·15E-03	2·02E-02	2·11E-05	1·12E-05	2·02E-06	7·28E-06	2·02E-02
10^7	4·24E-06	2·02E-06	6·26E-07	3·50E-06	9·92E-08	4·24E-06	1·45E-05	1·45E-05
10^7	9·92E-08	3·05E-05	2·02E-06	1·05E-05	1·30E-06	1·30E-06		3·05E-05
10^7		1·30E-06		6·26E-07	4·97E-06	9·86E-06	4·93E-06	9·86E-06
10^8	3·50E-06		9·92E-08	3·50E-06	2·02E-06			3·50E-06
10^8	5·69E-06	1·19E-05	2·02E-06	2·02E-06		4·34E-06	1·33E-06	1·19E-05
10^8	4·97E-06	3·50E-06	6·26E-07	1·30E-06	5·53E-06	6·11E-06	1·33E-06	6·11E-06
10^9		3·50E-06	2·76E-06		1·92E-06		1·33E-06	3·50E-06
10^9	9·92E-08			8·42E-06	4·93E-06	6·07E-05		6·07E-05
10^9	1·33E-06				3·74E-06		7·47E-07	3·74E-06
10^{10}	1·92E-06	6·93E-03		1·29E-05				6·93E-03
10^{10}		3·13E-06	1·92E-06			7·47E-07		3·13E-06
10^{10}	4·34E-06	2·53E-06	1·33E-06			2·31E-07	2·53E-06	4·34E-06

Dose-Expansion Module

Ogawa OSP IgG (RAU)								
Dose (CFU)	D1	D7	D15	D29	D57	D85	D180	max
0								
0		1·08E-05					2·26E-06	1·08E-05
0		7·71E-06		1·53E-06				7·71E-06
0	8·98E-06	9·29E-06		4·77E-06				9·29E-06
0	1·27E-05	8·36E-06	1·50E-05	8·67E-06				1·50E-05
0		9·89E-06						9·89E-06
0			1·10E-06					1·10E-06
2×10^7		3·74E-04					7·10E-04	7·10E-04
2×10^7		7·46E-02	4·72E-04					7·46E-02
2×10^7	4·34E-06	4·77E-06	3·88E-06	2·26E-06			5·18E-06	5·18E-06
2×10^7	8·67E-06	2·86E-06		2·86E-06				8·67E-06
2×10^7	1·30E-05	1·45E-05	2·86E-06	7·71E-06			1·10E-06	1·45E-05
2×10^7	8·36E-06	1·13E-05	7·71E-06					1·13E-05
2×10^7		8·98E-06	5·18E-06					8·98E-06
2×10^7	1·19E-05		8·36E-06					1·19E-05
2×10^7		7·71E-06		5·57E-06				7·71E-06
2×10^7		9·59E-06	7·71E-06	3·40E-06				9·59E-06
2×10^7		1·53E-06						1·53E-06
2×10^7		2·10E-06						2·10E-06
2×10^7			2·10E-06					2·10E-06
2×10^7	1·84E-06						1·84E-06	1·84E-06
2×10^8		5·20E-04	2·53E-04	3·53E-05				5·20E-04
2×10^8	6·40E-04	7·18E-05		3·26E-04			1·10E-06	6·40E-04
2×10^8	1·05E-05	1·08E-05	5·95E-06					1·08E-05
2×10^8		2·14E-05	6·68E-06	1·27E-05				2·14E-05
2×10^8		1·71E-04	4·34E-06	5·95E-06			1·84E-06	1·71E-04
2×10^8			7·37E-06					7·37E-06
2×10^8	3·40E-06	7·37E-06		1·82E-05				1·82E-05
2×10^8								
2×10^8		5·14E-05	7·03E-06				4·72E-05	5·14E-05
2×10^8		5·31E-05		4·63E-07				5·31E-05
2×10^8	2·91E-07	2·53E-06	3·27E-06	6·58E-07			9·16E-05	9·16E-05
2×10^8			6·58E-07	1·10E-06				1·10E-06
2×10^8				6·92E-06			1·10E-06	6·92E-06

Dose-Escalation Module

Ogawa OSP IgA (RAU)								
Dose (CFU)	D1	D7	D15	D29	D57	D85	D180	max
10^5	4·06E-06	7·70E-06	4·26E-06	3·27E-06			3·07E-06	7·70E-06
10^5	3·07E-06	2·66E-06	7·04E-06	8·53E-06				8·53E-06
10^5	5·62E-06	6·95E-06	5·04E-06	5·04E-06				6·95E-06
10^6	5·59E-06	6·64E-04	5·80E-06	9·82E-06	1·85E-05	4·10E-05		6·64E-04
10^6	1·88E-06	1·39E-03	3·49E-06	5·79E-06	6·89E-06	2·17E-07	4·66E-06	1·39E-03
10^6	6·16E-06	2·52E-03	6·08E-03	5·87E-06	8·96E-07	1·63E-06	4·27E-07	6·08E-03
10^7	4·22E-06	1·91E-06	1·47E-06	1·91E-06	2·76E-06	3·56E-05	3·51E-05	3·56E-05
10^7	2·94E-06	3·67E-02	1·63E-05	3·07E-05	4·27E-06	1·47E-06	3·54E-07	3·67E-02
10^7	5·79E-07	8·96E-07	1·25E-05	6·57E-07	3·33E-06	1·30E-06	1·88E-06	1·25E-05
10^8	5·85E-07	3·31E-06	2·30E-06	2·83E-06	1·87E-05	1·35E-06	2·84E-06	1·87E-05
10^8	4·51E-06	7·84E-04	6·16E-06	3·41E-06	1·10E-05	3·89E-06	3·57E-06	7·84E-04
10^8	2·85E-06	3·80E-05	5·87E-06	1·38E-05	2·69E-05	2·12E-06	6·57E-07	3·80E-05
10^9	4·04E-06	2·01E-05	2·83E-07	1·73E-05	3·96E-06	1·16E-05	2·29E-06	2·01E-05
10^9	6·38E-06	8·72E-06	2·79E-05	1·89E-04	1·63E-05	6·93E-05		1·89E-04
10^9	1·96E-06	2·45E-06	5·12E-06	1·71E-06	6·31E-06	2·61E-06	1·80E-06	6·31E-06
10^{10}	5·57E-06	2·34E-04	1·22E-06	2·17E-07		1·47E-06	2·21E-06	2·34E-04
10^{10}	5·02E-07	5·37E-05	3·09E-05			2·61E-06	1·09E-05	5·37E-05
10^{10}		6·15E-05	3·15E-05	7·41E-05	3·06E-05	5·03E-05	1·82E-04	1·82E-04

Dose-Expansion Module

Ogawa OSP IgA (RAU)								
Dose (CFU)	D1	D7	D15	D29	D57	D85	D180	max
0	2·53E-06		2·12E-06	1·63E-06	1·54E-07		9·84E-08	2·53E-06
0	1·23E-05	2·14E-06	1·15E-05	1·07E-05			1·36E-05	1·36E-05
0	1·88E-06	9·77E-07	1·06E-06	5·02E-07			4·58E-06	4·58E-06
0	2·05E-06	3·07E-06	5·52E-06	4·06E-06			5·33E-06	5·52E-06
0	2·14E-06	2·53E-06	4·95E-06	3·48E-07				4·95E-06
0	4·75E-06	2·05E-06	5·81E-06	4·26E-06			4·26E-06	5·81E-06
0	5·91E-06	4·26E-06	2·46E-06	2·56E-06			4·94E-06	5·91E-06
2×10^7	1·06E-06	1·80E-06	3·73E-06				2·12E-06	3·73E-06
2×10^7	3·25E-06	2·94E-05	3·65E-06	3·25E-06			3·25E-06	2·94E-05
2×10^7	2·14E-06	2·38E-04	2·93E-06	8·23E-06			1·37E-06	2·38E-04
2×10^7	3·48E-07	3·33E-06	3·48E-07	9·47E-06				9·47E-06
2×10^7	3·48E-07	4·91E-04	1·60E-05	9·47E-06			1·52E-05	4·91E-04
2×10^7	3·48E-07	9·59E-03	9·16E-05	8·86E-05			2·71E-06	9·59E-03
2×10^7	1·44E-06	6·93E-05	3·72E-05	3·47E-06				6·93E-05
2×10^7	2·86E-06	2·71E-03	2·76E-06	2·23E-05			1·23E-06	2·71E-03
2×10^7	3·73E-06	6·18E-06	6·59E-06	2·53E-06			6·54E-07	6·59E-06
2×10^7	1·03E-06	3·91E-04	2·84E-04	7·42E-06			4·84E-06	3·91E-04
2×10^7	3·48E-07	1·93E-03	5·16E-05	1·73E-05			3·63E-05	1·93E-03
2×10^7	4·06E-06	6·03E-05	8·71E-06	4·07E-05			7·32E-06	6·03E-05
2×10^7	3·27E-06	8·07E-06	1·04E-05	5·04E-06			4·26E-06	1·04E-05
2×10^7	2·66E-06	3·03E-05	1·25E-05	3·47E-06			2·26E-06	3·03E-05
2×10^8	5·79E-07	3·98E-05	8·46E-06	2·22E-05	1·30E-06		1·14E-06	3·98E-05
2×10^8	1·14E-06	6·09E-06	4·35E-06	8·16E-07			9·77E-07	6·09E-06
2×10^8	2·26E-06	7·14E-06	4·36E-06	9·26E-06			1·33E-05	1·33E-05
2×10^8	1·37E-06	1·00E-02	2·48E-05	2·93E-06				1·00E-02
2×10^8	5·36E-06	5·08E-04	5·77E-06	2·14E-06				5·08E-04
2×10^8			4·13E-06					4·13E-06
2×10^8	7·41E-06	1·84E-05	1·19E-05	9·05E-06				1·84E-05
2×10^8	3·57E-06	1·74E-05	3·07E-06	1·53E-05			1·47E-05	1·74E-05
2×10^8		8·95E-04	5·53E-05	3·33E-06			3·69E-05	8·95E-04
2×10^8	1·60E-06	1·32E-01	1·68E-04	4·79E-04			2·32E-06	1·32E-01
2×10^8	6·54E-07	5·74E-05	1·46E-07	6·56E-06				5·74E-05
2×10^8	2·57E-06	2·19E-05	7·48E-07	1·01E-05			1·48E-06	2·19E-05
2×10^8	7·14E-06	4·06E-06	4·06E-06	4·26E-06			4·94E-06	7·14E-06

Dose-Escalation Module

CT-B IgM (RAU)								
**Dose (CFU)**	**D1**	**D7**	**D15**	**D29**	**D57**	**D85**	**D180**	**max**
10^5	1·10E-04	4·13E-04	3·13E-06					4·13E-04
10^5				4·50E-03				4·50E-03
10^5	5·75E-05	1·19E-04	3·13E-06	9·17E-06				1·19E-04
10^6	2·73E-07	1·46E-03	1·25E-04					1·46E-03
10^6		3·60E-04		3·10E-05		1·20E-05		3·60E-04
10^6	4·18E-05	3·44E-03	8·79E-04	2·58E-04		1·20E-05		3·44E-03
10^7	5·19E-04	1·00E-03	1·40E-03	1·01E-04	3·60E-04	5·88E-04		1·40E-03
10^7		1·01E-04			7·66E-05		7·68E-06	1·01E-04
10^7		3·38E-04	5·31E-05		2·81E-04	1·65E-03	1·30E-05	1·65E-03
10^8		4·25E-04	1·20E-05			4·18E-05		4·25E-04
10^8	5·68E-03	2·34E-03	2·21E-02	1·62E-02	5·72E-03	1·36E-03	3·10E-05	2·21E-02
10^8	5·31E-05	2·09E-03	1·50E-04					2·09E-03
10^9	4·04E-04	1·64E-03	7·66E-05	4·57E-04			1·27E-03	1·64E-03
10^9		5·31E-05		4·71E-05				5·31E-05
10^9			8·96E-05					8·96E-05
10^{10}		2·93E-04						2·93E-04
10^{10}		3·10E-05						3·10E-05
10^{10}		8·99E-04				1·06E-04	6·17E-06	8·99E-04

Dose-Expansion Module

CT-B IgM (RAU)								
**Dose (CFU)**	**D1**	**D7**	**D15**	**D29**	**D57**	**D85**	**D180**	**max**
0							1·59E-02	1·59E-02
0	7·27E-05	1·91E-03	4·36E-03	2·78E-05				4·36E-03
0	2·75E-04	5·26E-06	7·82E-05					2·75E-04
0				8·29E-06			1·17E-05	1·17E-05
0		1·18E-04						1·18E-04
0								
0	1·44E-05	8·72E-05		3·04E-05			5·75E-05	8·72E-05
2×10^7	1·05E-03	4·25E-04		7·34E-03			3·50E-03	7·34E-03
2×10^7								
2×10^7								
2×10^7	6·21E-05	8·93E-05	2·78E-05	1·54E-04			2·31E-04	2·31E-04
2×10^7	1·54E-05	1·01E-03	1·73E-04					1·01E-03
2×10^7	1·12E-04			4·18E-05				1·12E-04
2×10^7	3·70E-05		1·36E-04					1·36E-04
2×10^7		2·04E-04		3·08E-04				3·08E-04
2×10^7			5·17E-05	5·17E-05				5·17E-05
2×10^7	3·70E-05	1·24E-04		4·18E-05			5·75E-05	1·24E-04
2×10^7	2·35E-05	4·72E-03		2·70E-05				4·72E-03
2×10^7		1·17E-03	1·29E-03	9·18E-05				1·29E-03
2×10^7	7·12E-07		1·98E-04	3·13E-06			1·73E-05	1·98E-04
2×10^7		3·13E-06						3·13E-06
2×10^8	1·41E-03						1·04E-02	1·04E-02
2×10^8		7·84E-03					1·51E-04	7·84E-03
2×10^8		2·69E-04		2·49E-04				2·69E-04
2×10^8		4·37E-04	1·60E-04					4·37E-04
2×10^8		6·54E-04	6·21E-05	8·29E-06			2·50E-04	6·54E-04
2×10^8			3·24E-05					3·24E-05
2×10^8		6·19E-04		1·91E-04			7·19E-05	6·19E-04
2×10^8		1·54E-05	5·17E-05					5·17E-05
2×10^8		2·69E-04	2·95E-04	1·14E-03			5·14E-04	1·14E-03
2×10^8		3·48E-04						3·48E-04
2×10^8	3·13E-06	2·53E-04	1·42E-04					2·53E-04
2×10^8								
2×10^8			7·00E-05					7·00E-05

Dose-Escalation Module

CT-B IgG (RAU)								
**Dose (CFU)**	**D1**	**D7**	**D15**	**D29**	**D57**	**D85**	**D180**	**max**
10^5		5·43E-05	8·19E-04	1·68E-04				8·19E-04
10^5				2·49E-05				2·49E-05
10^5		3·60E-05	6·83E-05	8·26E-06				6·83E-05
10^6	8·38E-08	1·96E-03	1·50E-06	1·57E-07		1·57E-07		1·96E-03
10^6		3·46E-03	1·57E-07		8·38E-08			3·46E-03
10^6		2·54E-02	7·72E-04	2·21E-05	7·43E-06	4·68E-07		2·54E-02
10^7		3·26E-05	3·97E-05	1·08E-05	2·47E-07			3·97E-05
10^7		3·27E-06	3·35E-05	7·62E-06	1·33E-06			3·35E-05
10^7		4·42E-05				2·00E-06		4·42E-05
10^8		4·20E-06	8·64E-05	5·53E-06	1·57E-07			8·64E-05
10^8		1·34E-03	8·52E-05	4·68E-07		3·63E-06		1·34E-03
10^8		7·48E-04	3·02E-05	4·96E-06				7·48E-04
10^9		3·33E-03	1·59E-05	9·32E-06		5·68E-07	2·06E-06	3·33E-03
10^9								
10^9		3·75E-06	1·27E-05		5·91E-06			1·27E-05
10^{10}		5·73E-05	2·37E-07					5·73E-05
10^{10}		6·86E-04	1·98E-05					6·86E-04
10^{10}		4·75E-03	1·26E-04	9·00E-06	5·30E-06	2·38E-06		4·75E-03

Dose-Expansion Module

CT-B IgG (RAU)								
**Dose (CFU)**	**D1**	**D7**	**D15**	**D29**	**D57**	**D85**	**D180**	**max**
0								
0	1·65E-06	2·83E-06	3·37E-07	7·85E-07			3·28E-06	3·28E-06
0			3·06E-06	2·27E-07				3·06E-06
0								
0	3·37E-07	2·09E-06						2·09E-06
0	2·80E-07							2·80E-07
0								
2×10^7		4·85E-02		1·10E-04			1·38E-04	4·85E-02
2×10^7								
2×10^7			1·50E-06	5·20E-07				1·50E-06
2×10^7		2·03E-04	4·61E-05	7·17E-07			1·58E-05	2·03E-04
2×10^7		2·67E-05	1·13E-05	1·87E-06				2·67E-05
2×10^7	5·85E-07	6·79E-05	5·96E-05					6·79E-05
2×10^7		3·96E-07	3·63E-05	2·61E-05				3·63E-05
2×10^7		1·22E-03	1·35E-05	1·22E-05				1·22E-03
2×10^7			2·91E-06	8·88E-06				8·88E-06
2×10^7		1·43E-05	2·31E-06					1·43E-05
2×10^7	5·95E-06	1·72E-02	5·55E-05	1·99E-05			8·68E-06	1·72E-02
2×10^7		4·69E-04	1·95E-04	9·07E-06				4·69E-04
2×10^7								
2×10^7		9·20E-05						9·20E-05
2×10^8	3·03E-04						1·31E-04	3·03E-04
2×10^8		1·74E-02						1·74E-02
2×10^8	5·85E-07	1·10E-03	3·31E-05					1·10E-03
2×10^8	1·28E-06	6·44E-03	2·31E-05	5·20E-07			1·09E-06	6·44E-03
2×10^8		3·34E-02	1·60E-05	2·39E-06			1·77E-05	3·34E-02
2×10^8			1·95E-04					1·95E-04
2×10^8	1·43E-06	5·76E-03	1·17E-04	9·93E-06				5·76E-03
2×10^8		5·73E-06	1·10E-04	8·14E-06				1·10E-04
2×10^8			5·88E-06	7·30E-05			4·85E-05	7·30E-05
2×10^8	2·54E-07	7·28E-03	5·05E-05	5·81E-05				7·28E-03
2×10^8			1·86E-06	4·95E-07				1·86E-06
2×10^8								
2×10^8		2·73E-05		7·79E-07				2·73E-05

Dose-Escalation Module

CT-B IgA (RAU)								
Dose (CFU)	D1	D7	D15	D29	D57	D85	D180	max
10^5	7·40E-05	1·61E-04	4·56E-03	1·78E-04			3·32E-05	4·56E-03
10^5	2·96E-05	1·43E-06	1·33E-05	6·85E-05				6·85E-05
10^5	2·45E-06	2·56E-05	2·07E-04	1·69E-05				2·07E-04
10^6	8·65E-06	6·30E-02	6·25E-05	1·68E-05	2·29E-05	6·30E-06		6·30E-02
10^6	4·12E-05	4·13E-01	4·17E-04	5·17E-05	1·07E-05	2·71E-06	6·13E-06	4·13E-01
10^6	6·36E-06	1·65E-01	4·11E-03	1·26E-04	5·36E-05	2·08E-05	2·50E-06	1·65E-01
10^7	1·79E-05	6·70E-04	6·63E-04	2·12E-04	1·12E-05	6·69E-06	3·78E-06	6·70E-04
10^7	7·52E-06	2·03E-04	5·70E-04	1·55E-05	8·76E-06	2·05E-06	1·04E-06	5·70E-04
10^7	4·06E-06	4·46E-04	1·23E-05	6·27E-06	1·92E-06	3·52E-06	3·73E-06	4·46E-04
10^8	5·02E-06	1·24E-03	4·44E-03	1·01E-04	2·92E-05	1·48E-05	1·29E-06	4·44E-03
10^8	3·22E-05	1·86E-03	3·19E-04	1·23E-05	2·17E-05	4·03E-05	4·07E-06	1·86E-03
10^8	7·93E-06	6·02E-02	1·28E-03	3·13E-04	4·66E-05	2·15E-05		6·02E-02
10^9	8·76E-06	2·91E-02	5·49E-05	1·01E-05	5·64E-06	6·37E-06	1·92E-06	2·91E-02
10^9	1·09E-05	2·42E-05	1·38E-05	7·72E-06	7·14E-06	1·21E-05		2·42E-05
10^9	9·70E-07	1·12E-04	2·45E-04	1·40E-06	1·74E-05	2·44E-06	1·07E-06	2·45E-04
10^{10}	6·68E-05	1·41E-04	2·64E-05	1·72E-05		5·02E-06	4·18E-06	1·41E-04
10^{10}	1·60E-06	1·71E-02	1·06E-04			9·02E-07	5·35E-07	1·71E-02
10^{10}		3·65E-03	1·27E-04	4·77E-05	1·55E-05	1·24E-05	3·03E-06	3·65E-03

Dose-Expansion Module

CT-B IgA (RAU)								
Dose (CFU)	D1	D7	D15	D29	D57	D85	D180	max
0	1·01E-04	3·71E-05	1·14E-05	6·15E-05	7·91E-06		5·56E-06	1·01E-04
0	3·42E-05	1·47E-05	4·27E-05	2·40E-05			4·70E-05	4·70E-05
0	1·31E-06	2·08E-06	1·68E-06	5·78E-06			1·28E-05	1·28E-05
0	1·91E-05	5·33E-05		9·63E-07			2·33E-05	5·33E-05
0	5·48E-05	3·92E-07	7·30E-06	6·79E-06			1·17E-05	5·48E-05
0	6·55E-07	5·14E-08	1·84E-06	2·05E-06			1·60E-05	1·60E-05
0	4·17E-06	6·58E-06					2·94E-05	2·94E-05
2×10^7	1·97E-06	2·69E-04	7·95E-05				4·60E-06	2·69E-04
2×10^7	6·56E-06	9·23E-04	5·80E-05	3·16E-05			7·08E-06	9·23E-04
2×10^7	4·79E-06	1·15E-05	1·68E-06	1·86E-07			1·36E-05	1·36E-05
2×10^7	4·79E-06	3·07E-04	2·30E-04	1·91E-05			3·07E-05	3·07E-04
2×10^7	2·87E-06	4·06E-04	7·86E-05	1·58E-04			1·75E-06	4·06E-04
2×10^7	3·32E-06	1·46E-03	5·23E-04	1·32E-05				1·46E-03
2×10^7	7·81E-06	2·12E-05	1·14E-04	1·96E-05				1·14E-04
2×10^7		2·63E-02	3·45E-04	6·79E-06			2·25E-07	2·63E-02
2×10^7	2·00E-05	1·52E-05	1·02E-04	1·70E-04			1·53E-05	1·70E-04
2×10^7	1·05E-05	1·35E-05	1·14E-05	6·89E-05			9·83E-07	6·89E-05
2×10^7	6·79E-06	3·17E-02	1·79E-04	3·46E-05			1·18E-04	3·17E-02
2×10^7	1·87E-05	8·05E-03	1·46E-03	7·99E-05			1·03E-05	8·05E-03
2×10^7	5·39E-07	1·70E-05	9·62E-06	7·46E-06			5·29E-06	1·70E-05
2×10^7		1·03E-04	3·78E-05	1·56E-08			7·53E-07	1·03E-04
2×10^8		8·05E-05	1·22E-04	4·68E-05	7·46E-06		4·25E-06	1·22E-04
2×10^8	7·40E-06	6·81E-04	3·34E-04	1·86E-04			5·02E-06	6·81E-04
2×10^8	1·42E-05	3·49E-03	6·67E-05	1·25E-04			1·14E-05	3·49E-03
2×10^8	7·30E-06	6·24E-02	1·03E-04	4·54E-05			1·69E-05	6·24E-02
2×10^8	5·14E-07	4·27E-02	8·09E-06	3·19E-05			3·06E-05	4·27E-02
2×10^8			8·17E-04					8·17E-04
2×10^8	1·80E-05	1·13E-02	1·92E-04	1·69E-05			8·33E-06	1·13E-02
2×10^8	9·38E-06	2·93E-06	3·55E-04	1·04E-04			2·37E-05	3·55E-04
2×10^8	2·02E-06	1·39E-04	2·29E-03	1·24E-03			1·40E-05	2·29E-03
2×10^8	9·23E-06	9·34E-01	1·14E-04	2·17E-04			5·06E-06	9·34E-01
2×10^8	5·39E-07	2·58E-06	8·38E-06	1·16E-05			1·73E-04	1·73E-04
2×10^8	8·48E-06	1·52E-05	2·43E-05	2·21E-05			2·25E-07	2·43E-05
2×10^8		3·61E-06	5·92E-06	5·23E-06				5·92E-06

Dose-Escalation Module

TcpA IgM (RAU)								
Dose (CFU)	D1	D7	D15	D29	D57	D85	D180	max
10^5	1·40E-06	2·49E-06	2·74E-05	6·54E-05			2·49E-06	6·54E-05
10^5		1·68E-03	5·57E-04					1·68E-03
10^5	1·40E-06	1·23E-03	2·44E-04	1·63E-05				1·23E-03
10^6		1·67E-03	1·58E-04		3·26E-04			1·67E-03
10^6		1·92E-03		2·46E-04				1·92E-03
10^6		9·44E-04	5·67E-04					9·44E-04
10^7		3·84E-04					5·16E-05	3·84E-04
10^7						1·47E-04		1·47E-04
10^7		1·44E-03	2·39E-04			1·09E-03		1·44E-03
10^8	1·05E-04							1·05E-04
10^8	1·15E-02	1·67E-03	5·36E-01	3·18E-01	1·03E-02	5·73E-04	1·64E-04	5·36E-01
10^8		7·86E-03	1·47E-04				2·66E-06	7·86E-03
10^9	9·48E-04	1·71E-03	2·54E-04	1·56E-03	8·29E-05	5·94E-05	1·41E-03	1·71E-03
10^9				1·77E-05				1·77E-05
10^9		1·10E-05			3·24E-05	2·12E-05		3·24E-05
10^{10}		1·31E-03	1·41E-04	7·51E-05		2·66E-06		1·31E-03
10^{10}		2·27E-04					4·38E-05	2·27E-04
10^{10}		3·10E-03		3·24E-05		1·45E-04		3·10E-03

Dose-Expansion Module

TcpA IgM (RAU)								
Dose (CFU)	D1	D7	D15	D29	D57	D85	D180	max
0		3·20E-03			5·51E-03			5·51E-03
0	2·24E-05	7·02E-06	3·30E-04	1·23E-05			6·16E-04	6·16E-04
0	6·36E-05	3·06E-05	9·48E-06	1·22E-06			1·42E-07	6·36E-05
0	2·44E-05	2·44E-05		5·79E-05			4·47E-04	4·47E-04
0		6·43E-08	1·76E-06	3·97E-05			2·49E-06	3·97E-05
0	1·54E-05			2·74E-05				2·74E-05
0	7·32E-05	1·61E-04	1·02E-04	1·28E-04			5·46E-06	1·61E-04
2×10^7		1·14E-02		5·51E-03				1·14E-02
2×10^7		6·06E-02					9·55E-03	6·06E-02
2×10^7	7·80E-07	3·58E-03	5·51E-05	4·90E-06			1·01E-04	3·58E-03
2×10^7	4·42E-07	1·08E-04	7·24E-05	3·14E-06			7·32E-05	1·08E-04
2×10^7	1·76E-06	1·76E-03	7·80E-07	6·07E-05			7·32E-05	1·76E-03
2×10^7	2·06E-05	1·01E-04	3·14E-06	3·06E-05				1·01E-04
2×10^7		1·70E-05	4·72E-05					4·72E-05
2×10^7	2·40E-06	6·07E-05	1·70E-05				6·17E-05	6·17E-05
2×10^7		1·38E-05	4·90E-06	8·49E-05			1·40E-06	8·49E-05
2×10^7	3·97E-05	8·99E-04	1·88E-05	3·06E-05			1·15E-05	8·99E-04
2×10^7		4·91E-03	3·02E-04	2·40E-04			1·38E-05	4·91E-03
2×10^7	4·02E-05	2·97E-04	2·16E-05	5·46E-06			2·49E-06	2·97E-04
2×10^7	3·85E-06	3·09E-02	1·70E-04	9·27E-06				3·09E-02
2×10^7	1·63E-05	5·81E-04	3·04E-05	5·07E-05			7·27E-06	5·81E-04
2×10^8			4·01E-03					4·01E-03
2×10^8	7·59E-03		6·01E-02					6·01E-02
2×10^8	4·42E-07	2·62E-04	1·76E-06	1·54E-05			4·02E-05	2·62E-04
2×10^8	6·07E-05	3·74E-05	8·21E-06	9·81E-05			1·63E-05	9·81E-05
2×10^8	7·80E-07	1·84E-03	4·46E-05	1·54E-05			1·19E-04	1·84E-03
2×10^8			9·81E-05					9·81E-05
2×10^8		2·24E-05	1·01E-04	9·48E-06			5·93E-05	1·01E-04
2×10^8	5·24E-05	2·40E-06	1·76E-06	2·24E-05				5·24E-05
2×10^8		3·06E-05	1·38E-05	7·80E-07			7·85E-04	7·85E-04
2×10^8		4·70E-05	1·42E-07				3·36E-05	4·70E-05
2×10^8		4·92E-04	6·66E-05				8·53E-05	4·92E-04
2×10^8	1·42E-07		1·89E-05	6·01E-07			2·16E-05	2·16E-05
2×10^8	4·70E-05	4·36E-05	2·49E-06	3·69E-05			1·40E-06	4·70E-05

Dose-Escalation Module

	TcpA IgG (RAU)							
Dose (CFU)	D1	D7	D15	D29	D57	D85	D180	max
10^5	2·71E-05	3·76E-05	1·41E-05	1·57E-05			1·33E-05	3·76E-05
10^5	2·95E-05		3·20E-05	4·40E-05				4·40E-05
10^5	5·68E-06	8·64E-06	1·49E-05	1·02E-05				1·49E-05
10^6		1·39E-05						1·39E-05
10^6	3·61E-06	5·29E-05						5·29E-05
10^6		6·79E-06		1·58E-05	1·21E-05	1·06E-06		1·58E-05
10^7	1·21E-05			8·51E-06	2·22E-06	3·61E-06	8·20E-06	1·21E-05
10^7				8·51E-06	1·06E-06		1·76E-06	8·51E-06
10^7				3·61E-06		1·06E-06	4·84E-07	3·61E-06
10^8	2·22E-06	1·06E-06	2·22E-06				9·41E-08	2·22E-06
10^8		1·21E-05		1·06E-06		4·84E-07	4·84E-07	1·21E-05
10^8	1·95E-05			3·08E-05	1·93E-05	9·41E-08	9·24E-06	3·08E-05
10^9		2·32E-05		1·06E-06	5·21E-06	9·41E-08	1·06E-06	2·32E-05
10^9			4·28E-06	2·54E-06	1·06E-06	3·38E-06		4·28E-06
10^9		5·21E-06	1·25E-05	1·06E-06	4·28E-06	4·84E-07	1·06E-06	1·25E-05
10^{10}	8·20E-06	3·38E-06	2·54E-06	9·41E-08		5·21E-06		8·20E-06
10^{10}	4·84E-07		1·06E-06				4·28E-06	4·28E-06
10^{10}		3·38E-06	1·76E-06	1·81E-05	6·18E-06	7·18E-06	1·06E-06	1·81E-05

Dose-Expansion Module

	TcpA IgG (RAU)							
Dose (CFU)	D1	D7	D15	D29	D57	D85	D180	max
0					2·35E-03			2·35E-03
0	1·63E-05		4·05E-06	1·29E-05				1·63E-05
0				1·28E-07			4·56E-05	4·56E-05
0	1·28E-07	1·28E-07	2·09E-05				1·81E-06	2·09E-05
0	1·55E-05						1·82E-05	1·82E-05
0		1·12E-06	1·33E-05	4·28E-06			1·02E-05	1·33E-05
0	4·28E-06	2·63E-05	1·02E-05	2·30E-05			1·17E-05	2·63E-05
2×10^7				1·81E-03				1·81E-03
2×10^7		3·76E-03					1·54E-03	3·76E-03
2×10^7		1·50E-06					6·66E-06	6·66E-06
2×10^7	5·30E-06	1·81E-05	1·28E-07	1·28E-07			1·98E-05	1·98E-05
2×10^7	1·12E-06	2·37E-05	2·85E-07				8·64E-06	2·37E-05
2×10^7		1·25E-06	1·96E-07	4·92E-07			3·04E-07	1·25E-06
2×10^7		4·66E-06		1·29E-05				1·29E-05
2×10^7		1·01E-06	5·60E-07				2·79E-05	2·79E-05
2×10^7	1·21E-05	1·29E-05					1·49E-05	1·49E-05
2×10^7	2·92E-06	2·92E-06	1·12E-06	5·05E-07			2·14E-05	2·14E-05
2×10^7			2·06E-05	5·04E-05			1·49E-05	5·04E-05
2×10^7	3·36E-05	2·38E-05	1·65E-05	1·17E-05				3·36E-05
2×10^7	3·44E-05	2·98E-06		9·42E-06			2·71E-05	3·44E-05
2×10^7	2·22E-05	2·06E-05	8·64E-06				1·49E-05	2·22E-05
2×10^8			2·57E-03					2·57E-03
2×10^8	6·05E-04						3·28E-05	6·05E-04
2×10^8		4·05E-06	5·05E-07	1·12E-06			1·82E-05	1·82E-05
2×10^8		4·66E-06					3·92E-05	3·92E-05
2×10^8		5·48E-05	3·47E-06				2·55E-05	5·48E-05
2×10^8								
2×10^8		4·66E-06					1·73E-05	1·73E-05
2×10^8		7·38E-06	4·66E-06	2·92E-06			1·65E-05	1·65E-05
2×10^8		1·93E-06		8·11E-06			1·73E-05	1·73E-05
2×10^8	2·55E-05	1·10E-05	1·33E-05	1·98E-05			1·17E-05	2·55E-05
2×10^8	2·47E-05	1·65E-05	2·63E-05	1·17E-05			2·14E-05	2·63E-05
2×10^8	7·87E-06	1·73E-05	1·33E-05	9·42E-06			1·65E-05	1·73E-05
2×10^8	1·33E-05	1·96E-04	5·27E-05	1·49E-05			5·68E-06	1·96E-04

Dose-Escalation Module

TcpA IgA (RAU)								
**Dose (CFU)**	**D1**	**D7**	**D15**	**D29**	**D57**	**D85**	**D180**	**max**
10^5	2·34E-05	5·28E-05	6·40E-05	1·40E-04			2·90E-05	1·40E-04
10^5	2·03E-06	7·72E-06	7·03E-06	6·37E-06				7·72E-06
10^5	1·76E-05	1·99E-05	4·74E-05	5·62E-05				5·62E-05
10^6	5·21E-05	7·39E-05	2·60E-05	6·28E-05	1·19E-05	1·95E-05		7·39E-05
10^6	8·15E-06	2·86E-04	6·55E-06	1·21E-05	2·04E-05	6·30E-05	7·00E-06	2·86E-04
10^6	2·86E-05	7·56E-05	1·93E-05	3·85E-05	2·25E-06	6·33E-06	2·44E-06	7·56E-05
10^7	2·16E-05	2·12E-04	3·80E-05	3·96E-05	1·00E-05	9·54E-06	3·77E-05	2·12E-04
10^7	1·31E-05	2·79E-05	1·31E-05	5·21E-06	1·16E-05	8·84E-06	3·48E-06	2·79E-05
10^7	1·61E-05	1·50E-05	1·19E-05	1·88E-05	1·45E-05	9·77E-06	9·28E-06	1·88E-05
10^8	5·65E-06	1·45E-05	5·95E-05	1·19E-05	3·75E-05	1·02E-05	9·66E-06	5·95E-05
10^8	6·52E-05	1·73E-04	3·65E-05	3·77E-05	2·88E-05	2·12E-05	7·93E-06	1·73E-04
10^8	1·85E-05	1·90E-04	5·76E-05	1·45E-04	1·66E-04	1·41E-05	1·47E-05	1·90E-04
10^9	1·42E-05	6·16E-05	9·77E-06	2·21E-05	1·15E-05	1·81E-05	5·48E-06	6·16E-05
10^9	4·40E-05	2·52E-05	1·64E-05	6·16E-06	1·23E-06	5·70E-06		4·40E-05
10^9	2·09E-06	2·56E-05	2·10E-05	2·62E-06	1·68E-06	4·19E-06	8·42E-06	2·56E-05
10^{10}	7·57E-06	2·77E-05	2·19E-04	7·59E-05		1·45E-06	1·71E-07	2·19E-04
10^{10}	2·03E-07	2·37E-05	1·52E-06			7·03E-08	1·60E-06	2·37E-05
10^{10}	4·01E-07	1·11E-04	2·99E-06	2·12E-05	5·04E-06	6·27E-06	8·31E-07	1·11E-04

Dose-Expansion Module

TcpA IgA (RAU)								
**Dose (CFU)**	**D1**	**D7**	**D15**	**D29**	**D57**	**D85**	**D180**	**max**
0	1·40E-05	1·18E-05	1·30E-05	1·98E-05	3·63E-06		9·63E-06	1·98E-05
0	4·46E-05	4·02E-05	7·43E-05	8·07E-05			8·71E-05	8·71E-05
0	1·35E-05	7·38E-06	6·68E-06	1·79E-05			2·88E-05	2·88E-05
0	1·99E-05	3·30E-05	2·75E-05	3·54E-05			3·98E-05	3·98E-05
0	2·96E-05	2·21E-05	3·30E-05	2·81E-05			3·30E-05	3·30E-05
0	3·57E-05	1·96E-05	1·33E-05	1·30E-05			2·40E-05	3·57E-05
0	4·25E-05	2·56E-05	1·53E-05	3·48E-05			2·18E-05	4·25E-05
$2×10^7$	8·77E-06	8·07E-05	1·49E-05	3·63E-06			7·55E-06	8·07E-05
$2×10^7$	2·78E-05	2·02E-05	1·05E-05	3·22E-05			2·62E-05	3·22E-05
$2×10^7$	2·14E-05	1·21E-04	9·21E-05	2·48E-05			3·88E-05	1·21E-04
$2×10^7$	3·43E-05	4·74E-05	1·40E-05	7·30E-05			1·19E-05	7·30E-05
$2×10^7$	2·80E-05	4·86E-04	8·07E-05	1·85E-04			6·37E-06	4·86E-04
$2×10^7$	2·48E-05	1·25E-04	4·72E-06	1·60E-05				1·25E-04
$2×10^7$	1·40E-05	2·34E-04	3·73E-05	2·14E-05				2·34E-04
$2×10^7$	2·31E-05	3·05E-05	2·31E-05	2·87E-05			2·25E-05	3·05E-05
$2×10^7$	6·73E-05	3·12E-05	5·31E-05	2·09E-05			1·70E-05	6·73E-05
$2×10^7$	1·78E-05	2·14E-05	1·20E-05	1·96E-05				2·14E-05
$2×10^7$	3·75E-05	9·05E-05	4·01E-05	4·37E-05			1·36E-04	1·36E-04
$2×10^7$	3·42E-05	4·83E-05	3·48E-05	6·54E-05			1·33E-05	6·54E-05
$2×10^7$	5·81E-06	7·03E-05	5·62E-05	3·05E-05			2·47E-05	7·03E-05
$2×10^7$	2·56E-05	5·20E-05	2·87E-05	2·81E-05			3·18E-05	5·20E-05
$2×10^8$	9·37E-07	6·33E-06	1·13E-05	2·85E-05	1·55E-05		3·12E-05	3·12E-05
$2×10^8$	1·30E-05	4·24E-05	2·68E-05	9·97E-06			1·37E-05	4·24E-05
$2×10^8$	1·20E-05	3·12E-05		2·48E-05			3·96E-05	3·96E-05
$2×10^8$	2·96E-05	4·60E-05	3·43E-05	5·98E-05			2·46E-05	5·98E-05
$2×10^8$	1·78E-05	3·10E-04	2·64E-05				3·00E-07	3·10E-04
$2×10^8$			3·42E-05					3·42E-05
$2×10^8$	5·71E-05	2·08E-04	1·02E-04	6·38E-05			8·10E-08	2·08E-04
$2×10^8$	1·53E-05	5·14E-05	2·37E-05	4·60E-05			8·37E-05	8·37E-05
$2×10^8$	2·18E-05	9·49E-05	3·78E-05	3·57E-05			2·18E-05	9·49E-05
$2×10^8$	7·03E-06	1·35E-04	1·54E-05	1·40E-04			2·25E-05	1·40E-04
$2×10^8$	3·12E-05	3·69E-05	5·34E-05	4·04E-05			1·06E-05	5·34E-05
$2×10^8$	2·52E-05	4·29E-05	1·65E-05	8·05E-05			1·40E-05	8·05E-05
$2×10^8$	5·11E-05	5·64E-03	3·48E-05	3·39E-05			1·23E-05	5·64E-03

Table S11: Geometric mean peak antibody responses (RAU) to Inaba, and Ogawa OSP, CT-B and TcpA in lymphocyte supernatant of vaccine and placebo recipients. PanChol group represents combined data of all vaccine recipients. Placebo: ≥4; PanChol: ≥41.

	IgM	IgG	IgA
**Placebo-geometric mean peak titer × 10 000 (95% CI)**			
**Inaba OSP**	0·05 (0·01-0·35)	0·25 (0·05-1·40)	0·02 (0·01-0·03)
**Ogawa OSP**	0·01 (0·01-0·03)	0·07 (0·03-0·19)	0·06 (0·04-0·09)
**CT-B**	3·60 (0·22-60·00)	0·02 (0·003-0·10)	0·36 (0·18-0·72)
**TcpA**	2·10 (0·37-12·00)	0·42 (0·08-2·30)	0·37 (0·25-0·57)
**PanChol-geometric mean peak titer × 10 000 (95% CI)**			
**Inaba OSP**	7·90 (2·90-22·00)	0·51 (0·28-0·90)	0·95 (0·43-2·10)
**Ogawa OSP**	10·00 (4·40-23·00)	0·22 (0·10-0·48)	0·99 (0·45-2·20)
**CT-B**	4·40 (2·50-7·80)	3·20 (1·40-7·50)	13·00 (5·30-30·00)
**TcpA**	6·20 (3·10-12·00)	0·26 (0·16-0·43)	0·81 (0·59-1·10)

Table S12: Geometric mean peak fold increase in antigen- and isotype-specific immune responses to Inaba, and Ogawa OSP, CT-B and TcpA in serum of vaccine (10^7 and 10^8 CFU of PanChol) and placebo recipients. Placebo:7; PanChol (10^7 CFU):17; PanChol (10^8 CFU):16.

	IgM	IgG	IgA
**Placebo-geometric mean peak fold increase (95% CI)**			
**Inaba OSP**	1·2 (1·1-1·4)	1·3 (1·0-1·6)	1·5 (1·2-1·9)
**Ogawa OSP**	1·4 (1·1-1·7)	1·4 (1·1-1·7)	1·3 (1·0-1·7)
**CT-B**	2·2 (1·1-4·4)	1·2 (1·0-1·6)	1·3 (1·0-1·8)
**TcpA**	2·6 (0·6-12·0)	1·2 (1·1-1·3)	1·2 (1·0-1·5)
**PanChol (10^7 CFU)-geometric mean peak fold increase (95% CI)**			
**Inaba OSP**	55·0 (25·0-121·0)	4·6 (2·1-10·0)	21·0 (7·4-59·0)
**Ogawa OSP**	33·0 (13·0-78·0)	5·7 (2·1-16·0)	13·0 (5·6-32·0)
**CT-B**	1·6 (1·2-2·2)	3·2 (1·9-5·5)	2·7 (1·6-4·4)
**TcpA**	2·1 (1·5-3·1)	1·9 (1·4-2·5)	1·6 (1·3-2·0)
**PanChol (10^8 CFU)-geometric mean peak fold increase (95% CI)**			
**Inaba OSP**	84·0 (31·0-227·0)	7·5 (3·0-19·0)	9·5 (4·2-21·0)
**Ogawa OSP**	48·0 (17·0-141·0)	9·1 (4·1-20·0)	9·1 (3·7-22·0)
**CT-B**	1·3 (1·1-1·5)	6·2 (3·3-11·0)	5·1 (2·4-11·0)
**TcpA**	1·7 (1·1-2·6)	2·0 (1·2-3·4)	2·1 (1·1-3·8)

Table S13: Geometric mean peak antibody responses (RAU) to Inaba, and Ogawa OSP, CT-B and TcpA in lymphocyte supernatant of vaccine (10^7 and 10^8 CFU of PanChol) and placebo recipients. Placebo: ≥4; PanChol (10^7 CFU): ≥14; PanChol (10^8 CFU): ≥15.

	IgM	IgG	IgA
**Placebo-geometric mean peak titer × 10 000 (95% CI)**			
**Inaba OSP**	0·05 (0·01-0·35)	0·25 (0·05-1·40)	0·02 (0·01-0·03)
**Ogawa OSP**	0·01 (0·01-0·03)	0·07 (0·03-0·19)	0·06 (0·04-0·09)
**CT-B**	3·60 (0·22-60·00)	0·02 (0·003-0·10)	0·36 (0·18-0·72)
**TcpA**	2·10 (0·37-12·00)	0·42 (0·08-2·30)	0·37 (0·25-0·57)
**PanChol (10^7 CFU)-geometric mean peak titer × 10 000 (95% CI)**			
**Inaba OSP**	8·20 (2·40-28·00)	0·55 (0·16-1·80)	1·10 (0·30-4·00)
**Ogawa OSP**	8·70 (2·00-38·00)	0·15 (0·04-0·59)	1·20 (0·31-5·00)
**CT-B**	3·20 (1·10-9·60)	1·10 (0·25-5·20)	4·60 (1·50-14·00)
**TcpA**	8·00 (2·60-25·00)	0·26 (0·10-0·72)	0·73 (0·46-1·20)
**PanChol (10^8 CFU)-geometric mean peak titer × 10 000 (95% CI)**			
**Inaba OSP**	8·80 (0·91-85·00)	0·50 (0·20-1·20)	0·62 (0·14-2·80)
**Ogawa OSP**	16·00 (4·00-61·00)	0·25 (0·09-0·68)	0·88 (0·19-4·10)
**CT-B**	6·30 (2·20-18·00)	5·90 (1·30-26·00)	20·00 (3·80-110·00)
**TcpA**	5·40 (1·20-25·00)	0·38 (0·15-0·99)	1·10 (0·55-2·10)

Table S14: Quantification of PanChol bacteria in fecal samples derived from vaccine and placebo recipients.
Dark blue: no sample; light blue: negative rectal swab; orange: positive rectal swab; D: day.

Dose-Escalation Module

	Abundace of PanChol (CFU/g)						
Dose (CFU)	D1	D1·2	D2	D3	D4	D5	Notes
10^4	9·00E+01	9·00E+01	9·00E+01	8·00E+02	9·00E+01	2·00E+03	
10^4	9·00E+01	9·00E+01	9·00E+01	9·00E+01	9·00E+01	9·00E+01	
10^4	9·00E+01	9·00E+01	9·00E+01	9·00E+01	9·00E+01	9·00E+01	
10^5	9·00E+01		9·00E+01		1·07E+07	2·50E+05	
10^5	9·00E+01	9·00E+01	9·00E+01	9·64E+06	1·90E+05	7·00E+02	
10^5	9·00E+01		1·90E+04	2·24E+07	2·90E+06	3·99E+06	
10^6				4·00E+04		2·17E+05	
10^6	9·00E+01	9·00E+01	9·00E+01	1·00E+02	5·80E+03	4·00E+02	
10^6			2·25E+04	1·54E+05	3·08E+06	4·20E+03	
10^7			9·00E+01	9·00E+01	3·80E+05		
10^7	9·00E+01	9·00E+01	9·00E+01	4·30E+05	8·20E+06	1·50E+07	
10^7	9·00E+01			1·76E+04	1·30E+04	7·00E+02	
10^8	9·00E+01	9·00E+01	1·00E+02	3·00E+02	4·00E+02	3·30E+04	
10^8	9·00E+01	9·00E+01	3·50E+04	4·90E+04	1·80E+03	2·41E+04	
10^8	9·00E+01	9·00E+01	1·20E+05	3·60E+03	1·88E+05	1·30E+06	
10^9	9·00E+01	9·00E+01	1·56E+06	1·00E+05	4·20E+03	1·21E+04	
10^9			2·00E+02	3·49E+06	5·00E+06	2·60E+05	
10^9				3·00E+07	2·20E+03	8·30E+06	
10^{10}	9·00E+01	7·90E+05	4·40E+03	3·00E+02	9·00E+02	1·00E+02	
10^{10}	9·00E+01	5·30E+05	3·30E+04	3·67E+07	1·93E+04	1·29E+05	
10^{10}			1·10E+06	1·10E+04	9·70E+05	2·10E+04	

Dose-Expansion Module

	Abundace of PanChol (CFU/g)						
Dose (CFU)	D1	D1·2	D2	D3	D4	D5	Notes
0	9·00E+01		9·00E+01	9·00E+01		9·00E+01	
0	9·00E+01		9·00E+01	9·00E+01	9·00E+01	9·00E+01	
0	9·00E+01						
0	9·00E+01	9·00E+01	9·00E+01	9·00E+01	9·00E+01	9·00E+01	
0		9·00E+01	9·00E+01	9·00E+01	9·00E+01	9·00E+01	
0	9·00E+01	9·00E+01	9·00E+01	9·00E+01	9·00E+01	9·00E+01	
0	9·00E+01		9·00E+01	9·00E+01	9·00E+01	9·00E+01	
0	9·00E+01	9·00E+01	9·00E+01	9·00E+01	9·00E+01	9·00E+01	
2×10^7	9·00E+01	9·00E+01	4·00E+02	1·40E+03	3·00E+02	9·00E+01	
2×10^7	9·00E+01	9·00E+01	9·00E+01	5·00E+04	2·42E+05	4·30E+05	
2×10^7			9·00E+01	6·00E+02			
2×10^7	9·00E+01		5·00E+02	5·20E+05	4·00E+02	1·66E+04	
2×10^7	9·00E+01	9·00E+01	4·00E+02	1·80E+05	9·30E+03	4·10E+03	
2×10^7	9·00E+01		9·00E+01	5·00E+04	3·60E+05	9·80E+06	
2×10^7	9·00E+01	9·00E+01	1·97E+07	2·00E+05			early discharge
2×10^7	9·00E+01	9·00E+01	1·00E+02	6·60E+03	9·00E+01	2·08E+06	
2×10^7	9·00E+01		1·20E+03	3·76E+07	3·50E+06	8·50E+03	
2×10^7	9·00E+01	9·00E+01	3·30E+04	2·20E+04	5·50E+03	3·10E+05	
2×10^7	9·00E+01		8·30E+04	1·40E+05		1·37E+06	
2×10^7	9·00E+01		9·00E+01			1·00E+02	
2×10^7	9·00E+01	9·00E+01	9·00E+01	1·40E+06	5·10E+03	2·00E+02	
2×10^7	9·00E+01	9·00E+01	9·00E+01		1·00E+02	9·00E+01	
2×10^8	9·00E+01	9·00E+01	9·00E+01	9·00E+01	9·00E+01	9·00E+01	
2×10^8			9·00E+01	2·00E+04	4·60E+04	1·10E+05	
2×10^8	9·00E+01		1·33E+05	1·16E+05	7·80E+04	9·70E+04	
2×10^8		9·00E+01		9·00E+01		1·50E+05	
2×10^8		9·00E+01	8·00E+03	9·00E+01	1·00E+02	8·00E+02	
2×10^8	9·00E+01	3·00E+03	3·76E+06	7·00E+05	3·12E+04	8·30E+05	
2×10^8	9·00E+01		9·00E+01	4·50E+05	1·82E+07	5·20E+05	
2×10^8	9·00E+01	9·00E+01	2·01E+06	7·40E+06	4·20E+06	5·80E+06	
2×10^8	9·00E+01	9·00E+01	1·00E+02	9·50E+05	3·80E+06	2·00E+05	
2×10^8	9·00E+01	9·00E+01	9·00E+01	9·00E+01	9·00E+01	9·00E+01	
2×10^8	9·00E+01	9·00E+01	2·10E+03	5·80E+04	2·00E+02	9·00E+01	
2×10^8	9·00E+01	9·00E+01	9·00E+01	1·30E+03	2·30E+05	1·30E+03	
2×10^8	9·00E+01	9·00E+01	9·00E+01	9·70E+06			early discharge
2×10^8	9·00E+01	9·00E+01	1·23E+04	1·00E+02	6·60E+05	7·80E+03	

no sample
negative rectal swab
positive rectal swab

Limit of detection: 100 CFU/g
No shedding is presented as 90 CFU/g.
