# Supplementary material for: Safety and immunogenicity of PanChol, a single-dose live-attenuated oral cholera vaccine: results from a phase 1a, double-blind, randomised, placebo-controlled trial
Source: Lancet Infect Dis. 2026 May;26(5):497–509. doi: 10.1016/S1473-3099(25)00682-6 (PMC13103297; doi:10.1016/S1473-3099(25)00682-6)
Supplement: Supplementary appendix 2 [file mmc2.pdf]

THE LANCET
Infectious Diseases

Supplementary appendix 2

This appendix formed part of the original submission and has been peer reviewed. We post it as supplied by the authors.

Supplement to: Leitner DR, Walsh SR, Suzuki M, et al. Safety and immunogenicity of PanChol, a single-dose live-attenuated oral cholera vaccine: results from a phase 1a, double-blind, randomised, placebo-controlled trial. *Lancet Infect Dis* 2026; published online Jan 7. https://doi.org/10.1016/S1473-3099(25)00682-6.

PanChol-100: Safety and Immunogenicity of PanChol: First-in-Human Study of a Novel Live Attenuated Oral Cholera Vaccine

**Investigational Product**	**PanChol**
**Protocol Number**	**PanChol-100**
**Version Number**	**4.0**
**Version Date**	**10/02/2024**
**IRB Amendment**	**73**
**Short Title**	**First in Human Phase 1 Ascending Dose Study of PanChol in Healthy Volunteers**
**Plan of Development**	**Phase 1**
**Sponsor**	**IND Holder: Lindsey R. Baden, MD**

Confidentiality Statement

This document contains confidential proprietary information that is under the control of the Sponsor at Brigham and Women's Hospital. The information is only to be used in connection with authorized clinical studies of the investigational drug described in this document. You may disclose the contents of the document while acting in the capacity of an investigator, potential investigatory or consultant to study personnel under your supervision or to and Institutional Review Board who needs to know the contents and who has been advised of the confidential nature of the document. You may not copy, disclose or publish any information to others without the written approval of the Sponsor.

For Review Only

PROTOCOL AND AMENDMENTS

Version Number	Version Date	Details of Revisions
1.0	January 14, 2022	Original version submitted to FDA in February 2022<br>Approved by BWH IRB on 04AUG2022.
2.0	August 29, 2022	Updated version with the responses to FDA and IRB suggested changes. Additional updates in regards to PSRT, stool sample, DSMB, Institutional Ethics Committee, monitoring, blinding treatment, unblinded randomization scheme, informed consent, clinical laboratory values, study drug accountability, source documents, fasting, and stool culture.
2.0	October 20, 2022	Updated version with additional IRB suggested changes.
2.1	March 6, 2023	Administrative update to correct protocol version
3.0	January 10, 2024	Implement 21 December 2023 DSMB recommendations
4.0.	October 02, 2024	Protocol amendment for the addition of lower doses of PanChol than previously included in module 1.

Itemized Revisions	Protocol Section
Standardized language:<br>All mentions of "Study Center" were changed to "Site".<br><br>All mentions of "Clinical Team" were changed to "Study Team"	
Creation of table with itemized revisions	
Updated version number and date	
Day 2 clinical labs added	Appendix 1 Schedule of Assessments<br><br>Section 6.7.1 Clinical Lab Assessments
Inpatient stool assessment will occur each day	Section 6.6.5 Stool Examination
Language was edited to specify that participants will be given doxycycline to eradicate the shedding of the vaccine organism on ~Day 5.	Protocol Synopsis; Study Design<br><br>Section 2.5.4 Duration of Treatment
Language was added to specify administration of doxycycline, "In this study, doxycycline will be administered as 200 mg on the first day, followed by 100 mg twice a day for 4 days."	Section 8.3 Other Protocol Required Treatment Procedures
Language was added to Module 2 design, "All three subjects in each cohort will receive a single dose based on the CRM model fit to all the available dose-response data including from Modules 1 and 2."	Section 2.5.2 Module 2

For Review Only

Exclusion Criteria<br>• Individuals who do not speak English will not be enrolled into this trial. This study involves more than minimal risk and no prospect of direct benefit for participants. Additionally, a subject who did not speak English may not be able to easily communicate safety concerns in a timely fashion to the study investigators.<br>• Childcare workers with direct contact with children ≤ 2 years of age<br>• Individuals whose occupation involves handling of food<br>• Healthcare workers who have direct contact with patients who are immunodeficient, HIV-positive, or have an unstable medical condition<br>• Have diarrhea within 48 hours before enrollment<br>• Have a history of hypersensitivity to any of the tetracyclines<br>• Have a history of hypersensitivity to streptomycin or any aminoglycoside due to the known cross-sensitivity of patients to drugs in this class.<br>• Individuals who have a household member who are immunodeficient, HIV-positive, or have an unstable medical condition.	Protocol Synopsis; Exclusion Criteria<br><br>Section 5.2 Exclusion Criteria
Additional language for screening procedures:<br>• Recruitment for this study will be through the prior IRB approved vaccine-screening protocol (IRB #2002-P-000343) and directly through this trial. Recruitment materials will be used to engage interested and eligible individuals, which will be managed through the prior IRB approved vaccine-screening protocol (IRB #2002-P-000343). The Research Study Volunteer Program (RSVP) for Health will also be used to recruit participants. Participants will sign the consent form, approved under the vaccine-screening protocol (IRB #2002-P-000343), for this general pre-screening protocol to undergo screening procedures. Screening procedures include performing laboratory tests, collecting medical history, and performing physical examinations. Those individuals determined to be eligible, based on the inclusion and exclusion criteria, will be enrolled in the study and will sign the study specific consent form.	Section 4.1 Screening
Pre-Screen Visit procedures outlined:<br>• Obtain medical history, height, weight, physical exam.<br>• Draw a blood sample to perform routine blood test and to test for HIV infection. We may draw about 2 tablespoons of blood.<br>• Collect urine sample to perform routine analysis and to test for pregnancy, if they are a female who can become pregnant	Section 4.1 Screening

For Review Only

Inpatient Period Procedures updated<br>• Each participant will have a private room and private bathroom during the inpatient period of the study.<br>• At discharge, staff will review with each participant the importance of good hand hygiene to minimize potential spread to family and other close contacts."	Section 6.4.1 Inpatient Period
Vital Signs Procedures updated<br>• At 30 minutes post vaccination an assessment will be conducted including vital signs, inquiring whether participant is having any signs and symptoms which may prompt a detailed physical exam by clinicians.	Section 6.6.2 Vital Signs
Remuneration/Payments	Section 12.9 Remuneration/Payments
Subject Texting Preference	Section 12.10 Participant Texting Preference
Safety Analysis Procedures<br>• All AEs will be collected and reported until D29. From D30 to D180, medically attended adverse events (MAAEs), new-onset chronic medical conditions (NOCMC) and SAEs will be collected and reported.	Section 8.4.4 New onset chronic medical conditions<br><br>Section 9.4.2 Non-Serious Adverse Events<br><br>Section 10.7 Safety Analysis
Safety Monitoring Rules for Reactogenic Events updated<br>• Specify study staff as personnel who will actively solicit solicited adverse events during specified post-vaccination period.<br>• Tiredness was included as solicited AE.	**1.1.1 Section 8.4.1 Safety Monitoring Rules for Reactogenic Events**
Privacy and Confidentiality section added	Section 11.6 Privacy and Confidentiality
Appendix 3 revised<br>• Include number and types of qualifications of members<br>• Frequency of meetings	Appendix 3: Data Monitoring Committee
Schedule of Assessments<br>• D2 and D6 updates<br>• Visit day for "Visit 7" was updated to "D180"	Appendix 1: Schedule of Assessments
Study Drug Preparation<br>• The appearance of PanChol will vary depending on dose. In all cases, it will be a non-viscous, homogenous liquid with no visible contaminants. At doses of 10^9 and 10^{10} the liquid will be cloudy cream-colored. At the lower doses the liquid be clear.	Section 8.1 Study Drug Preparation
Product Storage<br>• Both PanChol and the placebo will be prepared in opaque amber bottles by the pharmacy and therefore the	Section 8.1.1 Product Storage and Handling

For Review Only

investigators and the participants will not be able to discern whether they received drug products vs placebo.	
Study Drug Administration<br>• The volume of the study product consumed will be collected and recorded. Participants will be monitored by study staff for 30 minutes after product administration. Immediate reactogenicity events and adverse events will be collected and recorded.	Section 8.2 Study Drug Administration
Stool sample kit will be given to participant during screening visit and instructed to collect and bring sample on day of admission.	Section 4.1 Screening
Language was updated for DSMB's recommendations post DSMB meeting.	Protocol Synopsis<br><br>Section 3.10 Data Safety Monitoring Board
Spelled out PSRT.<br>Deleted row "Brigham and Women's Hospital" as this was not needed.	Protocol Synopsis
Deleted all mentions of Independent or Institutional Ethics Committee (IEC) as these are not applicable.	Study Glossary<br><br>Section 11.3 Institutional Review Board<br><br>Section 11.5 Subject Confidentiality<br><br>Section 12.1 Protocol Amendments<br><br>Section 12.2 Study Termination<br><br>Section 12.3 Study Documentation and Storage<br><br>Section 12.7 Site Audits
Deleted original section 2.6.2 Chemistry as this section was not applicable. Other section numbers were updated accordingly.	Previous section 2.6.2 Chemistry
Clarified on-site and remote monitoring of the site by Sponsor with source data verification will be according to the Safety Monitoring Plan.	Section 3.4 Quality Control and Quality Assurance
Language updated from "proceeding" to "preceding".	Section 3.11 Dose Escalation and Initiation of Expansion Cohorts
Updated language to clarify that sponsor and all subjects, monitors and Site personnel related to the study will be blinded throughout Module 3.	Section 4.4 Blinding of Treatment Assignment
Updated language to clarify study statistician will maintain securely an unblinded randomization scheme.	Section 4.5 Unblinding of Treatment Assignment

For Review Only

Deleted language detailing informed consent forms being prepared in the language(s) of potential subjects as only English-speaking participants will be enrolled.	Section 11.1 Informed Consent
Deleted language detailing clinical laboratory values will be entered into eCRFs.	Section 12.5 Data Capture Methods
PSRT review will happen routinely.	Section 2.5.2 Module: Adaptive Dose-finding/Optimization
Updated language to clarify PSRT will evaluate treatment-related Grade 3 or higher AE as potentially dose-limiting.	Section 2.12.2.1 Module
Updated language to clarify that Investigational Pharmacist will destroy used or thawed vials.	Section 7.3 Study Drug Accountability
Updated language to clarify source documents may be eCRFs and paper CRFs.	Section 12.3 Study Documentation and Storage
Included language to specify participant will require to fast 60 minutes before and after vaccine administration.	Section 3.7.1 Study Drug Administration and Inpatient Period
Deleted "Stool will be transported in Cary Blair medium. Only"	Section 6.7.5 Stool culture
**Version 3.0 January 10, 2024 Protocol Itemized Revisions**	
Revised language to update Modules based on DSMB recommendations.	Section 2.5 Rationale Study Duration Total number of modules changed from three to two, removing module 2 (adaptive dose/optimization) and Figure 1 updated.
Added Section 2.5.2 Rationale for Module Updates	Section 2.5.2 added to provide rationale for not needing Module 2 and Dose Selection for Module 3.
Revised Section 2.53 to 2.54 Module Expansion Cohort	This section revised to include dosing updates and participant totals updated (28 active product and 8 placebo)
Revised Section 2.5 to 2.55 Duration of Treatment	Revised to indicate doxycycline will be started on day 5 instead of day 6.
Revised Section 3.2 Study Design	Revised to reflect change from three to two modules.
Deleted Section 3.2.2 Module 2 Adaptive Dose Finding/Optimization	This section deleted to reflect DSMB module recommendations.
Revised Section 3.2 to 3.2.2 Module 3 Expansion Cohort	Module 3 participant number and dose level updated. Table 4 revised to reflect these changes.
Revised Section 3.6 Number of Subjects	Revised section to reflect DSMB recommendations.

For Review Only

Revised Section 3.10 DSMB	Paragraph 2 updated module number from 3 to 2.
Revised Section 4.1 Screening	Recruitment method updated.
Revised Section 4.2 Randomization	Changed Module from 2 to 3.
Revised Section 4.4 Blinding of Treatment Assignment	Changed Module from 2 to 3.
Revised Section 6.4.1 Inpatient period	Doxycycline administration starting on day 5 instead of day 6.
Revised Section 8.3 Other Protocol Required Treatment Procedures	Doxycycline administration starting on day 5 instead of day 6.
Revised Section 10 Statistical Considerations	The overview describes protocol revision based on results from Module 1.
Revised Section 10.1 Sample Size Considerations	Total number of modules changed from 3 to 2. After the first module (dose-ranging), the next module (expansion) will require 36 participants.
Revised Section 10.4.1 Randomization/Blinding	Updated modules and number of participants.
Appendix 1: Schedule of Assessments Table	Doxycycline administration start day updated from Day 6 to Day 5.
**Version 4.0 October 02, 2024 Protocol Itemized Revisions**	
Revised Study Design in Protocol Synopsis	Updated to reflect that volunteers will return on Day 85.
Revised Exclusion Criteria in Protocol Synopsis	Allergy or intolerance to Panchol components updated to include only serious allergic reactions.
Revised Study Drug Dosage in Protocol Synopsis	Two additional doses of 10^5 and 10^4 added to module 1.
Revised Rationale for Dose Selection in Protocol Synopsis	Updated to include dose de-escalation.
Revised Study Visit Schedule and Procedures in Protocol Synopsis	Update to reflect baseline stool may be collected up to 10 days pre-dose or on Day 1
Revised Study Visit Schedule and Procedures in Protocol Synopsis	Updated to reflect that volunteers will return on Day 85.
Revised Figure 1	Figure updated to include dose de-escalation.
Revised Section 2.5.1	Revised to include dose de-escalation.
Addition of 2.5.2 Update on Module 1 results	2.5.2 added to include results from Module 1 which informed this protocol revision.
Addition of 2.5.6 Rationale for dose de-escalation	2.5.6 added to include rational for additional two doses into Module 1.

For Review Only

Revised Section 2.5.8 Duration of Post Treatment Follow-Up	Updated to reflect that volunteers will return on Day 85.
Updated Table 1. Quantitative Composition of PanChol Drug Product	Updated quantity of dose to begin at $1x10^4$.
Revised Section 3.2 Study Design	Updated to reflect that volunteers will return on Day 85.
Revised Table 2	Cohort 6 and 7 added into the Module 1 design.
Revised Section 3.6 Number of Subjects	Number of subjects updated to approximately 57.
Revised Section 3.7 Overall Study Duration and Follow-up	Update to reflect baseline stool may be collected up to 10 days pre-dose or on Day 1
Revised Section 3.7.2 Study Drug Administration and Inpatient Period	Doxycycline administration start day updated to Day 5.
Revised Section 3.7.3 Outpatient Follow-Up	Updated to reflect that volunteers will return on Day 85.
Revised Section 5.2 Exclusion Criteria	Allergy or intolerance to Panchol components updated to include only serious allergic reactions.
Revised Section 10.1 Sample Size Considerations	Additional 6 participants added to module 1.
Revised Appendix 1: Schedule of Assessments	Table format updated for inpatient period. Additional screening period for Day-10 to Day -1 included for baseline stool sample collection. Blood sample for Proteomics/Metabolomics included for inpatient and outpatient follow up. Footnotes e and f included for Stool sample for culture on inpatient Day 6, Day 7, and Day 8. Stool sample for exploratory endpoints updated to occur on Day 1 through Day 5 of inpatient period.

For Review Only

STUDY CONTACTS

Key Sponsor Contact	Lindsey Baden, MD<br>Brigham and Women's Hospital<br>15 Francis Street<br>Boston, MA 02115<br>lbaden@bwh.harvard.edu

For Review Only

BRIGHAM AND WOMEN'S HOSPITAL SIGNATURE PAGE

**Title**	Safety and Immunogenicity of PanChol: First-in-human study of a novel live attenuated oral cholera vaccine
**Protocol Number**	PanChol 100
**Version Number**	4.0
**Version Date**	October 02, 2024
**IRB Amendment**	73

The design of this study as outlined by this protocol has been reviewed and approved by:

____________________ ____________

Lindsey Baden, M.D. Date

For Review Only

PRINCIPAL INVESTIGATOR SIGNATURE PAGE

I hereby acknowledge that I have read the protocol, appendices and accessory materials related to Study PanChol -100 dated 02 October 2024 and agree to the following:

- To conduct this study as described in by the protocol and any accessory materials
- To protect the rights, safety, and welfare of participants under my care
- To provide oversight to all personnel to whom study activities have been delegated
- To control all investigational products provided by the Sponsor and maintain records of the disposition of these products`
- To conduct the study in accordance with all applicable local and national regulations, the requirements of the ethics committee of record for my clinical site and Good Clinical Practices as outlined in ICH E6(R2).
- To obtain approval for the protocol and all written materials provided to participants prior to initiating the study at my site
- To obtain informed consent-updated consent in the event of new information or amendments- from all participants enrolled at my study site prior to initiating any study specific procedures or administering investigational products to those participants
- To maintain records of each subject's participation and all data required by the protocol
- To ensure that confidential information contained in this document will not be used for any purpose other than the evaluation of the clinical investigation without prior written consent of the Sponsor.

Principal Investigator Signature:

Signed: ______________________________ Date: ______________

Name
Title

MM/DD/YY

For Review Only

TABLE OF CONTENTS

PROTOCOL AND AMENDMENTS....2
1.1.1 Section 8.4.1 Safety Monitoring Rules for Reactogenic Events....4
STUDY CONTACTS....9
BRIGHAM AND WOMEN'S HOSPITAL SIGNATURE PAGE....10
PRINCIPAL INVESTIGATOR SIGNATURE PAGE....11
TABLE OF CONTENTS....12
LIST OF TABLES....18
LIST OF FIGURES....19
PROTOCOL SYNOPSIS....20
STUDY GLOSSARY....26
1 OBJECTIVES AND ENDPOINTS....28
1.1 Objectives....28
1.1.1 Primary Objective(s)....28
1.1.2 Secondary Objectives(s)....28
1.1.3 Exploratory Objective(s)....28
1.2 Study Endpoints....28
1.2.1 Primary Endpoint(s)....28
1.2.2 Secondary Endpoint(s)....28
1.2.3 Exploratory Endpoint(s)....29
2 BACKGROUND AND RATIONALE....30
2 30
2.1 Overview of Disease....30
2.1.1 Cholera epidemiology and *V. cholerae* classification....30
2.1.2 Cholera Vaccines....30
2.2 PanChol....32
2.2.1 Design and Pre-clinical Development....32
2.2.2 Selection of the optimal PanChol serotype....33
2.3 Therapeutic Rationale....34
2.4 Rationale for Dose, Regimen and Route of Administration....34

For Review Only

2.5 Rationale for Study Duration 35
2.5.1 Module 1: Fixed Dose-Ranging 36
2.5.2 Rational for Not Needing Module 2 and Dose Selection for Module 3 37
2.5.3 Module 2 – No longer needed 38
2.5.4 Module 3: Expansion Cohort 38
2.5.5 Duration of Treatment 38
2.5.6 Duration of Post Treatment Follow-up 38
2.6 Investigational Drug Product PanChol 39
2.6.1 Mechanism of Action 39
2.6.2 Drug Substance PanChol 39
2.6.3 Drug Product PanChol 40
2.6.4 Preclinical Experience 41
2.6.5 Clinical Experience 42
2.7 Risk-Benefit Assessment 42
2.7.1 Potential Risks 42
2.7.2 Potential Benefits 43
2.8 Ethical Considerations 43
3 EXPERIMENTAL PLAN 44
3 44
3.1 Compliance Study 44
3.2 Study Design 44
3.2.1 Module 1: Fixed Dose-ranging 44
3.2.2 Module 3: Expansion Cohort 45
3.3 Measures to Minimize/Avoid Bias 45
3.4 Quality Control and Quality Assurance 45
3.5 Number of Sites 46
3.6 Number of Subjects 46
3.7 Overall Study Duration and Follow-up 46
3.7.1 Screening 46
3.7.2 Study Drug Administration and Inpatient Period 46
3.7.3 Outpatient Follow-up 47

For Review Only

3.8 End of Study 47
3.9 Protocol Safety Review Team 47
3.10 Data Safety Monitoring Board....47
3.11 Dose Escalation and Initiation of Expansion Cohorts 47
3.12 Study Stopping Rules 47
3.12.1 Study Pausing Rules 47
3.12.2 Dose Escalation Pausing Rules....48
4 SUBJECT ENROLLMENT 49
4 49
4.1 Screening 49
4.2 Randomization....49
4.3 Replacement of Subjects....49
4.4 Blinding of Treatment Assignment 50
4.5 Unblinding of Treatment Assignment 50
5 SUBJECT ELIGIBILITY 51
5 51
5.1 Inclusion Criteria 51
5.2 Exclusion Criteria 51
6 STUDY PROCEDURES 53
6 53
6.1 Study Schedule 53
6.2 Study Assessments....53
6.3 Screening and Enrollment....53
6.4 Planned Study Visits....53
6.4.1 Inpatient period 53
6.4.2 Follow-up period....53
6.5 Unscheduled Study Visits....53
6.5.1 Withdrawal from the Study or Discontinuation of the Study Product....53
6.5.2 Study Termination 54
6.6 Clinical Evaluations....54
6.6.1 Medical History 54

For Review Only

6.6.2 Vital Signs 54
6.6.3 Physical Examination 54
6.6.4 Height and Weight 54
6.6.5 Stool Examination 54
6.7 Laboratory Evaluations 54
6.7.1 Clinical Laboratory Assessments 54
6.7.2 HIV Testing 55
6.7.3 Pregnancy Test 55
6.7.4 Immunogenicity assessment 55
6.7.5 Stool culture 55
6.7.6 Fecal microbiota profile 55
7 STUDY DRUG 56
7 56
7.1 Study Drug Description 56
7.2 Packaging and Labeling 56
7.3 Study Drug Accountability 56
8 TREATMENT OF SUBJECTS 57
8 57
8.1 Study Drug Preparation 57
8.1.1 Product Storage/Stability and Handling 57
8.2 Study Drug Administration 57
8.3 Other Protocol-Required Treatment Procedures 57
8.4 Safety Monitoring Rules 58
8.4.1 Safety Monitoring Rules for Reactogenic Events 58
8.4.2 Unsolicited Adverse Events 58
8.4.3 Medically Attended Adverse Events 58
8.4.4 New-onset chronic medical condition 58
8.4.5 Stopping Rules for Adverse Events 58
8.5 Withdrawal of Subjects from the Study Procedures 58
8.6 Concomitant Therapy and Procedures 59
9 SERIOUS AND NON-SERIOUS ADVERSE EVENT REPORTING 60

For Review Only

9 60
9.1 Sponsor Review of Safety Information 60
9.2 Regulatory Requirements 60
9.3 Definitions 60
9.3.1 Adverse Event 60
9.3.2 Adverse Drug Reaction and Unexpected Suspected Adverse Drug Reaction 61
9.3.3 Serious Adverse Event 61
9.3.4 Adverse Event of Special Interest 61
9.3.5 Targeted Medical Event 62
9.4 Monitoring and Recording Adverse Events 62
9.4.1 Serious Adverse Events 62
9.4.2 Non-Serious Adverse Events 62
9.4.3 Evaluation of Adverse Events (Serious and Non-Serious) 63
9.5 Procedures for Handling Special Situations 64
9.5.1 Abnormalities of Laboratory Tests 64
9.5.2 Prescheduled or Elective Procedures or Routinely Scheduled Treatments 64
9.5.3 Dosing Errors 64
9.5.4 Contraception and Pregnancy 65
10 STATISTICAL CONSIDERATIONS 66
10 66
10.1 Sample Size Considerations 66
10.2 Populations 66
10.3 Definition of Baseline 66
10.4 Treatment Assignment Procedures 66
10.4.1 Randomization/Blinding 66
10.5 Missing data Handling 67
10.6 Demographics and Baseline Characteristics 67
10.7 Safety analysis 67
10.8 Immunogenicity analysis 67
11 INVESTIGATOR'S REGULATORY OBLIGATIONS 68

For Review Only

11 68
11.1 Informed Consent 68
11.2 Ethical Conduct of the Study 68
11.3 Institutional Review Board 68
11.4 Consent for Future Use of Stored Specimens and Data 68
11.5 Subject Confidentiality 68
11.6 Privacy and Confidentiality 69
12 ADMINISTRATIVE AND LEGAL OBLIGATIONS....70
12 70
12.1 Protocol Amendments 70
12.2 Study Termination 70
12.3 Study Documentation and Storage 70
12.4 Data Coordinating Center/Biostatistician Responsibilities 71
12.5 Data Capture Methods 71
12.6 Study Monitoring....71
12.7 Site Audits 71
12.8 Language....72
12.9 Remuneration/Payments....72
12.10 Participant Texting Preference 72
13 LITERATURE REFERENCES....73
Appendix 1 : Schedule of Assessments....78
Appendix 2: Genetic Alterations in PanChol 81
Appendix 3: Data Monitoring Committee / Data and Safety Monitoring Board ..82

For Review Only

LIST OF TABLES

Table 1. Quantitative Composition of PanChol Drug Product 41
Table 2. Module 1 design..44
Table 3. Module 3 design..45
Table 4. PanChol Characteristics 56
Table 5. Severity of AE proposed scale 63

For Review Only

LIST OF FIGURES

Figure 1. Study Design....36

Figure 2: Operating Characteristics: Cumulative Probability of Stopping at or Before a Given Dose (Red) With True Probability of Response (Blue) in 3+3 Design....37

For Review Only

PROTOCOL SYNOPSIS

**Protocol Title**	Safety and Immunogenicity of PanChol: First-in-Human Study of a Novel Live Attenuated Oral Cholera Vaccine
**Study Phase**	1 (healthy volunteers)
**Indication**	Prevention of disease caused by cholera
**Primary Objectives**	• To evaluate the reactogenicity and the safety of a single-dose PanChol over a range of doses in healthy volunteers.<br>• To evaluate the immunogenicity of a single-dose PanChol over a range of doses as measured by vibriocidal antibody titers.
**Secondary Objectives**	• To further characterize PanChol immune response, such as the magnitude of vibriocidal titers, the IgG, IgA, and IgM antibodies targeting Inaba- and Ogawa-specific polysaccharides, cholera toxin B subunit, and TCP, IgA- and IgG-antibody secreting cell responses (ALS/plasmablast responses) and/or the memory B cell (MBC) response.<br>• To characterize the stool shedding of the PanChol organisms after vaccination.<br>• To evaluate the changes of the gut microbiota after PanChol vaccination and to compare these changes with cholera-induced changes on microbiota
**Study Design**	This study is a first-in-human, Phase 1 study of the safety, tolerability, and immunogenicity of PanChol in healthy volunteers. There will be two modules in this clinical trial assessing dosing, safety, and immunogenicity:<br>1) a fixed dose-ranging module, and<br>2) a placebo-controlled expansion module.<br>Participants will be enrolled at the Brigham and Women's Hospital (BWH). For the first days of the trial, participants will be inpatients at the Center for Clinical Investigation (CCI) in BWH, for optimal safety monitoring and for fecal and blood samples collection. PanChol or placebo will be administered on Day 1. On Day 5, participants will be starting doxycycline to eradicate the shedding of the vaccine organisms. On Day 7, those who are no longer excreting PanChol in their stool will be discharged. After discharge, volunteers will return on days 15, 29, 57, 85, and 180 for monitoring of general health, AE assessment, immune responses, and fecal microbiota composition.
**Number of Subjects**	Approximately 51 adult healthy volunteers are planned to be enrolled in this study if all planned treatment groups are conducted.
**Study Population**	**Inclusion Criteria**<br>1. Healthy adults aged from 18 to 55 years old.

For Review Only

	2. Considered healthy, as judged by the clinical investigator, according to medical history, physical examination, vital signs, screening laboratories, and medication history.<br>3. Understanding and agreeing to comply with the study protocol including the inpatient period.<br>4. Female participants must be non-pregnant and non-lactating and either<br>a. surgically sterile (history of bilateral ligation, bilateral salpingectomy, bilateral oophorectomy, total hysterectomy) or postmenopausal (defined as as amenorrhea for at least 12 consecutive months before screening without an alternative medical cause)<br>b. be of child-bearing potential and practicing an acceptable method of contraception or abstaining from all activities that could result in pregnancy for at least 28 days before vaccination until 3 months after receiving the IP.<br><br>Acceptable methods of contraception include barrier methods (such as condom, diaphragm, or cervical cap used in conjunction with spermicide), intrauterine device, hormonal contraception (that may be taken or administered by oral, intravaginal, transdermal, subdermal or IM route), vasectomized partner (the vasectomized partner should be the sole partner for that participant).<br><br>**Exclusion Criteria**<br>Each participant must not meet any of the following exclusion criteria to be eligible for enrollment in the study:<br>1. Confirmed or suspected immunosuppressive condition, as a result of a disease (e.g., primary immune deficiency, malignancy, HIV infection) or have taken any systemic immunosuppressive therapy within 6 months of enrollment.<br>2. Pregnant or lactating women<br>3. History of gastrointestinal (GI) disorder, such as previous major GI surgery, malabsorption, or any chronic GI disorders that would interfere, according to the investigator, with the IP.<br>4. Acute GI or febrile illness within 7 days of enrollment.<br>5. Have any acute or chronic medical condition that, in the opinion of the investigator, would make vaccination unsafe or interfere with the evaluation of immune response to study vaccination.<br>6. History of cholera vaccination<br>7. History of cholera infection<br>8. Abnormal stool pattern, defined as < 3 or >21 stools per week.<br>9. Serious allergic reaction to PanChol or placebo component (sodium bicarbonate, lactose, ascorbic acid)<br>10. Use of any systemic antibiotics within 1 month of PanChol administration<br>11. Receipt of a live vaccine in the previous 4 weeks or planned in the 4 weeks following enrollment

For Review Only

12. Receipt of a killed or subunit (non-live) vaccine in the previous 2 weeks or planned in the 2 weeks following enrollment.
13. Individuals who do not speak English will not be enrolled into this trial. This study involves more than minimal risk and no prospect of direct benefit for participants. Additionally, a subject who did not speak English may not be able to easily communicate safety concerns in a timely fashion to the study investigators
14. Childcare workers with direct contact with children ≤ 2 years of age
15. Individuals whose occupation involves handling of food
16. Healthcare workers who have direct contact with patients who are immunodeficient, HIV-positive, or have an unstable medical condition
17. Use laxatives regularly
18. Have diarrhea within 48 hours before enrollment
19. Have a history of hypersensitivity to any of the tetracyclines
20. Have a history of hypersensitivity to streptomycin or any aminoglycoside due to the known cross-sensitivity of patients to drugs in this class.
21. Individuals who have a household member who are immunodeficient, HIV-positive, or have an unstable medical condition.

Study Drug Dosage and Administration

**Single Dose will be administered (Total N= 57)**			
**Module 1 (n=21)**	**Dose: CFU**	**Module 3 (n=36) Randomized**	
Cohort 1 (n=3)	~10^6		
Cohort 2 (n=3)	~10^7	PanChol ~2×10^7	n=14
Cohort 3 (n=3)	~10^8	PanChol ~2×10^8	n=14
Cohort 4 (n=3)	~10^9	Placebo	n=8
Cohort 5 (n=3)	~10^{10}		
Cohort 6 (n=3)	~10^5		
Cohort 7 (n=3)	~10^4		

Rationale for Dose Selection

To determine the starting dose in this trial, previous animal experiments and other cholera vaccine trials were used. In an infant rabbit model, a dose of 10^9 CFU of PanChol did not cause cholera-like illness and was shown to reduce intestinal colonization by the WT challenge strains, slowed disease progression, and reduced mortality in an infant rabbit model of cholera. Based on FDA approval of Vaxchora®, published studies and to ensure an appropriate safety margin, a starting dose of 10^6 CFU will be used. This dose is 2-3 logs (100-1000×) lower than doses shown to be safe with previous live

For Review Only

	OCVs. In the first module of the study, the fixed-dose ranging module, the dose will be increased stepwise by factors of 10, in a total of five different cohorts, with a maximum dose of 10^{10} CFU. The lowest dose of the fixed-dose ranging module (10^6) elicited robust immunogenicity. Two additional cohorts will be added in a dose de-escalation design: 10^5 and 10^4 CFU. These lower doses are expected to be safe given the safety and tolerability at higher doses, and will help identify the minimum dose that elicits a robust immune response.
**Adjustment of Dose**	Adjustment of dose is not permitted.
**Safety Review Committee**	Safety oversight will be conducted by a DSMB. The DSMB members will be separate and independent of study personnel participating in this study and should not have scientific, financial, or other conflicts of interest related to this study.<br>The DSMB will review data following completion of the 48-hour safety data from the final participants of Module 1 and will conduct ad hoc reviews as appropriate when a halting rule is met or for immediate concerns regarding observations during this study. Additional data, interim statistical reports, or the unblinding of a treatment assignment (for Module 2) may be requested as deemed necessary for the DSMB review of participant safety or any concerns with the study. After each meeting, the DSMB will make recommendations on the continuation of the study.
**Study Stopping Criteria**	Enrollment will be paused, and an *ad hoc* DSMB safety review will be triggered if one or more of the following criteria are met:<br>1) One participant experiences a treatment-related serious adverse event (SAE)<br>2) If three or more participants experience the same treatment-related Grade 3 or higher AE
**Study Visit Schedule and Procedures**	Detailed information regarding study procedures are outlined in Section 6, and Appendix 1.<br>Blood and stool samples will be collected regularly throughout the study for safety and immunogenicity analysis. The safety of PanChol will be monitored in an ongoing fashion during the study by the Sponsor (or designee), study team and Protocol Safety Review Team (PSRT) prior to dose escalation. Participants may be required to attend additional visits for monitoring of AEs or abnormal investigation results.<br><br>The study comprises up to 56 Day Screening Period, a minimum of 6 days of inpatient period and an outpatient follow-up period. The length of each subject's participation is approximately 24 weeks from screening to last visit. Baseline evaluations will be performed pre-dose on Day 1. Baseline stool may be collected up to 10 days pre-dose or on Day 1. For the first days of the trial, participants will be inpatients at the Center for Clinical Investigation (CCI) in BWH, for optimal safety monitoring and for fecal and blood samples collection. PanChol or placebo will be administered on Day 1. On Day 5, participants will be starting doxycycline to eradicate the shedding of the vaccine organisms. On Day 7, those who are no longer excreting PanChol in

For Review Only

	their stool will be discharged. After discharge, volunteers will return on for outpatient visits on days 15, 29, 57, 85, and 180 for monitoring of general health, AE assessment, immune responses, and fecal microbiota composition. The final visit will be on day 180.
**Primary Endpoints**	The primary endpoints will be:<br>• The incidence of solicited and unsolicited adverse events, including serious adverse events, following PanChol vaccination.<br>• The seroconversion (4-fold rise titer over baseline) of the vibriocidal titers to both Inaba and Ogawa *V. cholerae* between pre- and post-vaccination with PanChol.
**Secondary Endpoints**	The secondary endpoints will be:<br>• The magnitude of pre- and post-vaccination serum vibriocidal titers to both Inaba and Ogawa *V. cholerae*<br>• The stool shedding of PanChol organisms using quantitative and qualititative stool cultures.
**Exploratory Endpoints**	Exploratory endpoints may include:<br>• The changes of IgG, IgA, and IgM antibodies targeting Inaba- and Ogawa-specific polysaccharides, CT-B and TCP<br>• The changes of IgA- and IgG-antibody secreting cell responses (ALS/plasmablast responses) and the memory B cell (MBC) response.<br>• The stool microbiota modification according to 16S rRNA sequencing and/or metagenomics
**Statistical Considerations**	**Sample Size**<br>No formal sample size calculation was performed. Based on experience from previous studies with other cholera vaccines, the chosen cohort sizes are considered sufficient to meet the objectives of the study while minimizing unnecessary exposure.<br>**Safety and Tolerability**<br>Reactogenicity will be assessed by soliciting specific and pre-defined signs and symptoms occurring after the study product administration during the inpatient period.<br>All AEs will be collected and reported until D29. From D30 to D180, medically attended adverse events (MAAEs), new-onset chronic medical conditions (NOCMC), and SAEs will be collected and reported.<br>The severity of AEs and SAEs will be graded according to the FDA Toxicity Grading Scale for Healthy Adults and Adolescent Volunteers Enrolled in Preventive Vaccine Clinical Trials, September 2007.<br>Any AE not listed in this scale will be graded as follows:<br>• **Mild:** The event is easily tolerated by the subject and does not affect the subject's usual daily activities<br>• **Moderate:** The event causes the subject more discomfort and interrupts the subject's usually daily activities

For Review Only

	• **Severe:** The event is incapacitating and causes considerable interference with the subject's usual daily activities<br>**Immunogenicity**<br>Immunogenicity measures include pre-and post-vaccination antibody measurement, such as titers of vibriocidal antibodies to both Inaba and Ogawa *V. cholerae* will be measured. IgG, IgA, and IgM antibodies targeting Inaba- and Ogawa-specific polysaccharides, cholera toxin B subunit, and TCP, may be performed.

For Review Only

STUDY GLOSSARY

Abbreviation	Definition
ADR	Adverse drug reaction
AE	Adverse Event/Adverse Experience
AESI	Adverse event of special interest
ALS	Antibody in Lymphocyte Supernatant
BWH	Brigham and Women's Hospital
CBC	Complete blood count
CCI	Center for Clinical Investigation
CDC	Centers for Disease Control and Prevention
CFU	Colony forming units
CRF	Case Report Form
CRISPR	Clustered regularly interspaced short palindromic repeats
CRM	Continual reassessment method
CSR	Clinical study report
CT	Cholera toxin
CT-B	Cholera toxin B subunit
CTXΦ	Cholera toxin phage
DCC	Data Coordinating Center
DLT	Dose-limiting toxicity
DSMB	Data Safety Monitoring Board
eCRF	Electronic Case Report Form
EDC	Electronic Data Capture
ET	Early termination
ETEC	Enterotoxigenic Escherichia coli
FDA	Food and Drug Administration
FIH	First in human
FSH	Follicular stimulating hormone
GCP	Good Clinical Practice
GF	Germ-free
GI	Gastrointestinal
ICE	Integrative conjugative element
ICF	Informed Consent Form
ICH	International Conference on Harmonization
Ig (A, G, M)	Immunoglobulin (class A, G, M)

For Review Only

Abbreviation	Definition
IND	Investigational New Drug Application
IP	Investigational product
IRB	Institutional Review Board
IUD	Intrauterine contraception device
IUS	Intrauterine hormone-releasing system
MAAE	Medically attended adverse event
MBC	Memory B cell
MGB	Mass General Brigham
N	Number (typically refers to participants)
NOCMC	New-onset chronic medical condition
OAEI	Other adverse events of interest
OC	Operating characteristics
OCV	Oral cholera vaccine
PE	Protective efficacy
PI	Principal Investigator
PSRT	Protocol Safety Review Team
QA	Quality Assurance
QC	Quality Control
RSVP	Research Study Volunteer Program
SAE	Serious Adverse Event/Serious Adverse Experience
SI	Small intestine
SoA	Schedule of Assessments
SXT	Sulfamethoxazole/Trimethoprim
TCP	Toxin-co-regulated pili
TME	Targeted medical event
US	United States
WHO	World Health Organization
WOCBP	Women of childbearing potential
WRAIR	Walter Reed Army Institute of Research
WT	Wild-type

For Review Only

1 OBJECTIVES AND ENDPOINTS

1.1 Objectives

1.1.1 Primary Objective(s)

The primary objectives are:

- To evaluate the reactogenicity and the safety of a single-dose PanChol over a range of doses in healthy volunteers.
- To evaluate the immunogenicity of a single-dose PanChol over a range of doses as measured by vibriocidal antibody titers.

1.1.2 Secondary Objectives(s)

The secondary objectives are:

- To further characterize PanChol immune response, such as the magnitude of vibriocidal titers, the IgG, IgA, and IgM antibodies targeting Inaba- and Ogawa-specific polysaccharides, cholera toxin B subunit (CT-B), and toxin co-regulated pili (TCP), IgA- and IgG-antibody secreting cell responses (ALS/plasmablast responses) and/or the memory B cell (MBC) response.
- To characterize the stool shedding of the PanChol organisms after vaccination.
- To evaluate the changes of the microbiota after PanChol vaccination and to compare these changes with cholera-induced changes on microbiota.

1.1.3 Exploratory Objective(s)

- The changes of IgG, IgA, and IgM antibodies targeting Inaba- and Ogawa-specific polysaccharides, CT-B and TCP.
- The changes of IgA- and IgG-antibody secreting cell responses (ALSplasmablast responses) and the MBC response.
- The stool microbiota modification according to 16S rRNA sequencing and/or metagenomic

1.2 Study Endpoints

1.2.1 Primary Endpoint(s)

The primary endpoints will be:

- The incidence of solicited and unsolicited adverse events, including serious adverse events, following PanChol vaccination.
- The seroconversion (4-fold rise titer over baseline) of the vibriocidal titers to both Inaba and Ogawa *V. cholerae* between pre- and post-vaccination with PanChol.

1.2.2 Secondary Endpoint(s)

The secondary endpoints will be:

- The magnitude of pre- and post-vaccination serum vibriocidal titers to both Inaba and Ogawa *V. cholerae*.
- The stool shedding of PanChol organisms using quantitative and qualitative stool cultures.

For Review Only

1.2.3 Exploratory Endpoint(s)

Exploratory endpoints may include:

- The changes of IgG, IgA, and IgM antibodies targeting Inaba- and Ogawa-specific polysaccharides, CT-B and TCP.
- The changes of IgA- and IgG-antibody secreting cell responses (ALSplasmablast responses) and the MBC response.
- The stool microbiota modification according to 16S rRNA sequencing and/or metagenomics.

For Review Only

2 BACKGROUND AND RATIONALE

2.1 Overview of Disease

2.1.1 Cholera epidemiology and *V. cholerae* classification

Cholera, a severely dehydrating diarrheal disease that can be fatal within hours of onset, is caused by *Vibrio cholerae*, a Gram-negative bacterium (Clemens et al., 2017). Recent estimates suggest there are approximately 2.9 million cholera cases and 95,000 deaths attributable to cholera annually worldwide (Ali et al., 2015). Most deaths occur among children under five years (Harris et al., 2012). Humans become infected with *V. cholerae* after ingestion of contaminated water or food or via fecal-oral transmission. After passing through the stomach, *V. cholerae* multiplies to high densities in the small intestine (SI), a process referred to as intestinal colonization. Colonization is dependent upon *V. cholerae*'s production of the TCP (Herrington et al., 1988; Taylor et al., 1987). The severe secretory diarrhea that is the hallmark of *V. cholerae* infection is induced by cholera toxin (CT), an AB5-type protein toxin secreted by the pathogen in the SI, which binds via its pentameric B subunit (CT-B) to the GM1 ganglioside on host cells and is subsequently internalized. The evolution of pathogenic *V. cholerae* from ancestral strains has been profoundly shaped by their acquisition of mobile genetic elements. Importantly, CT is encoded in the genome of cholera toxin phage (CTXΦ), a lysogenic filamentous phage, and other live cholera vaccines, which have been deleted for the genes encoding CT (*ctxAB*), can revert to toxigenicity via infection with CTXΦ (Waldor and Mekalanos 1996).

Serogroup classification of *V. cholerae* is determined by the composition of the LPS O-antigen and the O1 serogroup is thought to have given rise to all pandemic cholera. The O1 serogroup includes two principal serotypes, Ogawa and Inaba, which differ in the methylation of the O- antigen, as well as a more unusual Hikojima serotype, which expresses both Ogawa and Inaba determinants. Two biotypes of *V. cholerae* O1, 'El Tor' and 'classical', have been described. Classical *V. cholerae* likely caused the first 6 recorded cholera pandemics (Devault et al., 2014) and was more virulent than El Tor *V. cholerae*; however, this *V. cholerae* biotype is now thought to be extinct. The ongoing seventh cholera pandemic, which began in 1961, is caused by El Tor *V. cholerae* (Kaper et al., 1995). In recent years, a 'variant' El Tor strain has emerged and spread extensively (Safa et al., 2010). This strain caused the cholera outbreak in Haiti (Chin et al., 2011), which began in 2010 (Ivers 2017; Luquero 2016). Variant El Tor is now the predominant cause of cholera worldwide (Domman et al., 2017; Ghosh et al., 2014; Kumar et al., 2012; Mutreja et al., 2011; Reimer et al., 2011; Weill et al., 2017; Weill et al., 2019). The genome of the Haitian *V. cholerae* isolate, like that of other recent variants El Tor isolates, includes polymorphisms in CT-B, the SXT integrative conjugative element (ICE), which encodes multiple genes conferring resistance to antibiotics, TCP, and additional virulence-associated genes (Chin et al., 2011; Son et al., 2011; Satchell et al., 2016).

2.1.2 Cholera Vaccines

2.1.2.1 Killed OCV

Two types of killed oral cholera vaccines (OCV) have been developed over the past few decades: a killed whole-cell monovalent (O1) vaccine with CT-B (WC-rBS, Dukoral) and killed modified whole cell bivalent (O1 and O139) vaccines without CT-B (mWC, Shanchol, Euvichol) (World Health Organization 2017).

WC-rBS vaccine is a World Health Organization (WHO) pre-qualified OCV and is currently licensed in >60 countries, but not in the US. A randomized doubled-blind trial in Bangladesh conducted from 1985 to 1990, when El Tor and classical cholera strains co-circulated, showed a protective efficacy (PE) of 85% at 4-6 months post-vaccination (Clemens et al., 1986). During the first, second, and third year of follow-up, the PE dropped to 62%, 58%, and 18%, respectively, against combined El Tor and classical infections, with

For Review Only

slightly lower PE for El Tor (Clemens et al., 1990). For children 2-5 years old, the PE was 100% at 4-6 months but rapidly fell to 38% after one year. Before 2011, WC-rBS was the only WHO pre-qualified OCV. However, its use was not ideal for mass vaccination because it requires a buffer to be dissolved in potable water before administration and it is relatively expensive to manufacture due to the inclusion of recombinant CT-B (Pezzoli et al., 2019).

Shanchol and Euvichol are modified versions of Dukoral that do not contain CT-B. These vaccines have significantly lower manufacturing costs and do not require co-administration with an oral buffer which facilitates their administration (Pezzoli et al., 2019; Desai et al., 2016). In trials in India and Bangladesh, the efficacy of the standard two dose Shanchol regimen varied between 37 - 65% after 2 years of follow-up (Bhattacharya et al., 2013; Qadri et al., 2015). However, in both trials, the vaccine had lower efficacy in children under 5, the population at highest risk for cholera. A single dose of Shanchol had lower PE of 40% after 6 months of follow-up (Qadri et al., 2016). A more recent placebo-controlled study carried out in Bangladesh showed that a single dose of Shanchol conferred 57% protection against cholera at 2 years of follow-up in older children and adults, but no protection in children younger than 5 (Qadri et al., 2018). A study of vaccination with Shanchol during a recent epidemic in Guinea suggested that such 'reactive' vaccination may be feasible and valuable in the setting of an ongoing epidemic (Luquero et al., 2014; Luquero et al., 2013).

Although administration of these killed OCVs will undoubtedly contribute to control of cholera, they all have four important limitations: 1) they are not as effective in children less than 5 years old; 2) they require at least 2 doses for maximum efficacy; 3) they all have modest efficacy; and 4) there is at least a 7-10 day lag between the time of immunization and the onset of protection. To reduce the cost and simplify manufacturing of killed OCVs, a killed whole-cell El Tor Hikojima serotype strain is in clinical development (Hillchol). Phase I/II clinical trials of this vaccine appear promising (Chowdhury et al., 2021), but it will have similar limitations as other killed OCVs. Another potential limitation of all killed OCVs is that they fail to produce *V. cholerae* antigens that are only expressed in the intestine. Such antigens may constitute supplemental targets of protective immunity. For example, TCP is not expressed in the growth conditions used to produce killed OCVs, but antibodies to TCP can confer protection in animal models (Taylor et al., 2004) and may contribute to protection in humans as well (Harris et al., 2008). Furthermore, the heat and formalin killing performed in vaccine preparation does not optimally preserve *V. cholerae*'s native antigens (Kabir 2014).

2.1.2.2 Live Vaccines

Infection with wild-type *V. cholerae* engenders long-lived immunity, and the development of live attenuated OCVs has also been explored, although none are in current use in endemic regions. Analogous to natural infection, such vaccines are expected to prompt immunity after a single dose and to display antigens (such as TCP) that are expressed in vivo but not under ordinary culture conditions. Antibodies to the O1 LPS O-antigen are thought to be the principal basis for immunity following natural infection, but additional *V. cholerae* antigens, including CT-B, also likely contribute to the development of immunity (Kauffman et al., 2016). At present, there are no live OCVs licensed for use in cholera endemic regions. CVD 103-HgR (Vaxchora) has been licensed by the Food and Drug Administration (FDA) for use in adult travelers. CVD 103-HgR, a Δ*ctxA* version of a classical *V. cholerae* strain, was found to be safe and to confer high levels of protection in volunteer challenge studies. A recent trial in Mali showed that a single high-dose ($\geq 2\times10^9$ colony forming units (CFU)) of CVD 103-HgR led to higher rates of seroconversion (measured as serum vibriocidal titers) than a single dose of Shanchol (Sow et al., 2017). The one placebo-controlled trial of CVD 103-HgR carried out in a cholera-endemic area revealed a PE of only 14%, but the trial had significant design challenges (Richie et al., 2000; Harris 2016). However, the use of this vaccine to limit the spread of cholera during an outbreak (reactive vaccination) appears to have contributed to a

For Review Only

decrease in the incidence of cholera (Calain et al., 2004). While CVD 103-HgR's safety and efficacy in volunteer studies have been encouraging, widespread adoption of this live vaccine for use in endemic countries is potentially hazardous. Classical *V. cholerae*, which is thought to have been more virulent than El Tor *V. cholerae*, is now extinct. Thus, the use of this live vaccine allows for the chance of recombination of extinct classical *V. cholerae* genes with those of contemporary variant El Tor strains. In addition, CVD 103-HgR is highly susceptible to reversion mediated by CTXΦ infection.

Peru-15, a ΔCTX prophage, Δ*recA* derivative of a 1991 'traditional' (non-variant) El Tor isolate was likewise found to be safe and to confer protection against challenge with El Tor *V. cholerae* after a single dose in volunteer studies (Cohen et al., 2002; Kenner et al., 1995). Additionally, trials in Bangladesh showed that Peru-15 safely prompted the development of vibriocidal titers likely to be protective against cholera in 77% of vaccinees under 5 (Qadri et al., 2007), including in children as young as 9 months of age. Despite promising early clinical data, the development of Peru-15 is not being actively pursued solely due to business considerations.

CV638, another live attenuated OCV developed from an El Tor strain isolated in 1991, has been shown to be safe and immunogenic in small trials (Garcia et al., 2005). However, this vaccine strain retains the CTXΦ attachment site as well as sequences that could enable the strain to revert to toxigenicity by CTXΦ infection. In addition, this vaccine does not over-express CT-B, eliminating the potential for this vaccine to elicit the formation of anti-CT-B antibodies, which have been shown to confer short-term protection against enterotoxigenic *E. coli* (Clemens et al., 1990). VA1.4, a vaccine developed from a nontoxigenic traditional El Tor also exhibited safety but less robust immunogenicity than Peru-15 (Kanungo et al., 2014); it too remains susceptible to reversion by CTXΦ infection.

2.2 PanChol

2.2.1 Design and Pre-clinical Development

A new live attenuated cholera vaccine is needed because previous vaccines were created in *V. cholerae* strains that are currently extinct (CVD 103-HgR) or no longer prevalent (Peru-15). Also, these vaccines were developed before knowledge that CT is encoded by a mobile element and are therefore capable of CTXΦ-mediated reversion to toxigenicity. PanChol (formerly known as Haiti^{V}), is the first vaccine created in the variant El Tor background that is predominant in the world today. PanChol includes ten different genetic modifications (Appendix 2), and whole-genome sequencing confirmed that all planned mutations are present. Mutations were engineered to minimize the risk of reactogenicity while maintaining PanChol's ability to colonize the intestine so that, like wild-type *V. cholerae,* it may impart long-term immunity after a single oral dose. Engineering steps were introduced to 1) abrogate its capacity to cause cholera-like diarrhea by deleting the genes encoding CT; 2) reduce potential vaccine reactogenicity by deleting *V. cholerae*'s five flagellins (Rui et al., 2010), hemolysin, and MARTX toxin; 2) eliminate the vaccine strain's capacity to transfer genes conferring resistance to antibiotics, which lie within the SXT ICE; 3) enable the vaccine candidate to produce the non-toxic B subunit of CT that is found in current variant El Tor strain (the *ctxB7* allele), an antigen that may elicit protection against diarrheal disease caused by enterotoxigenic *E. coli* as well as *V. cholerae* (Kauffman et al., 2016); 4) minimize potential gene acquisition by deleting *recA*, markedly reducing the strain's capacity for DNA recombination; and 5) minimize the potential for the vaccine to acquire the CTXΦ by deleting the phage's chromosomal attachment site. PanChol also encodes a clustered regularly interspaced short palindromic repeats (CRISPR)/Cas9 system specifically targeting the toxin gene *ctxA*, thereby preventing the strain's capacity to revert to toxigenicity by phage transduction or other means.

PanChol (or the nearly identical Haiti^{V} construct) was found to robustly colonize the SI of infant rabbits for at least 4 days without causing diarrhea or other untoward effects. In marked contrast, wild-type (WT) *V.*

For Review Only

cholerae leads to severe cholera-like diarrhea, with >90% of animals dying within 1 day of oral inoculation in this model (Hubbard et al., 2018). When animals were challenged with lethal doses of WT Ogawa or Inaba strains 24 hours after oral inoculation of PanChol, there was ~1000× reduction in their colonization compared to control animals that had been inoculated with either formalin killed PanChol or buffer. Moreover, there was a marked slowing of disease progression and reduction in mortality in animals pretreated with PanChol vs those inoculated with formalin killed vaccine. In particular, all 8 of 8 animals inoculated with the killed vaccine developed diarrhea (median onset 15 hours) and progressed to a moribund state within 29 hours of inoculation of the WT challenge strain (median 18.8 hours). In contrast, animals inoculated with live vaccine were significantly slower (P<0.01, Log-Rank test) to develop diarrhea (median 28.3 hours; one animal did not develop diarrhea) and showed a marked increase in survival time post lethal challenge (median > 41.3 hours) and in survival time post onset of diarrhea (>13 hours vs. 5 hours in control animals) (Hubbard et al., 2018). Additionally, 4 of 7 animals inoculated with live vaccine had not reached a moribund state when the study was concluded 40 hours post lethal challenge.

Thus, PanChol protects from disease even before the development of adaptive immune responses. Although the mechanisms of this rapid 'probiotic-like' protection, are under investigation, its impact in the context of reactive vaccination during cholera epidemics could be profound. Mathematical modeling revealed that an intervention that works at the speed of PanChol-mediated protection could markedly improve the public health impact of reactive vaccination (Hubbard et al., 2018).

Since *V. cholerae* does not colonize untreated adult mice, PanChol was orally administered to adult germ-free (GF) mice (Ali et al., 2011) as an initial means to evaluate its immunogenicity. Following a single oral dose of PanChol, 10/10 mice developed anti Inaba vibriocidal antibody titers of > 1/1000 (where seroconversion is defined as >4× increase over baseline titers), and 8/10 mice developed anti Ogawa vibriocidal titers of >1/1000 (Luquero and Azman 2018; Sit et al., 2019); in contrast only 1 of 5 animals seroconverted, demonstrating that the killed vaccine is less immunogenic in this model. Vibriocidal titers are a strong clinical correlate of protection in human infections. To assess protection, pups of immunized females were challenged with lethal doses of either Ogawa or Inaba WT strains. There was robust clinical protection from both challenges and 17/17 and 8/12 pups were protected from lethal Inaba WT or Ogawa WT challenge respectively (Sit et al., 2019). Pups challenged with an O139 serogroup toxigenic strain exhibited some clinical protection as well, although there was not a significant reduction in the intestinal burden of this strain (Luquero and Azman 2018). Cross-fostering experiments revealed that essentially all protection in these experiments was derived from the milk (presumably antibodies) of the immunized dams. The observation that animals were protected from challenge despite relatively robust colonization suggests that protection from the disease may result from immunity to diarrheal factors such as CT-B, in addition to those that impede colonization. The capacity of PanChol to induce immune responses to in vivo-expressed antigens, including CT-B, is a property that heightens the appeal of live vs killed OCVs. Collectively, these pre-clinical studies using both infant rabbits and mice have revealed that PanChol appears to be safe and well-tolerated and has the potential to provide both rapid probiotic-like protection as well as to elicit long-term immunity to cholera.

2.2.2 Selection of the optimal PanChol serotype

Based on the knowledge that the O1 O antigen is a critical target of protective immunity against cholera and that the methylation that distinguishes Ogawa from Inaba strains can impact anti-*V. cholerae* immune responses (Ali et al., 2011), genetically matched serotype variants (Inaba, Ogawa, and Hikojima) of PanChol, as well as isogenic Ogawa and Inaba WT challenge strains, were engineered, to determine which, if any, O1 serotype would be the most immunogenic and protective in the germfree mouse OCV immunization model. All three PanChol variants (Inaba, Ogawa, and Hikojima) were both immunogenic and protective in this mouse model. There were only minor differences between the vaccines, and a

For Review Only

consistently single superior vaccine serotype across both the immunogenicity and protection assays was not identified. Since the Hikojima version of PanChol was at least as immunogenic as the Inaba and Ogawa versions of PanChol at inducing vibrocidal antibody responses against Inaba and Ogawa *V. cholerae*, the Hikojima version of PanChol was chosen for further development and manufacturing. This single-strain Hikojima formulation simplifies the manufacture of a bivalent (Ogawa and Inaba) vaccine.

2.3 Therapeutic Rationale

While there has been progress in the clinical evaluation and deployment of killed oral whole-cell cholera vaccines in recent years, these vaccines have several critical limitations. First, current killed vaccines are least effective in children less than five years old, the population at greatest risk of death from cholera (Luquero and Azman 2018). Second, the utility of killed vaccines to quell epidemics ('reactive' vaccination) is inherently hampered by the lag time between vaccination and the development of protective, adaptive immune responses (usually 7-10 days). Third, compared to live-attenuated vaccines, killed vaccines are thought to elicit less long-lived immune responses. This novel live-attenuated cholera vaccine (PanChol) has the potential to circumvent the limitations of current killed vaccines. Our pre-clinical protection and immunogenicity data in two animal models (Hubbard et al., 2018; Sit et al., 2019) coupled with previous clinical trials of a comparable live-attenuated cholera vaccine (Qadri et al., 2007) suggest that PanChol can provide rapid (within a day), single-dose, long-lived protection from cholera in young children as well as in adults. Thus, PanChol could become a transformative tool for global cholera control.

In contrast to all existing cholera vaccines, PanChol has the advantage of being created using a variant El Tor O1 *V. cholerae* strain, which is the globally predominant cause of cholera. PanChol is highly engineered to ensure biosafety for both the vaccinated individual and the community as well as to enhance its genetic stability. Also, in contrast to other live vaccines, it was engineered to be resistant to reversion to toxigenicity, including a CRISPR system that targets the CT gene; furthermore, PanChol is the first bivalent live attenuated OCV. The vaccine strain's genome has been sequenced and a patent application covering its design and sequence has been submitted. In both infant rabbit and infant mouse models, the vaccine confers protection from a lethal *V. cholerae* challenge even before eliciting protective adaptive immune responses (Hubbard et al., 2018). Epidemic modeling suggests that the extremely rapid protection engendered by this vaccine could have a significant impact in reactive vaccine campaigns to limit epidemic spread.

2.4 Rationale for Dose, Regimen and Route of Administration

To determine the starting dose in this trial, previous animal experiments and other cholera vaccine trials were used. In an infant rabbit model, a dose of 10^9 CFU of PanChol did not cause cholera-like illness and was shown to reduce intestinal colonization by the WT challenge strains, slowed disease progression, and reduced mortality in an infant rabbit model of cholera (Hubbard et al., 2018). As mentioned previously, CVD 103-HgR (Vaxchora®) is an FDA-approved live attenuated OCV for use in persons 2 through 64 years of age (Package Insert 2020).

A large Phase 3 randomized (8:1), double-blind, placebo-controlled, multi-center study assessed the safety of CVD 103-HgR at a dose of 10^9 CFU. A total of 3146 participants were enrolled, including 2795 receiving the vaccine. Vaccine reactogenicity was mild and resolved within 1-3 days. Diarrhea and headache were more frequently reported in vaccine recipients. There were no differences in unsolicited adverse events between the groups and no study-related serious adverse events (McCarty et al., 2018). Peru-15 was an El Tor *V. cholerae*-derived live OCV that was shown to be safe in a phase 1/ 2 study of 240 children from Bangladesh (Qadri et al., 2007). The vaccine doses, 2×10^7 CFU or 2×10^8 CFU, were both safe. Side effects included mild symptoms that occurred at a similar rate between participants receiving Peru-15 or placebo. A randomized controlled trial was also conducted in 70 adults (40 in the vaccine group, 30 in the placebo

For Review Only

group) in which 2×10^8 CFU, was shown to be safe, with no cases of fever or diarrhea after vaccination and importantly, no serious adverse events were reported. Vibriocidal antibody responses were seen in 75% of the vaccine recipients (Qadri et al., 2005). Peru-15 pCTB was created by inserting a plasmid carrying the gene for the nontoxic CT-B (*ctxB*) into the parental Peru-15 strain. In the first-in-human study, Peru-15 pCTB was safe and immunogenic in 62 adults at doses ranging from 10^7 to 10^{10} CFU (Chen et al., 2015).

Given these previous studies and to ensure an appropriate safety margin, a starting dose of 10^6 CFU will be used. This dose is 2-3 logs (100-1000×) lower than doses shown to be safe with previous live OCVs. In the first module of the study, the fixed-dose ranging module, the dose will be increased stepwise by factors of 10, in a total of five different cohorts, with a maximum dose of 10^{10} CFU.

2.5 Rationale for Study Duration

This study is a first-in-human, Phase 1 study of the safety, tolerability, and immunogenicity of PanChol in healthy volunteers (Figure 1). There will be two modules in this clinical trial assessing dosing, safety, and immunogenicity:

- A fixed dose-ranging module, and
- A placebo-controlled expansion module.

For Review Only

Figure **1**. Study Design

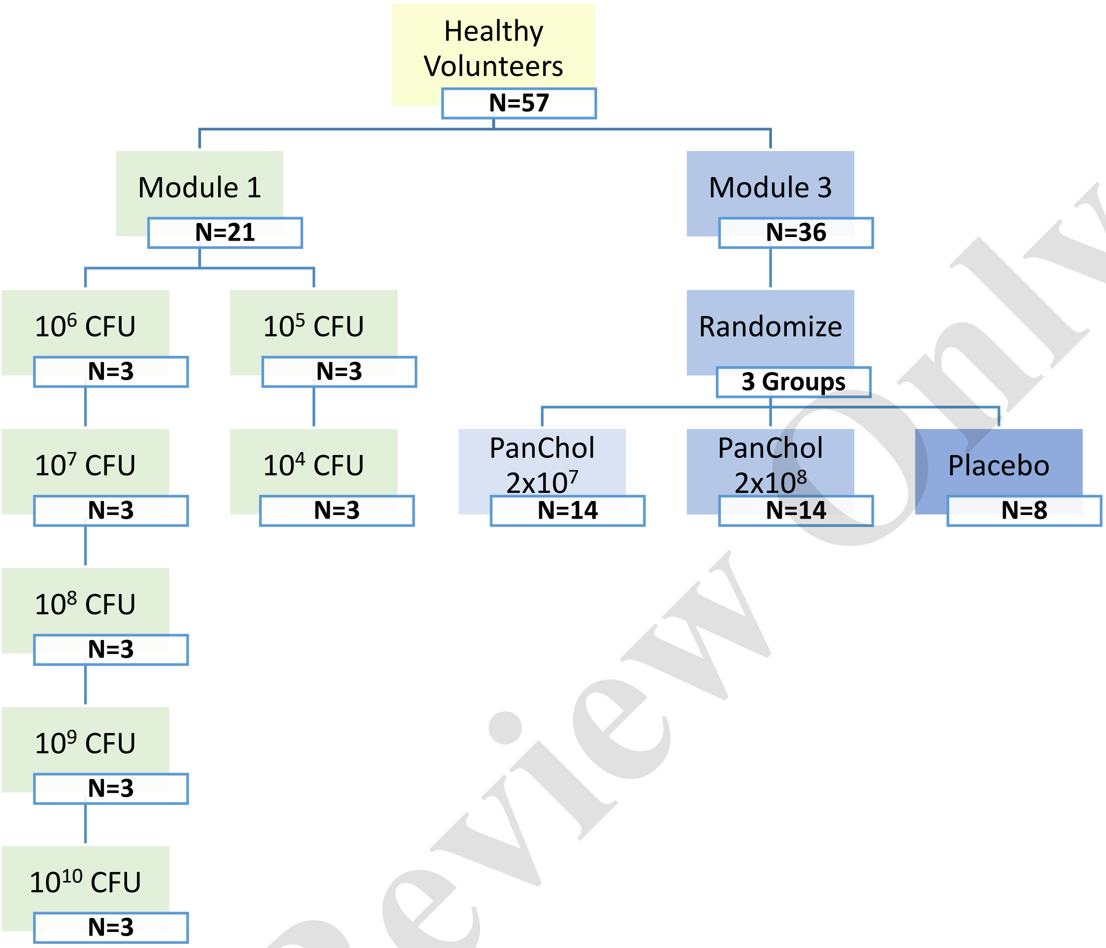

In Module 1, for each cohort, 3 additional participants may be enrolled if one participant experiences a dose-limiting toxicity.

2.5.1 Module 1: Fixed Dose-Ranging

The first, fixed dose-ranging module utilizes a classical "3+3 design". The name is derived from the typical cohort size at a given dose [3], and the typical expansion size at that dose [3] if 1 dose-limiting side effect is observed. This module will address the uncertainty regarding the relationship between dose and adverse events (AEs). A set of pre-specified doses (log10 values 6, 7, 8, 9, and 10) will be employed, based on animal experiments and other live OCV trials. Three participants will be administered each dose (15 participants total). In this module only, there will be a minimum of 48 hours of observation between each participant treated, only one participant will be treated per day, and the doses will be approached in ascending order.

The PSRT will review the 48-hour safety data for all three participants in a given dose-tier before escalating to the next dose-tier. Side effects of Grade 3 or above based on the FDA guidelines for Toxicity Grading Scale for Healthy Adult and Adolescent Volunteers Enrolled in Preventive Vaccine Clinical Trials (Huang

For Review Only

et al., 2015) and judged related to the vaccine will be considered dose-limiting. If the same dose-limiting toxicity (DLT) is experienced by more than 1 participant in a dose cohort, the escalation will cease. If a single individual experience a DLT in a given dose cohort, three more participants will be treated at the same dose level. The escalation continues only if 1 or fewer of 6 participants at that dose experience a DLT. We do not expect to observe serious DLTs at any of the listed doses. If no DLTs are observed, the subsequent double blind and placebo-controlled expansion cohort module will begin at dose $2x10^7$ or $2x10^8$ CFU. If the lowest tested dose 10^6 elicits a sufficient immune response, two additional doses will be tested at dose 10^5 or 10^4 CFU. These lower doses are expected to be safe given that the initial 5 doses were safe and well tolerated. The lower doses will help identify the minimum dose that still elicits a robust immune response. The Data Safety Monitoring Board (DSMB) will review the module following completion of the 48-hour safety data from the final participants. All participants will contribute blood and stool for immune and microbiome profiling respectively. The operating characteristics (OC) for a 3+3 dose-ranging module such as this reveal that it typically recommends conservative doses and often underestimates the optimal dose (Hsiao et al., 2014). For example, if we employ log dose levels of 10^6, 10^7, 10^8, 10^9, 10^{10} and cohort sizes of 3, the design yields a high probability of terminating (red cumulative probability curve in Figure 2 based on 5000 replicates) below the "target" dose assuming the true dose-response curve is as shown in blue.

Figure **2.** Operating Characteristics: Cumulative Probability of Stopping at or Before a Given Dose (Red) With True Probability of Response (Blue) in 3+3 Design

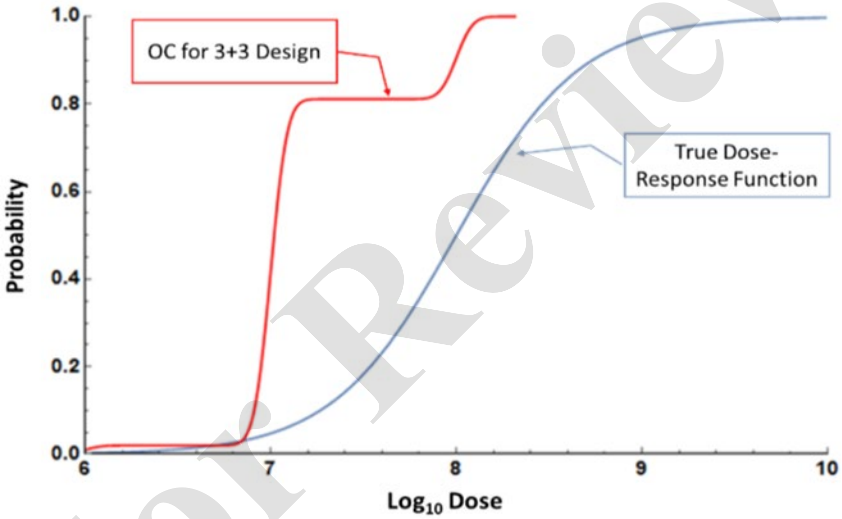

For Review Only

2.5.2 Brief update of Module 1 results (as of September 2024)

A protocol revision has been made given the results of Module 1. In the first module, which tested 5 Panchol doses, 15 adults received the vaccine. No grade 3 or above AEs attributable to the vaccine were observed at any dose. The PanChol strain was detected in the stool of all vaccinees for four days after vaccination, before administration of doxycycline. There was no clear relationship between dose and shedding. The vaccine elicited robust vibriocidal antibody titers against both Ogawa and Inaba serotype target cells at all doses. Luminex-based assays found that all vaccinees generated specific antibodies against the Ogawa and Inaba O-antigen specific polysaccharides and the B-subunit of cholera toxin.

2.5.3 Rational for Not Needing Module 2 and Dose Selection for Module 3

This protocol revision is based on results from Module 1, in which it has been observed that the dose-toxicity response is flat over 5 orders of magnitude. No serious adverse events were observed at any dose while there were definitive immune responses. A result of these findings is that Module 2 (refined dose finding) is not necessary. In Module 1, 15 subjects had at least a dose of log 6 with no SAEs. Similarly 12 subjects had at least a dose of log 7 with no SAEs, and so on. Corresponding one sided upper 95% confidence bounds are approximately .2, .25, .33, and .5 for log doses 6, 7, 8, and 9. These bounds are conservative because each does not fully utilize information from higher doses. In any case, the need for Module 2 is obviated because there is no adverse response to titrate using refined dose finding. Hence the protocol is revised to proceed directly to a revised Module 3. Because of the flat and zero dose response from Module 1, two doses have been selected for Module 3 based on a combination of clinical/biological considerations and potential manufacturing and field logistics. These chosen log doses are 7 and 8, emphasizing that these are within the range already tested.

2.5.4 Module 2 – No longer needed

2.5.5 Module 3: Expansion Cohort

Given the findings in Module 1, Module 3 will assess PanChol at 2 doses in an additional 28 participants. The purpose of the expansion cohort is to gather additional clinical experience at the optimal dose and increase the precision with which the rate of AEs is estimated. The expansion module will be randomized, double blind and placebo-controlled (28 receiving active product, 8 receiving placebo, 36 participants total). The placebo recipients will provide the laboratory with blinded immunogenicity and microbiome samples as well as providing a less biased estimate of vaccine reactogenicity; i.e., a measure of side effects with the buffer alone. This small number of placebo recipients has been deemed appropriate for early phase vaccine studies (Huang et al., 2015).

2.5.6 Rationale for adding two doses to Module 1 for dose de-escalation

Given the results of Module 1, which found that all five doses tested (10^6, 10^7, 10^8, 10^9, 10^{10}) elicited robust vibriocidal antibody titers against both Ogawa and Inaba serotype target cells, two additional lower doses will be tested. Module 1 did not find a dose-response relationship, likely explained by the ability of the vaccine strain to rapidly colonize and replicate in the small intestine. The dose de-escalation will evaluate whether these two additional doses are also sufficient to produce a robust immune response. Consistent with the initial 5 doses, the expansion of module 1 will be unblinded without a control group (3 participants receiving each dose for a total of 6 additional participants).

2.5.7 Duration of Treatment

For the first days of the trial, participants will be inpatients at the Center for Clinical Investigation (CCI) in BWH, for optimal safety monitoring and for fecal and blood samples collection. PanChol or placebo will be administered as a single dose on Day 1. On ~Day 5, participants will be starting doxycycline to eradicate the shedding of the vaccine organisms. On Day 7, those who are no longer excreting PanChol in their stool will be discharged.

2.5.8 Duration of Post Treatment Follow-up

After discharge, volunteers will return on days 15, 29, 57, 85, and 180 for monitoring of general health, AE assessment, immune responses, and fecal microbiota

For Review Only

2.6 Investigational Drug Product PanChol

2.6.1 Mechanism of Action

A new live attenuated cholera vaccine is needed because previous vaccines were created in *V. cholerae* strains that are currently extinct (CVD 103-HgR) or no longer prevalent (Peru-15). Also, these vaccines were developed before knowledge that CT is encoded by a mobile element and are therefore capable of CTXΦ-mediated reversion to toxigenicity. PanChol (formerly known as Haiti^{V}), is the first vaccine created in the variant El Tor background that is predominant in the world today. PanChol includes ten different genetic modifications (Appendix 2), and whole-genome sequencing confirmed that all planned mutations are present. Mutations were engineered to minimize the risk of reactogenicity while maintaining PanChol's ability to colonize the intestine so that, like wild-type *V. cholerae*, it may impart long-term immunity after a single oral dose. Engineering steps were introduced to 1) abrogate its capacity to cause cholera-like diarrhea by deleting the genes encoding CT; 2) reduce potential vaccine reactogenicity by deleting *V. cholerae*'s five flagellins (Rui et al., 2010), hemolysin, and MARTX toxin; 2) eliminate the vaccine strain's capacity to transfer genes conferring resistance to antibiotics, which lie within the SXT ICE; 3) enable the vaccine candidate to produce the non-toxic B subunit of CT that is found in current variant El Tor strain (the ctxB7 allele), an antigen that may elicit protection against diarrheal disease caused by enterotoxigenic *E. coli* as well as *V. cholerae* (Kauffman et al., 2016); 4) minimize potential gene acquisition by deleting recA, markedly reducing the strain's capacity for DNA recombination; and 5) minimize the potential for the vaccine to acquire the CTXΦ by deleting the phage's chromosomal attachment site. PanChol also encodes a CRISPR/Cas9 system specifically targeting the toxin gene ctxA, thereby preventing the strain's capacity to revert to toxigenicity by phage transduction or other means.

Given the potential benefits of live-attenuated vs killed OCVs, the Waldor laboratory has developed a new second generation live-attenuated OCV, PanChol, that circumvents limitations of previous live-attenuated OCVs. In contrast to other live OCVs, PanChol is derived from a current circulating pandemic *V. cholerae* strain, thus eliminating the risk of vaccine-derived genes from extinct *V. cholerae* strains (such as CVD 103-HgR) recombining with circulating *V. cholerae* strains. Furthermore, PanChol is highly engineered to increase its genetic stability. The vaccine's potential for recombination has been greatly reduced by deletion of *recA* and its capacity for reversion to toxigenicity has been eliminated by several steps including elimination of the CTXΦ attachment site and a CRISPR system that targets *ctx*A. Additional engineering steps were designed to reduce its potential to be reactogenic. Finally, the vaccine was engineered to express both the Inaba and Ogawa *V. cholerae* serotypes. Data in pre-clinical murine and rabbit models suggest that the vaccine is well tolerated and immunogenic and has the potential to provide extremely rapid protection from cholera.

2.6.2 Drug Substance PanChol

The attenuated *Vibrio cholerae* strain PanChol is a live, attenuated derivative of *Vibrio cholerae* H1 isolated in Haiti in 2010. The bacterial isolate was shipped to the United States, with the use of an import license for this purpose (2010-10-108) that was provided through the Centers for Disease Control and Prevention (CDC) (Chin et al., 2011). Isolates were identified as *V. cholerae* at the Massachusetts General Hospital clinical microbiology lab and were determined to be susceptible to tetracycline and erythromycin but resistant to trimethoprim-sulfamethoxazole and nalidixic acid. Targeted mutations were created in the KW3 isolate as described below (Hubbard et al., 2018).

First, the CTXΦ prophage and surrounding sequences were deleted using primers TDPsCTX1/TDPsCTX2 and TDPsCTX3/TDPsCTX4 to amplify homologous regions upstream of the rtx toxin transporter at the 5′ end and upstream of a putative dehydrogenase on the 3′ end of this region. This results in a deletion of a

For Review Only

42,650-bp fragment that includes the entire CTX prophage, which includes *ctxAB*, the CTX attachment site, the RS1 and TLC satellite prophages, and the MARTX toxin genes *rtxABCDE*. The knockout was validated via polymerase chain reaction using primers TD1027/TDP1028.

Next, the *flaBDE* operon was deleted as previously described using pCVD442 derivatives (Millet et al., 2014; Rui et al., 2010). The *flaAC* operon deletion plasmid was constructed using primers TDP1172/1174 (upstream homology) and TDP1173a/TDP1173 (downstream homology). The SXT ICE–encoded antibiotic resistance loci *dfrA*, *sul2*, *strAB*, and *floR* were then deleted using primers TDP1193/TDP1194 + TDP1195/1196 (*dfrA*, trimethoprim resistance) and TDP1287/TDP1288 + TDP1291/TDP1292 (sulfamethoxazole, streptomycin, and chloramphenicol resistance loci). Whole-genome sequencing revealed that the second crossover in the allele exchange process occurred not between the homologous regions included in the suicide plasmid, but rather between duplicate sequences flanking the *flor/sul* region of the chromosome (N900_11210 and N900_11260). An SmR mutant of the vaccine precursor strain was isolated by plating on streptomycin (1000 µg/ml), and the *rpsL*K43R single nucleotide variant was confirmed by Sanger sequencing.

For CtxB overexpression, the htpG promoter was amplified from V. cholerae Peru-15 (Kenner et al., 1995) using primers FD54/FD103 (adding the strong ribosome binding site AGGAGG), and ctxB was amplified from HaitiWT, which contains the ctxB7 allele, using primers FD33/FD34. Homologous regions flanking the intergenic region of the validated neutral locus vc0610/N900_11550 (Abel et al, 2015) were amplified with pairs FD30/FD31 and FD73/FD74. These fragments were then cloned into pCVD442 in a one-step isothermal assembly reaction. CtxB overexpression was confirmed by Western blot on cell-free supernatants from cultures grown in AKI conditions (Iwanaga et al., 1986) using antisera to CT (Abcam, ab123129, anti-cholera toxin).

For the *cas9*-sgRNA module, *cas9* was amplified from plasmid DS_SpCas9 (www.addgene.org/48645/), with primers TDP1747/TDP1748. The sgRNA region was amplified from gBlock "VC_3x_sgRNA_gBlock", with primers TDP1761/TDP1762. Both fragments were combined and cloned in to the StuI site of pJL1 (Butterton et al., 1995) via isothermal assembly, then integrated into the lacZ gene creating a LacZ- strain. A majority of the *hlyA* gene was deleted using the suicide vector pCWΔ*hlyA* (Fullner et al., 2002), introduced to the vaccine strain via triparental mating with the helper plasmid pRK600.

The penultimate step in strain construction was to create a stable Hikojima serotype. The S158F point mutation was introduced in the *rfbT* gene using primers GBP116, GBP117, GBP118, and GBP119, then cloned into the pDS132 suicide vector. The point mutation was integrated into the vaccine strain and confirmed by verifying the *rfbT* gene sequence and serotyping with a slide agglutination assay against both anti-Inaba and anti-Ogawa serum.

Finally, a *recA* deletion plasmid was constructed using primer pairs Vc-recA5-F1/Vc-recA5-R1 and Vc-recA3-F1/Vc-recA3-R1; the deletion was verified with primers Vc-recA-SF2/Vc-recA-SR2. *recA* was deleted by homologous recombination resulting in the final strain, named PanChol for Pandemic *Vibrio cholerae* on Sept 7, 2018 (Sit et al., 2021).

2.6.3 Drug Product PanChol

PanChol is formulated as a frozen liquid suspension of approximately 2.6×10^9 cells/mL in cryopreservative solution (minimal media plus 5% sucrose). After removal from the freezer, vials of PanChol are thawed at

For Review Only

room temperature then diluted to the desired concentration in sodium bicarbonate buffer. The sodium bicarbonate buffer (2.5 g sodium bicarbonate [USP], 1.6 g ascorbic acid [USP], and 0.2 g lactose [NF] dissolved in 100 mL sterile water for irrigation) is prepared for each volunteer and is intended to reduce or neutralize stomach acid. PanChol bacterial suspension diluted in sodium bicarbonate buffer should be consumed no more than three hours after the PanChol vial is diluted in the delivery buffer. The components of PanChol are shown in Table 1.

Table 2. Quantitative Composition of PanChol Drug Product

Ingredient	Quantity per dose	Function
PanChol	Varying: 1×10^{4}-1×10^{10} cfu	Drug Substance
Sodium bicarbonate	2.5g	Buffer
Ascorbic acid	1.6g	Buffer
Lactose (anhydrous)	0.2g	Excipient

2.6.4 Preclinical Experience

The Waldor lab has evaluated the safety, immunogenicity and PE of PanChol in rabbit and murine models. Infant rabbits and infant mice and adult mouse models of cholera were used to test vaccine safety and two adult mouse models coupled with infant mouse challenges were used to test vaccine immunogenicity and PE. In these models, PanChol was well-tolerated, and no mortality was observed. In contrast, there was nearly 100% mortality of infant rabbits and infant mice given an equivalent dose of the wild-type (WT) strain from which PanChol was derived. These studies also showed that PanChol is highly immunogenic and protects infant mice from lethal challenge with a variety of WT *V. cholerae* strains. In addition, PanChol induces protection from lethal challenge even before the onset of adaptive immunity when administered 24 hours prior to challenge in infant rabbits.

Adult GF mice were used to study HaitiV immunogenicity. In contrast to normal adult mice, which are resistant to *V. cholerae* intestinal colonization, oral inoculation of GF mice with *V. cholerae* results in stable intestinal colonization without adverse effects (Crean et al., 2000). In the GF model, serum markers of immunity, such as vibriocidal titers, can be measured, but challenge studies are not possible due to the persistent colonization of the vaccine strain and the resistance of adult mice to diarrheal disease. Besides measuring serum markers in the orally vaccinated adult mice, neonatal pups (which are sensitive to *V. cholerae* induced diarrheal disease) born to these mice were subjected to challenge studies to evaluate vaccine PE.

PanChol (or the nearly identical HaitiV construct) was found to robustly colonize the SI of infant rabbits for at least 4 days without causing diarrhea or other untoward effects. In marked contrast, wild-type (WT) *V. cholerae* leads to severe cholera-like diarrhea, with >90% of animals dying within 1 day of oral inoculation in this model (Hubbard et al., 2018). When animals were challenged with lethal doses of WT Ogawa or Inaba strains 24 hours after oral inoculation of PanChol, there was ~1000× reduction in their colonization compared to control animals that had been inoculated with either formalin killed PanChol or buffer. Moreover, there was a marked slowing of disease progression and reduction in mortality in animals pretreated with PanChol vs those inoculated with formalin killed vaccine. In particular, all 8 of 8 animals inoculated with the killed vaccine developed diarrhea (median onset 15 hours) and progressed to a moribund state within 29 hours of inoculation of the WT challenge strain (median 18.8 hours. In contrast, animals

For Review Only

inoculated with live vaccine were significantly slower (P<0.01, Log-Rank test) to develop diarrhea (median 28.3 hours; one animal did not develop diarrhea) and showed a marked increase in survival time post lethal challenge (median > 41.3 hours) and in survival time post onset of diarrhea (>13 hours vs. 5 hours in control animals) (Hubbard et al., 2018). Additionally, 4 of 7 animals inoculated with live vaccine had not reached a moribund state when the study was concluded 40 hours post lethal challenge. Thus, PanChol protects from disease even before the development of adaptive immune responses. Although the mechanisms of this rapid 'probiotic-like' protection, are under investigation, its impact in the context of reactive vaccination during cholera epidemics could be profound. Mathematical modeling revealed that an intervention that works at the speed of PanChol-mediated protection could markedly improve the public health impact of reactive vaccination (Hubbard et al., 2018).

Since *V. cholerae* does not colonize untreated adult mice, PanChol was orally administered to adult GF mice (Ali et al., 2011) as an initial means to evaluate its immunogenicity. Following a single oral dose of PanChol, 10/10 mice developed anti Inaba vibriocidal antibody titers of > 1/1000 (where seroconversion is defined as >4× increase over baseline titers), and 8/10 mice developed anti Ogawa vibriocidal titers of >1/1000 (Luquero and Azman 2018; Sit et al., 2019); in contrast only 1 of 5 animals seroconverted, demonstrating that the killed vaccine is less immunogenic in this model. Vibriocidal titers are a strong clinical correlate of protection in human infections. To assess protection, pups of immunized females were challenged with lethal doses of either Ogawa or Inaba WT strains. There was robust clinical protection from both challenges and 17/17 and 8/12 pups were protected from lethal Inaba WT or Ogawa WT challenge respectively (Sit et al., 2019). Pups challenged with an O139 serogroup toxigenic strain exhibited some clinical protection as well, although there was not a significant reduction in the intestinal burden of this strain (Luquero and Azman 2018). Cross-fostering experiments revealed that essentially all protection in these experiments was derived from the milk (presumably antibodies) of the immunized dams. The observation that animals were protected from the challenge despite relatively robust colonization suggests that protection from the disease may result from immunity to diarrheal factors such as CT-B, in addition to those that impede colonization. The capacity of PanChol to induce immune responses to in vivo-expressed antigens, including CT-B, is a property that heightens the appeal of live vs killed OCVs. Collectively, these pre-clinical studies using both infant rabbits and mice have revealed that PanChol appears to be safe and well-tolerated and has the potential to provide both rapid probiotic-like protection as well as to elicit long-term immunity to cholera.

In preclinical testing in two mouse models, the vaccine did not elicit adverse effects and yielded robust protective immune responses. Moreover, in an infant rabbit model, PanChol confers protection from lethal *V. cholerae* challenge even before eliciting adaptive immune responses.

2.6.5 Clinical Experience

PanChol has not yet been tested in humans.

2.7 Risk-Benefit Assessment

2.7.1 Potential Risks

For a full discussion of the potential risks associated with PanChol treatment please refer to the **Guidance for the Investigator section of the Investigator's Brochure**. A summary of potential mitigation for these risks of treatment with PanChol together with mitigation for these risks.

The safety profile of PanChol has not been established as this will be the first-in-human study of the product. However, given the safety of PanChol in infant rabbits and infant mice and the safety of other very similar live-attenuated OCV such as CVD 103-HgR and Peru-15, major side effects of PanChol are not expected. Minor side effects such as abdominal pain, nausea/vomiting, lack of appetite, diarrhea, and headache may

For Review Only

be observed. Cholera-like diarrhea is not expected because PanChol does not produce CT, the *V. cholerae* virulence factor that accounts for the severe secretory diarrhea characteristic of cholera (Levine et al., 1988). Moreover, this protocol provides many risk management strategies including optimal inpatient monitoring after dose administration and fluid and doxycycline administration in case of reactive diarrhea. There is also a theoretical risk of transmission of PanChol organisms to close contacts. However, this risk is likely to be very low because PanChol will be administered to inpatient volunteers that will only be discharged once confirmation of negative stool cultures has been obtained.

2.7.2 Potential Benefits

This is the first study of PanChol so it is not anticipated that there will be any direct benefits to subjects participating in the study. The proposed study will generate critical knowledge regarding the safety, immunogenicity, and duration of shedding of a novel live attenuated cholera vaccine. We expect to identify a vaccine dose that is minimally reactogenic but sufficiently immunogenic to proceed with challenge studies as well as phase II studies in a cholera endemic region. If we learn that the vaccine is too reactogenic in humans, we will engineer additional attenuating mutations based upon our extensive knowledge of the in vivo growth requirements of *V. cholerae*. We will also learn how the live vaccine modifies the microbiota and triggers the formation of MBC. This knowledge will be invaluable in guiding the future development of PanChol.

2.8 Ethical Considerations

There are no ethical considerations.

For Review Only

3 EXPERIMENTAL PLAN

3.1 Compliance Study

The study will be conducted in compliance with the clinical study protocol, International Conference on Harmonization (ICH), Good Clinical Practices (GCP) as outlined in ICH E6 (R2) as well as the demands of national drug and data protection laws and all applicable local and national regulatory requirements.

3.2 Study Design

This study is a first in human (FIH), Phase 1 study of the safety, tolerability, and immunogenicity of PanChol in healthy volunteers. There will be two modules in this clinical trial assessing dosing, safety, and immunogenicity: 1) a fixed dose-ranging module, and 2) a placebo-controlled expansion module. Participants will be enrolled at the Brigham and Women's Hospital (BWH). For the first days of the trial, participants will be inpatients at the CCI in BWH for optimal safety monitoring and for fecal and blood samples collection. PanChol or placebo will be administered on Day 1. On ~Day 5, participants will be starting doxycycline to eradicate the shedding of the vaccine organisms. On Day 7, those who are no longer excreting PanChol in their stool will be discharged. After discharge, volunteers will return on days 15, 29, 57, 85, and 180 for monitoring of general health, AE assessment, immune responses, and fecal microbiota composition.

3.2.1 Module 1: Fixed Dose-ranging

The first, fixed dose-ranging module utilizes a classical 3+3 design (Table 2):

Table 3. Module 1 design

Module 1 (n=21)	Dose: CFU
Cohort 1 (n=3)	$\sim10^6$
Cohort 2 (n=3)	$\sim10^7$
Cohort 3 (n=3)	$\sim10^8$
Cohort 4 (n=3)	$\sim10^9$
Cohort 5 (n=3)	$\sim10^{10}$
Cohort 6 (n=3)	$\sim10^5$
Cohort 7 (n=3)	$\sim10^4$

*For each cohort, 3 additional participants may be enrolled if one participant experiences a dose-limiting toxicity .

Cohorts 1 to 5 will each have 3 participants who will be administered a single dose ranging from 10^6 to 10^{10} CFUs for a total of 15 participants. There will be a minimum of 48 hours of observation between each participant treated, only one participant will be treated per day, and the doses will be approached in ascending order. The PSRT will review the 48-hour safety data for all three participants in a given dose-tier before escalating to the next dose-tier. Side effects of Grade 3 or above based on the FDA guidelines for Toxicity Grading Scale for Healthy Adult and Adolescent Volunteers Enrolled in Preventive Vaccine Clinical Trials (https://www.fda.gov/media/73679/download) and judged related to the vaccine will be

For Review Only

considered dose-limiting. If the same DLT is experienced by more than 1 participant in a dose cohort, the escalation will cease. If a single individual experiences a DLT, three more participants will be treated at the same dose level. The escalation continues only if 1 or fewer of 6 participants at that dose experience a DLT. The DSMB will review the module following completion of the 48-hour safety data from the final participants. All participants will contribute blood and stool for immune and microbiome profiling respectively.

Cohort 6 and 7 will each have 3 participants who will be administered a single dose of either 10^5 or 10^4 CFUs for a total of 6 additional participants. As there were no dose limiting adverse events in cohorts 1-5 there will be no limitation on enrollment timing and the two dose de-escalations can be tested concurrently.

3.2.2 Module 3: Expansion Cohort

The expansion cohort module will employ the recommended doses from the module 1 to better define the PanChol dose level to take into later phase clinical trials.

Table 3. Module 3 design

**Module 3 (n=36)**	
**Randomized Treatment**	
PanChol at $\sim 2\times10^7$ and $\sim 2\times10^8$	Placebo
n=14 per dose tier	n=8

The purpose of the expansion cohort is to gather additional clinical experience at the optimal dose and increase the precision with which the rate of AEs is estimated. The expansion module will be a double-blind, randomized placebo-controlled trial that will include 8 participants randomized to placebo (36 participants total) (3). This design will provide blinded immunogenicity and microbiome samples as well as providing a less biased estimate of vaccine reactogenicity; i.e., a measure of side effects with the buffer alone. This small number of placebo recipients has been deemed appropriate for early phase vaccine studies (Huang et al., 2015).

3.3 Measures to Minimize/Avoid Bias

The following measures are included in the study design to minimize/avoid bias:

- Randomization for Module 3 to ensure no bias in treatment allocation
- Site staff, subjects and Sponsor blinded to treatment allocation to ensure objectivity in study assessments
- Pre-specification of critical study endpoints in the Statistical Analysis Plan

3.4 Quality Control and Quality Assurance

Quality control (QC) and quality assurance (QA) measures include:

- On-site and remote monitoring of the site by the Sponsor (or designee) with source data verification according to the Safety Monitoring Plan to ensure compliance with protocol and accuracy of data collection transcription
- Remote Sponsor (or designee) review of study protocol compliance and study data with issuance of data queries to correct errors as appropriate

For Review Only

- Audit of site as required
- Retraining of site staff as required

3.5 Number of Sites

This study will be conducted at one center (BWH) in Boston, Massachusetts, USA.

3.6 Number of Subjects

Approximately, 57 adult healthy volunteers are planned to be enrolled in this study if all planned treatment groups are conducted.

3.7 Overall Study Duration and Follow-up

The study comprises an up to 56-day Screening Period, an inpatient stay of at least 6 days, and an outpatient follow-up period. The length of each subject's participation is approximately 24 weeks from screening to last study visit. Baseline evaluations will be performed pre-dose on Day 1. Baseline stool may be collected up to 10 days pre-dose or on Day 1. In addition, subjects may be required to attend additional visits for monitoring of adverse events or abnormal investigation results.

3.7.1 Screening

Study eligibility for this study will be determined within 56 days prior to study Day 1.

3.7.2 Study Drug Administration and Inpatient Period

<u>For All Modules</u>

Participants will be enrolled at BWH. For the first days of the trial, participants will be inpatients at the CCI at BWH for optimal safety monitoring and for fecal and blood samples collection. PanChol or placebo will be administered on Day 1. Participants will be required to fast 60 minutes before and after vaccine administration. On Day 5, participants will be starting doxycycline to eradicate the shedding of the vaccine organisms. On Day 7, those who are no longer excreting PanChol in their stool will be discharged.

For Review Only

3.7.3 Outpatient Follow-up

For All Modules

After discharge, volunteers will return on days 15, 29, 57, 85, and 180 for monitoring of general health, AE assessment, immune responses, and fecal microbiota composition.

3.8 End of Study

The End of Study is last subject, last visit. On their completion of the study, including any subjects that terminate from the study before completion, subjects will be told to inform the Investigator if they develop any symptoms so that they can be adequately followed up.

3.9 Protocol Safety Review Team

The PSRT will be made up of the investigators, study clinicians, and biostatistical staff. Safety data from this clinical trial will be reviewed by the PSRT and they will be responsible for recommending decisions to dose escalate or expand cohorts. The PSRT will also review all AEs that potentially meet the stopping rules to confirm if study stopping rules have been met and in the event of a stopping rule being met will review safety data from all subjects to determine whether it is safe to resume dosing in cohorts at dose levels at which the study stopping rule was met or whether the trial should be terminated depending on the finding and safety outcome.

3.10 Data Safety Monitoring Board

Safety oversight will be conducted by a DSMB that is an independent group with expertise to interpret data from this study and will monitor participant safety. The DSMB members will be separate and independent of study personnel participating in this study and should not have scientific, financial, or other conflicts of interest related to this study.

The DSMB will review data following completion of the 48-hour safety data from the final participants of Module 1 and will conduct ad hoc reviews as appropriate when a halting rule is met or for immediate concerns regarding observations during this study. Additional data, interim statistical reports, or the unblinding of a treatment assignment (for Module 3) may be requested as deemed necessary for the DSMB review of participant safety or any concerns with the study. After each meeting, the DSMB will make recommendations on the continuation of the study.

3.11 Dose Escalation and Initiation of Expansion Cohorts

For Module 1, dose escalation may proceed when all subjects in the preceding cohort have been administered the study drug and the 48-hour safety evaluations demonstrate an acceptable safety profile by the PRST.

3.12 Study Stopping Rules

3.12.1 Study Pausing Rules

Enrollment will be paused and an *ad hoc* DSMB safety review will be triggered if one or more of the following criteria are met:

1) One participant experiences a treatment-related serious adverse event (SAE)
2) If three or more participants experience the same treatment-related Grade 3 or higher AE

For Review Only

3.12.2 Dose Escalation Pausing Rules

3.12.2.1 Module 1

Treatment-related Grade 3 or higher AE will be evaluated by PSRT as potentially dose-limiting. If the same dose-limiting toxicity (DLT) is experienced by more than 1 participant in a dose cohort, the escalation will cease. If a single individual experiences a DLT, three more participants will be treated at the same dose level. The escalation continues only if 1 or fewer of 6 participants at that dose experience a DLT.

3.12.2.2 Module 3

N/A

For Review Only

4 SUBJECT ENROLLMENT

4.1 Screening

Recruitment for this study will be through the prior IRB approved vaccine-screening protocol (IRB #2002-P-000343) and directly through this trial. Recruitment materials will be used to engage interested and eligible individuals, which will be managed through the prior IRB approved vaccine-screening protocol (IRB #2002-P-000343). The MassGeneral Brigham Rally volunteer portal will also be used to recruit participants. Participants will sign the consent form, approved under the vaccine-screening protocol (IRB #2002-P-000343), for this general pre-screening protocol to undergo screening procedures. Screening procedures include performing laboratory tests, collecting medical history, and performing physical examinations. Those individuals determined to be eligible, based on the inclusion and exclusion criteria, will be enrolled in the study and will sign the study specific consent form.

Before subjects may be enrolled into the study, the Sponsor or designee requires a copy of the Site's written independent institutional review board (IRB) approval of the protocol, informed consent form (ICF), and all other subject information and/or recruitment material, as applicable.

Subjects must sign the consent form before any screening tests or assessments are performed. At the time of consent, the subject will be assigned a unique screening number before any study procedures, including Screening procedures are performed. At the time of enrollment, subjects will be assigned a unique subject identification number (which may be the same as the screening number). This number will be used to identify the subject throughout the trial and must be used on all study documentation related to this subject. The screening number and the subject identification number must remain constant throughout the entire trial. Screening numbers and subject identification numbers once assigned, will not be re-used. Our site will be organizing Pre-Screens via phone call.

At this visit, we will:

- Ask subjects about their medical history.
- Take subject's height and weight and perform a physical exam
- Draw a blood sample to perform routine blood test and to test for HIV infection. We may draw about 2 tablespoons of blood.
- Collect urine sample to perform routine analysis and to test for pregnancy, if they are a female who can become pregnant.
- Participant will be asked to provide a stool sample. This can either be in clinic or at home and brought to clinic prior to dosing of vaccine.

4.2 Randomization

For Module 3 subjects will be randomized after all Screening assessments have been completed and after the Investigator has verified that they are eligible per criteria in Sections 5.1 and 5.2. No subject may begin treatment prior to randomization and assignment of a unique subject identification number. Subjects will be randomized to receive PanChol or placebo.

4.3 Replacement of Subjects

Subjects who withdraw or are withdrawn from this study or are lost to follow-up after signing the ICF may be replaced.

For Review Only

4.4 Blinding of Treatment Assignment

The Sponsor and all subjects, monitors and Site personnel related to the study will be blinded throughout Module 3, except for the pharmacist (or qualified designee) who dispenses/ prepares the Study Drug (PanChol or placebo) and the pharmacy monitor (and pharmacy monitor report reviewer if applicable) who monitors the pharmacy records and procedures.

4.5 Unblinding of Treatment Assignment

If a subject has suffered a Serious Adverse Event (as defined in Section 9.3.3) and/or when knowledge of the treatment assignment will impact the clinical management of the subject, the Investigator will have the ability to unblind the treatment assignment for that subject by reference to the randomization scheme held by the study site unblinded pharmacist. The Sponsor or designee will be informed of the unblinding of a subject within 24 hours. An unblinded randomization scheme will be maintained securely by the study statistician.

Every reasonable attempt should be made to complete the Early Termination (ET) study procedures and observations (see Appendix 1) prior to unblinding as knowledge of the treatment arm could influence subject assessment.

The Sponsor may also request unblinding of individual subjects if this required for adequate assessment of safety, in which case the unblinding code will be requested. Treatment assignment information will not be shared with the Study Team unless the knowledge of the treatment assignment is necessary for the clinical management of the subject. The Sponsor, Sub-investigators and all other blinded site staff involved in the day to day conduct of the clinical trial will remain blinded until all subjects in Module 3 have completed their last study visit.

For Review Only

5 SUBJECT ELIGIBILITY

To be eligible to participate in this study healthy volunteers must meet the following criteria within 56 days of Study Day 1 or at the time point specified in the individual eligibility criterion listed.

5.1 Inclusion Criteria

1. Must have given written informed consent (signed and dated) and any other authorizations required by local law and be able to comply with all study requirements.
2. Healthy adults aged from 18 to 55 years old.
3. Considered healthy, as judged by the clinical investigator, according to medical history, physical examination, vital signs, screening laboratories, and medication history.
4. Capable of understanding, consenting, and complying with the entire study protocol including the inpatient period.
5. Female participants must be non-pregnant and non-lactating and either
    a. surgically sterile (history of bilateral ligation, bilateral salpingectomy, bilateral oophorectomy, total hysterectomy) or postmenopausal (defined as as amenorrhea for at least 12 consecutive months before screening without an alternative medical cause)
    b. be of child-bearing potential and practicing an acceptable method of contraception or abstaining from all activities that could result in pregnancy for at least 28 days before vaccination until 3 months after receiving the IP.

    Acceptable methods of contraception include barrier methods (such as condom, diaphragm, or cervical cap used in conjunction with spermicide), intrauterine device, hormonal contraception (that may be taken or administered by oral, intravaginal, transdermal, subdermal or IM route), vasectomized partner (the vasectomized partner should be the sole partner for that participant)

5.2 Exclusion Criteria

1. Confirmed or suspected immunosuppressive condition, as a result of a disease (e.g., primary immune deficiency, malignancy, HIV infection) or have taken any systemic immunosuppressive therapy within 6 months of enrollment.
2. Pregnant or lactating women.
3. History of gastrointestinal (GI) disorder, such as previous major GI surgery, malabsorption, or any chronic GI disorders that would interfere, according to the investigator, with the IP.
4. Acute GI or febrile illness within 7 days of enrollment.
5. Have any acute or chronic medical condition that, in the opinion of the investigator, would make vaccination unsafe or interfere with the evaluation of immune response to study vaccination.
6. History of cholera vaccination.
7. History of cholera infection.
8. Abnormal stool pattern, defined as < 3 or >21 stools per week.
9. Serious allergic reaction to PanChol or placebo component (sodium bicarbonate, lactose, ascorbic acid)
10. Use of any systemic antibiotics within 1 month of PanChol administration.
11. Receipt of a live vaccine in the previous 4 weeks or planned in the 4 weeks following enrollment.
12. Receipt of a killed or subunit (non-live) vaccine in the previous 2 weeks or planned in the 2 weeks following enrollment.

For Review Only

13. Individuals who do not speak English will not be enrolled into this trial. This study involves more than minimal risk and no prospect of direct benefit for participants. Additionally, a subject who did not speak English may not be able to easily communicate safety concerns in a timely fashion to the study investigators
14. Childcare workers with direct contact with children ≤ 2 years of age
15. Individuals whose occupation involves handling of food
16. Healthcare workers who have direct contact with patients who are immunodeficient, HIV-positive, or have an unstable medical condition
17. Use laxatives regularly
18. Have diarrhea within 48 hours before enrollment
19. Have a history of hypersensitivity to any of the tetracyclines
20. Have a history of hypersensitivity to streptomycin or any aminoglycoside due to the known cross-sensitivity of patients to drugs in this class.
21. Individuals who have a household member who are immunodeficient, HIV-positive, or have an unstable medical condition.

For Review Only

6 STUDY PROCEDURES

6.1 Study Schedule

All required study procedures are outlined in Appendix 1.

Blood, stool and urine samples will be collected regularly throughout the study for safety and immunogenicity analysis as detailed in Appendix 1.

The safety of PanChol will be monitored in an ongoing fashion during the study by the Sponsor, Study Team and the PSRT prior to dose escalation.

6.2 Study Assessments

Timing of study assessments are shown in Appendix 1.

6.3 Screening and Enrollment

Screening will be performed no more than 56 days before the enrollment visit and will include written consent, collection of demographics, medical history as well as physical examination (including vital signs), laboratory tests, and other assessments according to Appendix 1.

6.4 Planned Study Visits

6.4.1 Inpatient period

On Day 1, volunteers will be admitted as inpatients at the CCI in BWH. Each participant will have a private room and private bathroom during the inpatient period of the study. For Module 3 only, eligible participants will be randomized to PanChol or matching placebo within 48 hours prior to IP administration. All participants will receive doxycycline on ~Day 5 to eradicate shedding of vaccine organisms. They will remain as inpatients minimally until Day 7; at that point, if they are no longer excreting PanChol in their stool, they will be discharged. Participants who are still shedding will be discharged once they have at least one negative stool culture. Before discharge, participants will receive counselling on the importance of hand hygiene following discharge to minimize the potential spread of the vaccine to family and close contacts.

6.4.2 Follow-up period

Participants will return to the clinic for in-person assessments (five visits: Day 15, 29, 57, 85, 180) according to the Schedule of Assessment (SoA, Appendix 1).

6.5 Unscheduled Study Visits

Unscheduled visits are permitted at the discretion of the investigator as needed, such as for safety assessment.

6.5.1 Withdrawal from the Study or Discontinuation of the Study Product

This is a single-dose study. However, if a participant discontinues from the study, every attempt should be made to keep the participant in the study and continue to perform the required study-related procedures until stabilization per the investigator. If a participant discontinues from the study before completion of the study at D180, an ET visit should be performed with evaluations and/or procedures outlined in the SoA. If a participant discontinues from the study before the end of the inpatient period, doxycycline will be

For Review Only

administered to eradicate shedding of vaccine organisms and stool cultures will be planned to confirm eradication of the vaccine organism.

6.5.2 Study Termination

If the study is prematurely terminated for any reason, the Investigator will promptly inform the study participants and assure appropriate therapy or follow-up for the participants, as necessary.

6.6 Clinical Evaluations

6.6.1 Medical History

A medical history, including details regarding illnesses, date(s) of onset, and whether condition(s) is currently ongoing, will be collected. Medication history will be collected on all subjects during screening and should be updated as needed prior to dosing and throughout study.

6.6.2 Vital Signs

Vital signs, such as blood pressure, pulse rate, respiratory rate, and temperature (oral), should be measured after the participant has rested comfortably. On Day 1, vital signs should be measured and recorded within approximately 30 minutes pre- and post-dose. On all other visit days, vital signs are only required to be measured and recorded once, if applicable. At 30 minutes post vaccination an assessment will be conducted including vital signs, inquiring whether participant is having any signs and symptoms which may prompt a detailed physical exam by clinicians.

6.6.3 Physical Examination

A full physical examination will be conducted at Screening. This may include general appearance, head/neck, chest/respiratory, heart/cardiovascular, GI/liver/spleen, extremities and skin assessments. A symptom-directed physical examination will be performed on all other visits according to SoA (Appendix 1) and according to Investigator discretion.

6.6.4 Height and Weight

Height and body weight will be measured at screening.

6.6.5 Stool Examination

During the inpatient period, all stools will be examined and graded. The consistency of stool will be graded as normal, loose (readily taking the shape of the container) or watery. Loose and watery stools will be weighed or approximate volume measured. Diarrhea will be graded according to the FDA guidelines for Toxicity Grading Scale for Healthy Adult and Adolescent Volunteers enrolled in Preventive Vaccine Clinical Trials.

6.7 Laboratory Evaluations

6.7.1 Clinical Laboratory Assessments

Clinical laboratory tests, such as complete blood count (CBC) with differential and biochemistry (creatinine, liver enzymes, electrolytes), will be performed at Screening and at Day 1, 2, 4, 7, 15 and 29. There will be a urinalysis and blood group done at screening. In the event of an unexplained clinically relevant abnormal laboratory test occurring after study product administration, the test should be repeated and followed up at the discretion of the investigator until it has returned to the normal range or stabilized, and/or a diagnosis is made to adequately explain the abnormality.

For Review Only

6.7.2 HIV Testing

HIV screening test will be performed at screening.

6.7.3 Pregnancy Test

Urine qualitative β-hCG test will be performed at screening and at Day 1 (for females of child-bearing potential). A point-of-care β-hCG test may be used.

6.7.4 Immunogenicity assessment

6.7.4.1 Antibody measurement

Pre- and post-vaccination antibody measurement, such as titers of vibriocidal antibodies to both Inaba and Ogawa *V. cholerae* will be measured. IgG, IgA, and IgM antibodies targeting Inaba- and Ogawa-specific polysaccharides, cholera toxin B subunit, and TCP, may be performed.

6.7.4.2 Antibody secreting cell response

Pre- and post-vaccination IgA- and IgG-antibody secreting cell responses (ALS/plasmablast responses) may be measured.

6.7.4.3 Memory B cell response

The kinetics of the MBC response to the O1- Inaba, Ogawa, CtxB and TCP antigens may be performed.

6.7.5 Stool culture

Stool culture will be performed at least daily during inpatient period. TCBS agar (selective agar for *Vibrio* spp.) will be used for culture. If there is no stool, a rectal swab may be used to collect a fecal sample.

6.7.6 Fecal microbiota profile

To assess the impact on PanChol on the microbiota composition, stools may be sent for 16s rRNA sequencing. These findings will be compared to studies of changes in the fecal microbiota composition following cholera (Hsiao et al., 2014). DNA from fecal samples will also be collected for potential metagenomics analyses.

For Review Only

7 STUDY DRUG

7.1 Study Drug Description

For additional information please refer to the Investigator's Brochure. Study Drug comprises PanChol or Placebo.

PanChol is a new live-attenuated OCV candidate derived from the current pandemic strain of *V. cholerae*. PanChol is highly engineered to ensure biosafety for both the vaccinated individual and the community as well as to enhance its genetic stability (Table 5).

The placebo for this study is Sterile USP grade bicarbonate buffer containing 2.5 g sodium bicarbonate, 1.6 g ascorbic acid, and 0.2 g lactose dissolved in 100 mL sterile water will be provided by the site.

Table 4. PanChol Characteristics

**Strength**	100 mL oral formulation consisting of PanChol drug substance colony forming units diluted in 100 mL sodium bicarbonate – ascorbic acid – lactose buffer.
**Volume/Formulation**	1.2 ± 0.2 mL per vial
**Route of Administration**	**Oral**

7.2 Packaging and Labeling

The Sponsor will provide the Investigator with PanChol labeled in accordance with US FDA regulatory requirements.

7.3 Study Drug Accountability

The study staff is required to document the receipt, dispensing and return/destruction of PanChol supplies to the Sponsor. The site must return all unused frozen vials of PanChol to the Sponsor or designee. Used or thawed vials should be destroyed by the Investigational Pharamcist after drug accountability has been done by the Investigational Pharmacist. The Investigational Pharmacist will be responsible for maintaining accurate records of the shipment and dispensing of the investigational product.

For Review Only

8 TREATMENT OF SUBJECTS

8.1 Study Drug Preparation

Please refer to the Pharmacy Manual provided by the Sponsor or designee for more detailed instruction for Study Drug (PanChol or placebo) preparation.

Frozen vials of PanChol will be thawed and diluted to the desired concentration with sterile USP grade bicarbonate buffer containing 2.5g sodium bicarbonate and 1.6g ascorbic acid dissolved in 100mL sterile water. The vaccine will be administered orally in a final volume of 100 mL. The starting dose is ~10^6 CFU. The appearance of PanChol will vary depending on dose. In all cases, it will be a non-viscous, homogenous liquid with no visible contaminants. At doses of 10^9 and 10^{10} the liquid will be cloudy cream-colored. At the lower doses the liquid be clear.

Placebo: Sterile USP grade bicarbonate buffer containing 2.5g sodium bicarbonate, 1.6g ascorbic acid, and 0.2g lactose dissolved in 100mL sterile water. The appearance of the placebo will be a clear liquid.

Any deviations in the proper preparation and administration of the product will be documented as protocol deviations. Study drug samples may also go to Waldor's lab to be assessed to confirm vaccine dose and/or viability.

8.1.1 Product Storage/Stability and Handling

Once received, PanChol will be stored in and dispensed by the BWH Investigational Pharmacy. PanChol will be stored upright and frozen at -80±10 °C in the storage area of the clinical investigative site pharmacy, in a secure, temperature-controlled, locked environment with restricted access.

Both PanChol and the placebo will be prepared in opaque amber bottles by the pharmacy and therefore the investigators and the participants will not be able to discern whether they received drug products vs placebo. Investigational product may be dispensed only by the investigator, by a staff member specifically authorized by the investigator, or by pharmacy staff, as appropriate.

8.2 Study Drug Administration

The Study Drug will be administered orally. A qualified clinical investigative site staff member under the supervision of the investigator or designee will administer PanChol, given as an oral solution. The volume of the study product consumed will be collected and recorded. Participants will be monitored by study staff for 30 minutes after product administration. Immediate reactogenicity events and adverse events will be collected and recorded.

8.3 Other Protocol-Required Treatment Procedures

All subjects will be administered doxycycline orally starting on ~Day 5, or earlier if they develop reactive diarrhea within the inpatient period (Grade 3 or more). Doxycycline is FDA approved for the treatment of *Vibrio cholerae* infection.https://www.accessdata.fda.gov/drugsatfda_docs/label/2013/050795s010lbl.pdf

In this study, doxycycline will be administered as 200 mg on the first day, followed by 100 mg twice a day for 4 days. Doxycycline is contraindicated in persons who have shown hypersensitivity to any of the tetracyclines. Adverse reactions observed in patients receiving tetracyclines may include anorexia, nausea, vomiting, diarrhea, rash, photosensitivity, urticaria, and hemolytic anemia. Doxycycline may increase the risk of *Clostridium difficile*-associated diarrhea.

For Review Only

8.4 Safety Monitoring Rules

Clinical safety parameters including laboratory tests and vital signs will be monitored throughout the study.

<u>Confirmation Guidance:</u>

At any time during the study (Study Drug Administration or Post Treatment Periods), clinical laboratory results that are ≥ Grade 3 in severity must be confirmed by repeating measurements, ideally in the same laboratory that performed the initial measurement on the new specimen.

8.4.1 Safety Monitoring Rules for Reactogenic Events

In this study, reactogenicity will be assessed by soliciting specific and pre-defined signs and symptoms occurring after the study drug administration. Solicited adverse events will be actively solicited during a specified post-vaccination period (D1 to D7) by the study staff. Solicited AE will include diarrhea, fever, abdominal pain, nausea, vomiting, anorexia, malaise, myalgia, tiredness, and headache

8.4.2 Unsolicited Adverse Events

Unsolicited events are any other AEs that occur following the administration of IP.

8.4.3 Medically Attended Adverse Events

Medically attended adverse events (MAAE) is defined as AE leading to hospitalization, emergency room visit or an otherwise unscheduled visit to or from medical personnel for any reason. If an AE leads to an unscheduled clinic visit with study personnel, this will be considered an MAAE.

8.4.4 New-onset chronic medical condition

New-onset chronic medical condition (NOCMC) is defined as any new ICD diagnosis (per current International Statistical Classification of Diseases and Related Health Problems) that is applied to the study participant during the course of the study, after receipt of the vaccine, that is expected to continue for at least 3 months and requires continued health care intervention.

8.4.5 Stopping Rules for Adverse Events

Enrollment will be paused, and an *ad hoc* DSMB safety review will be triggered if one or more of the following criteria are met:

- One participant experiences a treatment-related serious adverse event (SAE)
- If three or more participants experience the same treatment-related Grade 3 or higher AE

8.5 Withdrawal of Subjects from the Study Procedures

Subjects will be withdrawn from study procedures for any of the following:

- Withdrawal of consent
- The subject is unwilling or unable to comply with the protocol.

Other reasons for the withdrawal of subject from study procedures might include:

- At the discretion of the Investigator for medical reasons
- At the discretion of the Investigator or Sponsor for noncompliance
- Significant protocol deviation

For Review Only

All efforts will be made to complete and report the observations as thoroughly as possible up to the date of withdrawal. All information including the reason for withdrawal from the study, may be recorded on the case report form (CRF).

Any subject who withdraws consent to participate in the study with be removed from further treatment and study observation immediately upon the date of request. These subjects should be encouraged to complete the early termination of study procedures and observations at the time of the withdrawal (See Appendix 1).

8.6 Concomitant Therapy and Procedures

The use of concomitant therapies or procedures defined below must be recorded on the subject's CRF. The relationship of these therapies or procedures to the Study Product must also be documented on the appropriate CRF.

For Review Only

9 SERIOUS AND NON-SERIOUS ADVERSE EVENT REPORTING

9.1 Sponsor Review of Safety Information

Safety information will be collected, reviewed and evaluated by the Sponsor or designee in accordance with the conduct of the trial.

9.2 Regulatory Requirements

The Sponsor or designee is responsible for regulatory submissions and reporting SAEs including suspected unexpected serious adverse events (SUSARs) to the Investigator per the International ICH guidelines E2A and ICHG GCP as outlined by ICH E6(R2). FDA regulatory requirements will be followed.

The IRB as appropriate will be notified of any SAEs according to US regulations.

For the purposes of regulatory reporting of SUSARs there are no "expected" AEs in this study population. For Study Drug (PanChol or placebo) "expected" AEs refer to the Reference Safety Information in the Investigator's Brochure.

9.3 Definitions

9.3.1 Adverse Event

An adverse event (AE) can be any unfavourable or unintended sign (including an abnormal laboratory finding, for example) symptom or disease temporally associated with the use of the Study Drug, whether or not the AE is considered related to the Study Drug.

An adverse event can therefore be any of the following:

- Any unfavourable and unintended sign (including an abnormal laboratory finding) symptom or disease temporally associated with the use of the Study Drug, whether or not the AE is considered related to the Study Drug,
- Any new disease or exacerbation of an existing disease (a worsening in the character, frequency or severity of a known condition),
- Recurrence of an intermittent medical condition (e.g, headache) not present at baseline,
- Any deterioration in a laboratory value or other clinical test (e.g., ECG, X-ray) that is associated with symptoms or leads to a change in study treatment or concomitant treatment or discontinuation from the Study Drug.
- Adverse events that are related to protocol-mandated intervention, including those that occur prior to assignment of the study treatment (e.g., screening invasive procedures such as biopsies).

For Review Only

9.3.2 Adverse Drug Reaction and Unexpected Suspected Adverse Drug Reaction

Adverse Drug Reaction (ADR)

In the *pre-approval* clinical experience with a new medicinal product or its new usages particularly as the therapeutic doses may not have been established, ADR is defined as follows:

> All noxious and unintended responses to a medicinal product related to any dose should be considered adverse drug reactions.

The phrase "responses to a medicinal product" means that a causal relationship between the medicinal product and this adverse event has been determined as at least a reasonable possibility, i.e., the relationship cannot be ruled out.

Suspected Unexpected Adverse Drug Reaction

A suspected unexpected ADR is any ADR, the nature or severity of which is not consistent with the Reference Safety Information in the applicable Investigator's Brochure. A suspected adverse reaction implies a lesser degree of certainty about the causality than an adverse reaction.

9.3.3 Serious Adverse Event

A serious adverse event is any adverse event that in the view of either the Investigator or Sponsor meets any of the following criteria:

- Results in death.
- Is Life-threatening: that is, poses an immediate risk of death at the time of the event An AE is considered "life-threatening" if in the view of with the Investigator or Sponsor, its occurrence places the subject at immediate risk of death. It does not include an AE or suspected adverse reaction that had occurred in a more severe form might have caused death.
- Requires inpatient hospitalization or prolongation of existing hospitalization: Hospitalization is defined as an admission of greater than 24 hours to a medical facility and does not always qualify as an AE.
- Results in a persistent or significant incapacity or substantial disruption of the ability to conduct normal life functions.
- Results in a congenital anomaly or birth defect in the offspring of the subject (whether the subject is male or female).
- Important medical events that may not result in death, be life-threatening, or require hospitalization, may be considered an SAE when, based upon appropriate medical judgment, they may jeopardize the subject and may require medical or surgical intervention to prevent one of the outcomes listed in this definition. Examples of such events are: Intensive treatment in an emergency room or at home for allergic bronchospasm; Blood dyscrasias or convulsions that do not result in inpatient hospitalization or development of drug dependency or drug abuse.

9.3.4 Adverse Event of Special Interest

An adverse event of special interest (AESI) including both serious or non-serious events is one of scientific and medical concern specific to the Sponsor's product or program, for which ongoing monitoring and rapid communication to the Sponsor could be appropriate.

For Review Only

Adverse events of special interest if defined in the study protocol or associated documents are required to be reported by the Investigator to the Sponsor immediately, no more than 24 hours after the Investigator's first knowledge of the event.

At the time of study initiation, no AESI are specified for this study.

9.3.5 Targeted Medical Event

Other targeted medical events (TMEs) or other adverse events of interest (OAEI), are AEs including both serious or non-serious events, that may be defined in the study protocol or associated documents for further characterization or evaluation of product specific adverse events.

At the time of study initiation, no TMEs are specified for this study.

9.4 Monitoring and Recording Adverse Events

Any pre-existing conditions or signs and/or symptoms present in a subject prior to the start of the study (i.e., before administration of the Study Product) should be recorded in the Medical History and not recorded as AEs unless the pre-existing condition worsened. The investigator should group signs and symptoms into a single term that constitutes a **single unifying diagnosis** if possible. Before a diagnosis is confirmed all symptoms should be recorded as separate AEs.

9.4.1 Serious Adverse Events

In the interest of subject safety and in order to fulfill regulatory requirements, all SAEs (regardless of their relationship to the Study Drug) should be reported to the Sponsor or designee within 24 hours of the Site's first knowledge of the event. The collection of SAEs will begin after the subject receives the Study Product and stop at the end of the subject's follow-up period which is defined as the subject's last protocol-specified study visit. SAEs considered to be related to the Study Drug and any deaths will continue to be reported for 30 days after the subject's last visit. SAEs will be reported with an electronic data capture (EDC) system whenever possible. If EDC is not available the SAEs can be initially reported using a paper Initial Serious Adverse Event Form which should be E-mailed to the Sponsor or designee.

The SAE reporting instructions should include the E-mail address.

Detailed information should be actively sought and included on the Follow-Up Serious Adverse Event Forms as soon as additional information is available. All SAEs will be followed until resolution. SAEs that remain ongoing at the subject's last protocol-specified follow-up visit will be evaluated by the Investigator. If the Investigator considers that the subject's condition is unlikely to resolve, the Investigator will determine the follow-up requirement in discussion with the PSRT.

9.4.2 Non-Serious Adverse Events

Following initiation of investigational product, all AEs will be collected, regardless of cause or relationship, until D29. All AEs should be followed until resolution or stabilization, if possible. Whether or not considered treatment-related, all participants experiencing AEs must be monitored periodically per Investigator discretion until symptoms subside, any abnormal laboratory values have resolved or returned to baseline levels, or they are considered irreversible, or until there is a satisfactory explanation for the changes observed. Certain AEs may be followed beyond the protocol-defined follow-up period. Whether or not considered treatment-related, all participants experiencing AEs must be monitored periodically (per investigator discretion) until symptoms subside, any abnormal laboratory values have resolved or returned to baseline levels or they are considered irreversible, or until there is a satisfactory explanation for the changes observed. From D30 to D180, only medically attended adverse events (MAAE), new-onset chronic medical conditions (NOCMC), and SAE will be recorded and reported.

For Review Only

AEs that occur prior to administration of the Study Drug will be recorded as medical history.

9.4.3 Evaluation of Adverse Events (Serious and Non-Serious)

The Investigator's opinion of the following should be documented on the Adverse Event CRF.

9.4.3.1 Relationship to the Study Drug

The event's relationship to the Study Drug (PanChol or placebo) is characterized by one of the following:

Causality (Yes or No) should be determined by the investigator or qualified sub-investigator. An answer of Yes should be entered when, in their opinion, there is either (a) a *reasonable* possibility that the AE is associated with IP or (b) no reasonable alternative explanation can be identified. Otherwise, causality to IP should be categorized as No. A mere possibility of a causal relationship is not grounds for a Yes categorization.

9.4.3.2 Severity

The severity of AEs and SAEs will be graded based on the FDA Toxicity Grading Scale for Healthy Adult and Adolescent Volunteers enrolled in Preventive Vaccine Clinical Trials Sept 2007. Any AE not listed in this scale will be graded using Table 6.

Table 5. Severity of AE proposed scale

**Systemic Illnes**	**Mild (Grade 1)**	**Moderate (Grade 2)**	**Severe (Grade 3)**	**Potentially Life Threatening (Grade 4)**
Illness or clinical adverse event (as defined according to applicable regulations)	No interference with activity	Some interference with activity not requiring medical intervention	Prevents daily activity and requires medical intervention	ER visit or hospitalization

9.4.3.3 Treatment Given for Adverse Event

Any treatment (e.g., medication or procedures) given for the AE should be recorded on the Adverse Event CRF. Treatment should also be recorded on the concomitant medication CRF as appropriate.

9.4.3.4 Outcome of the Adverse Event

The AE's outcome is characterized by one of the following:

- **Fatal:** Subject died (date of death should be entered as the SAE resolution date)
- **Not Recovered/Not Resolved:** Subject terminates from the trial and the AE continues
- **Recovered/Resolved:** Subject recovered completely from the AE
- **Recovered/Resolved with Sequelae:** The signs/symptoms of the reported AE have improved but not completely resolved, and a new baseline for the subject is established since full recovery <u>is not</u> expected.
- **Recovering/Resolving:** The signs/symptoms of the reported AE have improved but not completely resolved but a full recovery <u>is expected</u>.
- **Unknown:** The outcome of the reported AE is not available, e.g., subject is lost to follow-up.

For Review Only

9.4.3.5 Follow-up of Adverse Event

Investigator Follow-up

During the study period, the Investigator should follow each AE until the event has resolved to baseline grade or better, the event is assessed as stable, the subject is lost to follow-up, or the subject withdraws consent. Every effort should be made to follow all SAEs considered to be related to the Study Drug or to study procedures until a final outcome can be reported.

Resolution of AE (with dates) should be documented on the Adverse Event CRF and in the subject's medical record to facilitate source data verification.

The Investigator should follow-up with all pregnancies reported during the study minimally until the end of the study. Pregnancies reported within a month of study drug administration will be followed until an outcome is available (Section 9.5.4).

9.5 Procedures for Handling Special Situations

9.5.1 Abnormalities of Laboratory Tests

Clinically significant abnormal laboratory tests may, in the opinion of the Investigator, constitute or be associated with an AE. Examples of these abnormal laboratory results that are associated with symptoms or require treatment, e.g., bleeding due to thrombocytopenia, tetany due to hypocalcemia, or cardiac arrhythmias due to hyperkalemia. Whenever possible, the underlying diagnosis should be listed in preference to abnormal laboratory values as AEs. Clinically significant abnormalities will be monitored by the Sponsor and PSRT that further follow-up is not required. Laboratory abnormalities deemed not clinically significant (NCS) by the Investigator should not be reported as AEs. Similarly, laboratory abnormalities reported as AEs by the Investigator should not be deemed NCS on the laboratory sheet.

The Investigator or appropriate designee is responsible for reviewing and signing the laboratory reports. The signed clinical laboratory reports will serve as source documents and should include the Investigator's assessment of clinical significance of out of range/abnormal laboratory values.

9.5.2 Prescheduled or Elective Procedures or Routinely Scheduled Treatments

A prescheduled or elective procedure or routinely scheduled treatment will not be considered an SAE, even if the subject is hospitalized; the Site must document all of the following:

- The prescheduled or elective procedure or routinely scheduled treatment was scheduled (or was on a waiting list to be scheduled) prior to obtaining the subject's consent to participate in the study.
- The condition that required the prescheduled or elective procedure or routinely scheduled treatment was present before and did not worsen or progress in the opinion of the Investigator between the subject's consent to participate in the study and the timing of the procedure or treatment.
- The prescheduled or elective procedure or routinely scheduled treatment is the sole reason for the intervention or hospital admission.

9.5.3 Dosing Errors

Study Drug (PanChol or placebo) errors (including overdose, underdose and administration error) should be documented as Protocol Deviations. A brief description should be provided in the deviation report including whether the subject was symptomatic (list symptoms) or asymptomatic and the event was accidental or intentional.

For Review Only

Dosing details should be captured on the Dosing CFR. If the subject receives a dose of Study Drug (PanChol or placebo) that exceeds protocol specifications and the subject is symptomatic, then the symptom(s) should be documented as an AE and be reported per Section 9.4.

An overdose is the accidental or intentional use of a drug in an amount higher than the dose being studied. An overdose or incorrect administration of study treatment is not itself an adverse event, but it may result in an adverse event. All adverse events associated with and overdose or incorrect administration of the Study Drug should be recorded on the Adverse Event CRF. If the associated adverse event fulfills seriousness criteria, the event should be reported to the Sponsor immediately (i.e., no more than 24 hours after learning of the event).

Should an overdose occur, the Investigator or designee should refer to the Guidance to Investigator's section of the Investigator's Brochure and contact the Sponsor or designee within 24 hours.

9.5.4 Contraception and Pregnancy

If a subject becomes pregnant or pregnancy is suspected, then the Site staff must be informed immediately. The pregnancy should be reported in the EDC **within 24 hours** of first learning of the occurrence of the pregnancy. If the EDC is not an option, the pregnancy can initially be reported using a paper Pregnancy Notification Form, which should be E-mailed to the Sponsor or designee. Follow-up information including delivery or termination should be reported within 24 hours if the subject is still enrolled in the clinical trial or via the Pregnancy Notification Form if the subject is no longer enrolled in the clinical trial.

Payment for all aspects of obstetrical care, child or related care will be the subject's responsibility.

Female Subjects: If a suspected pregnancy occurs while on the study and within 180 days of the administration of the Study Drug, a pregnancy test will be performed. If the pregnancy test is positive, the subject will be encouraged to complete the post-treatment follow-up portion of the study to the extent that study procedures do not interfere with the pregnancy. Regardless of the continued study participation, the Investigator with assist the subject in getting obstetrical care and the progress of the pregnancy will be followed until the end of the study. If pregnancy is reported within one month of study product administration, the participant will be followed until the outcome of the pregnancy is known (i.e., delivery, elective termination, or spontaneous abortion). If the pregnancy results in the birth of a child, the Site and Sponsor may request access to the mother and infant's medical records to obtain additional information relevant to the pregnancy progress and outcome. A longer follow-up may be requested if the newborn child experiences a medical condition. Follow-up will be performed to the extent permitted by the applicable regulations and privacy considerations, e.g., pregnancy ICF may be required.

For Review Only

10 STATISTICAL CONSIDERATIONS

Full details of statistical analyses with be described in the Statistical Analysis Plan (SAP) for PanChol. The SAP will be finalized prior to database lock. Any changes to the methods described in the final SAP will be described and justified as needed in the clinical study report (CSR). An overview is presented below.

The prior protocol revision for module 3 was based on results from the first five doses tested in Module 1, in which it has been observed that the dose-toxicity response is flat over 5 orders of magnitude. No serious adverse events were observed at any dose while there were definitive immune responses. A result of these findings is that Module 2 (refined dose finding) is not necessary. In Module 1, 15 subjects had at least a dose of log 6 with no SAEs. Similarly 12 subjects had at least a dose of log 7 with no SAEs, and so on. Corresponding one sided upper 95% confidence bounds are approximately .2, .25, .33, and .5 for log doses 6, 7, 8, and 9. These bounds are conservative because each does not fully utilize information from higher doses. In any case, the need for Module 2 is obviated because there is no adverse response to titrate using refined dose finding. Hence the protocol is revised to proceed directly to a revised Module 3. Because of the flat and zero dose response from Module 1, two doses have been selected for Module 3 based on a combination of clinical/biological considerations and potential manufacturing and field logistics. These chosen log doses are 7 and 8, emphasizing that these are within the range already tested.

Fourteen subjects will be randomly assigned to each masked dose along with 8 placebo controls. If no serious adverse events are seen at either dose, the one sided upper 95% confidence bound on the AE rate for the lower dose will be 10%, and for the upper dose will be 21% (ignoring results from Module 1). These will be appropriate to inform the next stage of development.

10.1 Sample Size Considerations

The sample size intended for this study is consistent with the recommendations in the Code of Federal Regulations CFR 312.21 for first-in-human investigation and will provide adequate evidence for PanChol's safety and immunogenicity so that rapid subsequent development and field trials can be planned. In total, about 57 healthy adult volunteers will be enrolled in 2 modules. The first module (dose-ranging) will require about 21 subjects, and the expansion module will require 36 including 8 subjects receiving placebo. With 28 subjects in the expansion cohort module, the total number at the optimal dose will be at least 26-30. If no AE were observed in such a cohort, the upper one-sided 95% exact binomial confidence bound on the event rate would be approximately 10%. This would be an appropriate clinical threshold on which to base field trials.

10.2 Populations

Safety Population: All subjects who receive Study Drug or Placebo.

10.3 Definition of Baseline

Unless specified otherwise, baseline will be the last value prior to administration of the Study Drug.

10.4 Treatment Assignment Procedures

10.4.1 Randomization/Blinding

Module 1 is a non-randomized module.

For Review Only

An automated system will be employed to manage participant randomization and treatment assignments during Module 3. This module will include 28 subjects randomized to PanChol and 8 participants randomized to placebo. Participants, investigators, and endpoint assessors will be blinded. The unblinded member of the IP formulation team will be provided with the treatment assignment codes for the preparation of the vaccine or placebo to be given to each subject.

10.5 Missing data Handling

Values for missing safety laboratory data will not be imputed. However, a missing baseline result will be replaced with a screening result, if available. If no pre-treatment laboratory value is available, the baseline value will be assumed to be normal (i.e., no grade [Grade 0]) for the summary of graded laboratory abnormalities. If safety laboratory results for a subject are missing for any reason at a time point, the subject will be excluded from the calculation of summary statistics for that time point. Values for missing vital signs data will not be imputed. However, a missing baseline result will be replaced with a screening result, if available.

10.6 Demographics and Baseline Characteristics

Demographic and baseline characteristics will be presented using standard descriptive statistics such as sample size, mean, standard deviation, median, minimum, and maximum for continuous variables and numbers and of participants for categorical variables.

10.7 Safety analysis

The primary analysis set for safety analyses will include all participants who received any amount of IP. The incidence of any AE (solicited and unsolicited) will be determined. Proportions of participants with AE, overall, by grade and relationship with vaccination will be calculated. All AEs will be collected and reported until D29. From D30 to D180, medically attended adverse events (MAAEs), new-onset chronic medical conditions (NOCMC), and SAEs will be collected and reported. For Module 3 analysis, proportions of subjects with AE will be compared between those who received PanChol and those who received placebo using Fisher's exact test with a two-sided 5% type I error rate.

10.8 Immunogenicity analysis

For both modules, immunogenicity data will be presented using descriptive statistics, including incidence, magnitude, and phenotype of cell responses and antibody titers, as applicable. For Module 3, seroconversion of vibriocidal titers pre- and post-vaccination will be compared between subjects who received PanChol and those who received placebo using Fisher's exact test with a two-sided 5% type I error rate.

For Review Only

11 INVESTIGATOR'S REGULATORY OBLIGATIONS

11.1 Informed Consent

Before a subject's participation in the trial, the Investigator is responsible for obtaining written informed consent from the subject after adequate explanation of the aims, methods, anticipated benefits and potential hazards of the study before any protocol-specific screening procedures or any Study Drug (PanChol or placebo) are administered.

11.2 Ethical Conduct of the Study

The study must be conducted in compliance with this clinical study protocol, GCP as outlined by ICH E6 (R2) as well as the demands of national drug and data protection laws and all applicable local and national regulatory requirements.

11.3 Institutional Review Board

A copy of the protocol proposed ICFs, other written subject information and any proposed advertising material must be submitted to the IRB for written approval.

A copy of the written approval from the IRB must be obtained by the Sponsor or designee before starting the study (recruitment of subjects and shipment of Study Drug) and should be documented in a letter to the Investigator specifying the protocol number, protocol version, protocol date, documents reviewed, and date on which the committee met and granted the approval.

Any modifications made to the protocol after receipt of IRB approval must also be submitted to the IRB for approval before implementation. The Investigator should notify the IRB of deviations from the protocol in accordance with ICH GCP, as outlined by ICH E6(R2). The Investigator should also notify the IRB of SAEs occurring at the Site and other AE reports received from the Sponsor or designee, in accordance with local procedures.

The Investigator will be responsible for obtaining annual IRB approval/renewal throughout the duration of the study. Copies of the Investigator's reports, all IRB submissions and continuance of approval must be sent to the Sponsor or designee.

11.4 Consent for Future Use of Stored Specimens and Data

Residual samples/specimens are those that are left over after protocol-specified testing and this study has been completed. Subjects will be asked for permission to keep any remaining (residual) specimens derived from venous blood and stool samples for possible use in future research studies, such as examining additional immunological assessments or testing for antibodies against other viruses or bacteria. These residual specimens will be stored coded indefinitely at BWH. The information provided to any collaborating investigators outside of the BWH will not contain directly identifiable information.

11.5 Subject Confidentiality

The investigator must assure that the subject's anonymity will be strictly maintained and that their identities are protected from unauthorized parties. On the CRFs or other documents submitted to the Sponsor or designee, subjects should be identified by subject identification number only. Documents that are not for submission to the Sponsor or designee (e.g., ICFs) should be kept in strict confidence by the Investigator.

In compliance with Federal and local regulations ICH GCP as outlined in ICH E6(R2) Guidelines, it is required that the Investigator and institution permit authorized representatives of the Sponsor, regulatory

For Review Only

agency(s) and the IRB direct access to review the subject's original medical records for verification of study-related procedures and data. Direct access includes examining, analyzing, verifying, and reproducing any records and reports that are important to the evaluation of the study. The Investigator is obliged to inform and obtain the consent of the subject to permit named representatives to have access to his/her study-related records without violating the confidentiality of the subject.

The investigator must maintain up-to-date traceable information or logs of codes, names, and addresses for all participants screened and for all participants enrolled in the trial.

In compliance with local and/or regional regulations, this clinical study may be registered, and study results may be posted on public registries, such as ClinicalTrials.gov.

11.6 Privacy and Confidentiality

- ☒ Study procedures will be conducted in a private setting
- ☒ Only data and/or specimens necessary for the conduct of the study will be collected
- ☒ Data collected (paper and/or electronic) will be maintained in a secure location with appropriate protections such as password protection, encryption, physical security measures (locked files/areas)
- ☒ Specimens collected will be maintained in a secure location with appropriate protections (e.g. locked storage spaces, laboratory areas)
- ☒ Data and specimens will only be shared with individuals who are members of the IRB-approved research team or approved for sharing as described in this IRB protocol
- ☒ Data and/or specimens requiring transportation from one location or electronic space to another will be transported only in a secure manner (e.g. encrypted files, password protection, using chain-of-custody procedures, etc.)
- ☒ All electronic communication with participants will comply with Mass General Brigham (MGB) secure communication policies
- ☒ Identifiers will be coded or removed as soon as feasible and access to files linking identifiers with coded data or specimens will be limited to the minimal necessary members of the research team required to conduct the research
- ☒ All staff are trained on and will follow the MGB policies and procedures for maintaining appropriate confidentiality of research data and specimens
- ☒ The PI will ensure that all staff implement and follow any Research Information Service Office (RISO) requirements for this research
- ☐ Additional privacy and/or confidentiality protections

For Review Only

12 ADMINISTRATIVE AND LEGAL OBLIGATIONS

12.1 Protocol Amendments

Protocol amendments must be made only with the prior approval of the Sponsor or designee.

Agreement from the Investigator must be obtained for amendments to both the protocol and ICF. The regulatory authority and IRB must be informed of all amendments and give approval for any amendments likely to affect the safety of the subjects or the conduct of the trial. The Investigator must send a copy of the approval letter from the IRB to the Sponsor or designee.

12.2 Study Termination

The Sponsor or designee reserves the right to terminate the study. The Investigator reserves the right to terminate their participation in the study according to the terms of the site contract. The Investigator should notify the IRB in writing of the trial's completion or early termination and send a copy of the notification to the Sponsor or designee

12.3 Study Documentation and Storage

An electronic CRF utilizing EDC application will be used for this study.

Source documents are original documents, data, and records from which the subject's CRF data are obtained. These include but are not limited to hospital records, clinical and office charts, laboratory and pharmacy records, diaries, imaging, and correspondence. Source documents may be eCRFs and paper CRFs.

The Investigator and Site staff are responsible for maintaining a comprehensive and centralized filing system of all study-related essential documentation in accordance with ICH GCP as outlined in ICH E6(R2) suitable for inspection at any time by representatives from the Sponsor or designee and/or applicable regulatory authorities. Elements should include:

- Subject files containing completed CRFs, informed consents and supporting copies of source documentation.
- Study files containing the protocol with all amendments, Investigator's Brochure, copies of pre-study documentation and all correspondence to and from the IRB and the Sponsor or designee.
- If drug supplies are maintained at the Site, proof of receipt, Study Drug Product Accountability Record, Return of Study Drug Product for Destruction, final Study Drug Product reconciliation and all drug-related correspondence.

In addition, all original source documents supporting entries in the CRFs must be maintained and be readily available. No study document should be destroyed without prior written agreement between the Sponsor or designee and the Investigator. Should the Investigator wish to assign the study records to another party or move them to another location, he/she must notify the Sponsor or designee.

The investigator is responsible to ensure the accuracy, completeness, legibility, and timeliness of the data reported. The source documents should be completed in a neat, legible manner to ensure accurate interpretation of data. Black or blue permanent ink is required to ensure clarity of reproduced copies. When making changes or corrections, cross out the original entry with a single line, and initial and date the change.

Clinical data will be stored in a secure, web-based EDC and eCRFs will be developed and maintained by the data coordinating center (DCC). Data reported in the eCRF derived from source data collection forms should be consistent or the discrepancies should be explained.

For Review Only

All documentation related to the study should be retained for the period of time required by applicable local law. If it becomes necessary for the applicable IRB, or applicable regulatory authorities to review or audit any documentation relating to the study, the investigator must permit direct access to all source documents/data.

12.4 Data Coordinating Center/Biostatistician Responsibilities

Data collection is the responsibility of the study personnel at the participating clinical study site under the supervision of the site principal investigator (PI). During the study, the site PI must maintain complete and accurate documentation for the study.

The data coordinating center (DCC) for this study will be responsible for database development and maintenance, data management, data quality review, data analysis, and reporting of the study data.

12.5 Data Capture Methods

Clinical data (including, but not limited to, AE/SAEs, concomitant medications, medical history, physical assessments) will be collected on data collection forms by study personnel then entered into eCRFs. Clinical laboratory values will be collected from the electronic medical record (EPIC). The EDC includes password protection and internal quality checks, such as automatic range checks, to identify data that appear inconsistent, incomplete, or inaccurate. eCRFs must be completed only by the Investigator or persons designated by the Investigator. eCRF data must be entered by trained site personnel with access to the secure eCRF system. All data entered into the eCRF must also be available in the source documents. Correction on source documents must be made to not obliterate the original data and must be initialed and dated by the person who corrected.

12.6 Study Monitoring

The DCC will conduct internal data review for completeness and timeliness of data entry into the EDC system and will also conduct internal data monitoring. Study monitors will perform source data verification to confirm that data entered into the eCRF in the EDC by authorized site personnel are accurate, complete, and verifiable from source documents; that the safety and rights of participants are being protected; and that the study is being conducted in accordance with the currently approved protocol, GCP guidelines of ICH, and all applicable regulatory requirements. Incomplete data or discrepancies between source data and the eCRFs will be tracked through queries generated and documented within the EDC and this will include documentation of query resolution by the study team and closing of queries by the DCC. Details related to DCC data review and DCC monitoring describing checks for completeness, automated logic checks programmed within the EDC, as well as data monitoring strategy (eg, risk-based monitoring), anticipated frequency, and monitoring techniques (central, remote, or on-site monitoring) will be provided in the study Data Management Plan and Data Monitoring Plan.

12.7 Site Audits

In accordance with ICH GCP as outlined in ICH E6(R2) and with the Sponsor's audit plans, this study may be audited. Inspection of Site facilities (e.g., pharmacy, drug storage areas, laboratories) and review of study-related records to evaluate the trial conduct and compliance with the protocol and applicable regulatory requirements.

A regulatory authority, IRB may visit the clinical investigative site to perform audits or inspections, including source data verification. The Investigator must allow monitoring, audits, IRB review, and regulatory agency inspections and provide direct access to source documents and eCRFs.

For Review Only

12.8 Language

CRFs must be completed in English. Generic names and trade names are acceptable for concomitant medications. Combination medications should be recorded using their trade name. All written information and other material to be used by subjects and investigative staff must use vocabulary and language that are clearly understood.

12.9 Remuneration/Payments

Subject will receive $200 for every day during the inpatient period and $50 for each of the outpatient visits visit. Parking vouchers may be provided. If subject does not complete the study, for any reason, they will be paid for each study visit they do complete.

The study vaccinations, study visits, examinations, laboratory tests, and all the other procedures that are done only for the research will be paid for by the study funds.

12.10 Participant Texting Preference

Text messages by mobile/cell phones are a common form of communication. This research study involves sending participant text messages that are relevant to the study. Texting over mobile/cell phones carries security risks because text messages to mobile/cell phones are not encrypted. This means that information participants send or receive by text message could be intercepted or viewed by an unintended recipient, or by participants mobile/cell phone provider or carrier.

Below are some important points about texting in this research study:

Text messages are not encrypted, and therefore carry security risks. This research study and MGB Healthcare are not responsible for any interception of messages sent through unencrypted text message communications.

Participant will be responsible for all fees charged by participants carrier's service plan for text messaging. This research study and Partners Healthcare are not responsible for any increased charges, data usage against plan limits or changes to data fees from the research texts.

Text messages will usually be read during regular business hours. Texts sent on nights or weekends may not be read until the next business day.

Text messaging should not be used in case of an emergency. If participants experience a medical emergency, call 911 or go to the nearest hospital emergency department.

Participants may decide to not send or receive text messages with staff associated with this research study at any time. Participants can do this in person or by sending the research number a text message that says, "Stop Research Text."

Participant's agreement applies to this research study only. Agreeing to other texts from MGB Healthcare, for example appointment reminders, is a separate process. Opting out of other texts from MGB Healthcare is a separate process as well. In addition, it is participants responsibility to update their mobile/cell phone number with this research study in the event of a change.

For Review Only

13 LITERATURE REFERENCES

Abel S, Abel zur Wiesch P, Chang HH, Davis BM, Lipsitch M, Waldor MK. Sequence tag-based analysis of microbial population dynamics. Nat Methods. 2015 Mar;12(3):223-6, 3 p following 226.

Ali M, Emch M, Park JK, Yunus M, Clemens J. Natural cholera infection-derived immunity in an endemic setting. J Infect Dis. 2011;204(6):912-8.

Ali M, Nelson AR, Lopez AL, Sack DA. Updated global burden of cholera in endemic countries. PLoS Negl Trop Dis. 2015;9(6):e0003832.

Ananthakrishnan R, Green S, Chang M, Doros G, Massaro J, LaValley M. Systematic comparison of the statistical operating characteristics of various Phase I oncology designs. Contemp Clin Trials Commun. 2017;5:34-48.

Bhattacharya SK, Sur D, Ali M, Kanungo S, You YA, Manna B, et al. 5 year efficacy of a bivalent killed whole-cell oral cholera vaccine in Kolkata, India: a cluster-randomised, double-blind, placebo-controlled trial. Lancet Infect Dis. 2013;13(12):1050-6.

Butterton JR, Beattie DT, Gardel CL, Carroll PA, Hyman T, Killeen KP, Mekalanos JJ, Calderwood SB. Heterologous antigen expression in Vibrio cholerae vector strains. Infect Immun. 1995 Jul;63(7):2689-96.

Calain P, Chaine JP, Johnson E, Hawley ML, O'Leary MJ, Oshitani H, et al. Can oral cholera vaccination play a role in controlling a cholera outbreak? Vaccine. 2004;22(19):2444-51.

Chen WH, Garza J, Choquette M, Hawkins J, Hoeper A, Bernstein DI, et al. Safety and immunogenicity of escalating dosages of a single oral administration of peru-15 pCTB, a candidate live, attenuated vaccine against enterotoxigenic Escherichia coli and Vibrio cholerae. Clin Vaccine Immunol. 2015;22(1):129-35.

Chin CS, Sorenson J, Harris JB, Robins WP, Charles RC, Jean-Charles RR, et al. The origin of the Haitian cholera outbreak strain. N Engl J Med. 2011;364(1):33-42.

Chowdhury F, Ali Syed K, Akter A, Rahman Bhuiyan T, Tauheed I, Khaton F, et al. A phase I/II study to evaluate safety, tolerability and immunogenicity of Hillchol(R), an inactivated single Hikojima strain based oral cholera vaccine, in a sequentially age descending population in Bangladesh. Vaccine. 2021;39(32):4450-7.

Clemens JD, Nair GB, Ahmed T, Qadri F, Holmgren J. Cholera. Lancet. 2017;390(10101):1539-49.

Clemens JD, Sack DA, Harris JR, Chakraborty J, Khan MR, Stanton BF, et al. Field trial of oral cholera vaccines in Bangladesh. Lancet. 1986;2(8499):124-7.

Clemens JD, Sack DA, Harris JR, Van Loon F, Chakraborty J, Ahmed F, et al. Field trial of oral cholera vaccines in Bangladesh: results from three-year follow-up. Lancet. 1990;335(8684):270-3.

Cohen MB, Giannella RA, Bean J, Taylor DN, Parker S, Hoeper A, et al. Randomized, controlled human challenge study of the safety, immunogenicity, and protective efficacy of a single dose of Peru-15, a live attenuated oral cholera vaccine. Infect Immun. 2002;70(4):1965-70.

Crean TI, John M, Calderwood SB, Ryan ET. Optimizing the germfree mouse model for in vivo evaluation of oral Vibrio cholerae vaccine and vector strains. Infect Immun. 2000 Feb;68(2):977-81.

Desai SN, Pezzoli L, Martin S, Costa A, Rodriguez C, Legros D, et al. A second affordable oral cholera vaccine: implications for the global vaccine stockpile. Lancet Glob Health. 2016;4(4):e223-4.

For Review Only

Devault AM, Golding GB, Waglechner N, Enk JM, Kuch M, Tien JH, et al. Second-pandemic strain of Vibrio cholerae from the Philadelphia cholera outbreak of 1849. N Engl J Med. 2014;370(4):334-40.

DHHS., FDA., Center for Biologics Evaluation and Research. Toxicity Grading Scale for Healthy Adult and Adolescent Volunteers Enrolled in Preventive Vaccine Clinical Trials 2007 [Available from: https://www.fda.gov/media/73679/download].

Domman D, Quilici ML, Dorman MJ, Njamkepo E, Mutreja A, Mather AE, et al. Integrated view of Vibrio cholerae in the Americas. Science. 2017;358(6364):789-93.

Fullner KJ, Boucher JC, Hanes MA, Haines GK 3rd, Meehan BM, Walchle C, Sansonetti PJ, Mekalanos JJ. The contribution of accessory toxins of Vibrio cholerae O1 El Tor to the proinflammatory response in a murine pulmonary cholera model. J Exp Med. 2002 Jun 3;195(11):1455-62.

Garcia L, Jidy MD, Garcia H, Rodriguez BL, Fernandez R, Ano G, et al. The vaccine candidate Vibrio cholerae 638 is protective against cholera in healthy volunteers. Infect Immun. 2005;73(5):3018-24.

Garrett-Mayer E. The continual reassessment method for dose-finding studies: a tutorial. Clin Trials. 2006;3(1):57-71.

Ghosh P, Naha A, Pazhani GP, Ramamurthy T, Mukhopadhyay AK. Genetic traits of Vibrio cholerae O1 Haitian isolates that are absent in contemporary strains from Kolkata, India. PLoS One. 2014;9(11):e112973.

Harris JB, LaRocque RC, Chowdhury F, Khan AI, Logvinenko T, Faruque AS, et al. Susceptibility to Vibrio cholerae infection in a cohort of household contacts of patients with cholera in Bangladesh. PLoS Negl Trop Dis. 2008;2(4):e221.

Harris JB, LaRocque RC, Qadri F, Ryan ET, Calderwood SB. Cholera. Lancet. 2012;379(9835):2466-76.

Harris JB. Editorial Commentary: Resurrecting a Live Oral Cholera Vaccine. Clin Infect Dis. 2016;62(11):1336-7.

Herrington DA, Hall RH, Losonsky G, Mekalanos JJ, Taylor RK, Levine MM. Toxin, toxin-coregulated pili, and the toxR regulon are essential for Vibrio cholerae pathogenesis in humans. J Exp Med. 1988;168(4):1487-92.

Hsiao A, Ahmed AM, Subramanian S, Griffin NW, Drewry LL, Petri WA, Jr., et al. Members of the human gut microbiota involved in recovery from Vibrio cholerae infection. Nature. 2014;515(7527):423-6.

Huang Y, Karuna ST, Janes H, Frahm N, Nason M, Edlefsen PT, et al. Use of placebos in Phase 1 preventive HIV vaccine clinical trials. Vaccine. 2015;33(6):749-52.

Hubbard TP, Billings G, Dorr T, Sit B, Warr AR, Kuehl CJ, et al. A live vaccine rapidly protects against cholera in an infant rabbit model. Sci Transl Med. 2018;10(445).

Ivers LC. Eliminating Cholera Transmission in Haiti. N Engl J Med. 2017;376(2):101-3.

Iwanaga M, Yamamoto K, Higa N, Ichinose Y, Nakasone N, Tanabe M. Culture conditions for stimulating cholera toxin production by Vibrio cholerae O1 El Tor. Microbiol Immunol. 1986;30(11):1075-83.

Kabir S. Critical analysis of compositions and protective efficacies of oral killed cholera vaccines. Clin Vaccine Immunol. 2014;21(9):1195-205.

For Review Only

Kanungo S, Sen B, Ramamurthy T, Sur D, Manna B, Pazhani GP, et al. Safety and immunogenicity of a live oral recombinant cholera vaccine VA1.4: a randomized, placebo controlled trial in healthy adults in a cholera endemic area in Kolkata, India. PLoS One. 2014;9(7):e99381.

Kaper JB, Morris JG, Jr., Levine MM. Cholera. Clin Microbiol Rev. 1995;8(1):48-86.

Kauffman RC, Bhuiyan TR, Nakajima R, Mayo-Smith LM, Rashu R, Hoq MR, et al. Single-Cell Analysis of the Plasmablast Response to Vibrio cholerae Demonstrates Expansion of Cross-Reactive Memory B Cells. MBio. 2016;7(6).

Kenner JR, Coster TS, Taylor DN, Trofa AF, Barrera-Oro M, Hyman T, et al. Peru-15, an improved live attenuated oral vaccine candidate for Vibrio cholerae O1. J Infect Dis. 1995;172(4):1126-9.

Kumar P, Mishra DK, Deshmukh DG, Jain M, Zade AM, Ingole KV, et al. Vibrio cholerae O1 Ogawa El Tor strains with the ctxB7 allele driving cholera outbreaks in south-western India in 2012. Infect Genet Evol. 2014;25:93-6.

Levine MM, Kaper JB, Herrington D, Ketley J, Losonsky G, Tacket CO, et al. Safety, immunogenicity, and efficacy of recombinant live oral cholera vaccines, CVD 103 and CVD 103-HgR. Lancet. 1988;2(8609):467-70.

Luquero FJ, Azman AA. Protection of young children with cholera vaccine. Lancet Infect Dis. 2018;18(9):947-8.

Luquero FJ, Grout L, Ciglenecki I, Sakoba K, Traore B, Heile M, et al. Use of Vibrio cholerae vaccine in an outbreak in Guinea. N Engl J Med. 2014;370(22):2111-20.

Luquero FJ, Grout L, Ciglenecki I, Sakoba K, Traore B, Heile M, et al. First outbreak response using an oral cholera vaccine in Africa: vaccine coverage, acceptability and surveillance of adverse events, Guinea, 2012. PLoS Negl Trop Dis. 2013;7(10):e2465.

Luquero FJ, Rondy M, Boncy J, Munger A, Mekaoui H, Rymshaw E, et al. Mortality Rates during Cholera Epidemic, Haiti, 2010-2011. Emerg Infect Dis. 2016;22(3):410-6.

McCarty JM, Lock MD, Hunt KM, Simon JK, Gurwith M. Safety and immunogenicity of single-dose live oral cholera vaccine strain CVD 103-HgR in healthy adults age 18-45. Vaccine. 2018;36(6):833-40.

Millet YA, Alvarez D, Ringgaard S, von Andrian UH, Davis BM, Waldor MK. Insights into Vibrio cholerae intestinal colonization from monitoring fluorescently labeled bacteria. PLoS Pathog. 2014 Oct 2;10(10):e1004405.

Mutreja A, Kim DW, Thomson NR, Connor TR, Lee JH, Kariuki S, et al. Evidence for several waves of global transmission in the seventh cholera pandemic. Nature. 2011;477(7365):462-5.

O'Quigley J, Pepe M, Fisher L. Continual reassessment method: a practical design for phase 1 clinical trials in cancer. Biometrics. 1990;46(1):33-48.

Pezzoli L, Oral Cholera Vaccine Working Group of the Global Task Force on Cholera C. Global oral cholera vaccine use, 2013-2018. Vaccine. 2019.

Qadri F, Ali M, Chowdhury F, Khan AI, Saha A, Khan IA, et al. Feasibility and effectiveness of oral cholera vaccine in an urban endemic setting in Bangladesh: a cluster randomised open-label trial. Lancet. 2015;386(10001):1362-71.

For Review Only

Qadri F, Ali M, Lynch J, Chowdhury F, Khan AI, Wierzba TF, et al. Efficacy of a single-dose regimen of inactivated whole-cell oral cholera vaccine: results from 2 years of follow-up of a randomised trial. Lancet Infect Dis. 2018;18(6):666-74.

Qadri F, Chowdhury MI, Faruque SM, Salam MA, Ahmed T, Begum YA, et al. Peru-15, a live attenuated oral cholera vaccine, is safe and immunogenic in Bangladeshi toddlers and infants. Vaccine. 2007;25(2):231-8.

Qadri F, Chowdhury MI, Faruque SM, Salam MA, Ahmed T, Begum YA, et al. Randomized, controlled study of the safety and immunogenicity of Peru-15, a live attenuated oral vaccine candidate for cholera, in adult volunteers in Bangladesh. J Infect Dis. 2005;192(4):573-9.

Qadri F, Wierzba TF, Ali M, Chowdhury F, Khan AI, Saha A, et al. Efficacy of a Single-Dose, Inactivated Oral Cholera Vaccine in Bangladesh. N Engl J Med. 2016;374(18):1723-32.

Reimer AR, Van Domselaar G, Stroika S, Walker M, Kent H, Tarr C, et al. Comparative genomics of Vibrio cholerae from Haiti, Asia, and Africa. Emerg Infect Dis. 2011;17(11):2113-21.

Richie EE, Punjabi NH, Sidharta YY, Peetosutan KK, Sukandar MM, Wasserman SS, et al. Efficacy trial of single-dose live oral cholera vaccine CVD 103-HgR in North Jakarta, Indonesia, a cholera-endemic area. Vaccine. 2000;18(22):2399-410.

Rui H, Ritchie JM, Bronson RT, Mekalanos JJ, Zhang Y, Waldor MK. Reactogenicity of live-attenuated Vibrio cholerae vaccines is dependent on flagellins. Proc Natl Acad Sci U S A. 2010;107(9):4359-64.

Safa A, Nair GB, Kong RY. Evolution of new variants of Vibrio cholerae O1. Trends Microbiol. 2010;18(1):46-54.

Satchell KJ, Jones CJ, Wong J, Queen J, Agarwal S, Yildiz FH. Phenotypic Analysis Reveals that the 2010 Haiti Cholera Epidemic Is Linked to a Hypervirulent Strain. Infect Immun. 2016;84(9):2473-81.

Sit B, Zhang T, Fakoya B, Akter A, Biswas R, Ryan ET, et al. Oral immunization with a probiotic cholera vaccine induces broad protective immunity against Vibrio cholerae colonization and disease in mice. PLoS Negl Trop Dis. 2019;13(5):e0007417.

Son MS, Megli CJ, Kovacikova G, Qadri F, Taylor RK. Characterization of Vibrio cholerae O1 El Tor biotype variant clinical isolates from Bangladesh and Haiti, including a molecular genetic analysis of virulence genes. J Clin Microbiol. 2011;49(11):3739-49.

Sow SO, Tapia MD, Chen WH, Haidara FC, Kotloff KL, Pasetti MF, et al. Randomized, Placebo-Controlled, Double-Blind Phase 2 Trial Comparing the Reactogenicity and Immunogenicity of a Single Standard Dose to Those of a High Dose of CVD 103-HgR Live Attenuated Oral Cholera Vaccine, with Shanchol Inactivated Oral Vaccine as an Open-Label Immunologic Comparator. Clin Vaccine Immunol. 2017;24(12).

Taylor RK, Kirn TJ, Meeks MD, Wade TK, Wade WF. A Vibrio cholerae classical TcpA amino acid sequence induces protective antibody that binds an area hypothesized to be important for toxin-coregulated pilus structure. Infect Immun. 2004;72(10):6050-60.

Taylor RK, Miller VL, Furlong DB, Mekalanos JJ. Use of phoA gene fusions to identify a pilus colonization factor coordinately regulated with cholera toxin. Proc Natl Acad Sci U S A. 1987;84(9):2833-7.

Vaxchora, package insert revised on November, 2020 https://www.fda.gov/media/128415/download

For Review Only

Waldor MK, Mekalanos JJ. Lysogenic conversion by a filamentous phage encoding cholera toxin. Science. 1996;272(5270):1910-4.

Weill FX, Domman D, Njamkepo E, Almesbahi AA, Naji M, Nasher SS, et al. Genomic insights into the 2016-2017 cholera epidemic in Yemen. Nature. 2019;565(7738):230-3.

Weill FX, Domman D, Njamkepo E, Tarr C, Rauzier J, Fawal N, et al. Genomic history of the seventh pandemic of cholera in Africa. Science. 2017;358(6364):785-9.

World Health O. Cholera vaccine: WHO position paper, August 2017 - Recommendations. Vaccine. 2018;36(24):3418-20.

For Review Only

Appendix 1 : Schedule of Assessments

	Screening	Screening	Inpatient period								Follow-up				
**Visit Number**	**1**	**1**	**2**								**3**	**4**	**5**	**6**	**7**
**Visit Day**	**D-56 to D-1**	**D-10 to D-1**	**D1**	**D2**	**D3**	**D4**	**D5**	**D6**	**D7**	**D8-d/c[d]**	**D15w**	**D29**	**D57**	**D85/ ET**	**D180**
**Visit window**	-	-	-	-	-	-	-	-	-	-	± 3d	± 5d	± 7d	± 14d	+ 28d
Informed consent	X														
Demography	X														
Medical History[a]	X														
Inclusion/Exclusion criteria	X		X												
Medication	X		Daily							Daily	X	X	X	X	X
Full physical exam	X														
Symptom-directed physical examination			Daily							Daily	X	X	X	X	X
Body weight and height	X														
Vital signs	X		Daily							Daily	X	X			
Clinical laboratory assessments[b]	X		X	X		X			X		X	X			
HIV testing	X														
Urinalysis	X														

For Review Only

	Screening	Screening	Inpatient period								Follow-up				
**Visit Number**	**1**	**1**	**2**								**3**	**4**	**5**	**6**	**7**
**Visit Day**	**D-56 to D-1**	**D-10 to D-1**	**D1**	**D2**	**D3**	**D4**	**D5**	**D6**	**D7**	**D8-d/c[d]**	**D15w**	**D29**	**D57**	**D85/ ET**	**D180**
Urine pregnancy test	X		X												
PanChol administration			X												
Doxycycline administration							X	X	X						
Blood sample for immunogenicity			X	X		X			X		X	X	X	X	X
Blood sample for proteomics/metabolomics			X	X		X			X						
Stool sample for culture		X[d]	Daily					X[e]	X[f]	X[f]					
Stool sample for exploratory endpoints		X[d]	Daily								X	X	X	X	X
Solicited AE			Daily												
Unsolicited AE			Daily							Daily	X	X			
MAAE, NOCMC, SAE			Daily							Daily	X	X	X	X	X

ET= Early Termination visit
AE= Adverse Event
MAAE= Medically Attended Adverse Event
NOCMC: New-onset chronic medical conditions
SAE= Serious Adverse Event

For Review Only

[a] Complete medical history will be taken at screening and any changes should be updated prior to dosing.
[b] Will include complete blood count with differential and biochemistry (creatinine, liver enzymes, electrolytes)
[c] If applicable. Participants will be discharged after the first negative stool culture
[d] If applicable
[e] No earlier than 7:00am, any stool sample may be cultured on Day 6.
[f] Any stool sample on Day 7 and Day 8 may be cultured.

For Review Only

Appendix 2: Genetic Alterations in PanChol

**Mutation**	**Rationale**
ΔCTXΦ	Attenuates by removing the genes encoding CT and the multifunctional toxin MARTX (*2*); protects against toxigenic reversion by preventing chromosomal integration of CTXΦ (*3*).
Δ*flaBDE*/Δ*flaAC*	Attenuates and reduces potential reactogenicity (*4*).
Δ*floR-strAB-sul2*/Δ*dfrA*	Prevents the dispersal of antibiotic resistance genes.
N900_11550: P*htpg-ctxB*	Constitutive expression of CtxB enables immune responses to B-subunit of cholera toxin that may protect against diarrheal disease caused by *V. cholerae* and ETEC (*5*)
lacZ: cas9-sgRNA_ctxA	Endonuclease targeting *ctxA* prevents toxigenic reversion.
hlyA	Removes suspected virulence factor (6).
wbeT^{S158F}	Stable Hikojima serotype (7).
Δ*recA*	Prevents homologous recombination- dependent gene acquisition

For Review Only

Appendix 3: Data Monitoring Committee / Data and Safety Monitoring Board

A Data Monitoring Committee (DMC) or Data and Safety Monitoring Board (DSMB) will be convened for safety monitoring of this research study. The following characteristics describe the DMC/DSMB convened for this study (Check all that apply):

☒ The DMC/DSMB is independent from the study team and study sponsor.

☒ A process has been implemented to ensure absence of conflicts of interest by DMC/DSMB members.

☒ The DMC/DSMB has the authority to intervene on study progress in the event of safety concerns, e.g., to suspend or terminate a study if new safety concerns have been identified or need to be investigated.

☒ Describe number and types of (i.e., qualifications of) members:

Safety oversight will be conducted by a DSMB that is an independent group with expertise to interpret data from this study and will monitor participant safety. The DSMB members will be separate and independent of study personnel participating in this study and should not have scientific, financial, or other conflicts of interest related to this study.

☒ Describe planned frequency of meetings:

The DSMB will review data following completion of the 48-hour safety data from the final participants of Module 1 and will conduct ad hoc reviews as appropriate when a halting rule is met or for immediate concerns regarding observations during this study. After each meeting, the DSMB will make recommendations on continuation of the study.

☒ DMC/DSMB reports with no findings (i.e., "continue without modifications") will be submitted to the IRB at the time of Continuing Review.

☒ DMC/DSMB reports with findings/modifications required will be submitted promptly (within 5 business days/7 calendar days of becoming aware) to the IRB as an Other Event.

For Review Only
